# Anomeric Triflates versus Dioxanium Ions: Different Product-Forming Intermediates from 3-Acyl Benzylidene Mannosyl and Glucosyl Donors

**DOI:** 10.1021/acs.joc.3c02262

**Published:** 2024-01-18

**Authors:** Wouter A. Remmerswaal, Hidde Elferink, Kas J. Houthuijs, Thomas Hansen, Floor ter Braak, Giel Berden, Stefan van der Vorm, Jonathan Martens, Jos Oomens, Gijsbert A. van der Marel, Thomas J. Boltje, Jeroen D. C. Codée

**Affiliations:** † Leiden Institute of Chemistry, Leiden University, Einsteinweg 55, Leiden 2300 RA, The Netherlands; ‡ Institute for Molecules and Materials, Radboud University, Heyendaalseweg 135, Nijmegen 6525 AJ, The Netherlands; § Institute for Molecules and Materials, FELIX Laboratory, Radboud University, Toernooiveld 7, Nijmegen 6525 ED, The Netherlands; ∥ Department of Chemistry and Pharmaceutical Sciences, Amsterdam Institute of Molecular and Life Sciences (AIMMS), Vrije Universiteit Amsterdam, De Boelelaan 1108, Amsterdam 1081 HZ, The Netherlands

## Abstract

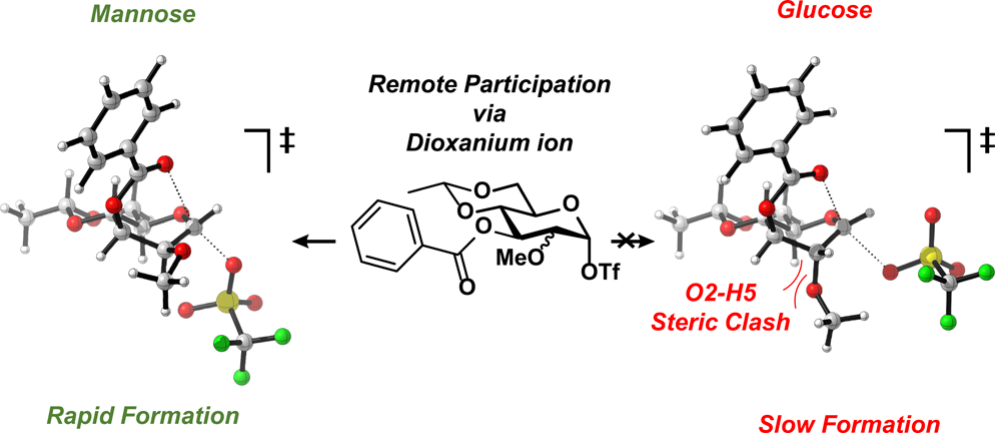

Minimal structural differences in the structure of glycosyl donors can have a tremendous impact on their reactivity and the stereochemical outcome of their glycosylation reactions. Here, we used a combination of systematic glycosylation reactions, the characterization of potential reactive intermediates, and in-depth computational studies to study the disparate behavior of glycosylation systems involving benzylidene glucosyl and mannosyl donors. While these systems have been studied extensively, no satisfactory explanations are available for the differences observed between the 3-*O*-benzyl/benzoyl mannose and glucose donor systems. The potential energy surfaces of the different reaction pathways available for these donors provide an explanation for the contrasting behavior of seemingly very similar systems. Evidence has been provided for the intermediacy of benzylidene mannosyl 1,3-dioxanium ions, while the formation of the analogous 1,3-glucosyl dioxanium ions is thwarted by a prohibitively strong flagpole interaction of the C-2-O-benzyl group with the C-5 proton in moving toward the transition state, in which the glucose ring adopts a *B*_2,5_-conformation. This study provides an explanation for the intermediacy of 1,3-dioxanium ions in the mannosyl system and an answer to why these do not form from analogous glucosyl donors.

## Introduction

Controlling the stereochemistry in glycosylation reactions is crucial for the assembly of oligosaccharides and glycoconjugates, which are required as molecular tools in glycobiological, glycomedical, and glycomaterial studies. However, full control over the stereochemical outcome of glycosylation reactions remains a major challenge. The wide variety of structurally different glycans has spurred the development of many different strategies to steer the stereochemical outcome of a glycosylation reaction. Neighboring group participation by acyl groups at C-2 is commonly used in the construction of 1,2-*trans* linkages. It is the most general means to control glycosylation stereoselectivity and (relatively) independent of the decoration pattern of the glycosyl donor.^1−3^ For the construction of 1,2-*cis*-glycosyl linkages, no general strategy is available,^4−6^ and translating methodology from one donor to another is often very challenging,^7^ if not impossible. The Crich β-mannosylation methodology, in which a 4,6-*O*-benzylidene protecting group is used in conjunction with nonparticipating protecting groups at the C-2 and C-3 functionalities, has become one of the most powerful means to construct 1,2-*cis*-mannosyl linkages.^8,9^ Strong evidence for the intermediacy of mannosyl α-triflates as product forming intermediates in glycosylations following an S_N_2-type mechanism has accumulated.^10^ However, the method is sensitive to changes in the protecting group pattern, and when a C-3-acyl group is present, the donors give rise to highly stereoselective α-mannosylation reactions.^11−13^ In contrast, benzylidene protected glucosyl donors provide mixtures of α/β-products with the anomeric product ratio critically hinging on the nucleophilicity of the acceptor.^11,14,15^ The stereoselectivity of these glucosylations appears to be relative insensitive to changes in the protecting group at C-3,^16^ although systematic studies have not been reported to dissect the influence of C-3 ester groups. There has been significant debate as to the origin of the high α-selectivity in the 3-acyl benzylidene mannose case.^17−19^ While Crich and co-workers have initially postulated long-range participation as a reasonable explanation for this stereoselectivity,^20^ they have more recently argued strongly against the possible formation of the 1,3-dioxanium ions as this would require the formation of a highly strained dioxanium ion from a C-3-acyl rotamer that is hardly populated requiring an intramolecular substitution reaction on a high-energy ring conformer.^21−23^ Rather they argued that the C-3-ester destabilizes the anomeric triflate, promoting reactions through an oxocarbenium ion intermediate.^22,23^ To investigate the formation of 1,3-mannosyl dioxanium ions, we recently investigated these glycosylation reactions with chemical exchange saturation transfer (CEST) NMR. We have forwarded spectroscopic evidence for the generation of 1,3-dioxanium ions from 3-acyl-4,6-benzylidene mannosyl α-triflates that can account for the formation of the observed α-products.^24−29^ With these CEST-NMR studies, we have not been able to detect the corresponding glucosyl 1,3-dioxanium ions.

To unravel the origin of the contrasting behavior of the 3-acyl benzylidene mannosyl and glucosyl donors, we here report a combination of systematic glycosylation reactions, using a series of alcohol nucleophiles of gradually changing nucleophilicity, with gas-phase IR spectroscopy to study the potential formation of the 1,3-dioxanium ions and computational experiments investigating different reaction paths of the anomeric triflates.^30^ Combined with our previous NMR studies^29^ that have revealed the formation of mannosyl 1,3-dioxanium ions and the absence of these species from the corresponding glucosyl systems, a picture emerges on the diverging reaction pathways followed in the mannose and glucose systems. The computational studies pinpoint the structural features responsible for the contrasting behavior of the mannosyl and glucosyl donors and illuminate the delicate balancing act between the various reactive intermediates. The study explains why long-range participation can take place in one donor (*i.e.*, the mannose system) but fails to control stereoselectivity in closely related systems (*i.e.*, the glucose donors).

## Results and Discussion

First, we established acceptor reactivity-stereoselectivity trends for the benzylidene mannosyl and glucosyl donors using a series of model acceptors of gradually increasing nucleophilicity (*i.e.*, 2,2,2-trifluoroethanol, TFE; 2,2-difluoroethanol, DFE; 2-fluoroethanol, MFE; ethanol, EtOH), in a set of preactivation glycosylation reactions,^31,32^ in which the glycosyl triflates are generated prior to the addition of the acceptors.^10^ The results of these model glycosylation reactions are summarized in Table 1b. As previously reported,^11,14,15^ the stereoselectivity of the glycosylation reactions of the 2,3-di-*O*-benzyl benzylidene glucose donor **1** gradually shifts from α- to β-stereoselectivity as the nucleophilicity of the acceptor increases. Conversely, the reactions of the 2,3-di-*O*-benzyl benzylidene mannose donor proceeded with β-selectivity, irrespective of the reactivity of the nucleophile. Installation of a benzoate group on the C-3 of the glucosyl donor had no effect on the stereochemical outcome of the glycosylation reactions. However, a tremendous shift in stereoselectivity was observed for the glycosylations of the mannosyl donors, providing exclusively the α-product, in line with previous experiments with a 3-*O*-benzoyl-2,4,6-tri-*O*-benzyl mannosyl donor. Similar to the glycosylations of the corresponding 2,4,6-tri-*O*-benzyl donors, the stark contrast between the mannose and glucose series may be related to the formation of a dioxanium ion intermediate. As mentioned above, we could detect the mannosyl dioxaniums in solution using CEST-NMR, while the glucosyl dioxanium ions were not observed.

**Table 1:**
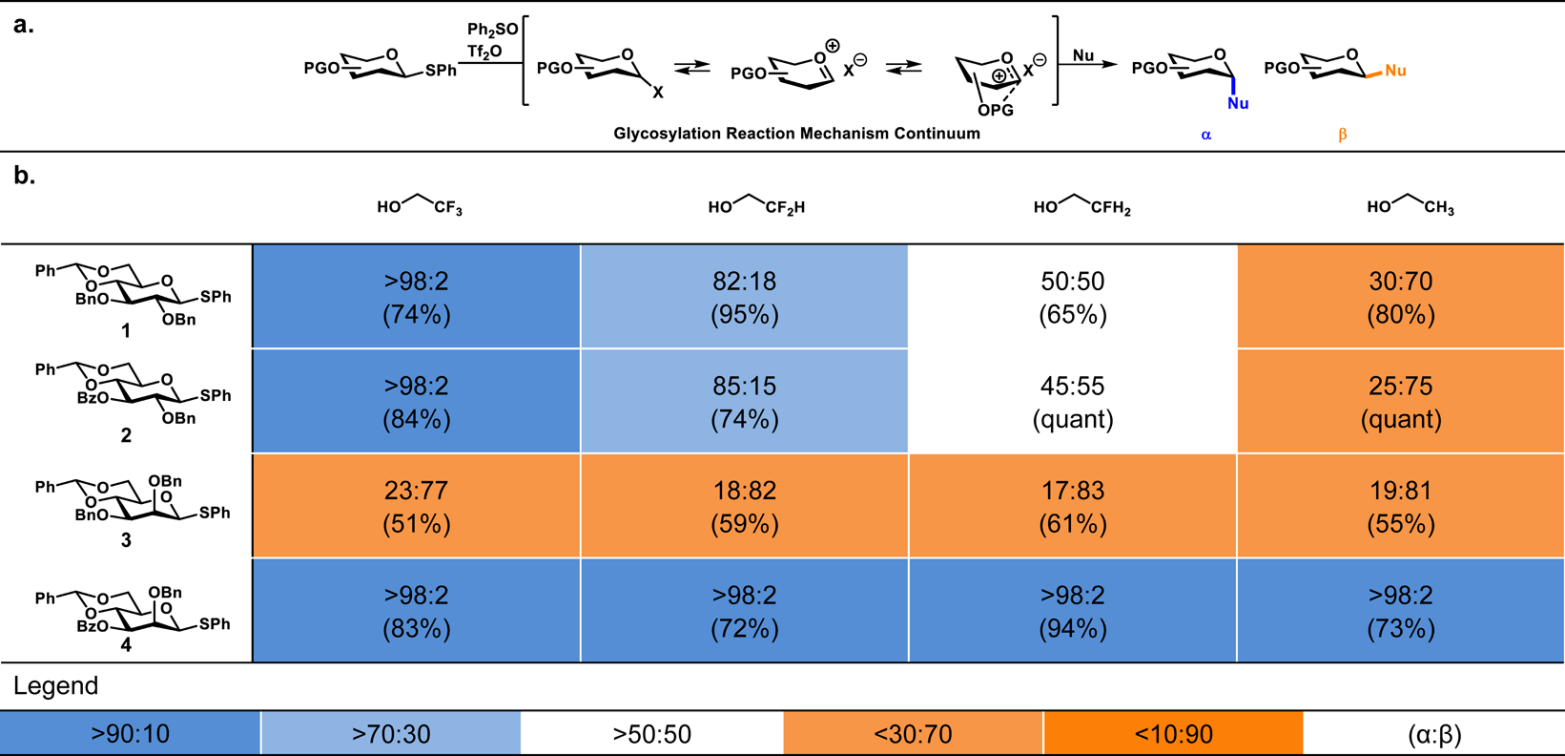
Model Glycosylation Reactions^a^

a The stereoselectivity of the reaction is expressed as α/β and based on ^1^H-NMR of purified α/β-product mixtures. Blue-colored cells represent α-selectivity, while orange-colored cells represent β-selectivity. The percentage given between brackets represents the yield after purification by column chromatography. Preactivation-based glycosylation conditions: donors **1**–**4** (1 equiv), Tf_2_O (1.3 equiv), Ph_2_SO (1.3 equiv), TTBP (2.5 equiv), DCM (0.05 M), –80 to −60 °C, then add nucleophile (2 equiv) at −80 °C, and allow to warm to −40 °C.

**Figure 1:**
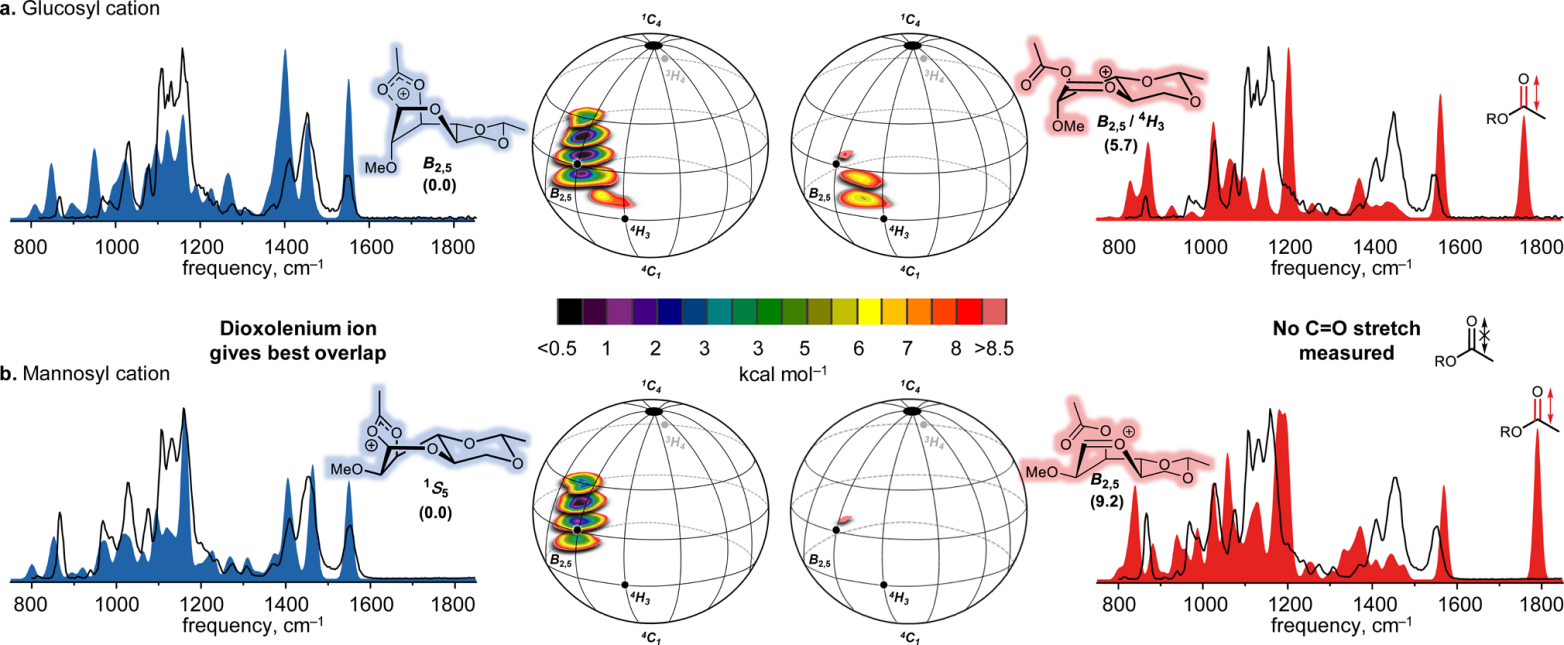
Conformational energy landscapes (CEL) and subsequent IR ion spectra of the 2-*O*-methyl-3-*O*-acetyl-4,6-*O*-ethylidene-glucosyl (a) and the -mannosyl cation (b). For the CELs, two acetyl ester rotamers (the oxocarbenium and dioxanium ions) were considered for all computed glycosyl cations generating two separate CEL maps. All energies are computed as B3LYP/6-311G(d,p) and expressed as the gas-phase Gibbs free energy at 298.15 K in kcal mol^–1^. The computed IR-spectra (filled) are compared with the measured IR-ion spectrum (black line) of the oxocarbenium or dioxanium ion.

To further investigate this contrast between the mannosyl and glucosyl donors, we investigated the stability of the 1,3-dioxanium ions using ion IRIS^33^ and computational chemistry (Figure 1a) as we have previously found the relative stability of the dioxanium ions with respect to the parent oxocarbenium ions to correlate to the stereoselectivity of the acylated glycosyl donors.^26^ To aid in spectroscopic elucidation, we used 2,3-di-*O*-methyl-4,6-ethylidene glucose (**5**) and mannose (**6**) donors. To obtain the cations of the sulfoxide donors, the proton adducts were generated by electrospray ionization (ESI+) and isolated in a Bruker AmaZon Speed ion trap (MS spectra can be found in Figures S1 and S2).^34^ Subsequently, the sulfoxide leaving group was expelled by collision induced dissociation (CID) to generate the glycosyl cations. An IR spectrum of the isolated cations was measured using the free-electron laser FELIX^35^ in the 600–1900 cm^–1^ range by monitoring the wavelength-dependent IR multiple photon-induced dissociation (IRMPD) yield.^36^

The relative stability of the cations was assessed through a density functional theory (DFT) protocol, in which the conformational energy landscape (CEL) maps for these cations were generated.^26,37−39^ This method maps the energy of the glycosyl cations as a function of their shape by probing the complete conformational space that these cations can occupy. We plotted the relative stability of the dioxanium ion versus the oxocarbenium ion for the glucosyl (Figure 1a) and the mannosyl (Figure 1b) cations. The CEL maps show that both the glucosyl and mannosyl 1,3-dioxanium ions are significantly more stable than their oxocarbenium ion counterparts, with a larger difference being found for the mannosyl ions (+9.2 kcal mol^–1^ vs +5.7 kcal mol^–1^). Furthermore, the conformational space available to the glucosyl and the mannosyl cations is restricted, and the 1,3-dioxanium ions preferentially take up a (skew)boat-like conformation, *i.e.*, *B*_2,5_ and ^1^*S*_5_ for the glucosyl and mannosyl dioxanium ions, respectively, in line with previous observations.^2,40^ For the mannosyl cation, the CEL map diverges significantly from the CEL map of the 3-*O*-benzoyl-2,4,6-tri-*O*-benzyl-mannosyl 1,3-dioxanium cation, which adopts a ^1^*C*_4_ conformation. In contrast, the conformation of the glucosyl 1,3-dioxanium cation (*B*_2,5_) is relatively similar to that of its 4,6-dibenzylated counterpart. The restriction of the conformational space is also apparent for the mannosyl oxocarbenium ion, which prefers to adopt a *B*_2,5_ conformation. The conformational restriction enforced by the benzylidene ring, thus, prevents the benzylidene mannosyl 1,3-dioxolenium ion to adopt the electronically most-favorable geometry (the ^1^*C*_4_ conformation), rendering the formation of the 3-*O*-benzoyl benzylidene mannosyl 1,3-dioxanium more difficult than the formation of its 4,6-dibenzyl counterpart, explaining the results we previously obtained in our CEST-NMR studies.^29,41^

The identified lowest energy conformers of the dioxanium and oxocarbenium cations were subsequently used to compute reference IR-spectra to aid in interpretation of the experimentally obtained spectra.^26,42^Figure 1a,b shows the computed spectra (filled) and the experimental spectra (black line) to confirm the formation of the dioxanium ions, as indicated by a characteristic dioxanium −[C–O]^+^ stretch (∼1550 cm^–1^) and the absence of the oxocarbenium [C1=O5]^+^ (∼1565 cm^–1^) and benzoyl C=O (∼1775 cm^–1^) stretches. Overall, these results show that in the gas phase and in the absence of counterions, both the 3-acyl glucosyl and mannosyl donors can form bridged dioxanium ions. The fact that the dioxanium ions can form from both the glucosyl and mannosyl donors upon ionization in the gasphase, but that only the mannosyl dioxanium ions form in solution, as judged from the CEST-NMR^22^ and, indirectly, by the stereochemical outcome of the glycosylation reactions (Table 1), indicates that the pathways for the formation of these ions may diverge.

**Figure 2:**
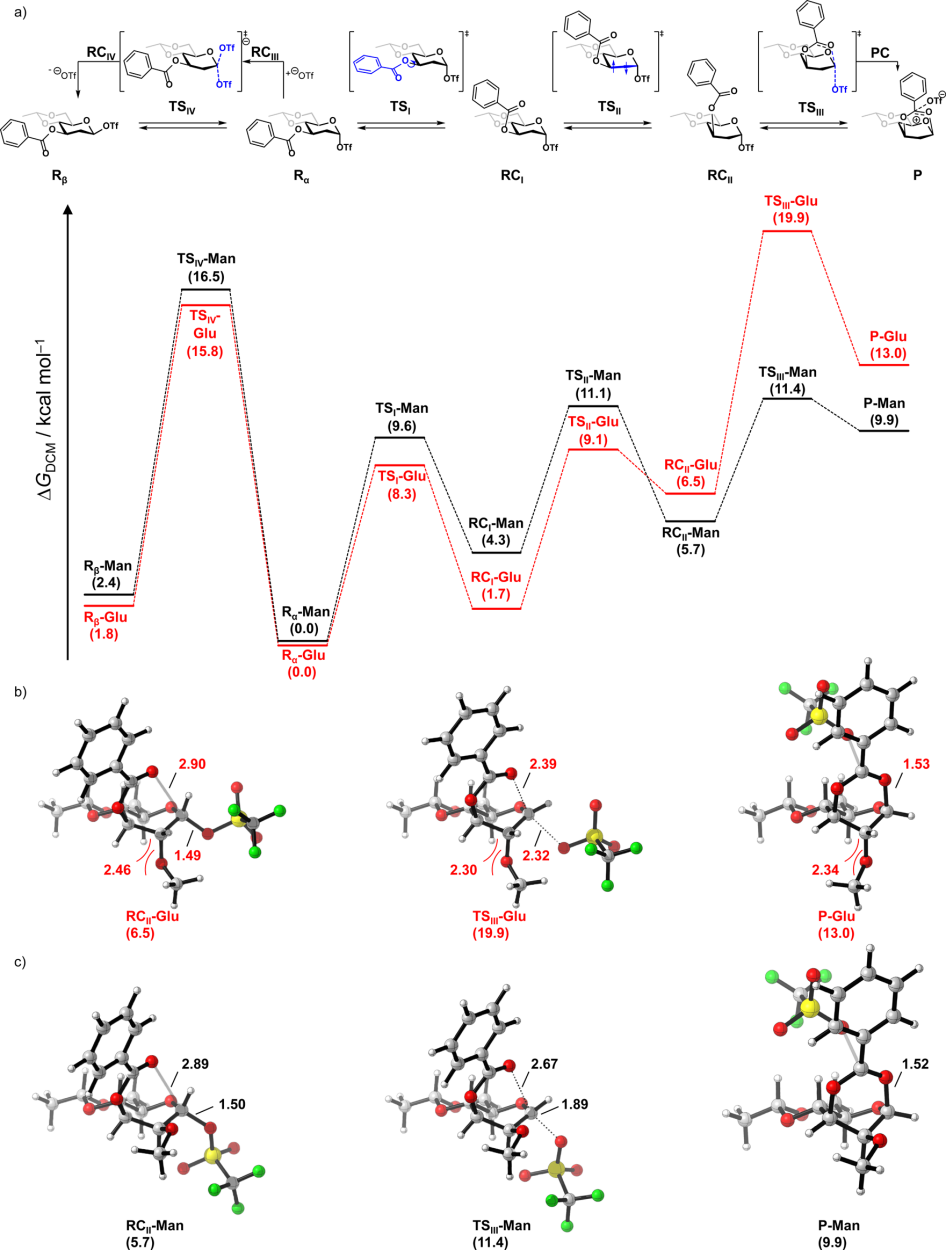
(a) The computed reaction profiles for the formation of the dioxanium ion and anomeric β-triflate from α-1-*O*-triflyl-2-*O*-methyl-3-*O*-benzoyl-4,6-*O*-ethylidene glucose (**R**_**α**_**-Glu**) and mannose (**R**_**α**_**-Man**). For clarity, the C-2 substituent is removed in all structures in (a). Gibbs free energies in dichloromethane (Δ*G*_DCM_, in kcal mol^–1^) are given relative to the anomeric α-triflate **R**_**α**_ for each separate potential energy surface. Structures of **RC**_**II**_, **TS**_**III**_, **PC**, and **P** for the (b) mannoside and (c) glucoside, visualized by Cylview.^48^ Distances are given in Å. Computed at PCM(CH_2_Cl_2_)-M06-2X/6-311++G(d,p)//PCM(CH_2_Cl_2_)-B3LYP-D3BJ/6-31+g(d), thermodynamic corrections were done at 213.15 K. See Table S2 for all data of the stationary points of the reaction profiles.

We, therefore, probed the pathways that lead to the formation of the dioxanium ions from the parent anomeric triflates using DFT computations (see Table S2 for all data of the stationary points of the reaction profiles). Figure 2a shows the reaction profiles for the formation of the dioxanium ions from 1-α-*O*-triflyl-2-*O*-methyl-3-*O*-benzoyl-4,6-*O*-ethylidene-glucose and mannose, each normalized to the individual anomeric α-triflate intermediate. The formation of the dioxanium ion consists of three consecutive steps starting from the most stable conformation of 1-α-*O*-triflyl-2-*O*-methyl-3-*O*-benzoyl-4,6-*O*-ethylidene-glycoside/mannoside (**R**_**α**_). The C-3 benzoate ester group first rotates around the H3–C3–O3–C_Benzoyl_ bond (**TS**_**I**_), transitioning the H3–C_Benzoyl_ relation from a *syn* toward an *anti*-orientation, to form an intermediate in which the benzoate ester is positioned above the ring (**RC**_**I**_). This transformation proceeds with a significant energy barrier, as previously postulated by Crich and is somewhat more difficult for the mannoside (+9.6) than for the glucoside (+8.3), as a result of torsion between the mannosyl benzoate ester and the axial C-2 group. The conformation of the ring then changes via **TS**_**II**_ to give a *B*_2,5_ conformation (**RC**_**II**_). The order of these two events was also changed (chair to boat transition before benzoate rotation), but this provided higher energy pathways (see Figure S3). Finally, the benzoate ester displaces the anomeric triflate (**TS**_**III**_, Figure 2b,c) to form the triflate anion stabilized dioxanium ion (**PC**). This step requires significantly more energy for the glucoside (+19.9) than for the mannoside (+11.4 kcal mol^–1^). Notably, for the mannoside,the height of this transition state (**TS**_**III**_**-man**) is similar to the **TS**_**II**_, indicating that expulsion of the triflate is rather favorable when the mannoside adopts the correct geometry. Repositioning of the triflate toward the carbonyl carbon of the benzoate group then forms the final triflate stabilized dioxanium ion (**P**), in which the alpha face of the dioxanium ion is available for nucleophilic attack. Similar to the **PC** and the solvent separated dioxanium ion (Table S2), the **P-man** (+9.9) is much more stable than the **P-glu** (+13.0).

Conversely, for the formation of the glucosyl dioxanium ion, expulsion of the anomeric triflate presents the highest overall barrier on the potential energy surface. To understand the differences in barrier height for **TS**_**III**_**man/glu**, we examined the structures surrounding the transition states: **RC**_**II**_, **TS**_**III**_, **PC**, and **P**. From the computed reaction profiles, the relative Gibbs free energy of **RC**_**II**_**-glu** is slightly higher than that of **RC**_**II-**_**man**, while the energies of **PC-man** and **PC-glu** are very different. Thus, the difference in energy between **TS**_**III**_**-man** and **TS**_**III**_**-glu** develops in going toward the transition state. When the conformations of **RC**_**II**_, **TS**_**III**_, **PC**, and **P** are compared, we note that the conformations of the glucose and mannose stationary points are virtually identical (*i.e.*, *B*_2,5_-like) in this part of the reaction profiles, and that during the transitioning between these points only minimal conformational changes occur. As the energy difference does not originate from deviating ring conformations, we compared the interactions of the ring substituents in the gluco and manno systems. In the *B*_2,5_ conformers, flagpole interactions of the axial C-2 and C-5 substituents are a major contributor to the ring strain. For the mannoside, this represents an interaction between two protons, while in the glucoside, it is a more severe oxygen–hydrogen interaction. In going to **TS**_**III**_, the C-2 and C-5 substituents are brought closer together (see Figure 2b,c), to forge the bond between C-1 and the benzoyl carbonyl, increasing the flagpole interactions, leading to a significantly higher energy penalty for the glucoside than for the mannoside. In addition, the triflate leaving group in the mannoside can be positioned in a more favorable orientation, allowing interaction between the triflate and the H-2 and H-5 protons. For the glucoside, this orientation would lead to unfavorable electrostatic repulsion between the pseudo axial triflate and the C-2 oxygen substituent. Instead, the glucose triflate avoids this steric clash through a different orientation, in which there is less interaction with the O-2. As a result, the transition state for the glucose system is significantly later with the triflate being further away from the positive charge, leading to an increase in energy.^43^

Finally, we probed the pathway for the formation of the anomeric β-triflate (**R**_**β**_) from its α-anomer. This in situ anomerization scheme has been forwarded to account for the formation of the α-glucosyl products, while it has been excluded for the mannosyl case because of the presumed instability of the β-mannosyl triflate. Indeed, Asensio and co-workers have previously been able to detect the β-glucosyl triflate by NMR spectroscopy of an ^13^C-labeled glycosyl triflate.^44^ We have also been able to detect the elusive β-mannosyl triflate by ^1^H/^19^F CEST,^29^ urging us to revisit the anomerization reaction in both cases. As shown in the reaction profiles for the glucosyl and mannosyl triflates (Figure 2a, left pathways), the activation energy (**TS**_**IV**_) for the formation of the anomeric β-triflate (**R**_**β**_) is slightly higher for the mannoside (+16.5 kcal mol^–1^) than that for the glucoside (+15.8 kcal mol^–1^). When the entire potential energy surface is regarded, the key distinction between the mannoside and the glucoside reaction profiles becomes apparent: for the glucosyl triflate, it is energetically more favorable to form the β-triflate (+15.8 kcal mol^–1^) than the dioxanium ion from the α-triflate (+19.9 kcal mol^–1^), while for the mannosyl triflate, the reverse is true (**TS**_**IV**_ and **TS**_**III**_ are, respectively, +16.5 kcal mol^–1^ and +11.4 kcal mol^–1^). The formation of the two different reactive intermediates accounts well for the dichotomy in the stereochemical outcome for the 3-*O*-benzoyl benzylidene mannose and glucose systems. The mannosyl dioxanium ion, detected by ^13^C CEST NMR,^29,41,45^ is responsible for the stereoselective formation of the α-mannosyl glycosylation products. In contrast, the glucosyl 1,3-dioxanium ion could be formed and characterized in the gas phase, but no evidence was found in solution. In this case, the generation of the mixtures of anomeric products can be explained using the anomeric triflates as product-forming intermediates, with reactive nucleophiles being capable of displacing the more prevalent α-glucosyl triflate and the weaker nucleophiles reacting with the more reactive β-triflates. As the pathway toward the glucosyl 1,3-dioxanium ion requires significantly more energy than the formation of the reactive β-triflate, as revealed by the computed potential energy surfaces, these ions do not partake in the reaction explaining the analogous behavior of the 3-*O*-benzyl and 3-*O*-benzoyl benzylidene glucosyl donors. Since the glucosylation reactions with the 2,3-di-*O*-benzyl benzylidene donors proceeded with identical stereoselectivity, it is likely that they proceed through a similar S_N_2-like mechanism.^46,47^

## Conclusion

In conclusion, our computational studies, combined with the series of systematic glycosylation reactions and spectroscopic analysis of reactive intermediates, have provided a detailed picture of the disparate glycosylation pathways of 2-*O*-benzyl-3-*O*-benzoyl-4,6-*O*-benzylidene mannosyl and glucosyl triflates. The study provides an explanation of how minimal changes in the donor structure (*i.e.*, the stereochemistry of a single stereocenter or changing a single remote benzyl group for a benzoate) impact the formation of different reactive intermediates. While it has previously been argued that 1,3-dioxanium ions cannot form from benzylidene mannosyl donors, spectroscopic evidence, both in the gas phase and in solution, and computational chemistry have shown these species to be favorable intermediates formed upon activation of the mannosyl donors. These species very well explain the “black-and-white” difference in stereochemical outcome of the 3-*O*-benzoyl vs the 3-*O*-benzyl benzylidene mannose donors, with the former providing solely α-mannosyl product and the latter predominantly the β-isomers. Furthermore, the computational studies in the glucose series support the intermediacy of a product-forming β-glucosyl triflate. The reaction pathways, reactive intermediates, and substituent effects defined in this study will help in the interpretation of future glycosylation results and the design of ever more effective stereoselective glycosylation chemistry to generate more and more complex oligosaccharides and glycoconjugates to fuel glycobiological, -medical, and -structural studies.

## Data Availability

The methods and data sets that support this article are available in this article and as online Supporting Information.

## Supporting Information Available

Ion spectroscopy methods, supplementary MS spectra, computational methods, supplementary reaction profiles, Cartesian coordinates of computed structures, organic synthesis procedures, and NMR spectra of new and selected compounds (PDF)
