# Supplementary material for: Anomeric Triflates versus Dioxanium Ions: Different Product-Forming Intermediates from 3-Acyl Benzylidene Mannosyl and Glucosyl Donors
Source: J Org Chem. 2024 Jan 18;89(3):1618–25. doi: 10.1021/acs.joc.3c02262 (PMC10845153; doi:10.1021/acs.joc.3c02262)
Supplement: Supplementary file 1 — jo3c02262_si_001.pdf [file jo3c02262_si_001.pdf]

Supplementary Information

"Anomeric Triflates versus Dioxanium Ions: Different Product-Forming Intermediates from 3-Acyl Benzylidene Mannosyl and Glucosyl Donors"

Wouter A. Remmerswaal,[a] Hidde Elferink,[b] Kas J. Houthuijs,[c] Thomas Hansen,[a,d] Floor ter Braak,[b] Giel Berden,[c] Stefan van der Vorm,[a] Jonathan Martens,[c] Jos Oomens,[c] Gijsbert A. van der Marel,[a] Thomas J. Boltje,[b] Jeroen D. C. Codée*[a]

[a] Leiden Institute of Chemistry, Leiden University
Einsteinweg 55, 2300 RA Leiden, The Netherlands
E-mail: jcodee@chem.leidenuniv.nl

[b] Radboud University, Institute for Molecules and Materials
Heyendaalseweg 135, 6525 AJ Nijmegen, The Netherlands

[c] Radboud University, Institute for Molecules and Materials, FELIX Laboratory
Toernooiveld 7, 6525 ED Nijmegen, The Netherlands

[d] Department of Chemistry and Pharmaceutical Sciences, Amsterdam Institute of Molecular and Life Sciences (AIMMS), Vrije Universiteit Amsterdam,
De Boelelaan 1108, 1081 HZ Amsterdam, The Netherlands

Table of contents

Supplementary Ion Spectroscopy Methods S4

Ion spectroscopy in a modified ion trap mass spectrometer S4

Simulation of IR spectra S4

Glycosyl cation MS spectra S5

Supplementary Figure 1. MS spectra of the generation of the 3-*O*-acetyl-4,6-*O*-ethylidene-2-*O*-methyl-glucosyl cation by expelling the leaving group from the ammonium adduct using CID. Fragment with m/z 245 was isolated (right) following CID of the ammonium adduct parent ion (m/z 288) (left). S5

Supplementary Figure 2. MS spectra of the generation of the 3-*O*-acetyl-4,6-*O*-ethylidene-2-*O*-methyl-mannosyl cation by expelling the leaving group from the ammonium adduct using CID. Fragment with m/z 245 was isolated (right) following CID of the ammonium adduct parent ion (m/z 288) (left). S5

Supplementary Computational Methods S6

Computational methods: generation of potential energy surfaces S6

Computational methods: generation of the computational energy landscapes S6

Importance of the order of conformational change to form RC_{II} S7

Computing reaction profiles of various other 3-*O*-acyloxy-donors. S7

Supplementary Table S1. Relative stability of the solvent separated dioxanium ions (P), and formation of the dioxanium ion through TSIII compared to each corresponding anomeric α-triflate**.** Gibbs free energies in dichloromethane (ΔG_{DCM}, in kcal mol^{-1}) are given relative to the anomeric α-triflate Rα. Computed at PCM(CH2Cl2)–M06-2X/6-311++G(d,p)//PCM(CH_2Cl_2)–B3LYP-D3BJ/6-31+g(d).. .... S8

Supplementary reaction profiles S8

Supplementary Figure 3. The computed reaction profiles for the formation of the dioxanium ion and anomeric β-triflate from: a) α-1-*O*-triflyl-2-*O*-methyl-3-*O*-benzoyl-4,6-*O*-ethylidene glucose (**$R_α$-Glu**) and b) mannose (**$R_α$-Man**). For clarity the C-2 substituent is removed in all chemdrawings. Gibbs free energies in dichloromethane (ΔG_{DCM}, in kcal mol^{-1}) are given relative to the anomeric α-triflate **$R_α$** for each separate potential energy surface. Computed at PCM(CH_2Cl_2)–M06-2X/6-311++G(d,p)//PCM(CH_2Cl_2)–B3LYP-D3BJ/6-31+G(d). See Supporting Information Table S2 for all data of the stationary points of the reaction profiles. S8

Supplementary Figure 4. The computed reaction profiles for the formation of the dioxanium ion and anomeric β-triflate from α-1-*O*-triflyl-2-*O*-methyl-3-*O*-benzoyl-4,6-*O*-ethylidene glucose (**$R_α$-Glu**), α-1-*O*-triflyl-2-deoxy-3-*O*-benzoyl-4,6-*O*-ethylidene glucose (**$R_α$-2dG**) and mannose (**$R_α$-Man**). For clarity the C-2 substituent is removed in all chemdrawings. Gibbs free energies in dichloromethane (ΔG_{DCM}, in kcal mol^{-1}) are given relative to the anomeric α-triflate **$R_α$** for each separate potential energy surface. Computed at PCM(CH_2Cl_2)–M06-2X//6-311++G(d,p)//PCM(CH_2Cl_2)–B3LYP-D3BJ/6-31+G(d). See Supporting Information Table S2 for all data of the stationary points of the reaction profiles. S9

Supplementary Figure 5. The computed reaction profiles for the formation of the dioxanium ion and anomeric β-triflate from α-1-*O*-triflyl-2,4,6-tri-*O*-methyl-3-*O*-benzoyl-glucose (**$R_α$-GluN**), and mannose (**$R_α$-ManN**). For clarity the C-2 substituent is removed in all chemdrawings. Gibbs free energies in dichloromethane (ΔG_{DCM}, in kcal mol^{-1}) are given relative to the anomeric α-triflate **$R_α$** for each separate potential energy surface. Computed at PCM(CH_2Cl_2)–M06-2X//6-311++G(d,p)//PCM(CH_2Cl_2)–B3LYP-D3BJ/6-31+g(d). See Supporting Information Table S2 for all data of the stationary points of the reaction profiles. S10

Supplementary Figure 6. The computed reaction profiles for the formation of the dioxanium ion and anomeric β-triflate α-1-*O*-triflyl-2-*O*-methyl-*O*-(4-*O*-methyl-benzoyl)-4,6-*O*-ethylidene-glucose (**$R_α$-GluPMBz**) and mannose (**$R_α$-ManPMBz**). For clarity the C-2 substituent is removed in all chemdrawings. Gibbs free energies in dichloromethane (ΔG_{DCM}, in kcal mol^{-1}) are given relative to the anomeric α-triflate

R_α for each separate potential energy surface. Computed at Computed at PCM(CH_2Cl_2)–M06-2X//PCM(CH2Cl2)–B3LYP-D3BJ/6-31+G(d). See Supporting Information Table S2 for all data of the stationary points of the reaction profiles. S11

Coordinates of computed structures S12

Supplementary Table S2. Cartesian coordinates (in Å), energies (*E*, *H* and qh-*G*, in Hartree), and number of imaginary vibrational frequencies (N_{imag}) of all stationary points and transition states, computed at PCM(CH_2Cl_2)–M06-2X/6-311++G(d,p)//PCM(CH_2Cl_2)–B3LYP-D3BJ/6-31+G(d). S12

Supplementary Organic Synthesis Methods S82

General experimental procedures S82

Preparation of the donors **1-4** S83

Supplementary scheme S1. Glucoside donor **2** synthesis. *Reagents and conditions:* a) 1. BnBr, NaH, DMF, 0 °C to RT; 2. DDQ, DCM:H_2O, **S1**: 82%; b) benzoyl chloride, pyridine, RT, **2**: quant. S83

Supplementary scheme S2. Mannoside donor **4** synthesis. *Reagents and conditions:* a) 1. Dibutyltin(IV) oxide, toluene, reflux; 2. 2-(bromomethyl)naphthalene, CsF, DMF, **S2**: 49%; b) BnBr, NaH, DMF, 0 °C to RT, **S3**: quant.; c) DDQ, DCM:H_2O, S4: 87%; d) benzoyl chloride, pyridine, RT, **4**: 69%,. S84

Model glycosylations **S5-S19** S86

Preparation of the IR donors **5-6** S91

Supplementary scheme S3. Glucoside donor **5** synthesis. *Reagents and conditions:* a) 1. 1,1-dimethoxyethane, camphorsulfonic acid, acetonitril, RT; 2. DDQ, DCM:H_2O, **S21**: 66%; b) acetic anhydride, pyridine, RT, **S22**: 63%; c) meta-chloroperoxybenzoic acid, DCM, **5**: quant. S91

Supplementary scheme S4. Mannoside donor **5** synthesis. *Reagents and conditions:* a) 1. 1,1-dimethoxyethane, camphorsulfonic acid, acetonitril, RT; 2. DDQ, DCM:H_2O, **S23**: 66%; b) acetic anhydride, pyridine, RT, **S24**: 63%; c) meta-chloroperoxybenzoic acid, DCM, **6**: quant. S92

NMR spectra of new and selected compounds S94

Supplementary References S164

Supplementary Ion Spectroscopy Methods

Ion spectroscopy in a modified ion trap mass spectrometer

The experimental apparatus is based on a modified 3D quadrupole ion trap mass spectrometer (Bruker, AmaZon Speed ETD) that has been coupled to the beam line of the FELIX infrared free electron laser (IR-FEL).[1,2] Ammonium adducts of each compound ($[M+NH_4]^+$) were generated by positive electrospray ionization from solutions of 10^{-6} M in 50:50 acetonitrile:water containing 2% ammonium acetate and introduced at 2 µl min^{-1}. The mass-isolated ions of interest were collisionally activated for 40 ms with an amplitude parameter of 0.2-0.4V to generate the relevant oxonium products. These fragment ions were subsequently mass isolated in an additional MS/MS stage and finally irradiated by the tunable mid-infrared beam. The FEL was tuned to provide 10 µs optical pulses at 10 Hz having 30–60 mJ pulse energy over the entire tuning range (bandwidth ~0.4% of the centre frequency). The pulse energy used for measurements was appropriately attenuated to avoid saturation of the signal. When a sufficient number of photons is absorbed, unimolecular dissociation occurs and generates frequency-dependent fragment ion intensities in the mass spectrometer. Relating the precursor ion intensity to the total fragmentation intensity in the observed mass spectra:

$$\text{yield} = -\ln\left(1 - \Sigma I(\text{fragment ions})/\Sigma I(\text{parent+fragment ions})\right)$$

for each frequency position generates an infrared spectrum (3 cm^{-1} step size).[3] The yield is obtained from several averaged mass spectra and is linearly corrected for laser power. The IR frequency is calibrated using a grating spectrometer.

Simulation of IR spectra

Vibrational spectra of the candidate geometries were generated using a previously reported workflow.[4,5] A SMILES code for the oxocarbenium and C-1,C-3 dioxanium ions served as input for the cheminformatics toolbox RDKit.[6] For each ion, 500 random conformations were generated using the distance geometry algorithm, which were minimized using the MMFF94 forcefield. The 40 most distinct geometries were selected based on the root-mean-squared distance between them. The geometries served as input for semi-empirical PM6 minimization and vibrational analysis with Gaussian16 Rev. C.01.[7] The resulting geometries were filtered for duplicates and subsequently minimized at the B3LYP/6-31++G(d,p) level, followed by vibrational analysis. The harmonic vibrational frequencies were scaled by 0.975 and broadened using a Gaussian function with a full-width at half-maximum of 25 cm^{-1}, as to match the experimental peak widths.

Glycosyl cation MS spectra

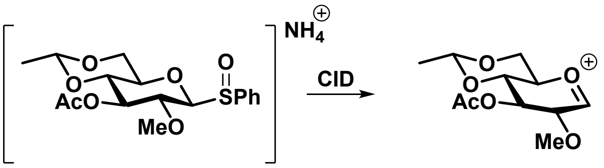

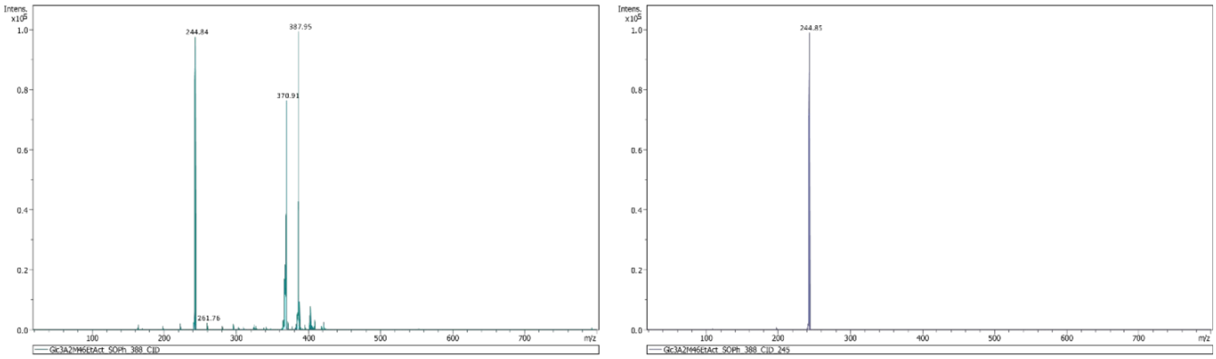

Supplementary Figure 1. MS spectra of the generation of the 3-*O*-acetyl-4,6-*O*-ethylidene-2-*O*-methyl-glucosyl cation by expelling the leaving group from the ammonium adduct using CID. Fragment with m/z 245 was isolated (right) following CID of the ammonium adduct parent ion (m/z 288) (left).

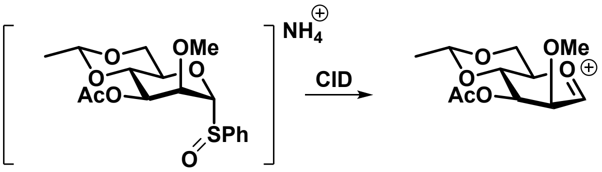

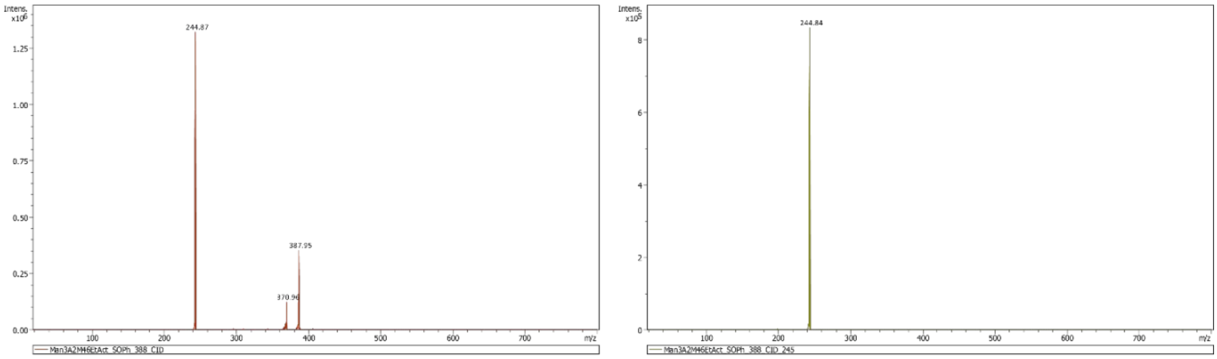

Supplementary Figure 2. MS spectra of the generation of the 3-*O*-acetyl-4,6-*O*-ethylidene-2-*O*-methyl-mannosyl cation by expelling the leaving group from the ammonium adduct using CID. Fragment with m/z 245 was isolated (right) following CID of the ammonium adduct parent ion (m/z 288) (left).

Supplementary Computational Methods

Computational methods: generation of potential energy surfaces

All computations were performed with Gaussian 09 Rev. D.01[8] at PCM(dichloromethane)-B3LYP-D3BJ-6-31+G(d) level of theory. Geometries were optimized without symmetry constraints. All calculated stationary points have been verified by performing a vibrational analysis, to be energy minima (no imaginary frequencies) or transition states (only one imaginary frequency). The character of the normal mode associated with the imaginary frequency of the transition state has been analyzed with an intrinsic reaction coordinate (IRC) calculation to ensure that it is associated with the reaction of interest.

Computational methods: generation of the computational energy landscapes

The initial structure for the conformational energy landscape (CEL) mapping of the six-membered glycosyl cation was optimized by starting from a ‘conformer distribution search’ option included in the Spartan 14[9] program by utilizing MM as the level of theory and MMFF[10] as the method. All generated geometries were re-optimized with Gaussian 09 Rev. D.01[8] by using B3LYP/6-311G(d,p), after which a vibrational analysis was computed to obtain the thermodynamic properties. The geometry with the lowest energy was selected as the starting point for the CEL. A complete survey of the possible conformational space was done by scanning three dihedral angles ranging from −60° to 60°, including the C1-C2-C3-C4 (D1), C3-C4-C5-O (D3) and C5-O-C1-C2 (D5). The resolution of this survey is determined by the step size which was set to 15° per puckering parameter, giving a total of 729 prefixed conformations per glycosyl cation spanning the entire conformational landscape. All other internal coordinates were unconstrained. Except when a C2-substituent was present on the oxocarbenium ring of interest, then the C2-H2 bond length was fixed based on the optimized structure to counteract rearrangements occurring for higher energy conformers. The 729 structures were optimized in the gas-phase with B3LYP/6-311G(d,p), after which a vibrational analysis was computed to obtain the thermodynamic properties. For this specific study two rotamers were taken into account, depending on the orientation of the C-2 acetyl protecting group (R1, C=O oriented towards C-1, in a way that makes C-1,C-3 dioxanium ion formation geometrically feasible. R2, C=O oriented away from C-1, in a way that makes C-1,C-3 dioxanium ion formation geometrically impossible.). For each rotamer CEL maps were separately computed and visualized. The $\Delta G^{T}_{gas,QH}$ were computed using the quasi-harmonic approximation in the gas phase according to the work of Truhlar using the goodvibes suite.[12,13] The quasi-harmonic approximation is the same as the harmonic oscillator approximation except that vibrational frequencies lower than 100 cm^{-1} were raised to 100 cm^{-1} as a way to correct for the breakdown of the harmonic oscillator model for the free energies of low-frequency vibrational modes. All optimized structures were checked for the absence of imaginary frequencies. To visualize the energy levels of the conformers on the Cremer-Pople sphere, we have generated slices dissecting the sphere that combine closely associated conformers. The OriginPro software was employed to produce the energy heat maps, contoured at 0.5 kcal/mol.[14] For ease of visualization, the Cremer-Pople globe is turned 180° with respect to its common representation.

Importance of the order of conformational change to form RC_{II}

In the main text only one order of conformational change to form $\mathbf{RC_{II}}$ is discussed: first the 2-*O*-benzoyl rotates, changing its orientation of the H3-$C_{Benzoyl}$ from syn to anti forming $\mathbf{RC_{I}}$. Subsequently, $\mathbf{RC_{I}}$ undergoes a conformational change from the starting ${}^{4}C_{1}$ conformation towards the B_{25} of $\mathbf{RC_{II}}$. Although other pathways can be identified, this specific order of conformational change is energetically the most favorable. The main determinant of this pathway is the rotation of the benzoate. This rotation preferentially occurs from the starting chair conformation. Rotation from the $B_{2,5}$ conformation is rather unfavorable for both the manno- and glucoside, yielding $\Delta\Delta G_{4C1\text{-}B25}$ of respectively +4.9 and +3.1 kcal mol^{-1} (Supplementary Figure 3). This due to a stronger interaction between the manno- and glucoside benzoate ester and the neighboring protons, as a result of the top side of the $B_{2,5}$ conformation being more crowded compared to the ${}^{4}C_{1}$ conformation.

Computing reaction profiles of various other 3-*O*-acyloxy-donors.

In our research employing ^{1}H-CEST-NMR we noted difficulties in observing the 3-*O*-benzoyl-2-*O*-benzyl-4,6-*O*-benzylidene mannose dioxanium ions,[15] even though these donors (Table 1) show remarkable stereoselectivity. This is not completely surprising, since ^{1}H-CEST-NMR has a quite narrow range for observable reaction rates. Replacing either the 3-*O*-benzoyl group with a 3-O-*p*-OMe-benzoyl (p-anisoyl) or the 4,6-*O*-benzylidene group with 4,6-di-*O*-mehtyl groups, lead to observable dioxanium ions. To corroborate these results, we computed the above-mentioned reaction profiles for the formation of the dioxanium ions from 1-α-*O*-triflyl-2,4,6-tri-*O*-methyl-3-*O*-benzoyl, 1-α-*O*-triflyl-2-*O*-methyl-3-*p*-*O*-benzoyl-4,6-*O*-ethylidene mannose (Supplementary figure 5 and 6). Variation of the protecting groups on C-3, C-4 and C-6 did not change the barrier heights for the formation of the dioxanium ion significantly. However, substitution of either the 3-*O*-benzoyl group with a 3-O-*p*-OMe-benzoyl (p-anisoyl) or the 4,6-*O*-benzylidene group with 4,6-di-*O*-mehtyl groups, lead to significant stabilization of the dioxanium ion **P** (Supplementary Table 1). These results match well with the observed ^{1}H-CEST-NMR results.

Supplementary Table S1. Relative stability of the solvent separated dioxanium ions (P), and formation of the dioxanium ion through TS_{III} compared to each corresponding anomeric α-triflate. Gibbs free energies in dichloromethane (ΔG_{DCM}, in kcal mol^{-1}) are given relative to the anomeric α-triflate R_{α}. Computed at PCM(CH_2Cl_2)–M06-2X/6-311++G(d,p)//PCM(CH_2Cl_2)–B3LYP-D3BJ/6-31+g(d).

**Dioxanium ion (P)**	**Relative dioxanium formation (TS_{III})** (ΔG_{DCM}, in kcal mol^{-1})	**Relative dioxanium stability (P)** (ΔG_{DCM}, in kcal mol^{-1})
2-*O*-methyl-3-O-benzoyl-4,6-*O*-ethylidene mannosyl	11.4	10.8
2-*O*-methyl-3-*O*-*p*-OMe-benzoyl-4,6-*O*-ethylidene mannosyl	11.2	8.1
2,4,6-tri-*O*-methyl-3-*O*-benzoyl mannosyl	9.7	8.8

Supplementary reaction profiles

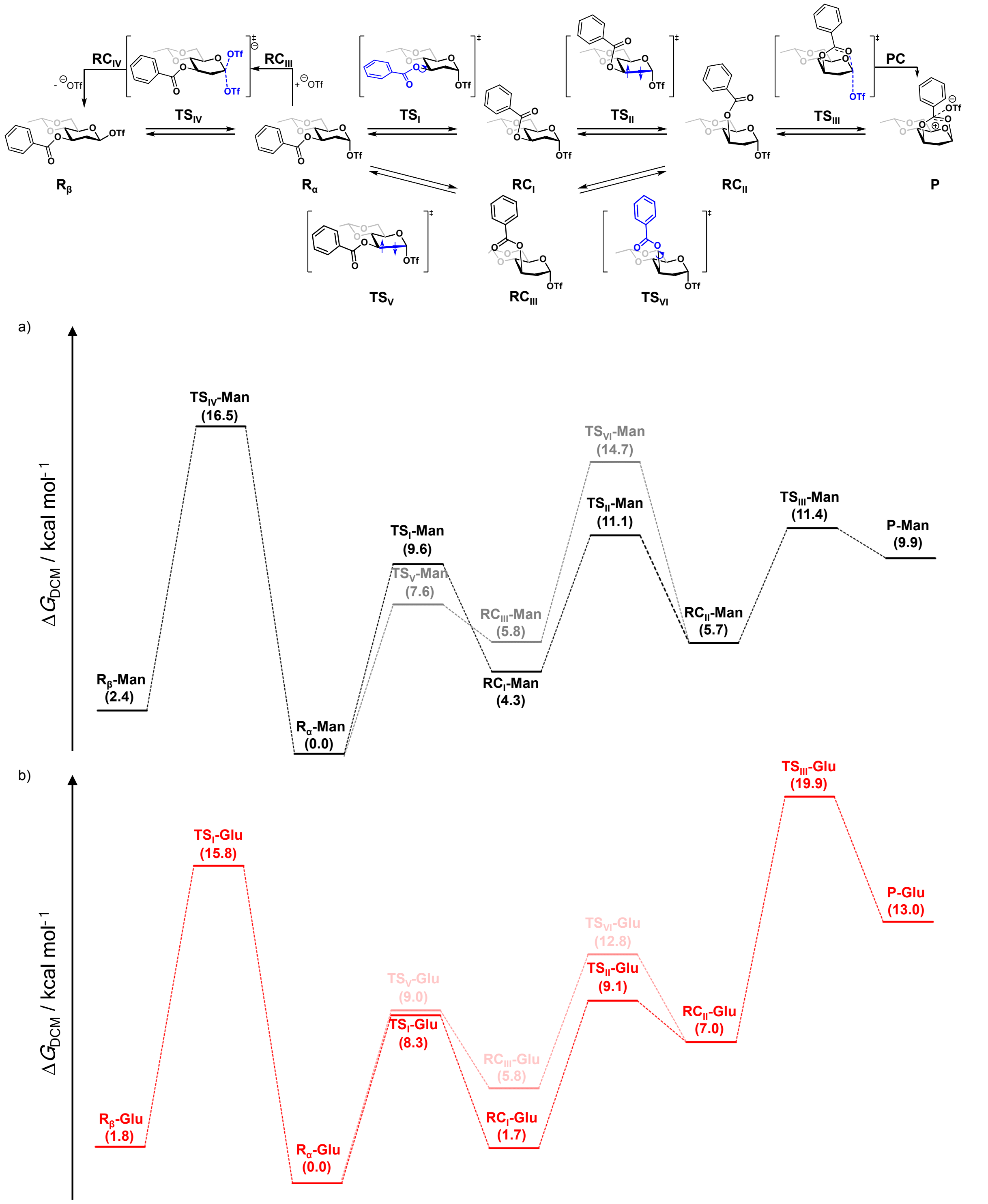

Supplementary Figure 3. The computed reaction profiles for the formation of the dioxanium ion and anomeric β-triflate from: a) α-1-*O*-triflyl-2-*O*-methyl-3-*O*-benzoyl-4,6-*O*-ethylidene glucose (**R_α-Glu**) and b) mannose (**R_α-Man**). For clarity the C-2 substituent is removed in all chemdrawings. Gibbs free energies in dichloromethane (ΔG_{DCM}, in kcal mol^{-1}) are given relative to the anomeric α-triflate **R_α** for each separate potential energy surface. Computed at PCM(CH_2Cl_2)–M06-2X/6-311++G(d,p)//PCM(CH_2Cl_2)–B3LYP-D3BJ/6-31+G(d). See Supporting Information Table S2 for all data of the stationary points of the reaction profiles.

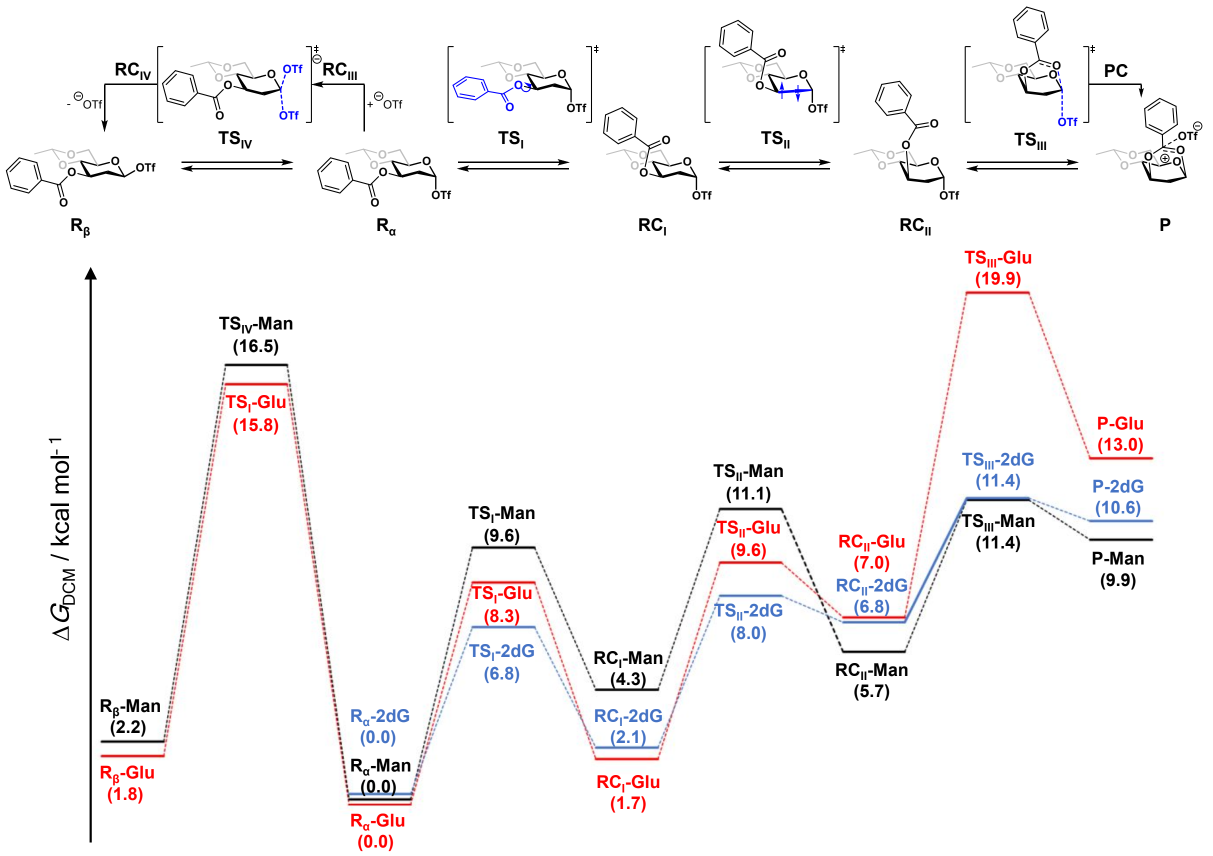

Supplementary Figure 4. The computed reaction profiles for the formation of the dioxanium ion and anomeric β-triflate from α-1-*O*-triflyl-2-*O*-methyl-3-*O*-benzoyl-4,6-*O*-ethylidene-glucose (**R$_\alpha$-Glu**), α-1-*O*-triflate-2-deoxy-3-*O*-benzoyl-4,6-*O*-ethylidene-glucose (**R$_\alpha$-2dG**) and α-1-*O*-triflyl-2-*O*-methyl-3-*O*-benzoyl-4,6-*O*-ethylidene-mannose (**R$_\alpha$-Man**). For clarity the C-2 substituent is removed in all chemdrawings. Gibbs free energies in dichloromethane (ΔG_{DCM}, in kcal mol^{-1}) are given relative to the anomeric α-triflate **R$_\alpha$** for each separate potential energy surface. Computed at PCM(CH2Cl2)–M06-2X//6-311++G(d,p)//PCM(CH2Cl2)–B3LYP-D3BJ/6-31+G(d). See Supporting Information Table S2 for all data of the stationary points of the reaction profiles.

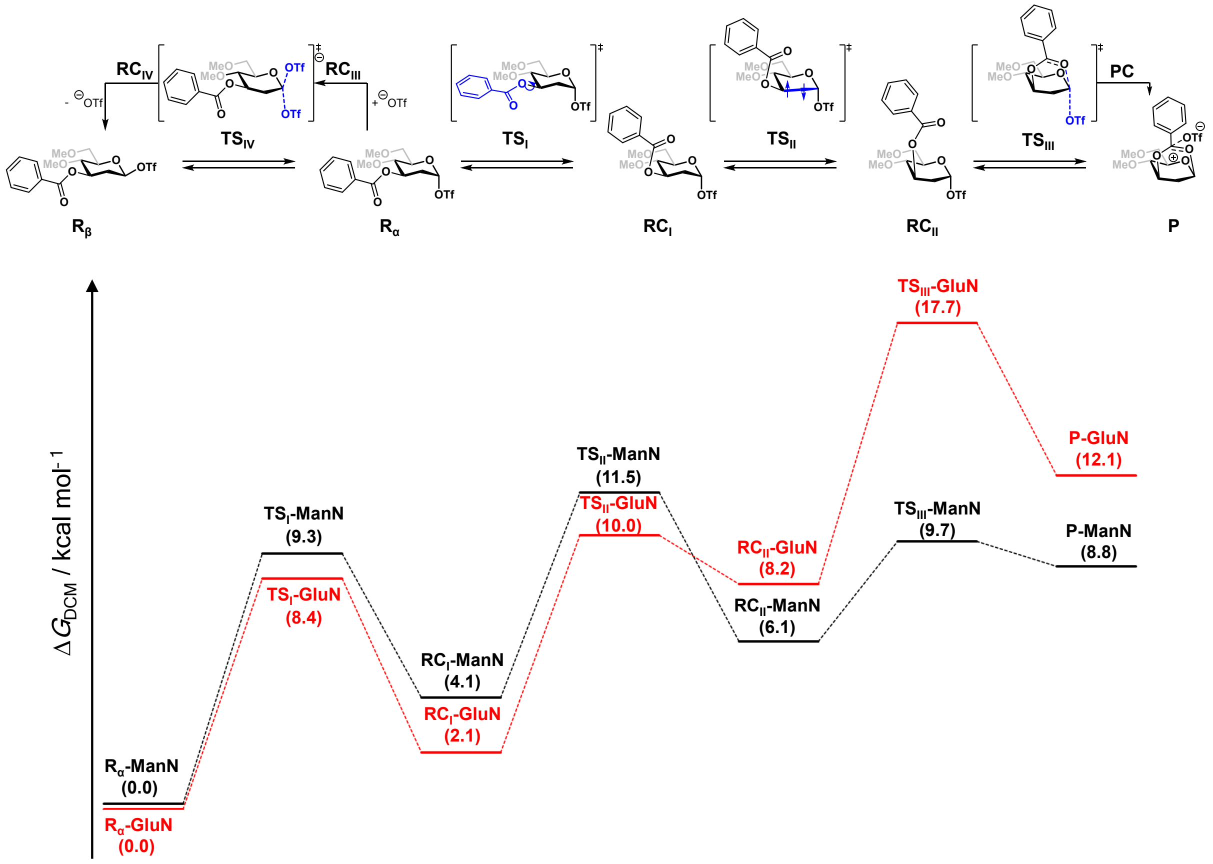

Supplementary Figure 5. The computed reaction profiles for the formation of the dioxanium ion and anomeric β-triflate from α-1-O-triflyl-2,4,6-tri-*O*-methyl-3-*O*-benzoyl-glucose (**R$_{\alpha}$-GluN**), and mannose (**R$_{\alpha}$-ManN**). For clarity the C-2 substituent is removed in all chemdrawings. Gibbs free energies in dichloromethane (ΔG_{DCM}, in kcal mol−1) are given relative to the anomeric α-triflate **R$_{\alpha}$** for each separate potential energy surface. Computed at PCM(CH_2Cl_2)–M06-2X//6-311++G(d,p)//PCM(CH_2Cl_2)–B3LYP-D3BJ/6-31+g(d). See Supporting Information Table S2 for all data of the stationary points of the reaction profiles.

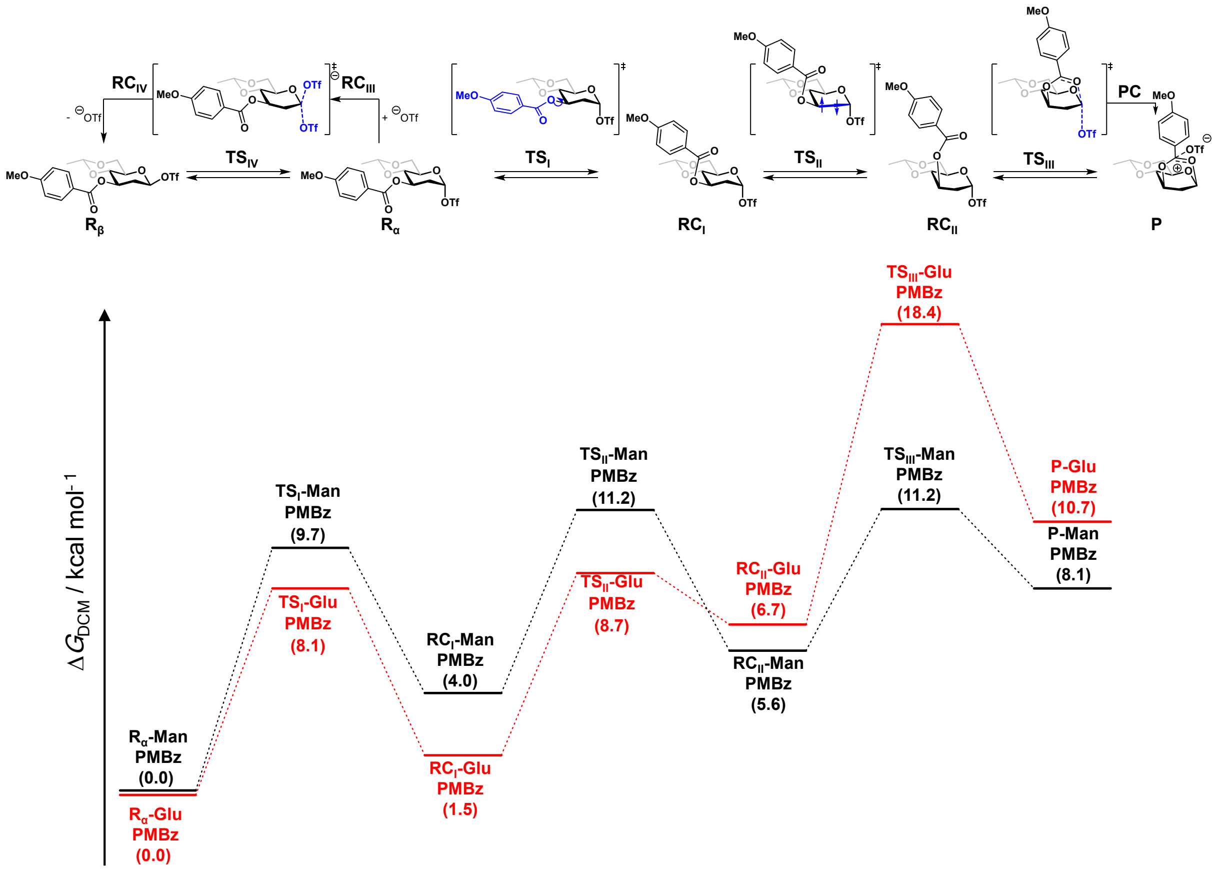

Supplementary Figure 6. The computed reaction profiles for the formation of the dioxanium ion and anomeric β-triflate α-1-*O*-triflyl-2-*O*-methyl-3-*O*-(4-*O*-methyl-benzoyl)-4,6-*O*-ethylidene-glucose (**R$_{\alpha}$-Glu**) and mannose (**R$_{\alpha}$-Man**). For clarity the C-2 substituent is removed in all chemdrawings. Gibbs free energies in dichloromethane (ΔG_{DCM}, in kcal mol^{-1}) are given relative to the anomeric α-triflate **R$_{\alpha}$** for each separate potential energy surface. Computed at Computed at PCM(CH_2Cl_2)–M06-2X//PCM(CH_2Cl_2)–B3LYP-D3BJ/6-31+G(d). See Supporting Information Table S2 for all data of the stationary points of the reaction profiles.

Coordinates of computed structures

Supplementary Table S2. Cartesian coordinates (in Å), energies (*E*, *H* and qh-*G*, in Hartree), and number of imaginary vibrational frequencies (N_{imag}) of all stationary points and transition states, computed at PCM(CH_2Cl_2)–M06-2X/6-311++G(d,p)//PCM(CH_2Cl_2)–B3LYP-D3BJ/6-31+G(d).

Trifluoromethanesulfonate
E = -961.585256
H = -961.553975
qh-*G* = -961.580315
N_{imag} = 0

O	1.445856	0.000000	-1.329367
S	0.000160	0.000000	-0.997635
O	-0.722959	1.252092	-1.328696
O	-0.722959	-1.252092	-1.328696
C	0.000138	0.000000	0.880533
F	0.629095	-1.089805	1.368136
F	0.629095	1.089805	1.368136
F	-1.258426	0.000000	1.367590

R_{β}-Glu (3-*O*-benzoyl-4,6-*O*-ethylidene-2-*O*-methyl-D-glucopyranose)
E = -2033.765922
H = -2033.382841
qh-*G* = -2033.431402
N_{imag} = 0

C	-1.003652	0.183112	-0.447208
C	0.800256	1.887478	-0.247615
C	-0.630040	1.521878	0.160930
C	0.036872	-0.879786	-0.066847
H	-1.082474	0.263521	-1.533762
O	-2.264935	-0.248571	0.082220
O	-1.514869	2.540471	-0.283718
H	0.868550	1.943478	-1.342914
C	1.144600	3.247051	0.346960
H	2.105974	3.616098	-0.014216
H	1.169187	3.184432	1.445048
O	0.150796	4.180391	-0.082817
C	1.450102	-0.348843	-0.358671
C	-1.165064	3.798677	0.290897
H	-1.199901	3.693412	1.388038
H	-0.675462	1.447732	1.259094
C	-2.132625	4.840553	-0.214652
H	-2.068178	4.911079	-1.304803
H	-1.892391	5.813653	0.222728
H	-3.153248	4.566702	0.066796
C	-3.295585	-0.442618	-0.778372
O	1.701352	0.893200	0.246787
S	3.848593	-1.490005	-0.380473
O	3.886239	-1.080663	-1.777053
O	4.280435	-2.800513	0.072591
C	4.856654	-0.227619	0.605295
F	4.663590	0.996558	0.122445
F	4.505207	-0.271216	1.890089
F	6.144550	-0.559190	0.480667
O	2.363246	-1.282058	0.244478
H	1.635700	-0.310261	-1.437863
O	-3.212859	-0.254268	-1.979391
C	-4.522098	-0.907204	-0.080307
C	-6.876092	-1.786321	1.145568
C	-4.554763	-1.118828	1.306783
C	-5.672595	-1.137633	-0.849489
C	-6.845614	-1.575792	-0.237429
C	-5.730817	-1.557515	1.915220

H	-3.665666	-0.940330	1.900320
H	-5.633850	-0.970325	-1.920663
H	-7.734351	-1.752964	-0.835951
H	-5.754115	-1.720880	2.988682
H	-7.790555	-2.127908	1.622395
O	-0.214701	-2.042151	-0.824477
C	-0.120526	-3.272752	-0.100342
H	-0.832701	-3.282206	0.734477
H	0.894309	-3.436896	0.277348
H	-0.379919	-4.061447	-0.808663

RC$_{IV}$-Glu (3-*O*-benzoyl-4,6-*O*-ethylidene-2-*O*-methyl-D-glucopyranose)
E = -2995.359497
H = -2994.943392
qh-*G* = -2995.001468
$\boldsymbol{N_{imag}}$ = 1

C	1.335901	-0.331116	-0.461794
C	-0.117540	-0.602276	-0.896739
C	-1.084498	-0.218615	0.230301
C	-0.631308	-0.858655	1.527850
C	0.810793	-0.442846	1.824159
H	1.559690	0.738296	-0.479365
H	-1.146635	0.865923	0.331214
H	-0.674692	-1.955564	1.427151
H	0.868782	0.648412	1.890215
H	-0.239434	-1.671479	-1.103895
O	1.664375	-0.910936	0.772838
O	-1.461267	-0.451834	2.608364
C	1.238579	-1.071474	3.142752
H	2.215807	-0.708945	3.466320
H	1.270630	-2.167497	3.049257
O	0.296522	-0.682035	4.146156
C	-1.035594	-1.055413	3.826048
H	-1.067224	-2.150591	3.693639
C	-1.946457	-0.587531	4.935350
C	-7.063031	-1.860654	-1.360378
C	-4.671507	-0.614335	-0.618718
C	-5.888524	-2.611888	-1.248687
C	-7.042759	-0.485787	-1.101214
C	-4.694053	-1.993092	-0.878761
C	-5.851097	0.136076	-0.731617
H	-5.819751	1.201140	-0.527634
H	-7.953910	0.099150	-1.187224
H	-7.991810	-2.345206	-1.648657
H	-5.903040	-3.679167	-1.450031
O	-2.372783	-0.750942	-0.129053
O	-0.446286	0.169886	-2.033043
O	0.290128	2.710515	0.942269
C	-0.296592	-0.492406	-3.288779
H	-0.842167	-1.445397	-3.292076
H	-0.727393	0.174892	-4.037830
H	0.757344	-0.672446	-3.528868
C	-3.423811	0.094931	-0.223570
O	-3.353285	1.292041	-0.008639
O	3.369644	-2.556161	-2.789013
H	-3.781892	-2.571964	-0.791572
S	1.469933	3.492293	0.498571
O	1.892938	4.578852	1.411358
O	2.572150	2.670632	-0.063145
C	0.827178	4.418718	-1.002427
F	-0.214663	5.208950	-0.674140

F	0.415780	3.566503	-1.960898
F	1.789472	5.203149	-1.532168
O	2.290337	-0.857431	-1.427639
S	2.650830	-2.427530	-1.530151
O	1.521622	-3.289464	-1.203116
C	3.960092	-2.659300	-0.163985
F	4.840850	-3.558157	-0.619783
F	4.586612	-1.511063	0.081411
F	3.402954	-3.125965	0.949272
H	-1.886150	0.500983	5.030522
H	-1.647744	-1.048043	5.881357

TS$_{IV}$-Glu (3-*O*-benzoyl-4,6-*O*-ethylidene-2-*O*-methyl-D-glucopyranose)
E = -2995.345454
H = -2994.930979
qh-*G* = -2994.989133
N_{imag} = 1

C	1.353827	0.116911	-0.566538
C	-0.126579	-0.020676	-0.976423
C	-1.068690	-0.089342	0.227189
C	-0.454067	-1.048806	1.223154
C	0.865050	-0.473016	1.723994
H	1.899283	0.924976	-1.042679
H	-1.230823	0.898979	0.655747
H	-0.280778	-2.016535	0.735073
H	0.673138	0.468149	2.243444
H	-0.236993	-0.962721	-1.527073
O	1.779712	-0.121184	0.650358
O	-1.282683	-1.238025	2.362624
C	1.509236	-1.474386	2.673893
H	2.412856	-1.073674	3.135987
H	1.744996	-2.409469	2.150332
O	0.568669	-1.705540	3.728976
C	-0.686438	-2.176837	3.258505
H	-0.522750	-3.116525	2.705918
C	-1.606936	-2.358782	4.440302
C	-6.950286	-1.905834	-1.502100
C	-4.649833	-0.666774	-0.506513
C	-5.694400	-2.478370	-1.728566
C	-7.057015	-0.713225	-0.778068
C	-4.544665	-1.862535	-1.233655
C	-5.910769	-0.094976	-0.281279
H	-5.977657	0.829950	0.281644
H	-8.031518	-0.267204	-0.601835
H	-7.843887	-2.387825	-1.888899
H	-5.610680	-3.403991	-2.290617
O	-2.309186	-0.628126	-0.252848
O	-0.507215	1.062209	-1.786495
O	0.508864	2.573091	1.027326
C	-0.107399	0.945058	-3.153042
H	-0.495262	0.014750	-3.589287
H	-0.536579	1.804856	-3.669518
H	0.983823	0.965654	-3.258974
C	-3.456612	0.028244	0.045233
O	-3.494989	1.062944	0.687014
O	2.466614	-3.313590	-2.687168
H	-3.569961	-2.303702	-1.406850
S	1.521216	3.538003	0.533319
O	2.266522	4.263881	1.587449
O	2.346577	3.048306	-0.600149
C	0.478318	4.885581	-0.255273

F	-0.359181	5.436275	0.646863
F	-0.264474	4.402617	-1.268915
F	1.259420	5.870140	-0.747939
O	2.181660	-1.064929	-1.694225
S	2.349067	-2.572849	-1.428986
O	1.439911	-3.072961	-0.389428
C	4.062931	-2.649375	-0.664700
F	4.378059	-3.930362	-0.417055
F	4.970657	-2.134172	-1.503858
F	4.098800	-1.966822	0.486653
H	-1.745546	-1.402919	4.954615
H	-1.176611	-3.082965	5.137793

RC$_{III}$-Glu (3-*O*-benzoyl-4,6-*O*-ethylidene-2-*O*-methyl-D-glucopyranose)
E = -2995.369735
H = -2994.953465
qh-*G* = -2995.011712
N$_{imag}$ = 0

C	1.461890	0.397553	0.076431
C	0.030851	0.538946	-0.466729
C	-0.990235	0.671594	0.663003
C	-0.758715	-0.465897	1.637737
C	0.681593	-0.415934	2.151044
H	2.170923	0.160172	-0.711887
H	-0.924594	1.642778	1.156500
H	-0.921909	-1.419170	1.115148
H	0.855723	0.534491	2.668336
H	-0.173305	-0.397897	-0.993384
O	1.612142	-0.548011	1.065898
O	-1.636681	-0.371842	2.755514
C	0.885633	-1.567676	3.127370
H	1.860641	-1.517394	3.615488
H	0.791812	-2.528820	2.602297
O	-0.101994	-1.457140	4.157161
C	-1.428291	-1.463210	3.646850
H	-1.580397	-2.398776	3.083368
C	-2.390342	-1.329829	4.802321
C	-6.871742	0.788062	-1.840913
C	-4.449556	1.251763	-0.523506
C	-5.846819	-0.161343	-1.907681
C	-6.686470	1.970299	-1.115521
C	-4.636924	0.066616	-1.251525
C	-5.478939	2.202429	-0.458946
H	-5.320224	3.114752	0.106304
H	-7.481511	2.708510	-1.062849
H	-7.812769	0.607469	-2.353173
H	-5.989183	-1.078769	-2.471320
O	-2.293289	0.528095	0.073462
O	-0.071882	1.643531	-1.338630
O	1.797282	1.745437	0.637706
C	0.023143	1.297264	-2.722898
H	-0.803802	0.636482	-3.012056
H	-0.042843	2.234675	-3.278177
H	0.973619	0.800825	-2.948783
C	-3.176465	1.543492	0.188344
O	-2.951834	2.572594	0.804230
O	0.407033	-3.831658	-3.052590
H	-3.839909	-0.666223	-1.301834
S	3.281459	2.323735	0.537185
O	3.538108	3.104160	1.738324
O	4.220101	1.320132	0.049670

C	3.039169	3.560071	-0.860239
F	2.053174	4.406855	-0.562882
F	2.750582	2.912880	-1.991688
F	4.178698	4.243569	-1.020156
O	1.616267	-1.859978	-2.096452
S	0.714751	-3.013081	-1.856411
O	-0.445178	-2.722120	-0.976763
C	1.761111	-4.172057	-0.812085
F	1.075011	-5.294520	-0.509098
F	2.878874	-4.537737	-1.472839
F	2.135637	-3.594336	0.345040
H	-2.207626	-0.390115	5.332714
H	-2.256686	-2.164910	5.495869

R$_{\alpha}$-Glu (3-*O*-benzoyl-4,6-*O*-ethylidene-2-*O*-methyl-ᴅ-glucopyranose)
E = -2033.769361
H = -2033.386082
qh-*G* = -2033.434300
N_{imag} = 0

C	-0.502270	0.525834	0.214106
C	0.856586	2.609763	0.352861
C	-0.559538	2.030170	0.400828
C	0.409743	-0.058415	1.294601
H	-0.141352	0.265444	-0.782181
O	-1.811818	-0.027091	0.401201
O	-1.331189	2.643145	-0.622014
H	1.308060	2.395151	-0.620919
C	0.769587	4.118377	0.542946
H	1.738386	4.600838	0.403012
H	0.387624	4.355594	1.546984
O	-0.103439	4.636549	-0.463701
C	1.768046	0.663712	1.323356
C	-1.398425	4.055186	-0.426218
H	-1.836163	4.239777	0.569055
H	-1.008293	2.254921	1.382012
C	-2.228481	4.652519	-1.535855
H	-1.761665	4.444531	-2.503501
H	-2.305784	5.734888	-1.400084
H	-3.232794	4.220039	-1.522042
C	-2.329411	-0.800667	-0.585834
H	2.394323	0.328289	2.147331
O	1.673809	2.038218	1.392142
O	2.461684	0.327979	0.050096
S	3.713895	-0.671652	-0.001772
O	4.030232	-1.179946	1.329077
O	4.726394	-0.064357	-0.852436
C	2.981875	-2.103649	-1.021322
F	3.963286	-2.585927	-1.790828
F	2.547701	-3.068664	-0.215861
F	1.989442	-1.665976	-1.792726
O	-1.753954	-1.022437	-1.636795
C	-3.670221	-1.332845	-0.230060
C	-6.194764	-2.383610	0.353130
C	-4.280456	-1.051551	1.002134
C	-4.329771	-2.141679	-1.167631
C	-5.588188	-2.665061	-0.875983
C	-5.540025	-1.577351	1.289947
H	-3.770469	-0.426275	1.725720
H	-3.846989	-2.351473	-2.116196
H	-6.095627	-3.290875	-1.604220
H	-6.010610	-1.358592	2.244048

H	-7.175633	-2.791779	0.580237
O	0.582810	-1.435297	1.059768
C	0.733096	-2.225277	2.242924
H	-0.140556	-2.107290	2.895752
H	1.644692	-1.958042	2.790730
H	0.805762	-3.261511	1.911130

TS_I-Glu (3-*O*-benzoyl-4,6-*O*-ethylidene-2-*O*-methyl-D-glucopyranose)
E = -2033.755912
H = -2033.373707
qh-*G* = -2033.421021
N_{imag} = 1

C	0.741013	0.097116	0.084048
C	-0.608157	2.162312	-0.526286
C	0.779376	1.558025	-0.358594
C	-0.224653	-0.701579	-0.812622
H	0.419843	0.012607	1.126831
O	2.138813	-0.272529	0.030224
O	1.501905	2.340070	0.584464
H	-1.151023	2.139964	0.423425
C	-0.458262	3.607281	-0.985898
H	-1.419518	4.123150	-1.014323
H	0.002352	3.643907	-1.984322
O	0.355927	4.291771	-0.031492
C	-1.517806	0.098438	-1.137365
C	1.629942	3.686953	0.134970
H	2.140146	3.672456	-0.842697
H	1.293181	1.594068	-1.332687
C	2.403285	4.465088	1.171363
H	1.866073	4.452011	2.124691
H	2.525662	5.500990	0.842955
H	3.391366	4.017545	1.310548
C	2.685341	-1.499407	-0.099672
H	-2.088524	-0.387606	-1.926013
O	-1.332431	1.413688	-1.515244
O	-2.319155	0.060036	0.105188
S	-3.923057	0.123532	0.034781
O	-4.392355	0.058624	-1.344262
O	-4.386988	1.141630	0.963660
C	-4.288464	-1.543308	0.829199
F	-5.612656	-1.656488	0.969732
F	-3.839377	-2.524239	0.044808
F	-3.704200	-1.618074	2.024523
O	2.075473	-2.521059	-0.350988
C	4.165861	-1.433575	0.074692
C	6.945827	-1.412148	0.384378
C	4.825374	-0.243326	0.419370
C	4.904548	-2.611362	-0.112100
C	6.290161	-2.599779	0.041761
C	6.212043	-0.236424	0.573118
H	4.251752	0.664397	0.566913
H	4.382993	-3.525294	-0.376332
H	6.858318	-3.513846	-0.104494
H	6.719908	0.685490	0.841481
H	8.025558	-1.403341	0.504766
O	-0.598189	-1.904769	-0.188906
C	-0.820751	-2.992431	-1.084642
H	0.090630	-3.210597	-1.651268
H	-1.651935	-2.787360	-1.771854
H	-1.078713	-3.851250	-0.462814

RC$_{I}$-Glu (3-*O*-benzoyl-4,6-*O*-ethylidene-2-*O*-methyl-D-glucopyranose)
E = -2033.766467
H = -2033.383083
qh-*G* = -2033.431519
N$_{imag}$ = 0

C	0.614595	0.226311	0.199752
C	-0.661397	2.284642	-0.336548
C	0.740065	1.678387	-0.236588
C	-0.267919	-0.524308	-0.813626
H	0.144260	0.181106	1.184563
H	0.234762	-0.544598	-1.786259
O	-0.586415	-1.829353	-0.384471
O	1.878618	-0.430126	0.430003
O	1.496305	2.427907	0.706651
H	-1.164420	2.227744	0.634566
C	-0.527997	3.744680	-0.750183
H	-1.488110	4.262852	-0.722872
H	-0.105250	3.816407	-1.763109
O	0.323760	4.393998	0.197124
O	-1.437024	1.575704	-1.319669
C	-1.588874	0.231481	-1.040348
H	-2.164527	-0.211149	-1.850472
C	0.189437	-2.875088	-0.974125
H	1.226027	-2.854681	-0.620071
H	0.172686	-2.804769	-2.069101
H	-0.278290	-3.810981	-0.663098
C	1.604964	3.788738	0.291943
H	2.073494	3.803784	-0.706036
H	1.221980	1.733957	-1.220207
C	2.419506	4.538342	1.317590
H	1.921498	4.498112	2.291265
H	2.528414	5.583201	1.013646
H	3.412449	4.088212	1.403630
C	2.803041	-0.526601	-0.551545
O	2.621143	-0.129608	-1.692322
C	4.047612	-1.186535	-0.081505
C	6.424509	-2.425297	0.710252
C	5.080473	-1.385296	-1.010308
C	4.208982	-1.610739	1.246809
C	5.396645	-2.228403	1.638296
C	6.265263	-2.003066	-0.614383
H	4.941923	-1.052740	-2.033588
H	3.410426	-1.454989	1.962796
H	5.520674	-2.555416	2.666451
H	7.063055	-2.155702	-1.335255
H	7.348225	-2.906828	1.018682
O	-2.351370	0.072524	0.221532
S	-3.951985	-0.053271	0.196868
O	-4.460343	0.611826	1.385163
O	-4.483012	0.209491	-1.134878
C	-4.118246	-1.905180	0.495701
F	-3.632833	-2.574527	-0.550690
F	-3.459786	-2.253124	1.600617

TS$_{II}$-Glu (3-*O*-benzoyl-4,6-*O*-ethylidene-2-*O*-methyl-D-glucopyranose)
E = -2033.755349
H = -2033.372620
qh-*G* = -2033.419836
N$_{imag}$ = 1

C	0.926459	0.502234	0.799062
C	-0.283474	2.342658	-0.347809

C	1.090593	1.792423	0.009269
C	-0.357647	-0.330732	0.488896
H	0.864915	0.783501	1.851486
H	-0.040918	-1.326578	0.164472
O	-1.146073	-0.459299	1.655884
O	2.122216	-0.311069	0.752037
O	1.804694	2.742522	0.797343
H	-0.910928	2.458834	0.543792
C	-0.118605	3.689277	-1.040648
H	-1.076577	4.189196	-1.192721
H	0.375952	3.550721	-2.013429
O	0.665352	4.533110	-0.194789
O	-0.871188	1.403670	-1.256715
C	-1.256694	0.220952	-0.673601
H	-1.341340	-0.510838	-1.473086
C	-0.632481	-1.413933	2.582592
H	0.341670	-1.108951	2.986756
H	-0.530900	-2.397928	2.105051
H	-1.357328	-1.471239	3.396092
C	1.938971	3.978982	0.100702
H	2.463002	3.782726	-0.849638
H	1.631617	1.606907	-0.926337
C	2.694556	4.945120	0.980268
H	2.141509	5.113898	1.909476
H	2.821575	5.898954	0.460659
H	3.680657	4.536884	1.218948
C	2.450449	-0.897778	-0.422536
O	1.752160	-0.807587	-1.421930
C	3.724214	-1.653118	-0.347381
C	6.113603	-3.101109	-0.298826
C	4.154807	-2.337844	-1.494040
C	4.494988	-1.695292	0.825131
C	5.687153	-2.418789	0.845424
C	5.346431	-3.059863	-1.468422
H	3.549271	-2.296087	-2.393196
H	4.161813	-1.163865	1.709114
H	6.283454	-2.450215	1.752514
H	5.677385	-3.589306	-2.356968
H	7.042711	-3.663761	-0.279494
O	-2.622555	0.443184	-0.122969
S	-3.893593	-0.299204	-0.739970
O	-5.051542	0.537760	-0.471857
O	-3.609915	-0.835776	-2.066526
C	-4.020899	-1.782907	0.411369
F	-2.915698	-2.528242	0.304028
F	-4.174986	-1.384231	1.672241

RC$_{II}$-Glu (3-*O*-benzoyl-4,6-*O*-ethylidene-2-*O*-methyl-D-glucopyranose)
E = -2033.757667
H = -2033.374516
qh-*G* = -2033.423108
N_{imag} = 0

C	1.153104	0.736996	1.012267
C	0.011304	2.554412	-0.258445
C	1.339062	1.822808	-0.042148
C	-0.324754	0.270113	1.137521
H	1.399598	1.167037	1.982707
H	-0.344059	-0.794216	1.392599
O	-0.899127	1.045922	2.180049
O	2.109929	-0.337550	0.890486
O	2.332956	2.744427	0.396031

H	-0.385229	2.929812	0.690285
C	0.239096	3.709977	-1.225949
H	-0.642726	4.347321	-1.311236
H	0.499574	3.318287	-2.220429
O	1.296704	4.524851	-0.716485
O	-0.906576	1.631861	-0.861267
C	-1.134488	0.455763	-0.165183
H	-0.993890	-0.380851	-0.842211
C	-1.589762	0.295889	3.181478
H	-0.911908	-0.418173	3.667331
H	-2.447405	-0.236148	2.757268
H	-1.940049	1.022126	3.917128
C	2.504696	3.800567	-0.546033
H	2.784314	3.353886	-1.514896
H	1.639721	1.380718	-1.001006
C	3.564270	4.739969	-0.023493
H	3.248670	5.160571	0.936354
H	3.723112	5.553855	-0.736463
H	4.505162	4.199666	0.113766
C	2.042781	-1.175564	-0.166670
O	1.163615	-1.102148	-1.012396
C	3.135948	-2.177220	-0.173079
C	5.161504	-4.099469	-0.270432
C	3.147819	-3.134750	-1.198956
C	4.142753	-2.185164	0.805187
C	5.152368	-3.145873	0.752947
C	4.158376	-4.093068	-1.246059
H	2.363770	-3.116586	-1.948514
H	4.132998	-1.443499	1.595557
H	5.931807	-3.150632	1.509142
H	4.165243	-4.833261	-2.040670
H	5.949507	-4.846366	-0.308017
O	-2.557246	0.452173	0.268186
S	-3.672574	-0.170200	-0.702895
O	-4.926539	0.500791	-0.403727
O	-3.177487	-0.335604	-2.063217
C	-3.787581	-1.898178	0.034827
F	-2.581590	-2.478500	-0.010118
F	-4.197369	-1.830546	1.303278

TS_{III}-Glu (3-*O*-benzoyl-4,6-*O*-ethylidene-2-*O*-methyl-D-glucopyranose)
E = -2033.736995
H = -2033.355352
qh-*G* = -2033.403708
N_{imag} = 1

C	1.386365	0.655271	1.085311
C	0.508286	2.442785	-0.473769
C	1.756748	1.725631	0.051069
C	-0.129456	0.303427	1.022937
H	1.567198	1.040068	2.088304
H	-0.300628	-0.709028	1.396604
O	-0.784972	1.277542	1.810946
O	2.241378	-0.497283	0.991139
O	2.617748	2.697172	0.619302
H	-0.073712	2.890292	0.330888
C	0.924032	3.482496	-1.503974
H	0.082413	4.094275	-1.830864
H	1.385624	2.998848	-2.376533
O	1.850311	4.353148	-0.853862
O	-0.330074	1.417910	-1.118825
C	-0.609856	0.386214	-0.417849

H	-1.089664	-0.419266	-0.955244
C	-1.693292	0.751591	2.789189
H	-1.160460	0.097478	3.490004
H	-2.510752	0.213781	2.305872
H	-2.086633	1.618859	3.321183
C	2.994293	3.677174	-0.354588
H	3.487052	3.153387	-1.189489
H	2.254366	1.246852	-0.803768
C	3.900583	4.683213	0.309407
H	3.371831	5.176858	1.130325
H	4.214044	5.435395	-0.419597
H	4.787455	4.179579	0.703614
C	2.078641	-1.308526	-0.073160
O	1.198680	-1.107276	-0.907949
C	3.035429	-2.428407	-0.119983
C	4.807279	-4.577306	-0.276616
C	2.906776	-3.368559	-1.155061
C	4.053961	-2.565824	0.837402
C	4.937041	-3.640830	0.754755
C	3.792410	-4.440770	-1.230578
H	2.114157	-3.249543	-1.885941
H	4.150549	-1.837041	1.633940
H	5.725665	-3.748936	1.493138
H	3.692776	-5.169168	-2.029483
H	5.497239	-5.414108	-0.336874
O	-2.870507	0.761543	-0.033460
S	-3.771980	-0.105270	-0.862651
O	-4.985856	0.568485	-1.353678
O	-3.034179	-0.948610	-1.831908
C	-4.412521	-1.341548	0.394684
F	-3.388473	-2.020187	0.951929
F	-5.084059	-0.719084	1.380508

PC-Glu (3-*O*-benzoyl-4,6-*O*-ethylidene-2-*O*-methyl-D-glucopyranose)

E = -2033.733685
H = -2033.351006
qh-*G* = -2033.400040
N_{imag} = 0

C	-1.721387	-0.558074	1.240206
C	-1.278552	-2.331256	-0.521212
C	-2.322232	-1.409332	0.110150
C	-0.178053	-0.593429	1.226127
H	-2.072085	-0.902189	2.212163
H	0.224593	0.290995	1.731854
O	0.215984	-1.781715	1.869710
O	-2.184059	0.807705	1.136836
O	-3.384233	-2.215875	0.586011
H	-0.864363	-3.020852	0.213231
C	-1.923647	-3.086159	-1.674187
H	-1.252110	-3.830310	-2.103931
H	-2.252212	-2.391939	-2.460830
O	-3.034582	-3.793077	-1.118998
O	-0.164923	-1.506604	-1.016638
C	0.271971	-0.590407	-0.224567
H	1.149254	-0.058171	-0.585834
C	1.573023	-1.781612	2.359074
H	1.722658	-0.936067	3.040809
H	2.288384	-1.735693	1.534375
H	1.684931	-2.719893	2.903227
C	-3.984529	-2.944264	-0.494338
H	-4.343619	-2.210737	-1.233835

H	-2.701607	-0.738168	-0.673514
C	-5.098740	-3.791740	0.066012
H	-4.697362	-4.500349	0.796678
H	-5.582965	-4.345699	-0.742738
H	-5.840971	-3.153541	0.553193
C	-1.690088	1.535726	0.132220
O	-0.810653	1.095384	-0.632282
C	-2.261383	2.878376	-0.001920
C	-3.310845	5.440690	-0.305255
C	-1.717484	3.746749	-0.964012
C	-3.332103	3.294044	0.808740
C	-3.852965	4.575926	0.652385
C	-2.244458	5.026475	-1.111747
H	-0.889508	3.412179	-1.579268
H	-3.748288	2.618223	1.546952
H	-4.680502	4.901429	1.274905
H	-1.825609	5.701141	-1.851722
H	-3.720138	6.439818	-0.422978
O	3.249563	-1.466309	-0.529270
S	3.833159	-0.184049	-1.000102
O	4.910467	-0.297670	-2.003704
O	2.814638	0.868261	-1.265849
C	4.715016	0.468375	0.522539
F	3.843693	0.647588	1.538417
F	5.663394	-0.392123	0.938341

P-Glu (3-*O*-benzoyl-4,6-*O*-ethylidene-2-*O*-methyl-D-glucopyranose)
E = -2033.748818
H = -2033.365176
qh-*G* = -2033.413614
N_{imag} = 0

C	-2.090166	-2.428630	-1.444255
C	-3.420652	-2.793677	-1.608325
C	-3.604173	-3.239597	0.776084
H	-1.822488	-2.899322	1.938309
H	-1.501572	-2.096470	-2.291131
H	-3.873703	-2.752768	-2.593380
H	-4.199063	-3.546973	1.629961
H	-5.218143	-3.473360	-0.633441
C	1.923004	-1.269883	-0.862570
C	2.501724	0.392760	0.963975
C	1.818503	0.183120	-0.385091
C	2.573156	-2.190843	0.185214
H	2.372527	-1.346515	-1.849978
H	2.365207	-3.238590	-0.070048
O	3.947081	-1.979070	0.355367
O	0.556317	-1.786180	-1.043883
O	2.401947	1.083491	-1.314914
H	3.580654	0.272173	0.861040
C	2.186907	1.815157	1.408463
H	2.705125	2.081602	2.331252
H	1.105731	1.948890	1.538027
O	2.692536	2.683724	0.385191
O	2.031000	-0.570246	1.939925
C	1.862030	-1.846829	1.491520
H	2.028432	-2.546067	2.306706
C	4.767199	-2.608104	-0.638831
H	4.608823	-2.163491	-1.627851
H	4.563488	-3.685255	-0.678377
H	5.798061	-2.438045	-0.327385
C	2.133481	2.431443	-0.896978

H	1.042988	2.547717	-0.842138
H	0.755429	0.421612	-0.273638
C	2.765030	3.372313	-1.892100
H	3.847819	3.215336	-1.923281
H	2.561139	4.406898	-1.602276
H	2.346078	3.194870	-2.886563
C	-0.133516	-2.088605	0.018579
O	0.374381	-2.090897	1.206259
C	-1.514539	-2.474951	-0.160184
C	-4.176268	-3.196833	-0.501536
C	-2.274155	-2.880476	0.953491
O	-3.420133	0.704869	-0.236882
S	-2.009752	1.140695	-0.162456
O	-1.191661	0.480833	0.889188
O	-1.307800	1.285155	-1.461273
C	-2.140019	2.908640	0.457260
F	-2.748296	2.955992	1.658345
F	-0.918440	3.468951	0.591343

P_{bare}-Glu (3-*O*-benzoyl-4,6-*O*-ethylidene-2-*O*-methyl-ᴅ-glucopyranose)
E = -1072.134569
H = -1071.784089
qh-*G* = -1071.822661
N_{imag} = 0

C	-0.597746	0.604053	-1.022398
C	-2.042520	0.167496	1.014717
C	-1.332569	-0.457465	-0.184914
C	-0.607984	1.991997	-0.351412
H	-0.943590	0.617166	-2.053484
H	0.166585	2.622230	-0.808709
O	-1.855374	2.624215	-0.375881
O	0.819057	0.221341	-1.110721
O	-2.289484	-1.153202	-0.960206
H	-2.839013	0.827336	0.670931
C	-2.643404	-0.961932	1.839883
H	-3.243463	-0.587854	2.670492
H	-1.859586	-1.628671	2.226918
O	-3.531302	-1.672067	0.970513
O	-1.112308	0.949114	1.804735
C	-0.232853	1.714566	1.101657
H	0.055097	2.582184	1.688818
C	-2.144881	3.313241	-1.601900
H	-2.204652	2.616954	-2.445686
H	-1.382468	4.075693	-1.800261
H	-3.114736	3.788142	-1.454248
C	-2.892356	-2.199221	-0.178428
H	-2.085013	-2.876647	0.143786
H	-0.585201	-1.175956	0.187550
C	-3.921820	-2.894295	-1.033207
H	-4.698010	-2.184203	-1.333907
H	-4.381379	-3.708824	-0.466899
H	-3.445805	-3.307791	-1.926512
C	1.553221	0.376848	-0.044345
O	1.119627	0.966275	1.021125
C	2.900635	-0.141915	-0.063138
C	5.497484	-1.134014	-0.097998
C	3.756756	0.104315	1.029958
C	3.348394	-0.887174	-1.173684
C	4.647349	-1.379635	-1.183760
C	5.053087	-0.394524	1.005416
H	3.402616	0.680812	1.876735

H	2.681400	-1.074880	-2.007108
H	4.999684	-1.955462	-2.033112
H	5.719289	-0.208387	1.841258

TS$_V$-Glu (3-*O*-benzoyl-4,6-*O*-ethylidene-2-*O*-methyl-D-glucopyranose)
E = -2033.754788
H = -2033.372464
qh-*G* = -2033.419893
N_{imag} = 1

C	-0.881002	0.020632	-0.094469
C	0.904652	1.676267	0.276398
C	-0.596191	1.429502	0.376333
C	0.103748	-1.042523	0.467373
H	-0.821130	0.000240	-1.184182
H	-0.463569	-1.663600	1.172878
O	0.618901	-1.853028	-0.563080
O	-2.217053	-0.340883	0.308820
O	-1.299953	2.348182	-0.449206
H	1.276215	1.415013	-0.717772
C	1.198680	3.141111	0.581597
H	2.239971	3.394336	0.375061
H	0.977627	3.353065	1.638407
O	0.392018	3.954457	-0.270645
O	1.550755	0.880550	1.279099
C	1.318096	-0.479851	1.290013
H	1.215277	-0.777491	2.332484
C	-0.288284	-2.828793	-1.076605
H	-1.109612	-2.359901	-1.630004
H	-0.693749	-3.441752	-0.260653
H	0.294774	-3.454959	-1.753413
C	-0.993564	3.697387	-0.107382
H	-1.255436	3.851972	0.952916
H	-0.908727	1.554661	1.425043
C	-1.769549	4.608967	-1.026390
H	-1.481670	4.422112	-2.065523
H	-1.559452	5.653051	-0.777829
H	-2.841690	4.425342	-0.913386
C	-3.153528	-0.551628	-0.650526
O	-2.908847	-0.512784	-1.844171
C	-4.488503	-0.848112	-0.072275
C	-7.037587	-1.418243	0.923611
C	-5.544261	-1.120828	-0.955286
C	-4.714273	-0.861120	1.313049
C	-5.987395	-1.145942	1.806493
C	-6.814609	-1.405350	-0.457698
H	-5.356339	-1.106566	-2.023585
H	-3.898587	-0.648735	1.994376
H	-6.160663	-1.155049	2.878590
H	-7.629469	-1.616325	-1.143900
H	-8.028034	-1.639653	1.311216
O	2.559399	-1.220933	0.972505
S	3.331565	-1.169870	-0.442021
O	3.595820	-2.524528	-0.902769
O	2.836969	-0.129348	-1.334050
C	4.961490	-0.534292	0.248428
F	4.791163	0.674794	0.788298
F	5.433992	-1.372904	1.174545

RC$_V$-Glu (3-*O*-benzoyl-4,6-*O*-ethylidene-2-*O*-methyl-D-glucopyranose)
E = -2033.759329
H = -2033.376334

qh-*G* = -2033.425086
N_{imag} = 0

C	1.186842	0.771731	0.719968
C	-0.072772	2.733021	-0.236127
C	1.259197	1.979045	-0.207955
C	-0.279362	0.374495	1.027609
H	1.682443	0.994433	1.665771
H	-0.322174	-0.693558	1.270021
O	-0.775813	1.150112	2.098173
O	1.872032	-0.313759	0.064069
O	2.299905	2.856347	0.205852
H	-0.362409	3.048777	0.769138
C	0.076491	3.947558	-1.143949
H	-0.802185	4.593720	-1.106670
H	0.244491	3.625266	-2.182242
O	1.183909	4.720100	-0.674130
O	-1.067581	1.857674	-0.793795
C	-1.163285	0.600788	-0.203766
H	-0.967651	-0.161939	-0.959412
C	-0.565124	0.558225	3.382956
H	0.500027	0.386278	3.575634
H	-1.105986	-0.394099	3.456629
H	-0.962103	1.265094	4.112968
C	2.396130	3.983517	-0.664015
H	2.587821	3.611039	-1.684381
H	1.461725	1.621589	-1.229713
C	3.504068	4.878550	-0.164837
H	3.274971	5.225848	0.847395
H	3.608238	5.743761	-0.825516
H	4.448654	4.327614	-0.149766
C	2.388255	-1.287846	0.855423
O	2.298790	-1.262027	2.072119
C	3.054895	-2.358393	0.074507
C	4.328781	-4.421859	-1.314432
C	3.641813	-3.421580	0.777467
C	3.107560	-2.331976	-1.328035
C	3.744238	-3.363494	-2.017796
C	4.277093	-4.449749	0.083670
H	3.593623	-3.429422	1.861226
H	2.654202	-1.510028	-1.869988
H	3.784411	-3.342123	-3.102819
H	4.731004	-5.271138	0.630127
H	4.823893	-5.223728	-1.854835
O	-2.546802	0.424789	0.260266
S	-3.640140	-0.226311	-0.726649
O	-4.919558	0.395049	-0.432641
O	-3.124626	-0.360229	-2.083241
C	-3.683028	-1.957983	0.009208
F	-2.456165	-2.489904	-0.052151
F	-4.081439	-1.906759	1.280063

TS$_{VI}$-Glu (3-*O*-benzoyl-4,6-*O*-ethylidene-2-*O*-methyl-D-glucopyranose)
E = -2033.748559
H = -2033.366205
qh-*G* = -2033.413844
N_{imag} = 1

C	1.178328	0.729983	0.995022
C	0.087234	2.434096	-0.555719
C	1.398609	1.899977	0.021010
C	-0.328376	0.386012	1.098846
H	1.507643	1.009161	1.997959

H	-0.474567	-0.580368	1.574061
O	-0.895906	1.426430	1.881559
O	2.088702	-0.308603	0.545820
O	2.112024	2.969283	0.630107
H	-0.539484	2.866723	0.225100
C	0.427407	3.503443	-1.582976
H	-0.462855	4.005447	-1.965782
H	0.990182	3.071120	-2.423473
O	1.209193	4.498662	-0.911843
O	-0.580965	1.322266	-1.179581
C	-0.976275	0.331885	-0.296746
H	-0.814737	-0.633254	-0.773462
C	-1.649062	0.968923	3.006808
H	-1.018102	0.380679	3.685365
H	-2.507602	0.368707	2.685504
H	-2.001279	1.864390	3.521889
C	2.399842	3.981129	-0.336866
H	3.001928	3.519881	-1.137648
H	1.987991	1.505184	-0.819851
C	3.131140	5.107440	0.351976
H	2.500539	5.535977	1.137056
H	3.379720	5.887190	-0.373358
H	4.055484	4.731582	0.799611
C	1.845959	-1.633909	0.495465
O	0.767291	-2.154557	0.732076
C	3.059618	-2.392481	0.087408
C	5.282663	-3.909757	-0.670424
C	2.946764	-3.783698	-0.056440
C	4.291858	-1.763229	-0.149101
C	5.398782	-2.523292	-0.526731
C	4.055319	-4.538963	-0.435016
H	1.989044	-4.257927	0.129514
H	4.378903	-0.688922	-0.036196
H	6.351452	-2.034497	-0.708474
H	3.963725	-5.615362	-0.546420
H	6.146702	-4.498698	-0.965101
O	-2.433793	0.470943	-0.039720
S	-3.483311	-0.310640	-0.969882
O	-4.686046	0.500995	-1.051978
O	-2.840279	-0.877836	-2.148522
C	-3.865720	-1.750874	0.179650
F	-2.729141	-2.392025	0.472843
F	-4.424558	-1.302165	1.305391

R_β-Man (3-*O*-benzoyl-4,6-*O*-ethylidene-2-*O*-methyl-D-mannopyranose)
E = -2033.765659
H = -2033.382585
qh-*G* = -2033.430809
N_{imag} = 0

C	-0.217673	-1.629182	0.806785
C	2.305488	-1.478648	0.747125
C	1.031019	-1.733255	-0.061226
C	0.055734	-0.690567	2.021667
H	-0.522787	-2.615909	1.158735
O	-1.251794	-1.070388	-0.009371
O	1.119513	-3.027045	-0.647674
H	2.385535	-2.212054	1.558042
C	3.505489	-1.622036	-0.184094
H	4.448966	-1.560098	0.361016
H	3.478845	-0.833581	-0.950507
O	3.454542	-2.914026	-0.789984

C	1.097371	0.372038	1.675286
C	2.250457	-3.125787	-1.511634
H	2.161386	-2.341525	-2.281600
H	0.960827	-0.976302	-0.854497
C	2.278394	-4.511867	-2.107443
H	1.359185	-4.691992	-2.671763
H	2.363300	-5.258386	-1.311728
H	3.133723	-4.608169	-2.782021
C	-2.534317	-1.413094	0.249757
O	2.320319	-0.152138	1.315926
S	0.762898	2.681000	0.304692
O	1.231706	3.317495	1.529732
O	-0.367187	3.191131	-0.455337
C	2.217221	2.640627	-0.893346
F	3.317371	2.215745	-0.272048
F	1.937556	1.825192	-1.914003
F	2.415014	3.879224	-1.353466
O	0.509261	1.116434	0.518701
H	1.241798	1.091870	2.478340
O	-2.848228	-2.339969	0.979098
C	-3.490669	-0.539971	-0.475356
C	-5.350161	1.079953	-1.787418
C	-4.833610	-0.936684	-0.555716
C	-3.081000	0.674922	-1.047052
C	-4.013546	1.482365	-1.698224
C	-5.759465	-0.130242	-1.215606
H	-5.136838	-1.874842	-0.102504
H	-2.047589	0.989751	-0.962979
H	-3.697712	2.426685	-2.131969
H	-6.797722	-0.441821	-1.283230
H	-6.073277	1.710044	-2.297870
O	-1.099110	-0.046895	2.491754
C	-1.754139	-0.717396	3.574976
H	-2.616619	-0.101200	3.833637
H	-1.083573	-0.794604	4.440309
H	-2.096258	-1.712083	3.270839

RC_{IV}-Man (3-*O*-benzoyl-4,6-*O*-ethylidene-2-*O*-methyl-D-mannopyranose)
E = -2995.365491
H = -2994.950781
qh-*G* = -2995.007008
N_{imag} = 0

C	-0.650770	-1.361794	-0.713918
C	0.008178	-0.995793	0.646050
C	0.343668	0.487825	0.728843
C	1.240776	0.124838	-1.458144
C	-0.140595	-0.431545	-1.806580
H	0.744401	0.768820	1.699192
H	-0.429329	-2.400534	-0.963791
H	1.950194	-0.693075	-1.298506
H	-0.844118	0.407770	-1.904174
O	1.185649	0.929670	-0.262507
O	2.884699	-0.649755	2.274602
O	-0.056917	-1.125053	-3.048769
C	1.715352	1.009252	-2.604665
H	2.746429	1.335877	-2.459918
H	1.064377	1.892242	-2.690467
O	1.682313	0.246225	-3.812506
C	0.387869	-0.263270	-4.092362
H	-0.314275	0.585270	-4.157983
S	3.755985	-1.526104	1.455433

O	3.075627	-2.165428	0.299251
O	4.664412	-2.411622	2.219123
C	4.936621	-0.321617	0.628996
O	-2.071562	-1.183642	-0.630133
F	5.628184	0.383837	1.546734
F	5.826311	-0.976759	-0.145114
F	4.276915	0.554630	-0.154254
C	0.446503	-1.056540	-5.375363
H	-0.544470	-1.453775	-5.612662
H	1.149651	-1.888114	-5.266438
H	0.776866	-0.412692	-6.195424
C	-2.822241	-2.219710	-0.198637
O	-2.415341	-3.368549	-0.128648
C	-4.201440	-1.787604	0.146593
C	-6.804271	-1.040742	0.835106
C	-5.198852	-2.763898	0.282723
C	-4.507550	-0.435367	0.364785
C	-5.807458	-0.067204	0.712597
C	-6.498826	-2.389609	0.619877
H	-4.945250	-3.806319	0.119216
H	-3.728475	0.313327	0.283168
H	-6.040552	0.978438	0.891768
H	-7.272097	-3.146228	0.717197
H	-7.816544	-0.750155	1.102608
O	-0.797187	-1.338545	1.748033
C	-0.432467	-2.574901	2.371152
H	-0.547880	-3.411766	1.673008
H	-1.119962	-2.706613	3.208865
H	0.598835	-2.529642	2.740002
H	0.975216	-1.507052	0.690387
O	-0.428097	2.788761	2.455610
S	-1.291976	2.573812	1.300390
O	-2.740361	2.702375	1.383312
O	-0.991849	1.187002	0.583954
C	-0.747489	3.803895	-0.021006
F	0.582266	3.854582	-0.094107
F	-1.245845	3.446742	-1.209769

TS$_{IV}$-Man (3-*O*-benzoyl-4,6-*O*-ethylidene-2-*O*-methyl-D-mannopyranose)
E = -2995.344817
H = -2994.931103
qh-*G* = -2994.988252
N_{imag} = 1

C	-0.773378	-0.213240	-1.878597
C	0.418710	-1.070555	-1.321004
C	1.209029	-0.356894	-0.252738
C	0.995185	1.560127	-1.572126
C	-0.504030	1.271050	-1.690317
H	1.367740	-0.794187	0.723484
H	-0.890049	-0.445487	-2.938524
H	1.561580	1.171678	-2.423056
H	-0.981853	1.587599	-0.755184
O	1.468096	0.896720	-0.358835
O	3.223694	-2.123616	1.441307
O	-1.020725	2.016289	-2.784268
C	1.211510	3.060085	-1.414189
H	2.269504	3.323821	-1.448603
H	0.781695	3.400723	-0.462253
O	0.578726	3.704554	-2.518773
C	-0.810619	3.415979	-2.596621
H	-1.284063	3.709520	-1.646417

S	3.922251	-1.850744	0.172813
O	3.022890	-1.230249	-0.876418
O	4.798092	-2.900435	-0.370890
C	5.083494	-0.433891	0.575883
O	-1.974729	-0.528962	-1.188744
F	5.963907	-0.805395	1.520521
F	5.769327	-0.055804	-0.517875
F	4.392715	0.625564	1.028689
C	-1.392732	4.149955	-3.778864
H	-2.462973	3.938875	-3.854984
H	-0.896411	3.828152	-4.699585
H	-1.251325	5.226865	-3.652193
C	-2.710847	-1.573946	-1.639366
O	-2.526217	-2.107134	-2.721649
C	-3.765165	-1.950913	-0.667500
C	-5.755721	-2.720326	1.130340
C	-4.813844	-2.777794	-1.096681
C	-3.705594	-1.516320	0.666222
C	-4.702525	-1.906835	1.560858
C	-5.810898	-3.156020	-0.199140
H	-4.839215	-3.111062	-2.129245
H	-2.873654	-0.902055	0.993428
H	-4.653088	-1.579464	2.595360
H	-6.627820	-3.789924	-0.531902
H	-6.531216	-3.019350	1.830371
O	0.011039	-2.311266	-0.823859
C	0.324193	-3.425677	-1.669072
H	-0.197604	-3.340321	-2.628786
H	-0.030676	-4.311370	-1.140335
H	1.406758	-3.495495	-1.824238
H	1.111811	-1.189080	-2.162900
O	0.628074	1.424752	2.898727
S	-0.706774	0.831350	2.662675
O	-1.358145	0.213755	3.839413
O	-0.830414	0.053316	1.402491
C	-1.792614	2.326076	2.320183
F	-1.332218	3.031212	1.260382
F	-3.057708	1.953239	2.041948

RC$_{III}$-Man (3-*O*-benzoyl-4,6-*O*-ethylidene-2-*O*-methyl-D-mannopyranose)
E = -2995.359446
H = -2994.943262
qh-*G* = -2995.001140
$\boldsymbol{N_{imag}}$ = 0

C	-0.819532	1.810660	-1.274751
C	0.416457	1.142577	-1.923390
C	1.274834	0.411552	-0.886158
C	1.069440	2.478277	0.269552
C	-0.443894	2.477739	0.049116
H	0.894154	-0.579684	-0.644957
H	-1.248676	2.540805	-1.962574
H	1.585974	3.044350	-0.513936
H	-0.893177	1.886313	0.859193
O	1.498416	1.111318	0.285703
O	2.319285	-2.267535	-1.685722
O	-0.930445	3.815356	0.107933
C	1.360073	3.126615	1.616567
H	2.429969	3.262735	1.783481
H	0.939982	2.518014	2.428755
O	0.769415	4.430899	1.605331
C	-0.631761	4.397664	1.376576

H	-1.097876	3.771336	2.155067
S	3.296087	-1.188401	-1.673928
O	2.598383	0.248495	-1.566557
O	4.331122	-1.066073	-2.690463
C	4.185237	-1.277579	-0.016139
O	-1.790782	0.794255	-0.990686
F	4.923673	-2.392416	0.007464
F	4.984170	-0.215578	0.127866
F	3.298066	-1.307125	0.976494
C	-1.157775	5.812228	1.390812
H	-2.237472	5.808306	1.216957
H	-0.670054	6.399828	0.606729
H	-0.956390	6.273400	2.361788
C	-2.769415	0.566665	-1.898895
O	-2.991036	1.307943	-2.843826
C	-3.540409	-0.660373	-1.582355
C	-5.032805	-2.958749	-1.044217
C	-4.730607	-0.904079	-2.285263
C	-3.093704	-1.574693	-0.616046
C	-3.843694	-2.720245	-0.348241
C	-5.475659	-2.050149	-2.013738
H	-5.060539	-0.191012	-3.033874
H	-2.163046	-1.402845	-0.089591
H	-3.488870	-3.416641	0.404993
H	-6.399005	-2.236553	-2.554935
H	-5.615220	-3.851887	-0.833822
O	0.076682	0.197814	-2.906947
C	-0.065396	0.739268	-4.221968
H	-0.888397	1.461196	-4.265040
H	-0.292180	-0.105142	-4.874928
H	0.870689	1.213486	-4.544759
H	1.028998	1.942169	-2.361785
O	0.650494	-2.667674	3.173714
S	-0.466481	-2.250389	2.293571
O	-1.668041	-3.117874	2.336319
O	-0.070119	-1.800393	0.938507
C	-1.067253	-0.666432	3.107294
F	-0.077259	0.247629	3.190795
F	-2.079616	-0.108886	2.410534

R_α-Man (3-*O*-benzoyl-4,6-*O*-ethylidene-2-*O*-methyl-D-mannopyranose)
E = -2033.769387
H = -2033.386180
qh-*G* = -2033.434577
N_{imag} = 0

C	-0.749751	0.060508	0.048558
C	1.221868	1.496435	0.487925
C	-0.309088	1.447437	0.464825
C	-0.153817	-0.960739	1.037095
H	-0.417329	-0.162114	-0.968220
O	-2.183586	0.008180	0.098496
O	-0.766742	2.430154	-0.455756
H	1.601187	1.279011	-0.516335
C	1.660636	2.897557	0.893061
H	2.742131	3.020718	0.814612
H	1.345328	3.111372	1.924836
O	1.067544	3.825491	-0.018017
C	1.364605	-0.759087	1.178714
C	-0.349836	3.732765	-0.052736
H	-0.736376	3.914672	0.963887
H	-0.699170	1.667265	1.468668

C	-0.874371	4.734595	-1.052087
H	-0.470300	4.516473	-2.045501
H	-0.575317	5.744383	-0.757419
H	-1.966133	4.684416	-1.090239
C	-2.808200	-0.847744	-0.745527
H	1.769224	-1.378332	1.976068
O	1.754854	0.540934	1.420551
O	1.917434	-1.236855	-0.118148
S	3.405814	-1.850351	-0.197180
O	3.409538	-2.808905	-1.289489
O	3.905922	-2.175814	1.132162
C	4.373906	-0.353606	-0.814264
F	5.605877	-0.758968	-1.128539
F	3.776610	0.150918	-1.896197
F	4.438282	0.579518	0.135134
O	-2.205955	-1.569852	-1.523170
C	-4.284052	-0.806113	-0.595348
C	-7.069456	-0.802184	-0.368093
C	-4.911324	0.043570	0.329400
C	-5.056605	-1.653074	-1.404255
C	-6.445584	-1.649782	-1.290365
C	-6.301693	0.042810	0.439994
H	-4.312586	0.697278	0.953019
H	-4.558610	-2.305208	-2.113910
H	-7.041283	-2.305895	-1.918089
H	-6.786017	0.700836	1.155445
H	-8.152214	-0.800348	-0.279494
O	-0.680745	-0.774308	2.339083
C	-1.679796	-1.721404	2.725924
H	-2.585911	-1.612527	2.120510
H	-1.297792	-2.746718	2.640028
H	-1.913011	-1.507276	3.770545

TS_I-Man (3-*O*-benzoyl-4,6-*O*-ethylidene-2-*O*-methyl-D-mannopyranose)
E = -2033.753691
H = -2033.371612
qh-*G* = -2033.419205
N_{imag} = 1

C	-0.769682	0.001124	-0.197475
C	1.170886	1.312680	0.661724
C	-0.358998	1.315740	0.460787
C	-0.188495	-1.165432	0.631399
H	-0.373579	-0.026440	-1.217716
O	-2.163083	-0.370763	-0.264918
O	-0.596237	2.449364	-0.364758
H	1.638632	1.289128	-0.328433
C	1.572380	2.604653	1.366993
H	2.657767	2.683736	1.452407
H	1.128407	2.636983	2.373260
O	1.132698	3.712915	0.584887
C	1.311059	-1.016166	0.909743
C	-0.262298	3.655686	0.303930
H	-0.811115	3.684852	1.259915
H	-0.872331	1.427160	1.425171
C	-0.619188	4.816967	-0.591731
H	-0.063832	4.746527	-1.532252
H	-0.367230	5.759342	-0.097070
H	-1.691066	4.802225	-0.806570
C	-3.231701	0.427287	-0.470057
H	1.671153	-1.783194	1.592310
O	1.653265	0.207039	1.436388

O	1.965697	-1.230144	-0.411345
S	3.457091	-1.834843	-0.499238
O	3.547455	-2.556485	-1.757733
O	3.853016	-2.426700	0.772216
C	4.465062	-0.257168	-0.727524
F	5.724828	-0.605689	-0.996042
F	3.966468	0.447964	-1.745169
F	4.430331	0.477604	0.383696
O	-3.194532	1.633425	-0.610226
C	-4.490932	-0.375628	-0.491103
C	-6.910306	-1.781728	-0.542254
C	-4.498409	-1.769789	-0.330863
C	-5.701007	0.308913	-0.677467
C	-6.906005	-0.391919	-0.702684
C	-5.705913	-2.468179	-0.356899
H	-3.564230	-2.299680	-0.187697
H	-5.683109	1.386690	-0.799990
H	-7.839962	0.143460	-0.846972
H	-5.707270	-3.547250	-0.232705
H	-7.849238	-2.327975	-0.561832
O	-0.834578	-1.173268	1.890978
C	-1.135994	-2.475762	2.390356
H	-1.812120	-3.004239	1.706439
H	-0.226516	-3.072650	2.538359
H	-1.629181	-2.329174	3.352673

RC_I-Man (3-*O*-benzoyl-4,6-*O*-ethylidene-2-*O*-methyl-D-mannopyranose)
E = -2033.762750
H = -2033.380214
qh-*G* = -2033.427724
N_{imag} = 0

C	-0.733639	-0.054413	-0.265739
C	1.121471	1.414101	0.426409
C	-0.403769	1.306203	0.322950
C	-0.146458	-1.154601	0.632211
H	-0.262885	-0.125189	-1.250467
O	-2.111691	-0.352262	-0.546868
O	-0.860895	2.349272	-0.529144
H	1.568748	1.300395	-0.567343
C	1.471439	2.789702	0.980732
H	2.548218	2.967727	0.970214
H	1.098020	2.887779	2.010603
O	0.877175	3.774864	0.133136
O	1.642573	0.402990	1.307064
C	1.346093	-0.886392	0.923478
H	1.728380	-1.550430	1.696122
C	-0.532218	3.622651	0.017558
H	-0.974430	3.686059	1.025154
H	-0.844863	1.413560	1.317318
C	-1.058091	4.693541	-0.906535
H	-0.598818	4.592039	-1.894803
H	-0.822894	5.681957	-0.501924
H	-2.143204	4.598691	-1.003724
C	-3.155838	0.070683	0.206021
O	-3.077819	0.851794	1.135471
O	-0.847787	-1.164800	1.856263
C	-0.932374	-2.449073	2.475214
H	-1.514406	-2.311227	3.387657
H	0.059005	-2.841479	2.736238
H	-1.443361	-3.161033	1.814364
H	-0.236845	-2.121357	0.122228

C	-4.435043	-0.530225	-0.267952
C	-6.889090	-1.607345	-1.071379
C	-4.486210	-1.451431	-1.325460
C	-5.618455	-0.152085	0.383938
C	-6.840794	-0.688986	-0.016798
C	-5.711604	-1.986726	-1.723738
H	-3.572625	-1.743470	-1.829996
H	-5.565736	0.560588	1.200114
H	-7.754105	-0.392737	0.490929
H	-5.747730	-2.699367	-2.542625
H	-7.841737	-2.025986	-1.383776
O	2.034806	-1.222235	-0.353847
S	3.548742	-1.774956	-0.349096
O	3.923003	-2.250304	0.976597
O	3.700992	-2.589408	-1.543348
C	4.515955	-0.187776	-0.673936
F	4.005469	0.436320	-1.738001
F	4.456446	0.616772	0.386129

TS$_{II}$-Man (3-*O*-benzoyl-4,6-*O*-ethylidene-2-*O*-methyl-D-mannopyranose)
E = -2033.752130
H = -2033.369530
qh-*G* = -2033.416921
$\boldsymbol{N_{imag}}$ = 1

C	-1.001270	0.303601	-0.863892
C	0.974505	1.480738	0.079253
C	-0.544546	1.496127	-0.034886
C	-0.160244	-1.013074	-0.663236
H	-0.872511	0.597467	-1.907518
O	-2.410100	0.050177	-0.788238
O	-0.959982	2.694904	-0.688851
H	1.449195	1.422728	-0.907951
C	1.434708	2.743848	0.797418
H	2.520922	2.846067	0.786114
H	1.084388	2.727066	1.839937
O	0.900792	3.873188	0.105304
O	1.326923	0.334547	0.868608
C	1.096595	-0.868398	0.251462
H	1.092450	-1.635781	1.023167
C	-0.517892	3.843482	0.027539
H	-0.921784	3.787444	1.051957
H	-0.963109	1.457898	0.974601
C	-0.987243	5.076719	-0.705089
H	-0.565042	5.094041	-1.714676
H	-0.666325	5.973087	-0.167055
H	-2.078739	5.075227	-0.773128
C	-2.999064	-0.234323	0.399755
O	-2.426933	-0.181047	1.474062
O	-0.983585	-2.039055	-0.161732
C	-0.584323	-3.351168	-0.558645
H	-1.342084	-4.031589	-0.167229
H	0.393439	-3.623008	-0.140271
H	-0.543948	-3.428710	-1.653222
H	0.205638	-1.301970	-1.654883
C	-4.432694	-0.590340	0.231083
C	-7.134967	-1.277973	0.017835
C	-5.037841	-0.677008	-1.032170
C	-5.186385	-0.851041	1.385363
C	-6.533254	-1.193328	1.278328
C	-6.386214	-1.019949	-1.135028
H	-4.454325	-0.477738	-1.923448

H	-4.705759	-0.782533	2.355526
H	-7.113381	-1.393941	2.174330
H	-6.852404	-1.086713	-2.113735
H	-8.184782	-1.544963	-0.065585
O	2.239904	-1.174436	-0.675068
S	3.628253	-1.740567	-0.095189
O	3.441887	-2.342183	1.219397
O	4.275641	-2.446786	-1.189705
C	4.624349	-0.153239	0.149013
F	4.469882	0.643155	-0.912209
F	4.228844	0.485733	1.248176

RC$_{II}$-Man (3-*O*-benzoyl-4,6-*O*-ethylidene-2-*O*-methyl-ᴅ-mannopyranose)
E = -2033.760397
H = -2033.377104
qh-*G* = -2033.425436
N_{imag} = 0

C	0.854763	0.600364	0.838435
C	-0.738770	2.084088	-0.398858
C	0.702068	1.918091	0.083119
C	-0.275885	-0.419668	0.473405
H	0.768263	0.819886	1.903513
O	2.172234	0.032098	0.748263
O	1.037124	3.006111	0.941630
H	-1.444056	2.022192	0.435417
C	-0.877315	3.439678	-1.080820
H	-1.915653	3.661719	-1.332961
H	-0.268442	3.463083	-1.996579
O	-0.444010	4.445825	-0.163408
O	-1.015097	1.057173	-1.365681
C	-0.841785	-0.244275	-0.942552
H	-0.261540	-0.803084	-1.671282
C	0.895808	4.252751	0.263261
H	1.546823	4.233693	-0.626600
H	1.357683	1.924371	-0.795604
C	1.265128	5.362185	1.217528
H	0.596887	5.346352	2.084111
H	1.176233	6.329112	0.714474
H	2.296515	5.232381	1.557219
C	2.619179	-0.445937	-0.434656
O	2.000750	-0.323253	-1.480099
O	0.138005	-1.759893	0.563841
C	0.290722	-2.234384	1.902917
H	0.502279	-3.301910	1.826185
H	-0.633625	-2.082107	2.474669
H	1.124940	-1.733631	2.407827
H	-1.086243	-0.229168	1.186937
C	3.938631	-1.112564	-0.309775
C	6.416385	-2.397907	-0.167933
C	4.579871	-1.265534	0.929466
C	4.542456	-1.606620	-1.475938
C	5.778258	-2.246775	-1.404092
C	5.816268	-1.907534	0.996521
H	4.110731	-0.885438	1.829542
H	4.034714	-1.483829	-2.426708
H	6.243547	-2.627807	-2.308368
H	6.311008	-2.026097	1.956023
H	7.379374	-2.897703	-0.112295
O	-2.147621	-0.982682	-1.054530
S	-3.520546	-0.435441	-0.439381
O	-3.300760	0.541214	0.624903

O	-4.475797	-0.181168	-1.507412
C	-4.045325	-2.038901	0.394238
F	-4.136029	-3.020909	-0.502659
F	-3.154263	-2.369803	1.333238

TS$_{III}$-Man (3-*O*-benzoyl-4,6-*O*-ethylidene-2-*O*-methyl-D-mannopyranose)
E = -2033.749943
H = -2033.368240
qh-*G* = -2033.416356
N_{imag} = 1

C	0.871712	0.554973	0.841212
C	-0.628516	2.027908	-0.549966
C	0.739014	1.899077	0.122702
C	-0.210082	-0.473677	0.351207
H	0.711507	0.721432	1.906618
O	2.198758	0.015569	0.793761
O	0.882526	2.969772	1.046873
H	-1.449964	1.911130	0.159650
C	-0.720033	3.379926	-1.242549
H	-1.716841	3.564815	-1.645461
H	0.020119	3.447253	-2.052940
O	-0.476385	4.375909	-0.248453
O	-0.715827	0.966045	-1.546133
C	-0.594334	-0.252681	-1.096283
H	-0.293201	-0.979065	-1.837762
C	0.787869	4.231729	0.381222
H	1.571653	4.267246	-0.392771
H	1.510134	1.974473	-0.654016
C	0.944454	5.325586	1.408132
H	0.148544	5.252086	2.155472
H	0.888491	6.302233	0.919615
H	1.914108	5.232203	1.904900
C	2.673228	-0.442378	-0.387920
O	2.049093	-0.328327	-1.432874
O	0.189170	-1.809305	0.474204
C	0.233374	-2.283806	1.823648
H	0.426523	-3.355289	1.760908
H	-0.725795	-2.110135	2.325772
H	1.041079	-1.799993	2.384677
H	-1.096615	-0.269406	0.964020
C	4.008433	-1.068326	-0.264148
C	6.522653	-2.276597	-0.121907
C	4.658341	-1.190126	0.974296
C	4.621206	-1.554171	-1.429463
C	5.875595	-2.156301	-1.356864
C	5.913503	-1.793849	1.041153
H	4.182114	-0.815985	1.873163
H	4.106230	-1.455346	-2.379091
H	6.348466	-2.531837	-2.259383
H	6.415913	-1.888787	1.999174
H	7.500361	-2.746781	-0.066036
O	-2.314106	-1.040145	-1.188645
S	-3.570929	-0.475017	-0.515008
O	-3.260715	0.559952	0.486714
O	-4.658223	-0.224201	-1.464196
C	-4.121925	-1.963090	0.486062
F	-4.355143	-3.011618	-0.313158
F	-3.172414	-2.299015	1.374372

PC-Man (3-*O*-benzoyl-4,6-*O*-ethylidene-2-*O*-methyl-D-mannopyranose)
E = -2033.748374

H = -2033.366143
qh-*G* = -2033.415328
N_{imag} = 0

C	0.937259	0.511946	0.889362
C	-0.450434	1.966225	-0.652581
C	0.838723	1.858815	0.164340
C	-0.090414	-0.514055	0.302450
H	0.733194	0.657688	1.949387
O	2.266176	-0.024727	0.862187
O	0.849223	2.930327	1.092903
H	-1.344379	1.837888	-0.037726
C	-0.477114	3.305275	-1.373932
H	-1.421312	3.470532	-1.894108
H	0.355027	3.376816	-2.089036
O	-0.370012	4.308468	-0.364071
O	-0.449445	0.870204	-1.636060
C	-0.241200	-0.296995	-1.165560
H	-0.217657	-1.108332	-1.879849
C	0.810818	4.192094	0.414473
H	1.682376	4.240361	-0.257811
H	1.689966	1.957235	-0.522061
C	0.818507	5.288351	1.449955
H	-0.061706	5.199190	2.093684
H	0.803973	6.263186	0.955060
H	1.721562	5.214198	2.061935
C	2.726246	-0.509161	-0.307935
O	2.068995	-0.442148	-1.347031
O	0.261937	-1.849256	0.507284
C	0.064722	-2.304584	1.851396
H	0.265794	-3.376061	1.837483
H	-0.967971	-2.125305	2.171110
H	0.761819	-1.812309	2.539417
H	-1.065210	-0.270112	0.753994
C	4.074176	-1.097129	-0.214567
C	6.620020	-2.234249	-0.116757
C	4.782796	-1.122079	0.997862
C	4.643099	-1.642910	-1.376398
C	5.914128	-2.210010	-1.324912
C	6.054306	-1.690895	1.041816
H	4.339860	-0.700360	1.892725
H	4.082716	-1.618232	-2.304722
H	6.354275	-2.633189	-2.222672
H	6.603712	-1.711665	1.978054
H	7.611096	-2.677142	-0.077999
O	-2.543499	-1.151618	-1.292509
S	-3.625499	-0.446040	-0.544685
O	-3.109534	0.495472	0.483688
O	-4.742011	0.049328	-1.369945
C	-4.386930	-1.821651	0.476928
F	-4.880564	-2.789426	-0.314976
F	-3.465352	-2.374416	1.290539

P-Man (3-*O*-benzoyl-4,6-*O*-ethylidene-2-*O*-methyl-D-mannopyranose)
E = -2033.754300
H = -2033.370568
qh-*G* = -2033.418735
N_{imag} = 0

C	2.687824	-1.609425	-1.356660
C	3.270782	-2.859333	-1.527044
C	3.632882	-3.029953	0.871913
H	2.957472	-1.355840	2.047782

H	2.305408	-1.059406	-2.208343
H	3.349593	-3.286681	-2.521309
H	3.994170	-3.588848	1.729046
H	4.187664	-4.549112	-0.553377
C	0.880928	2.150498	-0.763503
C	-0.882895	2.498673	1.025932
C	-0.561516	1.863794	-0.325609
C	1.648131	2.917532	0.323358
H	0.919652	2.612430	-1.747397
O	3.036720	2.949210	0.127516
H	1.231771	3.927653	0.424640
O	1.572823	0.870069	-0.936392
O	-1.499473	2.349152	-1.272774
H	-0.891702	3.590392	0.941637
C	-2.275917	2.026899	1.425473
H	-2.622434	2.501774	2.344817
H	-2.288642	0.936055	1.544101
O	-3.166821	2.440773	0.382623
O	0.094205	2.119518	2.021187
C	1.396651	2.129539	1.601010
H	2.055032	2.388406	2.426050
C	3.466139	3.766008	-0.968360
H	3.174525	3.327602	-1.929587
H	3.052277	4.778083	-0.880984
H	4.553875	3.805898	-0.905661
C	-2.818103	1.922061	-0.894069
H	-2.807051	0.825118	-0.845355
H	-0.668599	0.778354	-0.229503
C	-3.798434	2.445644	-1.913092
H	-3.769093	3.539526	-1.936500
H	-4.809466	2.119768	-1.653361
H	-3.545352	2.056581	-2.903254
C	1.965582	0.227369	0.123973
O	1.844284	0.700842	1.319222
C	2.582538	-1.067111	-0.062168
C	3.740304	-3.569126	-0.416036
C	3.055357	-1.779705	1.055297
O	-0.353549	-3.371573	-0.382871
S	-0.983030	-2.041647	-0.242263
O	-0.433666	-1.187624	0.843820
O	-1.238474	-1.309811	-1.506932
C	-2.709377	-2.443406	0.377606
F	-2.661298	-3.098436	1.553735
F	-3.432973	-1.317669	0.561624

P_{bare}-Man (3-*O*-benzoyl-4,6-*O*-ethylidene-2-*O*-methyl-D-mannopyranose)
E = -1072.140176
H = -1071.789765
qh-*G* = -1071.828117
N_{imag} = 0

C	-0.674267	0.722579	-0.909411
C	-2.276711	0.351356	1.016667
C	-1.625064	-0.271114	-0.217645
C	-0.528277	2.017472	-0.088459
H	-0.955087	0.883310	-1.947712
O	0.661460	0.129162	-0.978844
O	-2.645454	-0.697082	-1.098573
H	-2.931128	1.177098	0.719616
C	-3.127850	-0.718262	1.688507
H	-3.698240	-0.319193	2.528160
H	-2.502266	-1.552630	2.036036

O	-4.074808	-1.160096	0.712098
O	-1.267909	0.854196	1.920253
C	-0.246090	1.541571	1.328343
H	0.138650	2.304273	1.999828
C	-3.474568	-1.683964	-0.459204
H	-2.825124	-2.528693	-0.179266
H	-1.032791	-1.142974	0.102434
C	-4.559767	-2.086249	-1.425509
H	-5.174343	-1.217480	-1.679794
H	-5.193027	-2.851998	-0.969507
H	-4.113716	-2.491913	-2.337634
C	1.384053	0.115007	0.104558
O	0.977178	0.616576	1.224235
O	0.559147	2.809260	-0.477657
C	0.385154	3.496359	-1.724884
H	1.244266	4.159957	-1.823082
H	-0.539794	4.084724	-1.712397
H	0.369630	2.794921	-2.566679
H	-1.469216	2.580284	-0.110651
C	2.680790	-0.519152	0.047465
C	5.181528	-1.729729	-0.059352
C	3.094060	-1.164033	-1.136349
C	3.523080	-0.481956	1.177061
C	4.771410	-1.089177	1.116581
C	4.345064	-1.766577	-1.182257
H	2.438286	-1.189159	-1.998965
H	3.196049	0.020554	2.079962
H	5.426665	-1.063675	1.980949
H	4.670839	-2.265906	-2.088745

TS$_V$-Man (3-*O*-benzoyl-4,6-*O*-ethylidene-2-*O*-methyl-D-mannopyranose)
E = -2033.757306
H = -2033.374848
qh-*G* = -2033.422390
N_{imag} = 1

C	-1.060476	0.459699	-0.612241
C	1.058964	1.589219	0.082477
C	-0.463700	1.658485	0.099976
C	-0.184085	-0.843718	-0.529374
H	-1.175841	0.707493	-1.668571
O	-2.357960	0.242316	-0.052009
O	-0.904797	2.852154	-0.537664
H	1.443704	1.465409	-0.937011
C	1.628992	2.860322	0.700060
H	2.711858	2.919750	0.581332
H	1.380462	2.895511	1.771194
O	1.069082	3.982503	0.017708
O	1.427268	0.467907	0.898379
C	1.078657	-0.754327	0.380318
H	1.006603	-1.457257	1.207952
C	-0.349007	4.006589	0.086697
H	-0.646460	4.000081	1.148518
H	-0.787833	1.656297	1.151803
C	-0.849420	5.230917	-0.639954
H	-0.532093	5.198619	-1.686828
H	-0.445553	6.132237	-0.170444
H	-1.941580	5.267254	-0.597605
C	-3.271282	-0.374154	-0.840728
O	-3.065690	-0.641173	-2.012865
O	-0.992073	-1.908821	-0.085746
C	-0.616009	-3.188459	-0.598168

H	-1.378957	-3.889051	-0.255329
H	0.363463	-3.506166	-0.219623
H	-0.594508	-3.171048	-1.695342
H	0.169409	-1.045726	-1.546495
C	-4.537374	-0.656503	-0.118637
C	-6.953223	-1.232631	1.164737
C	-4.683277	-0.396284	1.252694
C	-5.604845	-1.207882	-0.843017
C	-6.809297	-1.493716	-0.202486
C	-5.890023	-0.685021	1.890053
H	-3.855900	0.025120	1.811709
H	-5.477948	-1.405900	-1.902144
H	-7.634303	-1.919126	-0.766328
H	-6.000716	-0.484631	2.951735
H	-7.891867	-1.456485	1.663909
O	2.180503	-1.245002	-0.516816
S	3.530236	-1.851463	0.113570
O	3.333837	-2.233508	1.506688
O	4.077627	-2.768601	-0.873921
C	4.670795	-0.345957	0.095407
F	4.602366	0.256010	-1.094352
F	4.332303	0.515946	1.052331

RC_V-Man (3-*O*-benzoyl-4,6-*O*-ethylidene-2-*O*-methyl-D-mannopyranose)
E = -2033.760055
H = -2033.376988
qh-*G* = -2033.425376
N_{imag} = 0

C	0.960651	0.735781	0.687027
C	-0.832902	2.060381	-0.501680
C	0.619213	2.043839	-0.028943
C	-0.199854	-0.283683	0.563614
H	1.177451	0.921165	1.739719
O	2.126183	0.185099	0.050729
O	0.847351	3.166325	0.814757
H	-1.526832	1.982692	0.339360
C	-1.090888	3.369551	-1.234084
H	-2.142536	3.489126	-1.499722
H	-0.476785	3.427592	-2.144725
O	-0.762715	4.432528	-0.335077
O	-1.014044	0.954142	-1.404845
C	-0.752295	-0.297679	-0.867462
H	-0.099202	-0.861730	-1.533896
C	0.583212	4.383176	0.113954
H	1.247060	4.417492	-0.765818
H	1.261793	2.115294	-0.919325
C	0.820591	5.537869	1.056082
H	0.143214	5.465154	1.912553
H	0.641384	6.483400	0.536737
H	1.853987	5.519370	1.413370
C	3.047455	-0.434277	0.829129
O	3.002601	-0.431353	2.047729
O	0.196022	-1.601541	0.851652
C	0.107368	-1.954952	2.235764
H	0.390458	-3.006882	2.297843
H	-0.919996	-1.828218	2.599132
H	0.798127	-1.357281	2.839678
H	-0.991606	0.036819	1.249434
C	4.112892	-1.083322	0.024403
C	6.158453	-2.336703	-1.410348
C	4.073794	-1.106844	-1.378418

C	5.178600	-1.691599	0.704601
C	6.198371	-2.315598	-0.011757
C	5.096312	-1.732805	-2.091225
H	3.247988	-0.640016	-1.902609
H	5.195350	-1.668014	1.789085
H	7.022534	-2.784649	0.517579
H	5.064054	-1.751128	-3.176635
H	6.953275	-2.823633	-1.968398
O	-1.976668	-1.141270	-0.939307
S	-3.416213	-0.576690	-0.500632
O	-3.314321	0.386152	0.593144
O	-4.233783	-0.297241	-1.671777
C	-4.040921	-2.194710	0.233588
F	-4.005106	-3.162327	-0.682333
F	-3.291739	-2.539260	1.282266

TS_{VI}-Man (3-*O*-benzoyl-4,6-*O*-ethylidene-2-*O*-methyl-D-mannopyranose)
E = -2033.746023
H = -2033.363577
qh-*G* = -2033.411123
N_{imag} = 1

C	0.862851	0.562890	0.813460
C	-0.747804	2.146912	-0.285886
C	0.710985	1.913022	0.109424
C	-0.360008	-0.360577	0.493880
H	0.861863	0.752342	1.889622
O	2.067050	-0.200040	0.599413
O	1.150509	2.953610	0.977405
H	-1.430707	2.068810	0.565090
C	-0.864764	3.534552	-0.906734
H	-1.904638	3.808073	-1.093542
H	-0.304790	3.569094	-1.852737
O	-0.340348	4.484975	0.021598
O	-1.078815	1.171304	-1.291448
C	-0.886310	-0.151745	-0.930144
H	-0.261231	-0.661529	-1.665293
C	1.016631	4.228003	0.352403
H	1.607493	4.216695	-0.578077
H	1.299543	1.926871	-0.809759
C	1.488920	5.287612	1.317631
H	0.876920	5.265403	2.224710
H	1.406846	6.274821	0.854331
H	2.533786	5.106815	1.585043
C	3.069994	0.020684	-0.275751
O	3.191140	1.006549	-0.983633
O	-0.088776	-1.732089	0.611155
C	0.047555	-2.185856	1.957598
H	0.145255	-3.271359	1.906556
H	-0.840917	-1.924598	2.546775
H	0.940052	-1.761511	2.432421
H	-1.150043	-0.072752	1.197376
C	4.045918	-1.105457	-0.258338
C	5.933577	-3.168508	-0.301509
C	3.799837	-2.297591	0.439890
C	5.238900	-0.953375	-0.980708
C	6.180509	-1.981107	-0.999248
C	4.742604	-3.325360	0.414938
H	2.873703	-2.417330	0.988342
H	5.416201	-0.028228	-1.518761
H	7.104769	-1.857796	-1.556141
H	4.547678	-4.248610	0.952828

H	6.666839	-3.970003	-0.317500
O	-2.155634	-0.900039	-1.129005
S	-3.557705	-0.391318	-0.527933
O	-3.379653	0.552037	0.572037
O	-4.481559	-0.118508	-1.617827
C	-4.080432	-2.030446	0.236632
F	-4.094936	-2.990287	-0.687891
F	-3.234331	-2.360338	1.214902

R$_{\alpha}$-2dGlu (3-*O*-benzoyl-2-deoxy-4,6-*O*-ethylidene-D-glucopyranose)
E = -1919.264538
H = -1918.915438
qh-*G* = -1918.960840
$\boldsymbol{N_{imag}}$ = 0

C	-0.861015	-0.060736	0.182907
C	1.102615	1.423624	0.499872
C	-0.427317	1.354777	0.499572
C	-0.210113	-1.013526	1.193406
H	-0.593703	-0.322274	-0.843335
O	-2.293936	-0.120287	0.318705
O	-0.914259	2.272084	-0.471292
H	1.473713	1.135470	-0.489653
C	1.531021	2.855110	0.796023
H	2.609726	2.983030	0.691706
H	1.229990	3.138214	1.815571
O	0.913319	3.710786	-0.167368
C	1.287464	-0.776888	1.345238
C	-0.503014	3.603291	-0.168694
H	-0.873103	3.853397	0.839587
H	-0.803918	1.636448	1.495854
C	-1.056434	4.527255	-1.225847
H	-0.668048	4.243394	-2.208827
H	-0.763015	5.558089	-1.008549
H	-2.148092	4.464069	-1.239863
C	-2.949347	-1.067657	-0.391568
H	1.714809	-1.325941	2.181449
O	1.655606	0.547704	1.495475
O	1.891804	-1.332316	0.091765
S	3.406525	-1.870545	0.083121
O	3.481860	-2.908452	-0.932142
O	3.900646	-2.069274	1.440144
C	4.319998	-0.384467	-0.634860
F	5.573287	-0.758874	-0.901703
F	3.720302	0.009904	-1.760915
F	4.332049	0.622760	0.238417
O	-2.377185	-1.877307	-1.103451
C	-4.420757	-1.001504	-0.200061
C	-7.199643	-0.950324	0.099475
C	-5.022076	-0.021167	0.604574
C	-5.216450	-1.954597	-0.853407
C	-6.601861	-1.928427	-0.702977
C	-6.409008	0.001689	0.751433
H	-4.406245	0.716061	1.106619
H	-4.738818	-2.706226	-1.472977
H	-7.215017	-2.667861	-1.209679
H	-6.872971	0.761793	1.373149
H	-8.279569	-0.929843	0.216061
H	-0.658155	-0.841085	2.178345

TS$_{I}$-2dGlu (3-*O*-benzoyl-2-deoxy-4,6-*O*-ethylidene-D-glucopyranose)
E = -1919.253931

H = -1918.905634
qh-*G* = -1918.950219
N_{imag} = 1

C	-0.766813	-0.318699	-0.027159
C	1.028114	1.264930	0.664518
C	-0.485594	1.057342	0.556425
C	-0.100786	-1.359130	0.886443
H	-0.343384	-0.377412	-1.033934
O	-2.192145	-0.422494	-0.243185
O	-1.008516	2.090062	-0.268774
H	1.464389	1.206004	-0.338770
C	1.296899	2.648731	1.241970
H	2.361390	2.889041	1.232745
H	0.921358	2.709502	2.274057
O	0.644115	3.610135	0.410635
C	1.371028	-1.026720	1.115538
C	-0.751729	3.374380	0.294120
H	-1.195503	3.399705	1.303358
H	-0.939885	1.119412	1.558371
C	-1.344204	4.422411	-0.615990
H	-0.882623	4.359764	-1.606311
H	-1.166948	5.418264	-0.200314
H	-2.422035	4.264379	-0.712026
C	-3.039733	-1.305721	0.322027
H	1.826616	-1.682565	1.854186
O	1.629348	0.272493	1.509281
O	2.041077	-1.303882	-0.197733
S	3.596104	-1.705115	-0.255511
O	3.773168	-2.553117	-1.423459
O	4.092321	-2.079918	1.062813
C	4.381621	-0.049598	-0.701536
F	5.664912	-0.265933	-0.998455
F	3.757865	0.471395	-1.761469
F	4.297916	0.797926	0.324141
O	-2.714226	-2.218309	1.062425
C	-4.447550	-1.028801	-0.083550
C	-7.119814	-0.588119	-0.782528
C	-4.791588	0.068760	-0.888140
C	-5.447447	-1.902599	0.368990
C	-6.778970	-1.682767	0.019512
C	-6.125544	0.285748	-1.234338
H	-4.018607	0.744533	-1.235040
H	-5.168967	-2.747045	0.990511
H	-7.549642	-2.362658	0.370878
H	-6.389259	1.136322	-1.856199
H	-8.157622	-0.416639	-1.054488
H	-0.571668	-1.363414	1.871260

RC$_{I}$-2dGlu (3-*O*-benzoyl-2-deoxy-4,6-*O*-ethylidene-ᴅ-glucopyranose)
E = -1919.261343
H = -1918.912017
qh-*G* = -1918.957415
N_{imag} = 0

C	0.775311	-0.338674	0.151244
C	-0.995208	1.266360	-0.472607
C	0.519980	1.068416	-0.361572
C	0.133304	-1.338803	-0.818932
H	0.331406	-0.435440	1.144738
O	2.164576	-0.638125	0.408944
O	1.037325	2.043514	0.536202
H	-1.454456	1.123021	0.511804

C	-1.268147	2.687301	-0.949600
H	-2.333789	2.922079	-0.932142
H	-0.883731	2.824452	-1.970923
O	-0.627193	3.589148	-0.044839
O	-1.562291	0.334755	-1.409395
C	-1.323551	-0.992051	-1.104512
H	-1.743638	-1.588344	-1.911453
C	0.771220	3.360650	0.060352
H	1.217566	3.457847	-0.943074
H	0.973395	1.197952	-1.351533
C	1.349745	4.351162	1.041014
H	0.882592	4.220799	2.022115
H	1.167510	5.370766	0.690046
H	2.428150	4.194868	1.133596
C	3.095343	-0.534412	-0.564934
O	2.838129	-0.233567	-1.720439
H	0.658363	-1.309376	-1.776705
C	-4.441397	-0.141509	0.565036
F	-3.878756	0.345068	1.674474
F	-4.318590	0.745578	-0.421997
F	-5.735073	-0.384249	0.788494
H	0.194703	-2.355772	-0.424089
C	4.462580	-0.833123	-0.063042
C	7.069951	-1.375947	0.788112
C	4.713453	-1.130141	1.285645
C	5.522614	-0.808706	-0.981970
C	6.821930	-1.080133	-0.556876
C	6.015577	-1.400062	1.706862
H	3.894313	-1.146238	1.995167
H	5.315136	-0.576952	-2.021261
H	7.639891	-1.061135	-1.271101
H	6.207854	-1.628183	2.751149
H	8.082883	-1.586735	1.119540
O	-2.060363	-1.363043	0.150113
S	-3.613197	-1.770345	0.096940
O	-4.026810	-2.090130	-1.263810

TS_{II}-2dGlu (3-*O*-benzoyl-2-deoxy-4,6-*O*-ethylidene-ᴅ-glucopyranose)
E = -1919.252252
H = -1918.903684
qh-*G* = -1918.948340
N_{imag} = 1

C	-1.047615	0.162250	-1.023107
C	0.834149	1.314795	0.117796
C	-0.678464	1.287769	-0.068159
C	-0.146601	-1.097461	-0.919569
H	-0.969444	0.568462	-2.032424
O	-2.454976	-0.174026	-0.941921
O	-1.117013	2.531200	-0.611922
H	1.349122	1.374908	-0.848799
C	1.212750	2.514924	0.977318
H	2.293469	2.656975	1.024459
H	0.821467	2.381120	1.996691
O	0.661668	3.688682	0.377974
O	1.200987	0.110535	0.804375
C	1.032646	-1.038956	0.072361
H	1.037021	-1.867528	0.775450
C	-0.749832	3.615704	0.235302
H	-1.191941	3.440444	1.230232
H	-1.136722	1.130096	0.915041
C	-1.239900	4.899695	-0.388559

H	-0.777735	5.036921	-1.371100
H	-0.978810	5.747054	0.251548
H	-2.326704	4.864708	-0.505436
C	-2.934871	-0.735396	0.191034
O	-2.230721	-1.004172	1.153142
H	-0.759188	-1.952410	-0.635247
C	4.589956	-0.252200	0.143598
F	4.571783	0.538496	-0.932924
F	4.082337	0.403798	1.186144
F	5.849825	-0.607818	0.414102
H	0.256948	-1.317120	-1.909560
C	-4.396716	-0.987807	0.123444
C	-7.144388	-1.500670	0.088100
C	-5.160465	-0.643609	-1.002822
C	-5.014666	-1.588965	1.230533
C	-6.384624	-1.844359	1.211813
C	-6.531356	-0.900731	-1.016803
H	-4.682143	-0.177821	-1.856661
H	-4.412476	-1.849249	2.094566
H	-6.859971	-2.309988	2.070133
H	-7.121237	-0.633225	-1.888571
H	-8.212305	-1.699671	0.073847
O	2.247274	-1.238301	-0.812652
S	3.608229	-1.825671	-0.203520
O	3.378275	-2.480528	1.079165

RC_{II}-2dGlu (3-*O*-benzoyl-2-deoxy-4,6-*O*-ethylidene-D-glucopyranose)
E = -1919.253508
H = -1918.904339
qh-*G* = -1918.949927
N_{imag} = 0

C	-0.821057	0.371819	-0.899584
C	0.656384	1.900166	0.413069
C	-0.773894	1.650713	-0.067446
C	0.343283	-0.585488	-0.560252
H	-0.726315	0.656623	-1.948087
O	-2.128810	-0.251212	-0.879922
O	-1.209160	2.754471	-0.856736
H	1.354351	1.926677	-0.429205
C	0.704158	3.228633	1.158582
H	1.727216	3.516938	1.406317
H	0.112993	3.161542	2.083743
O	0.178902	4.243662	0.300845
O	1.013586	0.853812	1.329454
C	0.904172	-0.439802	0.852920
H	0.380748	-1.045491	1.586174
C	-1.150346	3.970040	-0.114302
H	-1.780464	3.854056	0.783428
H	-1.416018	1.552869	0.817158
C	-1.622328	5.096416	-1.001180
H	-0.972201	5.177814	-1.877812
H	-1.597548	6.039585	-0.448210
H	-2.647289	4.905290	-1.331269
C	-2.586371	-0.810091	0.259591
O	-1.935668	-0.840617	1.294138
H	0.039193	-1.623220	-0.712172
C	4.170713	-1.976000	-0.637056
F	4.342506	-3.012472	0.184856
F	3.264517	-2.289678	-1.568262
F	5.330794	-1.679289	-1.230156
H	1.142442	-0.378537	-1.274413

C	-3.950723	-1.375221	0.109735
C	-6.518186	-2.463674	-0.078475
C	-4.661246	-1.292353	-1.098058
C	-4.531139	-2.004794	1.221412
C	-5.811238	-2.547527	1.126204
C	-5.942445	-1.836206	-1.188154
H	-4.212015	-0.804567	-1.955425
H	-3.971282	-2.061417	2.148926
H	-6.257857	-3.034512	1.988062
H	-6.491583	-1.770543	-2.122806
H	-7.516291	-2.886289	-0.152048
O	2.259182	-1.111153	0.926268
S	3.593649	-0.465634	0.325074
O	3.315995	0.576734	-0.659619

TS$_{III}$-2dGlu (3-*O*-benzoyl-2-deoxy-4,6-*O*-ethylidene-D-glucopyranose)
E = -1919.244998
H = -1918.897264
qh-*G* = -1918.942617
N_{imag} = 1

C	-0.823965	0.349791	-0.890412
C	0.579250	1.842253	0.563724
C	-0.785940	1.660207	-0.100444
C	0.289374	-0.628359	-0.438738
H	-0.661048	0.580564	-1.943137
O	-2.142642	-0.238787	-0.901003
O	-1.024212	2.765356	-0.961839
H	1.395231	1.805168	-0.160498
C	0.599548	3.165550	1.315686
H	1.591011	3.396569	1.707987
H	-0.124638	3.147582	2.143047
O	0.269647	4.190500	0.377152
O	0.737138	0.748585	1.511352
C	0.679450	-0.462679	1.007669
H	0.403494	-1.207106	1.740328
C	-0.997319	3.993840	-0.232526
H	-1.762399	3.937012	0.559221
H	-1.547348	1.637759	0.689963
C	-1.251727	5.126569	-1.196045
H	-0.471501	5.145801	-1.963091
H	-1.248819	6.078331	-0.657688
H	-2.224964	4.993521	-1.676610
C	-2.632654	-0.764203	0.242949
O	-1.994024	-0.776184	1.286760
H	0.003980	-1.664200	-0.626682
C	4.145001	-1.948691	-0.736165
F	4.417610	-3.057059	-0.035872
F	3.153647	-2.216845	-1.601579
F	5.235928	-1.592065	-1.428059
H	1.160825	-0.411183	-1.058999
C	-3.999694	-1.312444	0.085967
C	-6.577559	-2.369165	-0.118124
C	-4.689053	-1.247922	-1.135368
C	-4.605755	-1.907450	1.203317
C	-5.891502	-2.434391	1.099785
C	-5.975884	-1.776224	-1.233103
H	-4.219478	-0.787176	-1.996798
H	-4.061546	-1.950340	2.140786
H	-6.358750	-2.894859	1.965170
H	-6.509215	-1.725642	-2.177685
H	-7.580096	-2.779845	-0.197968

O	2.398529	-1.170211	1.075714
S	3.637798	-0.549716	0.407447
O	3.300618	0.568037	-0.488140

PC-2dGlu (3-*O*-benzoyl-2-deoxy-4,6-*O*-ethylidene-D-glucopyranose)
E = -1919.244410
H = -1918.896098
qh-*G* = -1918.942419
N_{imag} = 0

C	-0.888997	0.341040	-0.936262
C	0.425590	1.781605	0.666190
C	-0.864747	1.655225	-0.144931
C	0.168638	-0.652820	-0.400468
H	-0.683618	0.547946	-1.985682
O	-2.210805	-0.235546	-0.960084
O	-0.943033	2.770448	-1.016822
H	1.317752	1.717270	0.039263
C	0.404737	3.085724	1.449190
H	1.349967	3.270853	1.960839
H	-0.415505	3.085794	2.181117
O	0.232444	4.130329	0.491241
O	0.475092	0.641838	1.596175
C	0.336368	-0.512963	1.065809
H	0.332112	-1.337049	1.764616
C	-0.954904	3.995369	-0.274933
H	-1.816554	3.966554	0.411558
H	-1.711584	1.675080	0.553952
C	-1.033766	5.141916	-1.251405
H	-0.161488	5.129552	-1.911790
H	-1.058250	6.089686	-0.706801
H	-1.942427	5.054602	-1.853337
C	-2.696500	-0.765084	0.180294
O	-2.043902	-0.784198	1.222963
H	-0.068725	-1.683171	-0.665694
C	4.344231	-1.865627	-0.741222
F	4.834907	-2.925618	-0.075267
F	3.356936	-2.298420	-1.550645
F	5.324374	-1.358938	-1.509572
H	1.129959	-0.393958	-0.861561
C	-4.062437	-1.306426	0.042907
C	-6.643668	-2.352300	-0.135815
C	-4.769563	-1.221564	-1.167627
C	-4.651393	-1.915613	1.162466
C	-5.939683	-2.437188	1.070708
C	-6.058572	-1.745090	-1.252165
H	-4.312285	-0.749786	-2.029694
H	-4.093207	-1.974209	2.090675
H	-6.395039	-2.909130	1.936042
H	-6.606660	-1.680064	-2.187178
H	-7.648330	-2.759309	-0.205914
O	2.618494	-1.309184	1.175068
S	3.694603	-0.569685	0.447405
O	3.172028	0.486815	-0.455985

P-2dGlu (3-*O*-benzoyl-2-deoxy-4,6-*O*-ethylidene-D-glucopyranose)
E = -1919.248623
H = -1918.898866
qh-*G* = -1918.943890
N_{imag} = 0

C	3.072103	-0.764947	1.463245
C	4.315072	-0.157834	1.597477

C	4.714609	-0.422614	-0.786411
H	3.134372	-1.364877	-1.907292
H	2.423049	-0.886402	2.322367
H	4.641974	0.192016	2.571241
H	5.352243	-0.279341	-1.652750
H	6.100688	0.497500	0.584399
C	-0.733911	-2.528693	0.963084
C	-2.271820	-1.725782	-0.874096
C	-1.618302	-1.377526	0.461617
C	-0.657147	-3.662757	-0.050246
H	-1.006347	-2.847425	1.966743
H	0.108800	-4.391885	0.224040
H	-1.612922	-4.186813	-0.123335
O	0.635936	-2.002589	1.113788
O	-2.648725	-1.068382	1.387780
H	-2.992477	-2.539306	-0.737697
C	-3.015789	-0.485718	-1.354488
H	-3.588871	-0.676607	-2.263456
H	-2.311595	0.337810	-1.527260
O	-3.960861	-0.147096	-0.331990
O	-1.284356	-2.140752	-1.843146
C	-0.322477	-2.995999	-1.368218
H	0.005785	-3.651818	-2.169880
C	-0.429402	3.235670	-0.555399
F	0.003628	3.615722	-1.773237
F	-1.715728	2.842275	-0.679508
F	-0.405485	4.314737	0.250681
C	-3.360033	0.093324	0.933921
H	-2.631457	0.909366	0.836058
H	-0.984050	-0.496689	0.317463
C	-4.446467	0.406782	1.931656
H	-5.143010	-0.434221	2.006094
H	-4.993820	1.298297	1.613019
H	-4.003461	0.596153	2.913352
C	1.351503	-1.822059	0.042820
O	0.964535	-2.193631	-1.131379
C	2.652365	-1.205951	0.194455
C	5.133908	0.014834	0.475992
C	3.475443	-1.033360	-0.933627
O	1.982529	2.458897	0.196841
S	0.635289	1.853474	0.138699
O	0.465263	0.771494	-0.866672

P$_{bare}$-2dGlu (3-*O*-benzoyl-2-deoxy-4,6-*O*-ethylidene-D-glucopyranose)
E = -957.634986
H = -957.318520
qh-*G* = -957.353686
N$_{imag}$ = 0

C	-0.672146	-0.197654	1.507244
C	-2.221581	-1.066637	-0.287081
C	-1.558258	0.177105	0.302973
C	-0.577692	-1.708641	1.684253
H	-0.966655	0.337250	2.407168
O	0.694414	0.279779	1.232963
O	-2.567828	1.094922	0.675136
H	-2.912109	-1.498953	0.443789
C	-3.019717	-0.641063	-1.512408
H	-3.598759	-1.465406	-1.930743
H	-2.355856	-0.231444	-2.286672
O	-3.955633	0.345951	-1.070213
O	-1.227626	-2.054979	-0.633343

C	-0.252209	-2.248767	0.308902
H	0.110117	-3.271389	0.257501
C	-3.341942	1.475867	-0.475023
H	-2.650416	1.929575	-1.203294
H	-0.921802	0.633865	-0.470940
C	-4.416070	2.434281	-0.026463
H	-5.075212	1.944762	0.696748
H	-5.006261	2.755438	-0.888995
H	-3.960213	3.312782	0.438210
C	1.416077	-0.389574	0.380231
O	1.015594	-1.492110	-0.160384
H	0.196885	-1.976182	2.406631
H	3.231707	-1.563419	-1.209516
H	5.462745	-0.660054	-1.814996
H	4.702461	2.843898	0.576982
H	6.192838	1.538587	-0.920386
H	-1.523839	-2.124023	2.038762
C	2.714358	0.138830	0.024382
C	5.215945	1.145502	-0.655235
C	3.126220	1.387354	0.533273
C	3.558164	-0.603661	-0.825999
C	4.806670	-0.094372	-1.161762
C	4.377821	1.883726	0.189766

R$_{\alpha}$-GluN (3-*O*-benzoyl-2,4,6-tri-*O*-methyl-D-glucopyranose)
E = -2034.945589
H = -2034.540474
qh-*G* = -2034.591934
N_{imag} = 0

C	-0.713686	0.254497	-0.085387
C	0.792982	2.251824	-0.352867
C	-0.662088	1.768655	-0.273241
C	0.128424	-0.174268	1.110744
H	-0.382513	-0.257325	-0.990496
O	-2.079837	-0.103149	0.181502
O	-1.320444	2.115948	-1.481818
H	1.259259	1.848143	-1.259172
C	0.918660	3.760851	-0.395786
H	0.511352	4.116321	-1.353397
H	1.983239	4.034146	-0.350860
O	0.210169	4.335470	0.688718
C	1.540292	0.425679	0.996250
C	-2.477910	2.935029	-1.313551
H	-3.244428	2.415324	-0.725413
H	-1.147006	2.252334	0.581537
C	0.275999	5.754662	0.686659
H	-0.297414	6.102260	1.548633
H	-0.161413	6.167956	-0.234161
H	1.315536	6.102115	0.775502
C	-2.695282	-0.983134	-0.643228
H	2.156118	0.225334	1.870934
O	1.543452	1.787534	0.797946
O	2.154752	-0.261974	-0.166426
S	3.749265	-0.432861	-0.254175
O	4.387761	-0.059507	1.002854
O	4.209890	0.079657	-1.535030
C	3.793312	-2.311121	-0.368346
F	5.066633	-2.690502	-0.513321
F	3.295927	-2.841930	0.750228
F	3.081929	-2.722394	-1.417129
O	-2.148617	-1.514419	-1.594358

C	-4.110844	-1.212746	-0.250427
C	-6.783885	-1.699955	0.410915
C	-4.698564	-0.546336	0.836000
C	-4.867714	-2.122783	-1.003341
C	-6.199862	-2.365534	-0.672514
C	-6.032309	-0.791370	1.163178
H	-4.113244	0.157090	1.416713
H	-4.401917	-2.630761	-1.841166
H	-6.782367	-3.071418	-1.257275
H	-6.485131	-0.274022	2.003984
H	-7.822376	-1.888955	0.668104
O	0.140276	-1.579220	1.181151
C	0.421231	-2.106251	2.477914
H	-0.293863	-1.720102	3.216295
H	1.442615	-1.869624	2.801808
H	0.315777	-3.189301	2.397926
H	-0.309262	0.253984	2.025023
H	-2.862750	3.133904	-2.315839

TS$_{I}$-GluN (3-*O*-benzoyl-2,4,6-tri-*O*-methyl-D-glucopyranose)
E = -2034.932299
H = -2034.528253
qh-*G* = -2034.578567
N_{imag} = 1

C	-0.737674	0.082638	-0.208614
C	0.664584	2.225254	-0.188870
C	-0.741246	1.621299	-0.238255
C	0.192689	-0.469478	0.881331
H	-0.424582	-0.313148	-1.179832
O	-2.152930	-0.202723	-0.063512
O	-1.382118	2.047333	-1.432579
H	1.212693	1.960959	-1.100025
C	0.673672	3.735537	-0.067896
H	0.298634	4.167028	-1.007599
H	1.711338	4.072457	0.072356
O	-0.132974	4.140472	1.023870
C	1.513388	0.330772	0.934856
C	-2.470547	2.949621	-1.237658
H	-3.251983	2.490479	-0.619747
H	-1.296669	1.968467	0.639493
C	-0.138356	5.550361	1.200500
H	0.874051	5.925482	1.408779
H	-0.786982	5.762644	2.052949
H	-0.530563	6.058944	0.307276
C	-2.735789	-1.369305	0.284488
H	2.113161	0.047135	1.797561
O	1.378111	1.695839	0.952925
O	2.255274	-0.069603	-0.292306
S	3.854750	-0.148911	-0.288671
O	4.396379	0.133086	1.035804
O	4.350454	0.515850	-1.483999
C	4.046636	-1.998035	-0.584405
F	5.345679	-2.258138	-0.765668
F	3.603177	-2.676026	0.475048
F	3.363977	-2.360998	-1.670254
O	-2.166824	-2.319385	0.787037
C	-4.201743	-1.334636	0.007894
C	-6.955796	-1.373959	-0.483920
C	-4.790272	-0.332527	-0.778150
C	-4.998339	-2.358997	0.541001
C	-6.371171	-2.375718	0.298938

C	-6.163581	-0.355640	-1.023589
H	-4.171514	0.449574	-1.200641
H	-4.531166	-3.131816	1.142370
H	-6.984759	-3.168033	0.717837
H	-6.614585	0.418800	-1.637343
H	-8.025258	-1.388799	-0.674717
O	0.516545	-1.819387	0.644155
C	0.653813	-2.607801	1.823856
H	-0.290071	-2.630377	2.379108
H	1.460153	-2.232991	2.468503
H	0.905061	-3.616203	1.490646
H	-0.285525	-0.337767	1.861273
H	-2.870916	3.167865	-2.229885

RC$_{I}$-GluN (3-*O*-benzoyl-2,4,6-tri-*O*-methyl-D-glucopyranose)
E = -2034.942342
H = -2034.537078
qh-*G* = -2034.588640
N_{imag} = 0

C	0.618933	0.122318	0.434944
C	-0.714713	2.238603	0.391814
C	0.704998	1.650805	0.447092
C	-0.193364	-0.382067	-0.763491
H	0.130680	-0.210292	1.353811
H	0.319254	-0.121309	-1.695044
O	-0.444202	-1.770445	-0.696644
O	1.915157	-0.511080	0.533007
O	1.322850	2.071007	1.656003
H	-1.255763	1.962245	1.304725
C	-0.732640	3.749297	0.279757
H	-0.355107	4.174291	1.221470
H	-1.771127	4.085482	0.143311
O	0.072655	4.162428	-0.809850
O	-1.438892	1.713955	-0.749472
C	-1.540236	0.346085	-0.808429
H	-2.106570	0.091163	-1.701979
C	0.378479	-2.581204	-1.537305
H	1.417388	-2.597446	-1.189043
H	0.344925	-2.228951	-2.576220
H	-0.033751	-3.590561	-1.483049
C	2.470876	2.901355	1.477089
H	3.271006	2.361533	0.954873
H	1.267989	2.003059	-0.418707
C	0.093665	5.574579	-0.963132
H	-0.915218	5.965599	-1.159455
H	0.740074	5.793374	-1.815638
H	0.497084	6.062884	-0.063599
C	2.853001	-0.328906	-0.421035
O	2.669871	0.329144	-1.434148
C	4.122129	-1.029751	-0.096016
C	6.546861	-2.316109	0.431300
C	5.173252	-0.956606	-1.022611
C	4.289376	-1.750012	1.097148
C	5.500860	-2.390455	1.357032
C	6.381759	-1.598655	-0.758772
H	5.030248	-0.396471	-1.940586
H	3.476452	-1.803819	1.811917
H	5.629285	-2.946692	2.280926
H	7.193485	-1.540703	-1.477938
H	7.489144	-2.816240	0.636883
O	-2.293252	-0.153786	0.378815

S	-3.884799	-0.324134	0.323845
O	-4.405025	-0.011901	1.645451
O	-4.442010	0.258364	-0.890874
C	-3.988919	-2.193802	0.127355
F	-3.491664	-2.549686	-1.058309
F	-3.308755	-2.797206	1.101920
F	-5.276061	-2.550596	0.194833
H	2.812755	3.173937	2.477597

TS_{II}-GluN (3-*O*-benzoyl-2,4,6-tri-*O*-methyl-ᴅ-glucopyranose)
E = -2034.930490
H = -2034.525906
qh-*G* = -2034.576062
N_{imag} = 1

C	0.881055	0.234380	1.138007
C	-0.340073	2.304404	0.417497
C	1.021663	1.731424	0.798153
C	-0.348841	-0.533251	0.569734
H	0.795706	0.177638	2.224142
H	0.022241	-1.405410	0.022002
O	-1.181664	-0.976155	1.624935
O	2.118964	-0.478148	0.881351
O	1.501556	2.467173	1.911751
H	-1.057292	2.194989	1.241028
C	-0.299648	3.767960	0.026234
H	-0.082566	4.363377	0.924077
H	-1.288459	4.061142	-0.357171
O	0.697012	3.972587	-0.958356
O	-0.792686	1.548978	-0.724099
C	-1.205195	0.276204	-0.455220
H	-1.269765	-0.244908	-1.407053
C	-0.684675	-2.134617	2.290437
H	0.270053	-1.938909	2.796408
H	-0.550666	-2.960153	1.578238
H	-1.435598	-2.407177	3.033755
C	2.908529	2.363709	2.124095
H	3.196849	1.353188	2.438419
H	1.690101	1.865589	-0.057669
C	0.790261	5.331948	-1.358541
H	1.581830	5.390276	-2.108712
H	1.046563	5.977538	-0.505480
H	-0.155615	5.680295	-1.798738
C	2.489375	-0.672005	-0.404594
O	1.788409	-0.342675	-1.349746
C	3.816371	-1.324614	-0.522931
C	6.310429	-2.544585	-0.841810
C	4.293871	-1.623312	-1.807977
C	4.592880	-1.638777	0.603474
C	5.837396	-2.247034	0.440528
C	5.537485	-2.232217	-1.965726
H	3.683364	-1.373610	-2.669266
H	4.222918	-1.405855	1.595310
H	6.437952	-2.488666	1.312549
H	5.904649	-2.462683	-2.961449
H	7.280092	-3.018647	-0.965236
O	-2.598684	0.374848	0.091277
S	-3.846154	-0.185987	-0.723384
O	-5.018519	0.551135	-0.279026
O	-3.531442	-0.365651	-2.136807
C	-3.992093	-1.916793	0.000890
F	-2.880703	-2.610377	-0.269663

F	-4.178666	-1.860618	1.318260
F	-5.038826	-2.524135	-0.569695
H	3.155681	3.074224	2.915469

RC$_{II}$-GluN (3-*O*-benzoyl-2,4,6-tri-*O*-methyl-D-glucopyranose)

E = -2034.932335
H = -2034.527379
qh-*G* = -2034.578885
N_{imag} = 0

C	1.093109	0.662145	1.354187
C	-0.240427	2.499876	0.266683
C	1.174013	1.991169	0.570699
C	-0.306075	-0.004779	1.336373
H	1.273391	0.892413	2.403966
H	-0.192216	-1.087416	1.456444
O	-1.007495	0.551033	2.441074
O	2.182958	-0.238929	1.047150
O	1.838340	2.993898	1.320092
H	-0.803623	2.653202	1.194012
C	-0.259368	3.795641	-0.518553
H	0.098536	4.603774	0.134734
H	-1.296530	4.017914	-0.811658
O	0.566303	3.681473	-1.663391
O	-0.887207	1.494242	-0.540749
C	-1.071520	0.257753	0.022958
H	-0.861021	-0.496317	-0.728081
C	-1.675264	-0.398472	3.273575
H	-0.959439	-1.120943	3.687362
H	-2.459644	-0.927525	2.723214
H	-2.124889	0.173795	4.087157
C	3.255893	2.845128	1.354314
H	3.554665	1.942061	1.901990
H	1.685012	1.835215	-0.385302
C	0.587092	4.872655	-2.435224
H	1.248166	4.691183	-3.285499
H	0.972893	5.718312	-1.846484
H	-0.418916	5.123294	-2.802918
C	2.265204	-0.811603	-0.172315
O	1.404922	-0.674077	-1.028072
C	3.500319	-1.616464	-0.340336
C	5.801297	-3.148738	-0.750182
C	3.676940	-2.311089	-1.546626
C	4.481046	-1.691313	0.661199
C	5.628456	-2.456374	0.452919
C	4.824506	-3.075378	-1.749487
H	2.911638	-2.243004	-2.312522
H	4.343555	-1.152611	1.591652
H	6.387337	-2.512466	1.227785
H	4.958475	-3.612491	-2.683794
H	6.695994	-3.744132	-0.909111
O	-2.522399	0.115756	0.395005
S	-3.563707	-0.411172	-0.697561
O	-4.855815	0.184208	-0.395249
O	-2.999788	-0.406096	-2.041536
C	-3.664237	-2.216307	-0.177352
F	-2.440696	-2.759458	-0.221665
F	-4.144119	-2.314033	1.065095
F	-4.469895	-2.868046	-1.018558
H	3.649356	3.725067	1.867028

TS$_{III}$-GluN (3-*O*-benzoyl-2,4,6-tri-*O*-methyl-D-glucopyranose)

E = -2034.915862
H = -2034.512378
qh-*G* = -2034.563728
N_{imag} = 1

C	1.225869	0.582805	1.418955
C	0.183923	2.539843	0.158758
C	1.489863	1.918272	0.676946
C	-0.227738	0.066908	1.235381
H	1.331882	0.756626	2.489097
H	-0.278493	-1.010897	1.411348
O	-0.996518	0.795260	2.174767
O	2.239613	-0.402002	1.140180
O	2.068208	2.887516	1.524949
H	-0.496888	2.775026	0.978007
C	0.383638	3.754965	-0.717273
H	0.722593	4.581796	-0.077464
H	-0.580717	4.036010	-1.165600
O	1.339313	3.461282	-1.715764
O	-0.474086	1.518441	-0.684227
C	-0.689566	0.362252	-0.182411
H	-0.967353	-0.398989	-0.895839
C	-1.848241	-0.003923	3.004375
H	-1.252321	-0.710688	3.595302
H	-2.586500	-0.537499	2.402298
H	-2.354501	0.696077	3.670755
C	3.462450	2.684455	1.761328
H	3.644324	1.752832	2.311969
H	2.139859	1.733028	-0.185825
C	1.561072	4.555206	-2.597568
H	2.310867	4.230694	-3.321638
H	1.935364	5.431616	-2.049360
H	0.636431	4.828191	-3.125424
C	2.258172	-0.975178	-0.080181
O	1.392530	-0.741683	-0.916363
C	3.398973	-1.890421	-0.283461
C	5.526889	-3.635046	-0.750708
C	3.479765	-2.588277	-1.498814
C	4.387205	-2.067481	0.698314
C	5.448445	-2.939586	0.460702
C	4.542409	-3.458962	-1.729579
H	2.708942	-2.440627	-2.247629
H	4.322243	-1.525820	1.634982
H	6.213773	-3.076989	1.218622
H	4.604259	-3.999571	-2.669172
H	6.355175	-4.314048	-0.932071
O	-2.821136	0.441627	0.170248
S	-3.687978	-0.257669	-0.848211
O	-4.935622	0.459023	-1.154007
O	-2.920154	-0.806951	-1.985072
C	-4.247195	-1.781600	0.091991
F	-3.181598	-2.509179	0.481343
F	-4.943197	-1.438549	1.190276
F	-5.027153	-2.554352	-0.680829
H	3.799666	3.531023	2.361371

PC-GluN (3-*O*-benzoyl-2,4,6-tri-*O*-methyl-D-glucopyranose)
E = -2034.913940
H = -2034.509564
qh-*G* = -2034.561760
N_{imag} = 0

C	1.488883	-0.647484	-1.522352

C	1.682200	-2.543047	0.206645
C	2.437178	-1.516939	-0.650576
C	0.003544	-0.910475	-1.237903
H	1.676487	-0.836401	-2.578046
H	-0.614745	-0.092418	-1.622066
O	-0.316243	-2.142915	-1.843196
O	1.789328	0.759385	-1.346706
O	3.325129	-2.276435	-1.441570
H	1.253896	-3.320590	-0.426963
C	2.531899	-3.165159	1.292151
H	3.274250	-3.818390	0.811975
H	1.891716	-3.783851	1.937835
O	3.161971	-2.141358	2.035422
O	0.541606	-1.895883	0.894794
C	-0.153765	-1.015227	0.263991
H	-1.033533	-0.659694	0.794052
C	-1.705616	-2.301955	-2.194549
H	-1.994776	-1.535225	-2.922802
H	-2.347171	-2.241488	-1.312037
H	-1.778818	-3.290242	-2.649771
C	4.441834	-1.527798	-1.927176
H	4.121641	-0.715722	-2.592937
H	3.000125	-0.856785	0.020313
C	3.962749	-2.648504	3.096676
H	4.405092	-1.785969	3.598695
H	4.761186	-3.297689	2.709904
H	3.351967	-3.215571	3.813021
C	1.406492	1.328243	-0.206342
O	0.722231	0.721330	0.641797
C	1.844921	2.713505	-0.018661
C	2.648075	5.349076	0.388210
C	1.373825	3.422260	1.099756
C	2.719008	3.325240	-0.933944
C	3.117555	4.642953	-0.725325
C	1.777454	4.739393	1.298859
H	0.696861	2.937002	1.794101
H	3.079494	2.771415	-1.793181
H	3.793239	5.119933	-1.428374
H	1.413656	5.291482	2.159670
H	2.960910	6.377123	0.546019
O	-3.375006	-1.627968	0.613135
S	-3.743673	-0.264641	1.072454
O	-4.925787	-0.182062	1.954925
O	-2.577108	0.562588	1.478456
C	-4.316973	0.576029	-0.505499
F	-3.318504	0.621725	-1.416169
F	-5.349598	-0.085635	-1.061122
F	-4.711322	1.840511	-0.265000
H	5.062232	-2.228853	-2.487095

P-GluN (3-*O*-benzoyl-2,4,6-tri-*O*-methyl-D-glucopyranose)
E = -2034.926780
H = -2034.521341
qh-*G* = -2034.572585
N_{imag} = 0

C	2.735015	-1.937534	1.084996
C	4.120352	-2.046914	1.113347
C	4.149230	-2.460857	-1.282712
H	2.229538	-2.471151	-2.260012
H	2.180369	-1.712458	1.987161
H	4.651808	-1.918470	2.050630

H	4.703915	-2.653934	-2.195246
H	5.909394	-2.380421	-0.040742
C	-1.473527	-1.584225	0.944195
C	-2.502495	-0.059032	-0.861375
C	-1.900098	-0.147114	0.554762
C	-2.026242	-2.653936	-0.001185
H	-1.725148	-1.768648	1.986001
H	-1.617009	-3.639560	0.261205
O	-3.426664	-2.683167	-0.057082
O	-0.010356	-1.715463	0.928135
O	-2.917030	0.275764	1.446288
H	-3.571889	-0.260552	-0.775428
C	-2.267371	1.281896	-1.547295
H	-2.676140	1.226545	-2.567896
H	-1.189565	1.478816	-1.609799
O	-2.924322	2.291191	-0.803678
O	-1.956963	-1.025963	-1.807327
C	-1.528390	-2.239055	-1.377487
H	-1.676277	-2.978715	-2.160814
C	-4.057679	-3.200379	1.121534
H	-3.971422	-2.496833	1.956537
H	-5.109723	-3.329958	0.865676
H	-3.622425	-4.168590	1.399240
C	-2.423179	1.021294	2.559310
H	-1.735528	0.422656	3.172207
H	-1.020784	0.497872	0.627827
C	-2.793723	3.569387	-1.411299
H	-3.305844	4.283834	-0.763009
H	-3.260648	3.579997	-2.407450
H	-1.737837	3.855503	-1.506896
C	0.612382	-1.987862	-0.175202
O	-0.004614	-2.224902	-1.285141
C	2.055333	-2.098211	-0.136476
C	4.826289	-2.305301	-0.066706
C	2.763898	-2.361415	-1.323524
O	3.223873	1.500320	-0.118863
S	1.794251	1.420613	0.250836
O	0.926269	0.724286	-0.736952
O	1.507531	1.074567	1.662665
C	1.213881	3.201306	0.132351
F	1.333022	3.672402	-1.125210
F	-0.082003	3.306859	0.492252
F	1.934981	3.998396	0.944703
H	-3.294004	1.294674	3.157908

P_{bare}-GluN (3-*O*-benzoyl-2,4,6-tri-*O*-methyl-D-glucopyranose)
E = -1073.315809
H = -1072.943397
qh-*G* = -1072.984969
N_{imag} = 0

C	-1.005612	-1.098558	0.667670
C	-2.097854	0.863416	-0.600607
C	-1.635078	0.310614	0.761706
C	-1.276540	-1.790657	-0.670978
H	-1.299384	-1.703969	1.521872
H	-0.735830	-2.745129	-0.729011
O	-2.645212	-1.958644	-0.921906
O	0.453436	-0.974230	0.825921
O	-2.794971	0.284578	1.569378
H	-3.124883	0.529524	-0.759877
C	-2.045270	2.377309	-0.644757

H	-2.764600	2.772658	0.088355
H	-2.345639	2.721910	-1.644630
O	-0.729920	2.804271	-0.340960
O	-1.318946	0.395131	-1.741087
C	-0.741915	-0.829968	-1.721689
H	-0.686526	-1.231888	-2.730117
C	-3.251668	-3.050183	-0.215632
H	-3.296453	-2.852221	0.860825
H	-2.702273	-3.980454	-0.403409
H	-4.264864	-3.133723	-0.609212
C	-2.533511	0.430141	2.967309
H	-1.923902	-0.397226	3.353779
H	-0.884828	0.983398	1.194446
C	-0.588377	4.219174	-0.377647
H	0.450618	4.440612	-0.125444
H	-1.253403	4.700554	0.353528
H	-0.812746	4.610706	-1.379869
C	1.207080	-0.710622	-0.199595
O	0.751340	-0.655813	-1.404540
C	2.616428	-0.481523	0.032230
C	5.324534	-0.027052	0.469523
C	3.461658	-0.198084	-1.059219
C	3.130380	-0.536716	1.343536
C	4.484835	-0.309081	1.554391
C	4.813956	0.028518	-0.833251
H	3.054428	-0.155515	-2.062718
H	2.472088	-0.754103	2.176647
H	4.888708	-0.349109	2.560627
H	5.471100	0.249031	-1.667954
H	6.381918	0.151041	0.640523
H	-3.505059	0.420612	3.463242

R_{α}-ManN (3-*O*-benzoyl-2,4,6-tri-*O*-methyl-D-mannopyranose)
E = -2034.944948
H = -2034.540563
qh-*G* = -2034.591160
N_{imag} = 0

C	-0.770412	0.012767	-0.122697
C	1.216967	1.515068	-0.324366
C	-0.318367	1.450040	-0.344345
C	-0.181487	-0.546354	1.177236
H	-0.466186	-0.610030	-0.967206
O	-2.206933	0.025125	-0.045269
O	-0.748192	1.903174	-1.619888
H	1.602817	0.958296	-1.186951
C	1.753730	2.929743	-0.406932
H	1.521818	3.332292	-1.403392
H	2.847142	2.905444	-0.289002
O	1.161713	3.729797	0.600785
C	1.339380	-0.352023	1.173155
C	-1.743988	2.924978	-1.579052
H	-2.661435	2.561294	-1.101050
H	-0.715338	2.086707	0.451122
C	1.628298	5.070919	0.569013
H	1.396102	5.546631	-0.395373
H	2.714336	5.115525	0.736807
H	1.115498	5.606535	1.370748
C	-2.875137	-1.054009	-0.513661
H	1.780967	-0.638266	2.125374
O	1.751355	0.922596	0.885925
O	1.825068	-1.309785	0.127827

S	3.275200	-1.990801	0.251471
O	3.208018	-3.262100	-0.450516
O	3.794336	-1.874727	1.608451
C	4.306989	-0.850127	-0.839365
F	5.507970	-1.409001	-1.005298
F	3.712615	-0.704492	-2.026571
F	4.445470	0.343197	-0.262054
O	-2.315776	-2.036984	-0.972028
C	-4.346612	-0.896471	-0.391440
C	-7.126521	-0.684829	-0.191781
C	-4.924289	0.249446	0.177634
C	-5.166180	-1.935113	-0.857909
C	-6.552282	-1.827948	-0.758963
C	-6.311854	0.351510	0.276029
H	-4.289676	1.049987	0.539960
H	-4.706322	-2.815396	-1.294524
H	-7.184315	-2.632909	-1.122273
H	-6.757390	1.238313	0.717125
H	-8.206974	-0.602167	-0.114275
O	-0.653200	0.169496	2.306152
C	-1.665580	-0.500930	3.059693
H	-2.584059	-0.618947	2.474542
H	-1.313026	-1.484334	3.397151
H	-1.866555	0.129596	3.927851
H	-0.401301	-1.615394	1.268964
H	-1.955971	3.191270	-2.616764

TS_I-ManN (3-*O*-benzoyl-2,4,6-tri-*O*-methyl-D-mannopyranose)
E = -2034.930058
H = -2034.525991
qh-*G* = -2034.576341
N_{imag} = 1

C	-0.801581	-0.032282	-0.366117
C	1.185047	1.455717	-0.034780
C	-0.355169	1.433175	-0.246938
C	-0.256440	-0.865653	0.805092
H	-0.415562	-0.426327	-1.312005
O	-2.206599	-0.376125	-0.318790
O	-0.567044	2.118911	-1.470656
H	1.641862	1.098246	-0.965113
C	1.738224	2.839667	0.245866
H	1.651729	3.455968	-0.659839
H	2.806563	2.743738	0.488759
O	1.034809	3.429425	1.323838
C	1.251382	-0.693581	0.976799
C	-1.108288	3.433965	-1.349587
H	-2.065227	3.408739	-0.821511
H	-0.862464	1.926880	0.585785
C	1.544429	4.710083	1.667887
H	0.937437	5.083179	2.495371
H	1.471261	5.405857	0.818808
H	2.595221	4.644100	1.985434
C	-3.279366	0.332365	-0.712463
H	1.625192	-1.203659	1.862407
O	1.644920	0.612385	1.046657
O	1.844458	-1.372714	-0.221961
S	3.292492	-2.064716	-0.142049
O	3.303315	-3.144022	-1.116315
O	3.703219	-2.260292	1.242817
C	4.380893	-0.703971	-0.861454
F	5.610916	-1.196403	-1.023895

F	3.892672	-0.317373	-2.042753
F	4.423025	0.340457	-0.034307
O	-3.256223	1.447768	-1.196307
C	-4.537314	-0.436043	-0.467379
C	-6.953600	-1.784565	-0.045937
C	-4.539233	-1.709953	0.121130
C	-5.751492	0.157530	-0.842661
C	-6.954879	-0.514756	-0.633153
C	-5.745159	-2.379672	0.330435
H	-3.601970	-2.168955	0.412426
H	-5.737855	1.143235	-1.295534
H	-7.891888	-0.050222	-0.926421
H	-5.742116	-3.365240	0.787091
H	-7.891197	-2.308447	0.117969
O	-0.870021	-0.406620	1.994748
C	-1.121859	-1.428799	2.957596
H	-1.793342	-2.192124	2.543422
H	-0.192801	-1.906280	3.295799
H	-1.602208	-0.939743	3.806676
H	-0.487585	-1.921903	0.625929
H	-1.267929	3.790011	-2.369672

RC$_{I}$-ManN (3-*O*-benzoyl-2,4,6-tri-*O*-methyl-D-mannopyranose)
E = -2034.938156
H = -2034.533067
qh-*G* = -2034.584692
N_{imag} = 0

C	-0.786487	-0.152163	-0.354069
C	1.111918	1.430217	-0.308962
C	-0.426323	1.334939	-0.366736
C	-0.222818	-0.857457	0.881394
H	-0.329399	-0.607513	-1.237762
O	-2.174729	-0.498935	-0.513586
O	-0.876910	1.894563	-1.591799
H	1.529467	0.949338	-1.202292
C	1.640818	2.848951	-0.262966
H	1.436631	3.333380	-1.229535
H	2.731346	2.816268	-0.122770
O	1.024789	3.561694	0.793297
O	1.627462	0.750440	0.864868
C	1.283715	-0.567743	0.998744
H	1.695207	-0.920599	1.942453
C	-1.522190	3.165412	-1.473181
H	-2.416938	3.085424	-0.846933
H	-0.859489	1.851493	0.487524
C	1.512655	4.891026	0.905633
H	0.975858	5.358971	1.733383
H	1.327084	5.459339	-0.018007
H	2.591658	4.898647	1.118048
C	-3.203631	0.178898	0.042252
O	-3.103262	1.221595	0.662703
O	-0.873506	-0.367973	2.033230
C	-0.934932	-1.298788	3.113606
H	-1.495449	-0.806760	3.909998
H	0.064456	-1.558998	3.485841
H	-1.457506	-2.213592	2.804496
H	-0.371281	-1.939971	0.781249
C	-4.502064	-0.505005	-0.222503
C	-6.989395	-1.708944	-0.661729
C	-4.582436	-1.717421	-0.924716
C	-5.673097	0.100110	0.257783

C	-6.912049	-0.500109	0.038650
C	-5.824210	-2.315330	-1.142057
H	-3.678218	-2.184737	-1.296892
H	-5.597927	1.036944	0.799565
H	-7.815675	-0.027327	0.412352
H	-5.882701	-3.253616	-1.686081
H	-7.954929	-2.176873	-0.832571
O	1.898355	-1.387293	-0.097013
S	3.376343	-1.997134	0.061276
O	3.787339	-2.016653	1.459678
O	3.438604	-3.177730	-0.785260
C	4.412026	-0.680779	-0.806036
F	3.884007	-0.409708	-2.002693
F	4.452605	0.432633	-0.075193
F	5.648349	-1.160086	-0.960721
H	-1.803890	3.458088	-2.486896

TS_{II}-ManN (3-*O*-benzoyl-2,4,6-tri-*O*-methyl-D-mannopyranose)
E = -2034.926980
H = -2034.522475
qh-*G* = -2034.572803
N_{imag} = 1

C	-1.046489	0.064568	-1.133514
C	0.957212	1.437590	-0.548660
C	-0.564167	1.453691	-0.697933
C	-0.238042	-1.176187	-0.610340
H	-0.947902	0.056664	-2.220637
O	-2.460962	-0.111948	-0.957265
O	-0.949216	2.376946	-1.711463
H	1.440014	1.110304	-1.479288
C	1.554810	2.775911	-0.165725
H	1.433760	3.465896	-1.013902
H	2.630551	2.650352	0.023754
O	0.903401	3.280199	0.985166
O	1.260087	0.496992	0.508132
C	1.029177	-0.815041	0.214579
H	1.046665	-1.371540	1.150033
C	-1.697801	3.497160	-1.237216
H	-1.127763	4.070688	-0.497166
H	-0.991128	1.732550	0.263841
C	1.429530	4.532940	1.399727
H	0.866123	4.838370	2.283827
H	1.311899	5.290835	0.610919
H	-2.647014	3.172542	-0.791435
C	-3.007489	-0.053811	0.281257
O	-2.389900	0.258480	1.283996
O	-1.070994	-2.016936	0.155071
C	-0.712818	-3.397312	0.096578
H	-1.473419	-3.934338	0.665559
H	0.270688	-3.583179	0.547335
H	-0.706744	-3.751762	-0.942869
H	0.102086	-1.725008	-1.495570
C	-4.456924	-0.386626	0.260697
C	-7.184013	-1.003607	0.326606
C	-5.116435	-0.781508	-0.913388
C	-5.169017	-0.303604	1.466609
C	-6.528218	-0.610875	1.498674
C	-6.476981	-1.088298	-0.877233
H	-4.564880	-0.847776	-1.844011
H	-4.646638	0.001141	2.367279
H	-7.075815	-0.545164	2.434341

H	-6.985145	-1.394397	-1.787069
H	-8.243312	-1.243693	0.351700
O	2.168541	-1.342338	-0.630622
S	3.540062	-1.811974	0.052596
O	3.326887	-2.222157	1.435467
O	4.211807	-2.665406	-0.915751
C	4.539099	-0.207944	0.102246
F	4.372413	0.466145	-1.040088
F	4.156530	0.550506	1.127447
F	5.827644	-0.530315	0.244262
H	2.495267	4.447495	1.656835

RC_{II}-ManN (3-*O*-benzoyl-2,4,6-tri-*O*-methyl-D-mannopyranose)
E = -2034.935117
H = -2034.530091
qh-*G* = -2034.581442
N_{imag} = 0

C	0.808553	0.277453	1.156494
C	-0.717651	2.104763	0.308028
C	0.678528	1.786896	0.852432
C	-0.327423	-0.583398	0.527156
H	0.731164	0.163176	2.238675
O	2.125332	-0.248851	0.897385
O	0.850981	2.567264	2.023805
H	-1.487837	1.853275	1.044809
C	-0.899035	3.560229	-0.072579
H	-0.909481	4.160480	0.847938
H	-1.870912	3.676551	-0.575335
O	0.154563	3.973981	-0.922885
O	-0.922913	1.325896	-0.894047
C	-0.836023	-0.033858	-0.804219
H	-0.279286	-0.434402	-1.645876
C	2.210925	2.691432	2.434952
H	2.629607	1.724726	2.743109
H	1.412856	2.076149	0.094479
C	0.035627	5.336247	-1.305635
H	-0.897394	5.510620	-1.861446
H	0.888237	5.562692	-1.949480
H	0.056135	5.996722	-0.426048
C	2.582176	-0.338812	-0.369797
O	1.953448	0.062512	-1.335674
O	0.046580	-1.918377	0.289762
C	0.190428	-2.703270	1.474058
H	0.358859	-3.729045	1.142645
H	-0.722552	-2.658650	2.081891
H	1.047573	-2.368842	2.070111
H	-1.152545	-0.547138	1.249248
C	3.927569	-0.961952	-0.440067
C	6.457986	-2.126812	-0.670449
C	4.591157	-1.430984	0.704358
C	4.535762	-1.079581	-1.698824
C	5.797675	-1.660187	-1.812503
C	5.853711	-2.011819	0.585826
H	4.118843	-1.341141	1.675748
H	4.010811	-0.713938	-2.574969
H	6.266151	-1.749485	-2.788199
H	6.365797	-2.374975	1.472085
H	7.441471	-2.579697	-0.759435
O	-2.190804	-0.661633	-1.123585
S	-3.553087	-0.269699	-0.397960
O	-3.331999	0.435093	0.863757

O	-4.510653	0.229168	-1.374831
C	-4.099145	-2.012577	0.050718
F	-4.193310	-2.767381	-1.044640
F	-3.217486	-2.558313	0.894684
F	-5.293772	-1.940518	0.646610
H	2.218047	3.375623	3.285613

TS_{III}-ManN (3-*O*-benzoyl-2,4,6-tri-*O*-methyl-D-mannopyranose)
E = -2034.928285
H = -2034.524523
qh-*G* = -2034.575628
N_{imag} = 1

C	0.805679	0.197433	1.148221
C	-0.610839	2.101660	0.233436
C	0.710788	1.724007	0.912103
C	-0.308013	-0.608853	0.407796
H	0.666720	0.024371	2.215568
O	2.123329	-0.330599	0.920501
O	0.743736	2.442191	2.131198
H	-1.469278	1.848360	0.861205
C	-0.705547	3.559955	-0.157384
H	-0.798966	4.153990	0.762529
H	-1.613205	3.709318	-0.760865
O	0.448520	3.935322	-0.883219
O	-0.704175	1.324668	-1.004883
C	-0.672588	0.021716	-0.916628
H	-0.355426	-0.474142	-1.823697
C	2.049522	2.545253	2.698989
H	2.440834	1.561940	2.990195
H	1.531156	2.042629	0.261005
C	0.418295	5.299509	-1.281728
H	1.345758	5.492476	-1.824634
H	0.360513	5.963505	-0.407034
H	-0.439186	5.498530	-1.940518
C	2.600725	-0.396635	-0.341797
O	1.974683	0.019812	-1.304317
O	0.031649	-1.944880	0.154315
C	0.072589	-2.767024	1.323522
H	0.217613	-3.788963	0.970975
H	-0.871523	-2.698861	1.877765
H	0.907378	-2.485169	1.975645
H	-1.185802	-0.545729	1.062824
C	3.947930	-1.009369	-0.410414
C	6.486587	-2.153860	-0.635895
C	4.607930	-1.482524	0.734577
C	4.563287	-1.112115	-1.667247
C	5.829627	-1.683004	-1.778183
C	5.875024	-2.053225	0.618158
H	4.129996	-1.403536	1.704188
H	4.040775	-0.743203	-2.543442
H	6.304197	-1.761518	-2.751777
H	6.385109	-2.419636	1.504132
H	7.473701	-2.599047	-0.722982
O	-2.365991	-0.596741	-1.236531
S	-3.624677	-0.192506	-0.447017
O	-3.307237	0.512866	0.805227
O	-4.675231	0.347176	-1.311914
C	-4.238183	-1.882878	0.089369
F	-4.483184	-2.656590	-0.974646
F	-3.317740	-2.483088	0.860207
F	-5.368230	-1.744731	0.796920

H	1.952572	3.172612	3.586675

PC-ManN (3-*O*-benzoyl-2,4,6-tri-*O*-methyl-D-mannopyranose)
E = -2034.927236
H = -2034.523143
qh-*G* = -2034.575400
N_{imag} = 0

C	0.849421	0.104804	1.182319
C	-0.409519	2.071692	0.128439
C	0.794680	1.640900	0.975363
C	-0.187758	-0.646110	0.299663
H	0.641237	-0.111843	2.229214
O	2.172209	-0.422645	0.985015
O	0.650762	2.327707	2.200165
H	-1.355283	1.844863	0.628879
C	-0.382225	3.521272	-0.294984
H	-0.563093	4.136474	0.597590
H	-1.197681	3.701217	-1.010669
O	0.873964	3.815331	-0.871149
O	-0.412686	1.272326	-1.122682
C	-0.262756	0.013893	-1.030176
H	-0.290887	-0.543931	-1.956679
C	1.870741	2.442611	2.936382
H	2.254552	1.458007	3.232848
H	1.708125	1.962173	0.462424
C	0.970273	5.164862	-1.312570
H	1.969914	5.288889	-1.733255
H	0.835630	5.862210	-0.473636
H	0.218297	5.379909	-2.084619
C	2.636816	-0.522559	-0.271744
O	1.980215	-0.150823	-1.246269
O	0.098911	-1.999846	0.102338
C	-0.155845	-2.820640	1.247753
H	-0.003661	-3.849510	0.920407
H	-1.187942	-2.690728	1.593199
H	0.542962	-2.590608	2.060589
H	-1.170625	-0.495359	0.774522
C	3.990645	-1.098127	-0.360056
C	6.547864	-2.185806	-0.606897
C	4.707364	-1.467616	0.789881
C	4.557381	-1.274047	-1.632448
C	5.834080	-1.817639	-1.752925
C	5.984518	-2.010359	0.661771
H	4.265929	-1.330103	1.770232
H	3.991021	-0.984748	-2.511049
H	6.272606	-1.955277	-2.736556
H	6.540301	-2.297084	1.549330
H	7.543324	-2.609877	-0.702424
O	-2.690533	-0.634052	-1.428457
S	-3.719399	-0.090278	-0.498732
O	-3.145634	0.476817	0.751285
O	-4.788182	0.706501	-1.130295
C	-4.598987	-1.633879	0.101379
F	-5.164607	-2.293221	-0.925229
F	-3.733095	-2.469679	0.709462
F	-5.565020	-1.320176	0.983817
H	1.637035	3.023237	3.829825

P-ManN (3-*O*-benzoyl-2,4,6-tri-*O*-methyl-D-mannopyranose)
E = -2034.931314
H = -2034.525874

qh-*G* = -2034.577083
N$_{imag}$ = 0

C	2.853152	1.494495	0.989988
C	3.611163	2.659388	1.014939
C	4.036729	2.448702	-1.369643
H	3.144913	0.743273	-2.338020
H	2.370982	1.129861	1.888225
H	3.733911	3.202392	1.946388
H	4.492542	2.826875	-2.279034
H	4.786595	4.050689	-0.137288
C	0.627083	-2.087086	0.885496
C	-1.261601	-2.309649	-0.852832
C	-0.866561	-1.827746	0.556987
C	1.265481	-3.059345	-0.100581
H	0.713049	-2.406539	1.921368
O	2.641240	-3.261789	0.098059
H	0.708429	-4.006099	-0.087697
O	1.398120	-0.843153	0.851789
O	-1.655318	-2.578884	1.461517
H	-1.542962	-3.365193	-0.772895
C	-2.402195	-1.513740	-1.478548
H	-2.572690	-1.892995	-2.497603
H	-2.113532	-0.456573	-1.539852
O	-3.561732	-1.679349	-0.685305
O	-0.196505	-2.213788	-1.839612
C	1.095532	-2.398686	-1.455916
H	1.664326	-2.864809	-2.256996
C	2.965231	-4.076557	1.230227
H	2.712706	-3.574410	2.171325
H	2.443546	-5.039884	1.173129
H	4.042760	-4.237925	1.186673
C	-2.068805	-1.837067	2.610382
H	-1.207460	-1.480351	3.190891
H	-1.055417	-0.756164	0.657270
C	-4.682951	-0.997842	-1.233161
H	-5.519427	-1.162769	-0.550578
H	-4.937575	-1.396524	-2.226338
H	-4.487429	0.079352	-1.317360
C	1.905717	-0.413503	-0.259396
O	1.780694	-1.044634	-1.378465
C	2.690694	0.803159	-0.224369
C	4.200849	3.136447	-0.160889
C	3.283733	1.281145	-1.407391
O	0.017680	3.460802	-0.096526
S	-0.588047	2.160094	0.260880
O	-0.400287	1.079271	-0.744003
O	-0.402871	1.723482	1.664747
C	-2.432326	2.495508	0.169774
F	-2.806273	2.842974	-1.077815
F	-3.135160	1.398381	0.521553
F	-2.783057	3.494955	1.002282
H	-2.658629	-2.521453	3.222612

P$_{bare}$-ManN (3-*O*-benzoyl-2,4,6-tri-*O*-methyl-D-mannopyranose)
E = -1073.320145
H = -1072.947685
qh-*G* = -1072.989161
N$_{imag}$ = 0

C	0.895776	1.222263	0.632971
C	2.474701	-0.337895	-0.663490
C	1.886848	0.034265	0.709490

C	0.953978	1.939094	-0.713233
H	1.040541	1.889830	1.479258
O	-0.471602	0.722618	0.825562
O	3.000326	0.369054	1.510525
H	3.332980	0.315982	-0.851837
C	2.946716	-1.777844	-0.713970
H	3.787465	-1.891727	-0.013700
H	3.306793	-2.004091	-1.727828
O	1.874982	-2.630000	-0.358345
O	1.542132	-0.168444	-1.766611
C	0.656599	0.862869	-1.740991
H	0.470020	1.234484	-2.745407
C	2.796650	0.157723	2.910659
H	1.984587	0.785557	3.299500
H	1.352324	-0.827654	1.125997
C	2.239464	-4.005348	-0.375904
H	3.053071	-4.205411	0.335820
H	2.556990	-4.314989	-1.381528
H	1.352972	-4.571789	-0.084065
C	-1.167519	0.296526	-0.184715
O	-0.721645	0.299567	-1.394255
O	-0.013052	2.944585	-0.860997
C	0.304530	4.175446	-0.198003
H	-0.494301	4.870717	-0.456541
H	1.265987	4.564361	-0.554325
H	0.336686	4.047867	0.890034
H	1.970419	2.322300	-0.875768
C	-2.498831	-0.209388	0.073013
C	-5.057358	-1.184157	0.560021
C	-2.995037	-0.238876	1.391553
C	-3.286568	-0.668852	-1.000851
C	-4.564247	-1.155087	-0.750186
C	-4.274603	-0.727018	1.627585
H	-2.381022	0.115847	2.211190
H	-2.894333	-0.641492	-2.010852
H	-5.176970	-1.511143	-1.571788
H	-4.664077	-0.753258	2.639981
H	-6.056199	-1.565228	0.750247
H	3.730616	0.435333	3.400864

R$_{\alpha}$-GluPMBz
(3-*O*-(4-methoxybenzoyl)4,6-*O*-ethylidene-2-*O*-methyl-D-glucopyranose)
E = -2148.285677
H = -2147.868192
qh-*G* = -2147.919236
N_{imag} = 0

C	-0.105013	0.508101	0.155905
C	1.642863	2.269530	0.424095
C	0.141883	1.977989	0.442775
C	0.681613	-0.331264	1.166632
H	0.179307	0.253464	-0.866743
O	-1.496375	0.222677	0.346074
O	-0.491020	2.797036	-0.530457
H	2.052357	2.029264	-0.562363
C	1.855359	3.750562	0.708425
H	2.901110	4.039711	0.590776
H	1.523951	3.995260	1.728467
O	1.106296	4.493873	-0.256242
C	2.156203	0.119736	1.245100
C	-0.278360	4.178305	-0.244533
H	-0.674995	4.381858	0.764257

H	-0.258349	2.220184	1.440528
C	-0.969071	4.997739	-1.307097
H	-0.549358	4.762269	-2.289948
H	-0.829941	6.063167	-1.103431
H	-2.039393	4.773453	-1.311542
C	-2.201969	-0.287767	-0.698112
H	2.698236	-0.375317	2.048352
O	2.314851	1.483341	1.424429
O	2.754277	-0.290469	-0.039901
S	4.321603	-0.645732	-0.125598
O	4.913972	-0.718127	1.204979
O	4.921797	0.130597	-1.198313
C	4.140655	-2.409355	-0.758693
F	5.363736	-2.898931	-0.981683
F	3.522977	-3.154912	0.158877
F	3.441475	-2.417488	-1.892511
O	-1.709678	-0.508326	-1.792873
C	-3.614566	-0.532712	-0.341565
C	-6.314640	-1.028492	0.239786
C	-4.129995	-0.256711	0.939205
C	-4.470227	-1.058423	-1.318415
C	-5.811973	-1.308674	-1.040990
C	-5.463468	-0.501267	1.227707
H	-3.480360	0.150709	1.705153
H	-4.075009	-1.271904	-2.306023
H	-6.449250	-1.715599	-1.816422
H	-5.871235	-0.291405	2.211521
O	-7.602347	-1.231745	0.616593
O	0.561805	-1.687990	0.818531
C	0.681863	-2.596530	1.913887
H	-0.071034	-2.377021	2.682053
H	1.682200	-2.563092	2.363524
H	0.508535	-3.593017	1.504926
H	0.259460	-0.150930	2.166439
C	-8.522723	-1.759509	-0.343641
H	-8.617616	-1.087045	-1.203376
H	-9.478387	-1.830881	0.175552

TS$_{I}$-GluPMBz
(3-*O*-(4-methoxybenzoyl)4,6-*O*-ethylidene-2-*O*-methyl-D-glucopyranose)
E = -2148.272390
H = -2147.856146
qh-*G* = -2147.906311
N_{imag} = 1

C	0.159555	0.250925	0.003176
C	-1.399581	2.191609	-0.515059
C	0.043535	1.719245	-0.400649
C	-0.752635	-0.614708	-0.887647
H	-0.123575	0.110647	1.051336
O	1.582497	0.020506	-0.097198
O	0.714370	2.545023	0.543798
H	-1.909364	2.096280	0.448353
C	-1.401424	3.654390	-0.941425
H	-2.407864	4.076143	-0.930698
H	-0.975912	3.757662	-1.950619
O	-0.628579	4.391980	0.007801
C	-2.125354	0.064590	-1.153646
C	0.701390	3.907591	0.125605
H	1.182638	3.964293	-0.865137
H	0.523919	1.829175	-1.386290
C	1.426622	4.732498	1.160633

H	0.920302	4.647144	2.127070
H	1.441269	5.782403	0.855048
H	2.456128	4.378012	1.262793
C	2.241407	-1.151635	-0.250309
H	-2.673226	-0.456975	-1.935992
O	-2.079712	1.399896	-1.502170
O	-2.880875	-0.078246	0.110642
S	-4.481738	-0.203316	0.085003
O	-4.974710	-0.355940	-1.279054
O	-5.041710	0.774744	1.004273
C	-4.625880	-1.881414	0.926770
F	-5.922481	-2.134493	1.130041
F	-4.106583	-2.827355	0.143392
F	-3.986222	-1.861864	2.095808
O	1.721645	-2.215818	-0.533706
C	3.699787	-0.957425	-0.061363
C	6.472970	-0.697567	0.285970
C	4.257206	0.276820	0.324244
C	4.550939	-2.050486	-0.267682
C	5.928929	-1.933273	-0.098697
C	5.626979	0.406151	0.495798
H	3.609038	1.129500	0.490805
H	4.123507	-3.002961	-0.563223
H	6.561370	-2.796610	-0.265196
H	6.066651	1.352378	0.795320
O	7.797915	-0.472884	0.480205
O	-0.992709	-1.862747	-0.285958
C	-1.140962	-2.943403	-1.204860
H	-0.229245	-3.063926	-1.799396
H	-2.005488	-2.796823	-1.865764
H	-1.304312	-3.837338	-0.600680
H	-0.278022	-0.729077	-1.871360
C	8.711866	-1.557041	0.290392
H	9.699593	-1.147990	0.502785
H	8.676668	-1.921451	-0.742348

RC$_{I}$-GluPMBZ
(3-*O*-(4-methoxybenzoyl)4,6-*O*-ethylidene-2-*O*-methyl-ᴅ-glucopyranose)
E = -2148.283053
H = -2147.865566
qh-*G* = -2147.916774
N_{imag} = 0

C	0.026348	0.429033	0.158980
C	-1.562775	2.278350	-0.305203
C	-0.083300	1.892723	-0.242571
C	-0.752255	-0.425033	-0.858017
H	-0.412225	0.290128	1.149791
H	-0.271714	-0.346185	-1.838673
O	-0.858230	-1.773280	-0.456896
O	1.379296	-0.028416	0.351652
O	0.566208	2.729220	0.707581
H	-2.032111	2.123689	0.672133
C	-1.663503	3.750082	-0.685026
H	-2.691045	4.113539	-0.629416
H	-1.277401	3.907964	-1.702759
O	-0.903158	4.502460	0.263843
O	-2.240238	1.480095	-1.292652
C	-2.177733	0.122835	-1.043346
H	-2.696598	-0.386045	-1.852901
C	0.055080	-2.672899	-1.088895
H	1.085514	-2.494318	-0.762393

H	-0.004934	-2.587802	-2.181422
H	-0.249801	-3.676165	-0.785586
C	0.457347	4.098974	0.325172
H	0.900283	4.208425	-0.678641
H	0.365936	2.043984	-1.231571
C	1.165157	4.942322	1.357338
H	0.697070	4.803807	2.336844
H	1.106254	5.998041	1.078092
H	2.217006	4.649201	1.417867
C	2.288736	0.038847	-0.651287
O	2.015323	0.422329	-1.780571
C	3.623333	-0.420473	-0.219460
C	6.194075	-1.277421	0.505283
C	4.658369	-0.449670	-1.163765
C	3.891529	-0.829234	1.101306
C	5.161231	-1.252508	1.460222
C	5.938049	-0.873695	-0.815404
H	4.452765	-0.135621	-2.181728
H	3.100537	-0.810359	1.842019
H	5.380112	-1.568007	2.475392
H	6.718128	-0.886680	-1.566686
O	7.400802	-1.705354	0.952215
O	-2.880299	-0.177514	0.227840
S	-4.439831	-0.557598	0.230240
O	-5.030387	0.022089	1.425422
O	-5.027995	-0.389166	-1.092934
C	-4.300472	-2.410645	0.537029
F	-3.742758	-3.001469	-0.520279
F	-3.565996	-2.641499	1.624682
F	-5.533045	-2.896030	0.719261
C	8.497794	-1.747855	0.034008
H	8.286991	-2.433821	-0.793719
H	9.347026	-2.115308	0.609848

TS$_{II}$-GluPMBz
(3-*O*-(4-methoxybenzoyl)4,6-*O*-ethylidene-2-*O*-methyl-ᴅ-glucopyranose)
E = -2148.272327
H = -2147.855511
qh-*G* = -2147.905416
N_{imag} = 1

C	0.213462	0.875150	0.835547
C	-1.351113	2.346471	-0.413724
C	0.098574	2.144953	0.004844
C	-0.826126	-0.245795	0.517231
H	0.046173	1.164011	1.874495
H	-0.271791	-1.144547	0.231077
O	-1.600223	-0.532142	1.666031
O	1.565977	0.366256	0.854649
O	0.538122	3.259573	0.778963
H	-2.021455	2.340763	0.453956
C	-1.477527	3.671475	-1.154576
H	-2.518876	3.928643	-1.355405
H	-0.927377	3.620299	-2.105863
O	-0.944640	4.702334	-0.320569
O	-1.670712	1.268214	-1.302200
C	-1.792195	0.047825	-0.684197
H	-1.683947	-0.708924	-1.457387
C	-0.904714	-1.312466	2.635977
H	-0.047858	-0.771429	3.058124
H	-0.551627	-2.253172	2.192070
H	-1.622608	-1.525015	3.429704

C	0.409863	4.470521	0.038734
H	1.004159	4.372802	-0.885129
H	0.703639	2.067107	-0.906163
C	0.882778	5.613679	0.903374
H	0.267897	5.676867	1.806524
H	0.805350	6.554205	0.350756
H	1.926149	5.455336	1.190319
C	2.067869	-0.157528	-0.292539
O	1.398712	-0.258029	-1.313178
C	3.471547	-0.584840	-0.154468
C	6.141378	-1.428032	0.012321
C	4.104011	-1.173365	-1.258015
C	4.196102	-0.421476	1.042200
C	5.515077	-0.837659	1.125417
C	5.428697	-1.595761	-1.186358
H	3.547007	-1.299551	-2.180516
H	3.720232	0.034330	1.902832
H	6.083909	-0.716072	2.041736
H	5.891300	-2.047412	-2.055197
O	7.432186	-1.802114	0.191409
O	-3.189949	-0.032854	-0.172192
S	-4.232532	-1.074596	-0.782970
O	-5.563008	-0.543554	-0.535701
O	-3.813061	-1.544874	-2.098720
C	-4.001967	-2.528850	0.389315
F	-2.743537	-2.974452	0.303459
F	-4.268094	-2.168075	1.642700
F	-4.840071	-3.503229	0.019350
C	8.130942	-2.405994	-0.902224
H	9.133122	-2.615383	-0.528662
H	8.188993	-1.719637	-1.754081

RC_{II}-GluPMBz
(3-*O*-(4-methoxybenzoyl)4,6-*O*-ethylidene-2-*O*-methyl-D-glucopyranose)
E = -2148.274624
H = -2147.857308
qh-*G* = -2147.908605
N_{imag} = 0

C	0.246548	1.306352	1.048045
C	-1.479896	2.451111	-0.343699
C	0.016963	2.348650	-0.041516
C	-0.913036	0.274767	1.150005
H	0.250944	1.822696	2.007710
H	-0.501554	-0.695548	1.445698
O	-1.793703	0.769823	2.149451
O	1.562942	0.719521	0.997533
O	0.508768	3.612072	0.397221
H	-2.050665	2.646744	0.569763
C	-1.702548	3.571987	-1.351987
H	-2.763619	3.779423	-1.501021
H	-1.248236	3.301322	-2.316613
O	-1.110869	4.767031	-0.837881
O	-1.887677	1.212681	-0.940709
C	-1.674712	0.074551	-0.181720
H	-1.194583	-0.671746	-0.806529
C	-2.178396	-0.182746	3.142736
H	-1.296388	-0.569430	3.670077
H	-2.740268	-1.012303	2.701584
H	-2.816203	0.354078	3.847325
C	0.277517	4.619502	-0.584067
H	0.772587	4.307667	-1.519079

H	0.527003	2.054183	-0.967827
C	0.816821	5.929091	-0.062193
H	0.299709	6.202906	0.862681
H	0.662927	6.716558	-0.805270
H	1.887602	5.835931	0.139762
C	1.889191	-0.096725	-0.033044
O	1.083819	-0.409869	-0.900180
C	3.289680	-0.551340	0.023168
C	5.941497	-1.464039	0.040929
C	3.749307	-1.426933	-0.970114
C	4.178935	-0.134777	1.032887
C	5.489143	-0.585153	1.042368
C	5.063517	-1.886289	-0.970933
H	3.065445	-1.747757	-1.748951
H	3.837460	0.543763	1.806103
H	6.183995	-0.270231	1.814356
H	5.391062	-2.562023	-1.751326
O	7.238309	-1.847736	0.135070
O	-3.004083	-0.462467	0.220196
S	-3.789220	-1.445506	-0.774495
O	-5.211689	-1.205795	-0.594940
O	-3.165992	-1.498837	-2.090673
C	-3.404256	-3.084890	0.065281
F	-2.077673	-3.253689	0.121585
F	-3.903759	-3.096150	1.303189
F	-3.949033	-4.074302	-0.645457
C	7.765205	-2.739426	-0.853070
H	7.714058	-2.289727	-1.850687
H	8.806399	-2.901448	-0.574816

TS_{III}-GluPMBz
(3-*O*-(4-methoxybenzoyl)4,6-*O*-ethylidene-2-*O*-methyl-D-glucopyranose)
E = -2148.254553
H = -2147.838802
qh-*G* = -2147.889859
N_{imag} = 1

C	0.438542	1.397010	1.110136
C	-1.071100	2.584008	-0.534090
C	0.347733	2.487980	0.036300
C	-0.780415	0.427958	1.043768
H	0.399963	1.858191	2.096432
H	-0.509949	-0.548666	1.452554
O	-1.805012	1.049627	1.794871
O	1.707130	0.724043	1.088059
O	0.695646	3.752808	0.574309
H	-1.812210	2.762849	0.244045
C	-1.110629	3.670483	-1.598700
H	-2.123343	3.852756	-1.960411
H	-0.462046	3.404417	-2.445631
O	-0.663458	4.873891	-0.973621
O	-1.365365	1.281702	-1.150864
C	-1.213255	0.251155	-0.405632
H	-1.247745	-0.696663	-0.921785
C	-2.431280	0.207926	2.772356
H	-1.691789	-0.142544	3.502691
H	-2.927679	-0.637697	2.293004
H	-3.171882	0.835083	3.270609
C	0.645482	4.766953	-0.434371
H	1.336580	4.476665	-1.242266
H	1.026308	2.240098	-0.791124
C	1.018534	6.084833	0.197119

H	0.308153	6.331823	0.991846
H	1.000645	6.874869	-0.558555
H	2.024613	6.021313	0.620706
C	1.954677	-0.111634	0.053243
O	1.102277	-0.332027	-0.807464
C	3.293891	-0.702618	0.079131
C	5.834748	-1.882059	0.054966
C	3.637946	-1.632009	-0.914072
C	4.241968	-0.367024	1.066846
C	5.497599	-0.949097	1.054517
C	4.896428	-2.223236	-0.934457
H	2.907847	-1.891174	-1.673456
H	3.988375	0.350681	1.838584
H	6.238076	-0.700095	1.807659
H	5.135950	-2.939105	-1.710731
O	7.083645	-2.396092	0.128679
O	-3.365636	-0.314036	-0.073350
S	-3.841309	-1.480683	-0.895056
O	-5.219831	-1.345267	-1.392725
O	-2.822819	-1.970485	-1.850738
C	-3.944873	-2.852706	0.379921
F	-2.740122	-3.055129	0.950759
F	-4.819593	-2.539130	1.352416
F	-4.339376	-4.003025	-0.190334
C	7.495479	-3.356335	-0.851761
H	8.519462	-3.619992	-0.588470
H	7.471171	-2.921088	-1.856508

PC-GluPMBz
(3-*O*-(4-methoxybenzoyl)4,6-*O*-ethylidene-2-*O*-methyl-D-glucopyranose)
E = -2148.270637
H = -2147.852865
qh-*G* = -2147.903934
N_{imag} = 0

C	-1.959275	0.231118	-1.051281
C	-3.534156	0.344402	0.931425
C	-3.102663	-0.464152	-0.291033
C	-1.397599	1.437158	-0.293148
H	-2.235464	0.472765	-2.075634
H	-0.408317	1.682594	-0.692619
O	-2.284033	2.519668	-0.405454
O	-0.841519	-0.709129	-1.165844
O	-4.232904	-0.662679	-1.122353
H	-3.963859	1.293351	0.608148
C	-4.589899	-0.462691	1.674313
H	-5.016069	0.092565	2.511382
H	-4.171091	-1.410548	2.043053
O	-5.656918	-0.704303	0.750332
O	-2.403244	0.618525	1.789319
C	-1.234527	0.936255	1.146177
H	-0.605020	1.543842	1.787986
C	-1.702534	3.777045	-0.031641
H	-0.788806	3.963366	-0.608010
H	-1.471362	3.805079	1.039133
H	-2.450974	4.535696	-0.263200
C	-5.244006	-1.395504	-0.416243
H	-4.806315	-2.362487	-0.117951
H	-2.738343	-1.445042	0.052255
C	-6.433376	-1.561323	-1.329228
H	-6.832498	-0.580058	-1.603264
H	-7.212347	-2.135969	-0.820621

H	-6.134382	-2.094020	-2.236196
C	-0.149754	-0.947639	-0.080875
O	-0.379721	-0.330785	1.036542
C	0.856540	-1.963824	-0.124328
C	2.858949	-3.908949	-0.209195
C	1.709650	-2.153450	0.980934
C	1.021265	-2.761449	-1.281192
C	2.007786	-3.722962	-1.320789
C	2.703404	-3.116637	0.945503
H	1.599349	-1.528231	1.859045
H	0.371067	-2.615665	-2.136146
H	2.151303	-4.345568	-2.197249
H	3.356550	-3.239261	1.799722
O	3.791427	-4.865730	-0.345870
O	1.523942	2.274788	1.338657
S	2.431064	1.697187	0.315671
O	3.607370	0.973315	0.843711
O	1.730554	1.029080	-0.812369
C	3.170574	3.216957	-0.498397
F	2.204712	3.988210	-1.039163
F	3.845295	3.963919	0.396650
F	4.024510	2.871884	-1.481411
C	4.707254	-5.113611	0.733036
H	5.303112	-4.220344	0.944032
H	5.353209	-5.917608	0.382900

P-GluPMBz
(3-*O*-(4-methoxybenzoyl)4,6-*O*-ethylidene-2-*O*-methyl-D-glucopyranose)
E = -2148.269106
H = -2147.851122
qh-*G* = -2147.902150
N_{imag} = 0

C	2.340110	-1.487843	1.450476
C	3.696302	-1.310242	1.607430
C	4.019505	-1.569734	-0.804536
H	2.243301	-1.913571	-1.945213
H	1.683314	-1.437651	2.310679
H	4.129296	-1.123385	2.584022
H	4.660909	-1.591366	-1.676048
O	5.851005	-1.142511	0.735566
C	-1.821516	-1.822750	0.864062
C	-2.925272	-0.433566	-0.951151
C	-2.202297	-0.413251	0.393564
C	-2.118705	-2.897903	-0.196399
H	-2.233933	-2.051818	1.844305
H	-1.569725	-3.816185	0.052567
O	-3.483026	-3.163005	-0.376018
O	-0.366841	-1.859923	1.057840
O	-3.042492	0.242539	1.332388
H	-3.902235	-0.906680	-0.843166
C	-3.104764	1.015378	-1.386104
H	-3.689681	1.098893	-2.303789
H	-2.129976	1.500329	-1.521001
O	-3.861410	1.663457	-0.353799
O	-2.168241	-1.176817	-1.936888
C	-1.558520	-2.317813	-1.492462
H	-1.477756	-3.024461	-2.314499
C	-4.036830	-4.063028	0.592311
H	-4.014174	-3.631544	1.599480
H	-3.492963	-5.015729	0.584998
H	-5.072377	-4.226303	0.292646

C	-3.236966	1.604671	0.921387
H	-2.246270	2.073834	0.857011
H	-1.277599	0.163179	0.279224
C	-4.131571	2.282630	1.928673
H	-5.103007	1.779756	1.967727
H	-4.280584	3.328212	1.644887
H	-3.667509	2.247956	2.918329
C	0.390392	-1.867875	-0.009883
O	-0.093676	-2.025479	-1.205557
C	1.800127	-1.716487	0.162067
C	4.547982	-1.341727	0.482306
C	2.657085	-1.753634	-0.956525
O	2.557496	1.877154	0.098672
S	1.080329	1.820241	0.083454
O	0.482984	0.984707	-0.989813
O	0.427676	1.656210	1.405781
C	0.590540	3.561340	-0.422794
F	1.100888	3.875893	-1.629930
F	-0.752246	3.687258	-0.498599
F	1.038676	4.468595	0.467069
C	6.785824	-1.127264	-0.355017
H	6.794473	-2.094319	-0.867582
H	7.756906	-0.940066	0.101295

P$_{bare}$-GluPMBz
(3-*O*-(4-methoxybenzoyl)4,6-*O*-ethylidene-2-*O*-methyl-D-glucopyranose)
E = -1186.655592
H = -1186.270977
qh-*G* = -1186.312290
N$_{imag}$ = 0

C	-1.350584	0.641934	-1.017584
C	-2.685159	0.016772	1.045731
C	-1.946084	-0.506169	-0.184426
C	-1.479395	2.006215	-0.311381
H	-1.730709	0.641415	-2.036676
H	-0.785972	2.718634	-0.778027
O	-2.781390	2.517542	-0.278833
O	0.091372	0.414121	-1.158916
O	-2.843054	-1.287797	-0.950687
H	-3.555108	0.596311	0.735813
C	-3.143321	-1.186071	1.859358
H	-3.760238	-0.893565	2.710289
H	-2.283491	-1.770876	2.216755
O	-3.970521	-1.974161	0.996909
O	-1.820248	0.871532	1.830868
C	-1.029051	1.732565	1.121091
H	-0.824884	2.615065	1.721601
C	-3.178006	3.200957	-1.476672
H	-3.234265	2.513599	-2.328155
H	-2.479687	4.016260	-1.700631
H	-4.168915	3.607469	-1.273849
C	-3.307562	-2.406869	-0.177899
H	-2.423351	-2.998528	0.110636
H	-1.116292	-1.145539	0.155832
C	-4.276687	-3.195396	-1.022477
H	-5.134160	-2.570159	-1.288881
H	-4.628584	-4.066694	-0.463505
H	-3.781692	-3.536323	-1.935947
C	0.839559	0.609064	-0.101071
O	0.368170	1.124776	0.996928
C	2.214795	0.231913	-0.163098

C	4.908969	-0.505097	-0.276091
C	3.076010	0.509499	0.921674
C	2.726013	-0.423264	-1.312524
C	4.052903	-0.784524	-1.366361
C	4.409694	0.146916	0.871741
H	2.689708	1.013113	1.800426
H	2.070147	-0.642626	-2.147046
H	4.463841	-1.288097	-2.234478
H	5.054440	0.369629	1.712119
O	6.179377	-0.897634	-0.424299
C	7.123393	-0.666302	0.636588
H	6.813123	-1.191187	1.544934
H	8.066006	-1.073253	0.274094

R_α-ManPMBz
(3-*O*-(4-methoxybenzoyl)4,6-*O*-ethylidene-2-*O*-methyl-D-mannopyranose)
E = -2148.286242
H = -2147.869584
qh-*G* = -2147.919733
N_{imag} = 0

C	-0.173934	0.159982	0.086445
C	1.892401	1.477555	0.458868
C	0.361101	1.523349	0.469720
C	0.382109	-0.880349	1.078688
H	0.122644	-0.098345	-0.933073
O	-1.605306	0.196181	0.167564
O	-0.054843	2.518881	-0.457344
H	2.234568	1.220689	-0.549472
C	2.427041	2.854623	0.829694
H	3.511993	2.908262	0.724879
H	2.150348	3.104033	1.864561
O	1.872061	3.803578	-0.083629
C	1.912492	-0.770894	1.185890
C	0.451072	3.798820	-0.085971
H	0.099951	4.021167	0.935496
H	0.007704	1.782862	1.477659
C	-0.032500	4.815552	-1.091017
H	0.334320	4.556377	-2.089019
H	0.335895	5.809154	-0.820805
H	-1.125861	4.833465	-1.104502
C	-2.298489	-0.654886	-0.632752
H	2.295881	-1.400912	1.985319
O	2.387255	0.506173	1.396256
O	2.407412	-1.304147	-0.113594
S	3.858766	-1.996774	-0.213973
O	3.786592	-2.975597	-1.285859
O	4.373448	-2.322048	1.109892
C	4.889319	-0.566502	-0.885007
F	6.091675	-1.042048	-1.215404
F	4.295345	-0.055408	-1.965776
F	5.022636	0.382786	0.041013
O	-1.748696	-1.435978	-1.395262
C	-3.756942	-0.526267	-0.452473
C	-6.542132	-0.363626	-0.162742
C	-4.326308	0.393742	0.449089
C	-4.601531	-1.357072	-1.200701
C	-5.985444	-1.284655	-1.065135
C	-5.702234	0.474841	0.592771
H	-3.684687	1.042629	1.033965
H	-4.164080	-2.066688	-1.895156
H	-6.613477	-1.939266	-1.656680

H	-6.152452	1.180174	1.284022
O	-7.873144	-0.210836	0.048265
O	-0.104635	-0.640939	2.388057
C	-1.137171	-1.531375	2.818145
H	-2.046556	-1.402844	2.221328
H	-0.800460	-2.574471	2.760645
H	-1.346237	-1.274475	3.858260
H	0.129342	-1.890890	0.742522
C	-8.784275	-1.033524	-0.687648
H	-9.779245	-0.735288	-0.357589
H	-8.683803	-0.859914	-1.764641

TS_I-ManPMBz
(3-*O*-(4-methoxybenzoyl)4,6-*O*-ethylidene-2-*O*-methyl-D-mannopyranose)
E = -2148.270196
H = -2147.854004
qh-*G* = -2147.904324
N_{imag} = 1

C	-0.202006	0.142161	-0.172644
C	1.863878	1.275295	0.647481
C	0.338184	1.417482	0.470004
C	0.285201	-1.069670	0.652710
H	0.176893	0.073555	-1.197712
O	-1.622912	-0.099639	-0.217569
O	0.194979	2.563168	-0.360999
H	2.309434	1.201698	-0.350274
C	2.396144	2.529116	1.334794
H	3.485516	2.507109	1.401424
H	1.974704	2.609742	2.348002
O	2.047759	3.668390	0.551341
C	1.796319	-1.054734	0.909803
C	0.648499	3.736940	0.294104
H	0.121825	3.820149	1.259430
H	-0.148163	1.582906	1.440531
C	0.382481	4.922060	-0.601988
H	0.909312	4.796028	-1.553074
H	0.730876	5.839302	-0.118743
H	-0.690484	5.005798	-0.795034
C	-2.618582	0.799546	-0.393354
H	2.095094	-1.846742	1.593313
O	2.255423	0.135267	1.423384
O	2.411072	-1.336708	-0.417446
S	3.851796	-2.052633	-0.517314
O	3.867774	-2.798688	-1.764701
O	4.223826	-2.652242	0.757726
C	4.972163	-0.559365	-0.788052
F	6.196473	-1.006827	-1.073680
F	4.509640	0.167533	-1.807387
F	5.015121	0.191668	0.312025
O	-2.462854	1.996409	-0.545275
C	-3.941886	0.126236	-0.365896
C	-6.496876	-1.038477	-0.317648
C	-4.092155	-1.256278	-0.148625
C	-5.087978	0.908113	-0.557521
C	-6.360732	0.341303	-0.536453
C	-5.352885	-1.833010	-0.124211
H	-3.215592	-1.874842	0.002532
H	-4.975985	1.974274	-0.724670
H	-7.227365	0.972801	-0.688698
H	-5.477885	-2.898121	0.043973
O	-7.686213	-1.692757	-0.276062

O	-0.343039	-1.017736	1.920062
C	-0.693518	-2.293433	2.455043
H	-1.392242	-2.813393	1.787124
H	0.191915	-2.922330	2.616776
H	-1.176871	-2.102011	3.414451
H	0.017626	-1.987852	0.118086
C	-8.888368	-0.940284	-0.463761
H	-8.999172	-0.179758	0.317279
H	-9.700830	-1.663285	-0.391330

RC_{I}-ManPMBz
(3-*O*-(4-methoxybenzoyl)4,6-*O*-ethylidene-2-*O*-methyl-D-mannopyranose)
E = -2148.279369
H = -2147.862055
qh-*G* = -2147.913346
N_{imag} = 0

C	-0.171857	0.099647	-0.201629
C	1.840927	1.392621	0.397209
C	0.310652	1.438232	0.329955
C	0.324336	-1.023878	0.722653
H	0.264805	-0.051612	-1.193079
O	-1.578057	-0.067926	-0.439055
O	-0.057771	2.493886	-0.549238
H	2.249635	1.200197	-0.601031
C	2.342702	2.742871	0.894367
H	3.431363	2.809273	0.854838
H	2.006651	2.912152	1.927894
O	1.831116	3.755614	0.025721
O	2.278932	0.364082	1.302772
C	1.842530	-0.900438	0.975214
H	2.172855	-1.571751	1.765301
C	0.410873	3.743984	-0.054981
H	0.002337	3.885527	0.958897
H	-0.092518	1.624258	1.328610
C	-0.026754	4.832245	-1.004521
H	0.395224	4.652416	-1.998264
H	0.317025	5.804313	-0.639872
H	-1.117999	4.845015	-1.075092
C	-2.555767	0.475270	0.332824
O	-2.370526	1.297645	1.212408
O	-0.345692	-0.923985	1.960621
C	-0.562629	-2.174944	2.614338
H	-1.100774	-1.949411	3.536266
H	0.383313	-2.673681	2.862043
H	-1.169912	-2.837591	1.984252
H	0.124709	-1.992198	0.248459
C	-3.888501	-0.044948	-0.053890
C	-6.459046	-0.975289	-0.694016
C	-4.064865	-1.027784	-1.046280
C	-5.016445	0.459622	0.606264
C	-6.296620	0.005655	0.297159
C	-5.333716	-1.488161	-1.363430
H	-3.202807	-1.428311	-1.566793
H	-4.883661	1.217019	1.371659
H	-7.148919	0.415210	0.825286
H	-5.479487	-2.246048	-2.126728
O	-7.657637	-1.488266	-1.072639
O	2.461436	-1.354470	-0.302704
S	3.929700	-2.018982	-0.311528
O	4.335554	-2.401480	1.034742
O	3.967615	-2.945685	-1.430883

C	4.985889	-0.541836	-0.822716
F	4.479937	0.008017	-1.928976
F	5.015860	0.365654	0.152568
F	6.222564	-0.981203	-1.064459
C	-8.842024	-1.008546	-0.429182
H	-8.814737	-1.218662	0.645843
H	-9.666458	-1.552690	-0.889853

TS_{II}-ManPMBz
(3-*O*-(4-methoxybenzoyl)4,6-*O*-ethylidene-2-*O*-methyl-D-mannopyranose)
E = -2148.268623
H = -2147.851891
qh-*G* = -2147.901943
N_{imag} = 1

C	-0.387610	0.488773	-0.873528
C	1.656283	1.495520	0.115334
C	0.145469	1.632874	-0.022114
C	0.341274	-0.892668	-0.668105
H	-0.208458	0.781109	-1.910258
O	-1.811747	0.347448	-0.835254
O	-0.162767	2.867327	-0.669798
H	2.139414	1.405300	-0.865346
C	2.205653	2.711867	0.850830
H	3.296590	2.726286	0.854996
H	1.840095	2.714360	1.888318
O	1.773794	3.886307	0.162475
O	1.903091	0.319344	0.900708
C	1.586814	-0.856278	0.272569
H	1.504751	-1.623959	1.039175
C	0.358364	3.970418	0.063897
H	-0.064221	3.938578	1.081727
H	-0.292829	1.617007	0.979436
C	-0.000050	5.243785	-0.662904
H	0.437695	5.236721	-1.665990
H	0.382571	6.106913	-0.110990
H	-1.087054	5.329705	-0.746890
C	-2.452752	0.096963	0.337691
O	-1.902577	0.111940	1.426681
O	-0.572350	-1.851660	-0.190376
C	-0.269785	-3.190691	-0.581311
H	-1.094843	-3.807060	-0.220976
H	0.666343	-3.544987	-0.130555
H	-0.197039	-3.268265	-1.674267
H	0.701705	-1.204316	-1.654856
C	-3.892145	-0.165706	0.127350
C	-6.633108	-0.692730	-0.165064
C	-4.476108	-0.213028	-1.152779
C	-4.700873	-0.385852	1.249890
C	-6.062335	-0.648097	1.116972
C	-5.830074	-0.472732	-1.298568
H	-3.862783	-0.046869	-2.030762
H	-4.252309	-0.351727	2.237185
H	-6.661831	-0.814754	2.003421
H	-6.289961	-0.512596	-2.280970
O	-7.945115	-0.941148	-0.408897
O	2.721646	-1.256889	-0.634632
S	4.036257	-1.956742	-0.031133
O	3.794952	-2.451520	1.318792
O	4.585554	-2.801331	-1.080638
C	5.215439	-0.490725	0.109182
F	5.245548	0.174397	-1.048213

F	4.832471	0.330079	1.085893
F	6.432789	-0.969239	0.381407
C	-8.815116	-1.181293	0.701383
H	-8.496933	-2.067274	1.262060
H	-9.800721	-1.353450	0.269034

RC$_{II}$-ManPMBz
(3-*O*-(4-methoxybenzoyl)4,6-*O*-ethylidene-2-*O*-methyl-D-mannopyranose)
E = -2148.277251
H = -2147.859796
qh-*G* = -2147.910740
N_{imag} = 0

C	0.155735	0.876993	0.872149
C	-1.651568	2.007694	-0.443893
C	-0.222024	2.128060	0.082713
C	-0.750297	-0.345119	0.500599
H	-0.005447	1.094834	1.929004
O	1.558015	0.569578	0.831238
O	-0.129434	3.275308	0.924991
H	-2.359060	1.827858	0.371319
C	-2.023446	3.299165	-1.161951
H	-3.076523	3.314283	-1.448104
H	-1.401566	3.421650	-2.060917
O	-1.819313	4.386350	-0.257410
O	-1.696368	0.929493	-1.393299
C	-1.292577	-0.306683	-0.934323
H	-0.594292	-0.758511	-1.632881
C	-0.482305	4.459865	0.213781
H	0.190383	4.551071	-0.655408
H	0.449242	2.244083	-0.776105
C	-0.364034	5.635710	1.152653
H	-1.045129	5.506579	1.999550
H	-0.619711	6.558877	0.625065
H	0.661340	5.712210	1.525178
C	2.124626	0.164941	-0.331886
O	1.519072	0.152632	-1.394227
O	-0.096390	-1.582070	0.636750
C	0.107739	-1.988006	1.991326
H	0.514049	-2.999666	1.948880
H	-0.842505	-1.995633	2.540624
H	0.822802	-1.329813	2.498299
H	-1.605106	-0.297433	1.185386
C	3.533848	-0.233135	-0.159144
C	6.215655	-1.022309	0.070858
C	4.166766	-0.245853	1.098727
C	4.262908	-0.621033	-1.291181
C	5.594810	-1.013767	-1.188876
C	5.491648	-0.635670	1.213489
H	3.614088	0.049528	1.983078
H	3.775576	-0.613462	-2.260506
H	6.133692	-1.307551	-2.081145
H	5.989606	-0.650621	2.177781
O	7.504439	-1.387097	0.284031
O	-2.431563	-1.283007	-1.065769
S	-3.901430	-1.000871	-0.499856
O	-3.910266	0.032168	0.533332
O	-4.856797	-0.974782	-1.597464
C	-4.126813	-2.648922	0.379586
F	-3.986740	-3.658248	-0.480028
F	-3.223018	-2.763948	1.357042
F	-5.354727	-2.677367	0.906372

C	8.298033	-1.791747	-0.836294
H	8.390767	-0.977086	-1.562887
H	9.278760	-2.034222	-0.427376

TS$_{III}$-ManPMBz
(3-*O*-(4-methoxybenzoyl)4,6-*O*-ethylidene-2-*O*-methyl-D-mannopyranose)
E = -2148.267193
H = -2147.851202
qh-*G* = -2147.901904
N_{imag} = 1

C	0.194370	0.820525	0.875286
C	-1.499148	1.988382	-0.581834
C	-0.146514	2.107597	0.121670
C	-0.687164	-0.385502	0.388064
H	-0.019806	0.980214	1.932394
O	1.592951	0.514274	0.871184
O	-0.209126	3.206082	1.022710
H	-2.305266	1.746765	0.113792
C	-1.802774	3.291180	-1.307551
H	-2.807164	3.296053	-1.733413
H	-1.066656	3.466298	-2.105362
O	-1.753724	4.334949	-0.333948
O	-1.380299	0.908975	-1.554687
C	-1.065578	-0.263553	-1.072876
H	-0.617862	-0.938417	-1.788461
C	-0.498596	4.419307	0.324573
H	0.286550	4.570613	-0.434074
H	0.619403	2.296877	-0.640367
C	-0.553484	5.545492	1.326888
H	-1.343572	5.354192	2.059387
H	-0.761303	6.488224	0.813357
H	0.405628	5.627733	1.845646
C	2.171563	0.123228	-0.292350
O	1.558032	0.111922	-1.352168
O	-0.073536	-1.632597	0.553471
C	0.016718	-2.061606	1.915389
H	0.387061	-3.086987	1.885728
H	-0.969845	-2.038886	2.393513
H	0.718614	-1.436545	2.479202
H	-1.610648	-0.321232	0.976808
C	3.581806	-0.258472	-0.125015
C	6.272187	-1.015065	0.098234
C	4.220101	-0.253799	1.130698
C	4.309375	-0.646937	-1.258458
C	5.645552	-1.023578	-1.159059
C	5.549324	-0.627425	1.241789
H	3.668584	0.042528	2.015481
H	3.817664	-0.652618	-2.225541
H	6.183821	-1.318566	-2.051199
H	6.052232	-0.629171	2.203519
O	7.564857	-1.362971	0.308157
O	-2.613187	-1.330130	-1.190789
S	-3.969046	-0.980223	-0.562304
O	-3.873466	0.111156	0.422418
O	-5.054009	-0.941255	-1.545707
C	-4.279649	-2.524374	0.457352
F	-4.306279	-3.610678	-0.324768
F	-3.309676	-2.674575	1.373741
F	-5.456120	-2.416172	1.091014
C	8.359436	-1.768275	-0.811851
H	9.344050	-1.995922	-0.403943

H	8.440267	-0.958625	-1.545277

PC-ManPMBz
(3-*O*-(4-methoxybenzoyl)4,6-*O*-ethylidene-2-*O*-methyl-D-mannopyranose)
E = -2148.265338
H = -2147.848957
qh-*G* = -2147.900827
N_{imag} = 0

C	0.274944	0.795745	0.940633
C	-1.264970	1.960054	-0.700389
C	-0.014310	2.090216	0.170617
C	-0.552348	-0.394855	0.353939
H	0.019094	0.933283	1.990520
O	1.672157	0.478058	0.957514
O	-0.223751	3.170983	1.064542
H	-2.150824	1.701792	-0.115497
C	-1.483703	3.257213	-1.464090
H	-2.419372	3.248426	-2.024370
H	-0.646042	3.450879	-2.149201
O	-1.589102	4.289247	-0.483114
O	-1.043253	0.853614	-1.645334
C	-0.650757	-0.246033	-1.126462
H	-0.500105	-1.072569	-1.806794
C	-0.441203	4.391047	0.345136
H	0.439536	4.567959	-0.293034
H	0.839990	2.312936	-0.482686
C	-0.661846	5.498571	1.344592
H	-1.542694	5.279134	1.955393
H	-0.816348	6.444696	0.818824
H	0.212873	5.591945	1.993993
C	2.228063	0.034471	-0.188773
O	1.581200	-0.036278	-1.241082
O	-0.008624	-1.651870	0.627580
C	-0.189404	-2.080403	1.982520
H	0.170756	-3.108717	2.022771
H	-1.249444	-2.047786	2.258436
H	0.396926	-1.462152	2.672296
H	-1.576659	-0.294586	0.746908
C	3.638880	-0.324997	-0.062462
C	6.341194	-1.042764	0.100686
C	4.337151	-0.189779	1.154990
C	4.312309	-0.822704	-1.188550
C	5.653474	-1.181978	-1.117583
C	5.672173	-0.543857	1.235397
H	3.827349	0.193007	2.031607
H	3.774349	-0.928922	-2.124465
H	6.149596	-1.565091	-2.000499
H	6.222906	-0.445785	2.165178
O	7.642703	-1.360973	0.280100
O	-2.852247	-1.471873	-1.310952
S	-4.042342	-0.921546	-0.602718
O	-3.700600	0.114593	0.408101
O	-5.206268	-0.623025	-1.458075
C	-4.609822	-2.368827	0.445902
F	-4.941517	-3.418899	-0.326146
F	-3.631623	-2.760217	1.287139
F	-5.683687	-2.031721	1.182800
C	8.390127	-1.873000	-0.830189
H	8.428205	-1.140250	-1.643235
H	9.394260	-2.051954	-0.446843

P-ManPMBz
(3-*O*-(4-methoxybenzoyl)4,6-*O*-ethylidene-2-*O*-methyl-D-mannopyranose)
E = -2148.273963
H = -2147.855966
qh-*G* = -2147.906794
N_{imag} = 0

C	2.531521	0.993295	-1.368861
C	3.832046	0.573392	-1.541381
C	4.195501	0.676564	0.875796
H	2.512625	1.302308	2.039763
H	1.878351	1.100906	-2.226691
H	4.223801	0.348683	-2.527419
H	4.829248	0.547126	1.743501
O	5.919282	-0.022282	-0.690050
C	-1.480485	2.124685	-0.761250
C	-2.892149	1.000979	1.024148
C	-2.197364	0.838510	-0.326560
C	-1.561035	3.197929	0.334455
H	-1.816962	2.460687	-1.739708
O	-0.685312	4.277406	0.142111
H	-2.598449	3.538251	0.444690
O	-0.062331	1.823250	-0.946666
O	-3.173777	0.440401	-1.276714
H	-3.729313	1.702070	0.937779
C	-3.438749	-0.366033	1.418528
H	-4.028725	-0.322747	2.335604
H	-2.617114	-1.082982	1.539551
O	-4.328100	-0.775086	0.372009
O	-1.973594	1.494032	2.023734
C	-1.119036	2.482312	1.603175
H	-0.886057	3.145956	2.431964
C	-1.051184	5.154772	-0.928841
H	-0.940158	4.663930	-1.902509
H	-2.083784	5.504135	-0.805209
H	-0.366728	6.001696	-0.872432
C	-3.702994	-0.841534	-0.902910
H	-2.860259	-1.543697	-0.853796
H	-1.440667	0.052371	-0.231455
C	-4.733850	-1.247745	-1.925704
H	-5.548273	-0.516896	-1.950289
H	-5.141091	-2.229553	-1.668606
H	-4.270780	-1.305394	-2.914695
C	0.683357	1.673216	0.115545
O	0.238478	1.879654	1.317950
C	2.042456	1.268504	-0.070058
C	4.674243	0.404286	-0.421568
C	2.889901	1.104565	1.043278
O	1.885553	-2.482614	-0.292321
S	0.465620	-2.081251	-0.197912
O	0.141551	-1.130508	0.896536
O	-0.203233	-1.766603	-1.484136
C	-0.389729	-3.659052	0.355064
F	0.094295	-4.082893	1.539174
F	-1.719136	-3.466670	0.499011
F	-0.212446	-4.646699	-0.544110
C	6.832160	-0.253785	0.394214
H	6.439906	-1.020693	1.069103
H	7.751218	-0.604594	-0.073356

P_{bare}-ManPMBz
(3-*O*-(4-methoxybenzoyl)4,6-*O*-ethylidene-2-*O*-methyl-D-mannopyranose)

E = -1186.660948
H = -1186.276387
qh-*G* = -1186.317496
N_{imag} = 0

C	-1.447572	0.782695	-0.880088
C	-2.921166	0.087472	1.063601
C	-2.217502	-0.371417	-0.212844
C	-1.467562	2.043340	0.003999
H	-1.789509	0.947740	-1.899422
O	-0.045057	0.401359	-1.018198
O	-3.184705	-0.915205	-1.089852
H	-3.703647	0.812656	0.819106
C	-3.577475	-1.130316	1.701743
H	-4.179696	-0.861391	2.570661
H	-2.819560	-1.869686	1.998306
O	-4.470692	-1.675995	0.727054
O	-1.978592	0.698656	1.969478
C	-1.070502	1.543646	1.384772
H	-0.784875	2.325043	2.083886
C	-3.828240	-2.047125	-0.479934
H	-3.045659	-2.788828	-0.252690
H	-1.489167	-1.152327	0.054163
C	-4.862786	-2.576587	-1.440727
H	-5.614076	-1.807003	-1.641914
H	-5.353919	-3.452185	-1.007707
H	-4.383697	-2.866801	-2.379775
C	0.704704	0.428417	0.054082
O	0.247710	0.803233	1.213131
O	-0.523768	3.010020	-0.367481
C	-0.817694	3.694307	-1.592813
H	-0.097478	4.509747	-1.661401
H	-1.836703	4.099498	-1.572705
H	-0.697457	3.030321	-2.456399
H	-2.480858	2.461543	0.036766
C	2.062824	0.000488	-0.056317
C	4.728027	-0.816592	-0.262963
C	2.557665	-0.508356	-1.283552
C	2.924269	0.090553	1.058628
C	4.244166	-0.312430	0.962619
C	3.870539	-0.910496	-1.383181
H	1.901419	-0.581995	-2.143030
H	2.550188	0.483347	1.997121
H	4.889874	-0.233062	1.827532
H	4.269414	-1.303309	-2.312055
O	5.985800	-1.230287	-0.461089
C	6.932355	-1.166149	0.620236
H	7.862963	-1.554294	0.209225
H	6.602690	-1.790925	1.455598

Supplementary Organic Synthesis Methods

General experimental procedures

All chemicals (Acros, Fluka, Merck, and Sigma-Aldrich) were used as received unless stated otherwise. Dichloromethane was stored over activated 4 Å molecular sieves (beads, 8-12 mesh, Sigma-Aldrich). Before use traces of water present in the donor, diphenyl sulfoxide (Ph_2SO) and tri-*tert*-butylpyrimidine (TTBP) were removed by co-evaporation with dry toluene. The acceptors were stored in stock solutions (DCM, 0.5 M) over activated 3 Å molecular sieves (rods, size 1/16 in., Sigma-Aldrich). Trifluoromethanesulfonic anhydride (Tf_2O) was distilled over P_2O_5 and stored at –20 °C under a nitrogen atmosphere. Overnight temperature control was achieved by an FT902 Immersion Cooler (Julabo). Column chromatography was performed on silica gel 60 Å (0.04 – 0.063 mm, Screening Devices B.V.). TLC-analysis was conducted on TLC Silica gel 60 (Kieselgel 60 F_{254}, Merck) with UV detection by (254 nm) and by spraying with 20% sulfuric acid in ethanol followed by charring at ± 150 °C or by spraying with a solution of $(NH_4)_6Mo_7O_{24}\cdot H_2O$ (25 g/l) and $(NH_4)_4Ce(SO_4)_4\cdot 2H_2O$ (10 g/l) in 10% sulfuric acid in water followed by charring at ± 250 °C. High-resolution mass spectra were recorded on a Thermo Finnigan LTQ Orbitrap mass spectrometer equipped with an electrospray ion source in positive mode (source voltage 3.5 kV, sheath gas flow 10, capillary temperature 275 °C) with resolution R=60.000 at m/z=400 (mass range = 150-4000). ^{1}H and $^{13}C\{^{1}H\}$ NMR spectra were recorded on a Bruker AV-400 NMR instrument (400 and 101 MHz respectively), a Bruker AV-500 NMR instrument (500 and 126 MHz respectively), or a Bruker AV-850 NMR instrument (850 and 214 MHz respectively). For samples measured in $CDCl_3$ chemical shifts (δ) are given in ppm relative to tetramethylsilane as an internal standard or the residual signal of the deuterated solvent. Coupling constants (*J*) are given in Hz. To get better resolution of signals with small coupling constants or overlapping signals a gaussian window function (LB ± -1 and GB ± 0.5) was used on the ^{1}H NMR spectrum. All given ^{13}C APT spectra are proton decoupled. NMR peak assignment was made using COSY, HSQC. If necessary additional, HMBC and HMBC-GATED experiments were used to elucidate the structure further. The anomeric product ratios were based on the integration of ^{1}H NMR.

Preparation of the donors 1-4

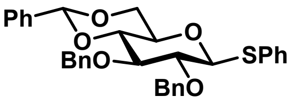

Phenyl 2,3-di-*O*-benzyl-4,6-*O*-benzylidene-1-thio-β-ᴅ-glucopyranoside (1). The title compound was prepared according to literature procedure.[16]

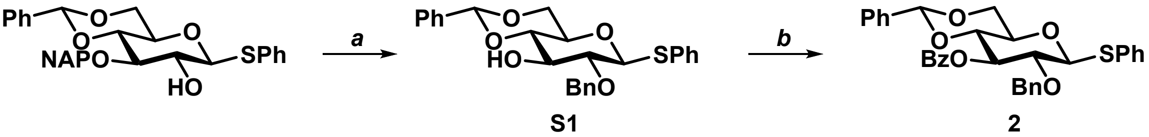

Supplementary scheme S1. Glucoside donor **2** synthesis. *Reagents and conditions:* a) 1. BnBr, NaH, DMF, 0 °C to RT; glucopyranoside; 2. DDQ, DCM:H_2O, **S1**: 82%; b) benzoyl chloride, pyridine, RT, **2**: quant.

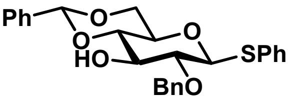

Phenyl 2-*O*-benzyl-4,6-*O*-benzylidene-1-thio-β-ᴅ-glucopyranoside (S1). The synthesis of **S1** started from phenyl 3-*O*-(2-naphthyl)methyl-4,6-*O*-benzylidene-1-thio-β-ᴅ-glucopyranoside[17] (1.34 g, 2.67 mmol), which was dissolved in dry DMF (10 ml, 0.25M) and cooled to 0 °C. Under inert atmosphere, NaH (60 Wt % dispersion in mineral oil, 0.16 g, 4.01 mmol, 1.5 eq.) was added portion wise and the reaction mixture was stirred for 10 minutes. Benzyl bromide (0.40 ml, 3.34 mmol, 1.25 eq.) was added and the reaction was stirred for 4 h while allowing to warm up to RT. Subsequently, the reaction was cooled to 0 °C and quenched with H_2O. The reaction mixture was diluted with H_2O and extracted with DCM. The organic layers were combined, washed with brine, dried over $MgSO_4$ and concentrated *in vacuo*. The crude reaction mixture was dissolved in DCM:H_2O (9:1, v:v, 15 ml, 0.2M), after which 2,3-Dichloro-5,6-dicyano-1,4-benzoquinone (0.910 g, 4,01 mmol, 1.5 eq.) was added. The reaction was protected from light and stirred for 1.5 h. The reaction was quenched by addition of sat. aq. $Na_2S_3O_3$ and filtered. The bi-phasic mixture was extracted with DCM. The organic layers were combined and washed with sat. aq. $NaHCO_3$, dried over $MgSO_4$, filtered and concentrated *in vacuo*. Flash column chromatography (100:0 → 80:20, pentane:EtOAc v:v) yielded the title compound (0.99 g, 2.19 mmol, 82%) as a white solid. TLC: R_f 0.18, (Pentane:EtOAc, 90:10, v:v); ^{1}H NMR (300 MHz, $CDCl_3$, HH-COSY, HSQC) δ 7.58 – 7.25 (m, 15H, CH_{arom}), 5.51 (s, 1H, C*H*Ph), 4.93 (d, J = 10.9 Hz, 1H, CH*H* Bn), 4.85 – 4.63 (m, 2Hz, H-1, C*H*H Bn), 4.35 (dd, J = 10.5, 4.8 Hz, 1H, H-6), 3.89 (td, J = 8.7, 2.5 Hz, 1H, H-3), 3.76 (dd, J = 10.5, 9.6 Hz, 1H, H-6), 3.58 – 3.33 (m, 3H, H-2, H-4, H-5), 2.58 (d, J = 2.5 Hz, 1H, 3-OH).; $^{13}C\{^1H\}$ NMR (75 MHz, $CDCl_3$, HSQC) δ 138.1 ($C_{q\text{-}arom}$), 137.0, 133.2, 132.3, 129.4, 129.2, 128.7, 128.5, 128.4, 128.2, 128.0, 126.4 (CH_{arom}), 101.9 (CHPh), 88.1 (C-1), 80.8 (C-2), 80.4 (C-4), 75.6 (CH_2 Bn), 75.5 (C-3), 70.2 (C-5), 68.7 (C-6); HRMS (ESI): $[M+Na]^+$ calcd for $C_{26}H_{26}O_5SNa$ 473.1393, found 473.1391.

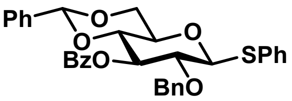

Phenyl 3-*O*-benzoyl-2-*O*-benzyl-4,6-*O*-benzylidene-1-thio-β-ᴅ-glucopyranoside (2). S1 (0.952 g, 2.11 mmol) was dissolved in pyridine (4 ml, 0.5 M), benzoyl chloride (0.445 g, 0.368 ml, 3.17 mmol, 1.5 eq.) was added and the reaction was stirred for 4 hours. It was diluted with ethyl acetate and sequentially washed with 1M HCl (aq.) and sat. aq. $NaHCO_3$. The organic layers were combined, dried over $MgSO_4$, filtered and concentrated *in vacuo*. Flash column chromatography (100:0 → 95:5, pentane:EtOAc v:v) yielded the title compound (1.26 g, quant) as a white solid. TLC: R_f 0.35, (Pentane:EtOAc, 90:10, v:v); ^{1}H NMR (400 MHz, $CDCl_3$, HH-COSY, HSQC, HMBC) δ 8.06 – 7.64

– 7.11 (m, 20H, CH_{arom}), 5.68 (dd, J = 9.8, 8.7 Hz, 1H, H-3), 5.49 (s, 1H C*H*Ph), 4.90 (d, J = 9.7 Hz, 1H, H-1), 4.85 (d, J = 10.5 Hz, 1H, CH*H* Bn), 4.61 (d, J = 10.5 Hz, 1H, C*H*H Bn), 4.40 (dd, J = 10.5, 4.9 Hz, 1H, H-6), 3.87 – 3.67 (m, 3H, H-2, H-4, H-6), 3.62 (td, J = 9.7, 5.0 Hz, 1H, H-5); $^{13}C\{^{1}H\}$ NMR (101 MHz, $CDCl_3$, HSQC, HMBC) δ 165.5 ($C_{q\text{-benzoyl}}$), 137.3, 136.9, 133.1 ($C_{q\text{-arom}}$), 129.9, 129.3, 129.1, 128.5, 128.4, 128.4, 128.3, 128.1, 128.0, 126.2 (CH^{arom}), 101.5 (CHPh), 88.7 (C-1), 79.3 (C-2), 78.8 (C-3), 75.5 (CH_2 Bn), 75.1 (C-3), 70.6 (C-5), 68.8 (C-6). HRMS (ESI): $[M+Na]^+$ calcd for $C_{33}H_{30}O_6SNa$ 577.1655, found 577. 1649.

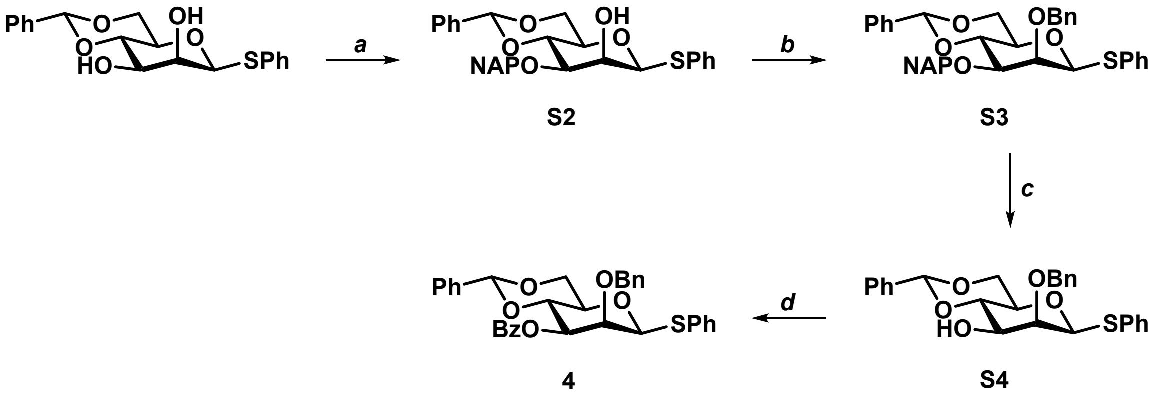

Supplementary scheme S2. Mannoside donor **4** synthesis. *Reagents and conditions:* a) 1. Dibutyltin(IV) oxide, toluene, reflux; 2. 2-(bromomethyl)naphthalene, CsF, DMF, **S2**: 49%; b) BnBr, NaH, DMF, 0 °C to RT, **S3**: quant.; c) DDQ, DCM:H_2O, **S4**: 87%; d) benzoyl chloride, pyridine, RT, **4**: 69%,.

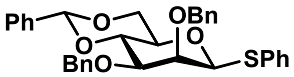

Phenyl 2,3-di-*O*-benzyl-4,6-*O*-benzylidene-1-thio-β-ᴅ-mannopyranoside (3). The title compound was prepared according to literature procedure.[16]

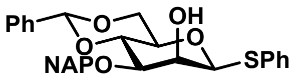

Phenyl 4,6-*O*-benzylidene-3-*O*-(2-naphthyl)methyl-1-thio-β-ᴅ-mannopyranoside (S2). The synthesis of **S2** started from phenyl 4,6-*O*-benzylidene-1-thio-β-ᴅ-mannopyranoside[16] (0.771 g, 2.14 mmol). Traces of solvent were removed from the glycoside by co-evaportation with toluene, and subsequently dissolved in toluene (8 ml, 0.3 M). Dibutyltin oxide (0.639 g, 2.57 mmol, 1.2 eq.) was added, and the resulting suspension was heated with an oil bath and refluxed for 1.5 h. The reaction mixture was concentrated *in vacuo*, coevaporated twice with toluene and dissolved in DMF (6 ml, 0.4 M). 2-(bromomethyl)naphthalene (0.709, 3.21 mmol, 1.5 eq.) and cesium fluoride (0.487, 3.21 mmol, 1.5 eq.) were added and it was stirred for 17 h. The reaction mixture was diluted with DCM and washed with sat. aq. $NaHCO_3$. The resulting tin paste was filtered from the solution using a Buchner funnel, and the filtrate was dried over $MgSO_4$. It was filtered off, and concentrated *in vacuo* to yield a white powder. Recrystalization from boiling EtOAc yielded the title compound (0.508 g, 1.044 mmol, 49%) as a white solid. TLC: R_f 0.25, (Pentane:EtOAc, 80:20, v:v); ^{1}H NMR (400 MHz, DMSO-d_6, HH-COSY, HSQC, HMBC) δ 7.94 – 7.18 (m, 17H, CH_{arom}), 5.71 (s, 1H, C*H*Ph), 5.68 (d, J = 6.1 Hz, 1H, 2-OH), 5.29 (d, J = 1.1 Hz, 1H, H-1), 4.88 (d, J = 12.9 Hz, 1H, C*H*H Nap), 4.77 (d, J = 13.0 Hz, 1H, C*H*H Nap), 4.31 (ddd, J = 6.0, 3.3, 1.3 Hz, 1H, H-2), 4.21 (dd, J = 10.2, 4.9 Hz, 1H, H-6), 4.08 (t, J = 9.6 Hz, 1H, H-4), 3.84 – 3.75 (m, 2H, H-3, H-6), 3.54 (td, J = 9.8, 4.9 Hz, 1H, H-5); ^{13}C{^{1}H} NMR (101 MHz, DMSO-d_6, HSQC, HMBC) δ 137.9, 136.5, 135.9, 132.8, 132.4 ($C_{q\text{-}arom}$), 129.1, 128.8, 128.5, 128.1, 127.6, 127.6, 126.2, 126.1, 126.1, 125.8, 125.7, 125.6 (CH_{arom}), 100.6 (CHPh), 87.2 (C-1), 78.0 (C-3), 77.2 (C-4), 70.4 (C-5), 70.1 (CH_2 Nap), 69.7 (C-2), 67.8 (C-6); HRMS (ESI): $[M+NH_4]^+$ calcd for $C_{30}H_{28}O_5SNH_4$ 518.1996, found 518.1998.

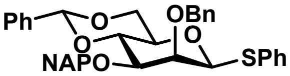

Pheny 2-*O*-benzyl-4,6-*O*-benzylidene-3-*O*-(2-naphthyl)methyl-1-thio-β-ᴅ-mannopyranoside (S3). **S2** (0.508 g, 1.04 mmol) was dissolved in dry DMF (5 ml, 0.2M) and cooled to 0 °C. Under inert atmosphere, NaH (60 Wt % dispersion in mineral oil, 0.063 g, 1.57 mmol, 1.5 eq.) was added portion wise and the reaction mixture was stirred for 10 minutes. Benzyl bromide (0.155 ml, 1.31 mmol, 1.25 eq.) was added and the reaction was stirred for 17 h while allowing to warm up to RT. Subsequently, the reaction was cooled to 0 °C and quenched with H_2O. The reaction mixture was diluted with H_2O and extracted with DCM. The organic layers were combined, washed with brine, dried over $MgSO_4$ and concentrated *in vacuo*. Flash column chromatography (100:0 → 90:10, pentane:EtOAc v:v) yielded the title compound (0.613 g, quant) as a white solid. TLC: R_f 0.39, (Pentane:EtOAc, 90:10, v:v); ^{1}H NMR (500 MHz, $CDCl_3$, HH-COSY, HSQC, HMBC) δ 7.90 – 7.12 (m, 29H, CH_{arom}), 5.66 (s, 1H, C*H*Ph), 5.15 (d, J = 11.1 Hz, 1H, C*H*H NAP), 5.02 (d, J = 12.6 Hz, 1H, C*H*H Bn), 4.93 – 4.87 (m, 2H, CH*H* Bn, CH*H* NAP), 4.84 (d, J = 1.3 Hz, 1H, H-1), 4.39 – 4.26 (m, 2H, H-4, H-6), 4.19 (dd, J = 3.1, 1.3 Hz, 1H, H-2), 3.95 (t, J = 10.3 Hz, 1H, H-6), 3.78 (dd, J = 9.8, 3.1 Hz, 1H, H-3), 3.42 (ddd, J = 10.1, 9.2, 4.9 Hz, 1H, H-5); ^{13}C{^{1}H} NMR (126 MHz, $CDCl_3$, HSQC, HMBC) δ 138.1, 137.7, 135.8, 135.1, 133.4, 133.1 ($C_{q\text{-}arom}$), 131.3, 129.1, 129.1, 128.8, 128.4, 128.4, 128.1, 127.9, 127.8, 127.6, 126.6, 126.3, 126.1, 125.8 (CH_{arom}), 101.7 (CHPh), 89.3 (C-1), 79.7 (C-3), 79.0 (C-2), 78.8 (C-3), 76.1 (CH_2 NAP), 73.3 (CH_2 Bn), 71.9 (C-5), 68.6 (C-6). HRMS (ESI): $[M+NH_4]^+$ calcd for $C_{37}H_{34}O_5SNH_4$ 608.2465, found 608.2463.

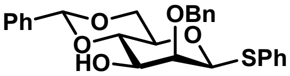

Phenyl 2-*O*-benzyl-4,6-*O*-benzylidene-1-thio-β-ᴅ-mannopyranoside (S4). **S3** (0.538 g, 0.910 mmol) was dissolved in DCM:H_2O (9:1, v:v, 5.5 ml, 0.2M), after which 2,3-Dichloro-5,6-dicyano-1,4-benzoquinone (0.310 g, 1,37 mmol, 1.5 eq.) was added. The reaction was protected from light and stirred for 1.5 h. The reaction was quenched by addition of sat. aq. $Na_2S_3O_3$ and filtered. The bi-phasic mixture was extracted with DCM. The organic layers were combined and washed with sat. aq. $NaHCO_3$, dried over $MgSO_4$, filtered and concentrated *in vacuo*. Flash column chromatography (90:10 → 80:20, pentane:EtOAc v:v) yielded the title compound (0.355 g, 0.788 mmol, 87%) as a white solid. TLC: R_f 0.40, (Pentane:EtOAc, 80:20, v:v); ^{1}H NMR (400 MHz, $CDCl_3$, HH-COSY, HSQC) δ 7.70 – 7.11 (m, 15H, CH_{arom}), 5.56 (s, 1H, C*H*Ph), 5.08 – 4.85 (m, 3H, H-1, C*H*H Bn, CH*H* Bn), 4.31 (dd, J = 10.5, 5.0 Hz, 1H, H-6), 4.15 (dd, J = 3.5, 1.3 Hz, 1H, H-2), 3.99 (t, J = 9.5 Hz, 1H, H-4), 3.94 – 3.81 (m, 2H, H-3, H-6), 3.41 (ddd, J = 10.2, 9.1, 5.0 Hz, 1H, H-5), 2.45 (d, J = 5.9 Hz, 1H, OH); ^{13}C{^{1}H} NMR (101 MHz, $CDCl_3$, HSQC, HMBC) δ 137.9, 137.3, 134.8 ($C_{q\text{-}arom}$), 131.3, 129.4, 129.2, 128.6, 128.6, 128.5, 128.2, 127.7, 126.4 (CH_{arom}), 102.2 (CHPh),

89.0 (C-1), 80.6 (C-2), 78.8 (C-4), 76.8 (CH_2 Bn), 73.0 (C-3), 71.3 (C-5), 68.5 (C-6); HRMS (ESI): $[M+Na]^+$ calcd for $C_{26}H_{26}O_5SNa$ 473.1393, found 473.1392.

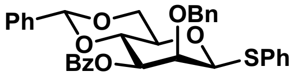

Phenyl 3-*O*-benzoyl-2-*O*-benzyl-4,6-*O*-benzylidene-1-thio-β-ᴅ-mannopyranoside (4). S4 (0.309 g, 0.686 mmol) was dissolved in pyridine (1.5 ml, 0.5 M), benzoyl chloride (0.145 g, 0.120 ml, 1.03 mmol, 1.5 eq.) was added and the reaction was stirred for 4 hours. It was diluted with ethyl acetate and sequentially washed with 1M HCl (aq.) and sat. aq. $NaHCO_3$. The organic layers were combined, dried over $MgSO_4$, filtered and concentrated *in vacuo*. Flash column chromatography (100:0 → 90:10, pentane:EtOAc v:v) yielded the title compound 0.261 g, 0.470 mmol, 69 %) as a white foam. TLC: R_f 0.40, (Pentane:EtOAc, 90:10, v:v); ^1H NMR (500 MHz, $CDCl_3$, HH-COSY, HSQC, HMBC) δ 8.19 – 7.14 (m, 20H), 5.62 (s, 1H, C*H*Ph), 5.36 (dd, *J* = 10.3, 3.3 Hz, 1H, H-3), 5.06 (d, *J* = 1.3 Hz, 1H, H-1), 4.81 (d, *J* = 11.0 Hz, 1H, C*H*H Bn), 4.77 (d, *J* = 10.9 Hz, 1H, CH*H* Bn), 4.49 (dd, *J* = 3.4, 1.3 Hz, 1H, H-2), 4.41 (dd, *J* = 10.3, 9.3 Hz, 1H, H-6), 4.35 (dd, *J* = 10.6, 4.9 Hz, 1H, H-4), 3.98 (t, *J* = 10.3 Hz, 1H, H-6), 3.60 (td, *J* = 9.9, 4.9 Hz, 1H, H-5); $^{13}C\{^1H\}$ NMR (126 MHz, $CDCl_3$, HSQC, HMBC) δ 166.2 ($C_{q\text{-benzoyl}}$), 137.5, 137.3, 134.5 ($C_{q\text{-arom}}$), 133.5, 131.7, 130.1 (CH_{arom}), 129.6 ($C_{q\text{-arom}}$), 129.2, 129.1, 128.6, 128.4, 128.4, 128.3, 128.0, 127.9, 126.2 (CH_{arom}), 101.8 (CHPh), 89.2 (C-1), 78.8 (C-2), 76.6 (CH_2 Bn), 75.7 (C-4), 74.4 (C-3), 71.9 (C-5), 68.6 (C-6). HRMS (ESI): $[M+Na]^+$ calcd for $C_{33}H_{30}O_6SNa$ 577.1655, found 577.1651.

Model glycosylations S5-S19

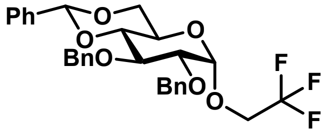

c

2,2,2-Trifluoroethyl 2,3-di-*O*-benzyl-4,6-*O*-benzylidene-ᴅ-glucopyranoside (S5). The title compound was prepared according the general glycosylation protocol, using **1** as the donor and 2,2,2-trifluoroethanol as the acceptor. Flash column chromatography (100:0 → 90:10, pentane:EtOAc v:v) yielded the title compound (39 mg, 0.074 mmol, 74%, α:β; 95:5) as a colorless oil. TLC: R_f 0.42, (90:10, pentane:EtOAc, v:v); ^1H NMR (850 MHz, $CDCl_3$, HH-COSY, HSQC, HMBC, HMBC-Gated) and $^{13}C\{^1H\}$ NMR (214 MHz, $CDCl_3$, HSQC, HMBC, HMBC-Gated) were in accordance to literature[16]; HRMS (ESI) M/Z: $[M + Na]^+$ Calcd for $C_{29}H_{29}F_3O_6Na$ 553.1808; Found 553.1804.

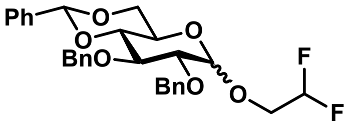

2,2-Difluoroethyl 2,3-di-*O*-benzyl-4,6-*O*-benzylidene-ᴅ-glucopyranoside (S6). The title compound was prepared according the general glycosylation protocol, using **1** as the donor and 2,2-difluoroethanol as the acceptor. Flash column chromatography (100:0 → 85:15, pentane:EtOAc v:v) yielded the title compound (40 mg, 0.095 mmol, 95%, α:β; 82:18) as a colorless oil. TLC: R_f 0.33, (90:10, pentane:EtOAc, v:v); ^1H NMR (850 MHz, $CDCl_3$, HH-COSY, HSQC, HMBC, HMBC-Gated) and $^{13}C\{^1H\}$ NMR (214 MHz, $CDCl_3$, HSQC, HMBC, HMBC-Gated) were in accordance to literature[16]; HRMS (ESI) M/Z: $[M + Na]^+$ Calcd for $C_{29}H_{30}F_2O_6Na$ 535.1903; Found 535.1902.

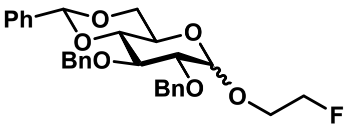

2-Fluoroethyl 2,3-di-*O*-benzyl-4,6-*O*-benzylidene-ᴅ-glucopyranoside (S7). The title compound was prepared according the general glycosylation protocol, using **1** as the donor and 2-fluoroethanol as the acceptor. Flash column chromatography (95:5 → 80:20, pentane:EtOAc v:v) yielded the title compound (32 mg, 0.065 mmol, 65%, α:β; 50:50) as a colorless oil. TLC: R_f 0.20, (90:10, pentane:EtOAc, v:v^1H NMR (850 MHz, $CDCl_3$, HH-COSY,

HSQC, HMBC, HMBC-Gated) and $^{13}C\{^{1}H\}$ NMR (214 MHz, $CDCl_3$, HSQC, HMBC, HMBC-Gated) were in accordance to literature[16]; HRMS (ESI) M/Z: $[M + Na]^+$ Calcd for $C_{29}H_{31}FO_6Na$ 517.1997; Found 517.1996.

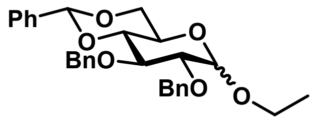

Ethyl 2,3-di-*O*-benzyl-4,6-*O*-benzylidene-D-glucopyranoside (S8). The title compound was prepared according the general glycosylation protocol, using **1** as the donor and ethanol as the acceptor. Flash column chromatography (100:0 → 90:10, pentane:EtOAc v:v) yielded the title compound (48 mg, 0.080 mmol, 80%, α:β; 30:70) as a colorless oil. TLC: R_f 0.51, (90:10, pentane:EtOAc, v:v); ^{1}H NMR (850 MHz, $CDCl_3$, HH-COSY, HSQC, HMBC, HMBC-Gated) and $^{13}C\{^{1}H\}$ NMR (214 MHz, $CDCl_3$, HSQC, HMBC, HMBC-Gated) were in accordance to literature[16]; HRMS (ESI) M/Z: $[M + Na]^+$ Calcd for $C_{29}H_{32}O_6Na$ 499.2091; Found 499.2090.

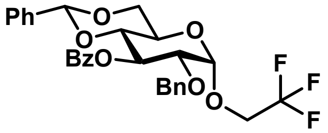

2,2,2-Trifluoroethyl 3-*O*-benzoyl-2-*O*-benzyl-4,6-*O*-benzylidene-D-glucopyranoside (S9). The title compound was prepared according the general glycosylation protocol, using **2** as the donor and 2,2,2-trifluoroethanol as the acceptor. Flash column chromatography (100:0 → 90:10, pentane:EtOAc v:v) yielded the title compound (46 mg, 0.084 mmol, 84%, α:β; >98:2) as a colorless oil. TLC: R_f 0.18, (90:10, pentane:EtOAc, v:v); ^{1}H NMR (850 MHz, $CDCl_3$, HH-COSY, HSQC, HMBC, HMBC-Gated) δ 8.11 – 7.19 (m, 15H, CH_{arom}), 5.84 (t, *J* = 9.7 Hz, 1H, H-3), 5.47 (s, 1H, C*H*Ph), 4.91 (d, *J* = 3.7 Hz, 1H, H-1), 4.64 (d, *J* = 12.5 Hz, 1H, C*H*H Bn), 4.59 (d, *J* = 12.4 Hz, 1H, CH*H* Bn), 4.28 (dd, *J* = 10.5, 4.9 Hz, 1H, H-6), 4.03 – 3.89 (m, 3H, H-5, C*H*HCF$_3$, CH*H*CF$_3$), 3.80 – 3.68 (m, 3H, H-2, H-4, H-6); $^{13}C\{^{1}H\}$ NMR (214 MHz, $CDCl_3$, HSQC, HMBC, HMBC-Gated) δ 165.5 ($C_{q\text{-benzoyl}}$), 137.5, 136.9 ($C_{q\text{-arom}}$), 133.1, 131.2 (CH_{arom}), 130.1 ($C_{q\text{-arom}}$), 129.9, 129.5, 129.1, 128.6, 128.5, 128.3, 128.2, 128.1, 126.2, 124.9 (CH_{arom}), 123.69 (q, *J* = 278.9 Hz, CF_3), 101.6 (CHPh), 98.5 (C-1), 79.3 (C-4), 77.1 (C-2), 72.9 (CH_2 Bn), 70.8 (C-3), 68.8 (C-6), 65.31 (dd, *J* = 70.3, 35.1 Hz, CH_2CF_3), 63.3 (C-5); ^{13}C-GATED NMR (214 MHz, $CDCl_3$) δ 98.5 ($J_{H1\text{-}C1}$ = 169 Hz, α); HRMS (ESI) M/Z: $[M + Na]^+$ Calcd for $C_{29}H_{27}F_3O_7Na$ 567.1601; Found 567.1599.

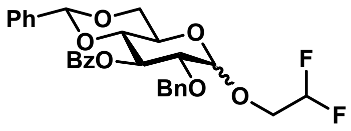

2,2-Difluoroethyl 3-*O*-benzoyl-2-*O*-benzyl-4,6-*O*-benzylidene-D-glucopyranoside (S10). The title compound was prepared according the general glycosylation protocol, using **2** as the donor and 2,2-difluoroethanol as the acceptor. Flash column chromatography (100:0 → 85:15, pentane:EtOAc v:v) yielded the title compound (39 mg, 0.074 mmol, 74%, α:β; 85:15) as a colorless oil. TLC: R_f 0.13, (90:10, pentane:EtOAc, v:v); Spectroscopic data for the major anomer: ^{1}H NMR (850 MHz, $CDCl_3$, HH-COSY, HSQC, HMBC, HMBC-Gated) δ 8.20 – 6.83 (m, 15H, CH_{arom}), 5.99 (tdd, *J* = 55.5, 4.7, 3.9 Hz, 1Hz, CHF_2), 5.83 (t, *J* = 9.7 Hz, 1H, H-3), 5.47 (s, 1H, C*H*Ph), 4.88 (d, *J* = 3.7 Hz, 1H, H-1), 4.63 (d, *J* = 12.3 Hz, 1H, C*H*H Bn), 4.59 (d, *J* = 12.4 Hz, 1H, CH*H* Bn), 4.29 (dd, *J* = 10.4, 4.9 Hz, 1H, H-6), 3.99 (td, *J* = 9.9, 4.9 Hz, 1H, H-5), 3.90 – 3.70 (m, 5H, H-2, H-4, H-6, C*H*HCHF$_2$, CH*H*CHF$_2$); $^{13}C\{^{1}H\}$ NMR (214 MHz, $CDCl_3$, HSQC, HMBC, HMBC-Gated) δ 165.5 ($C_{q\text{-benzoyl}}$), 137.5, 136.9 ($C_{q\text{-arom}}$), 133.2, 131.2 (CH_{arom}), 130.1 ($C_{q\text{-arom}}$), 130.0, 129.4, 129.1, 128.6, 128.5, 128.4, 128.3, 128.2, 128.1, 126.2, 124.9 (CH_{arom}), 114.2 (t, *J* = 241.4 Hz, CHF_2), 101.6 (CHPh), 98.4 (C-1), 79.4 (C-4), 77.3 (C-2), 73.0 (CH_2 Bn), 70.9 (C-3), 68.9 (C-6), 67.5 (t, J = 29.0 Hz, CH_2CHF_2), 63.1 (C-5); ^{13}C-GATED NMR (214 MHz, $CDCl_3$) δ 98.4 ($J_{H1\text{-}C1}$ = 173 Hz, α); Diagnostic peaks for the minor anomer: ^{1}H NMR (850 MHz, $CDCl_3$, HH-COSY, HSQC, HMBC, HMBC-Gated) δ 5.95 (tdd, *J* = 55.0, 4.8, 3.3 Hz, 1H, CHF_2), 4.79 (d, *J* = 11.6 Hz, 1H, C*H*H Bn), 4.70 (d, *J* = 7.5 Hz, 1H, H-1), 4.37 (dd, *J* = 10.6, 5.0 Hz, 1H, H-6), 3.63 – 3.56 (m, 2H, H-2, H-5); $^{13}C\{^{1}H\}$ NMR (214 MHz, $CDCl_3$, HSQC, HMBC, HMBC-Gated) δ 165.6 ($C_{q\text{-benzoyl}}$), 137.4, 136.8 ($C_{q\text{-arom}}$), 104.5 (C-1), 101.5 (CHPh), 79.1 (C-4), 78.7 (C-2), 74.5 (CH_2 Bn), 68.7 (C-6), 66.4 (C-5); ^{13}C-GATED NMR (214 MHz, $CDCl_3$) δ 104.5 ($J_{H1\text{-}C1}$ = 163 Hz, β); HRMS (ESI) M/Z: $[M + Na]^+$ Calcd for $C_{29}H_{28}F_2O_7Na$ 549.1695; Found 549.1693.

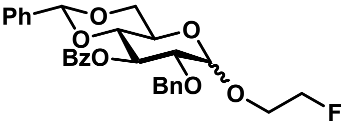

2-Fluoroethyl 3-*O*-benzoyl-2-*O*-benzyl-4,6-*O*-benzylidene-ᴅ-glucopyranoside (S11). The title compound was prepared according the general glycosylation protocol, using **2** as the donor and 2-fluoroethanol as the acceptor. Flash column chromatography (95:5 → 80:20, pentane:EtOAc v:v) yielded the title compound (quant, α:β; 45:55) as a colorless oil. TLC: R_f 0.06, (90:10, pentane:EtOAc, v:v); Spectroscopic data reported as a mixture of anomers: ^{1}H NMR (850 MHz, $CDCl_3$, HH-COSY, HSQC, HMBC, HMBC-Gated) δ 8.08 – 7.08 (m, 30H, CH_{arom}), 5.86 (t, *J* = 9.7 Hz, 1H, H-3 α), 5.59 (dd, *J* = 9.7, 9.1 Hz, 1H, H-3 β), 5.47 (s, 2H, C*H*Ph α, C*H*Ph β), 4.94 (d, *J* = 3.6 Hz, 1H, H-1 α), 4.83 (d, *J* = 11.6 Hz, 1H, C*H*H Bn β), 4.71 (d, *J* = 7.6 Hz, 1H, H-1 β), 4.69 – 4.58 (m, 7H, C*H*H Bn α, CH*H* Bn α, CH*H* Bn β, C*H*HF α, CH*H*F α, C*H*HF β, CH*H*F β), 4.37 (dd, *J* = 10.6, 5.0 Hz, 1H, H-6 β), 4.29 (dd, *J* = 10.4, 5.0 Hz, 1H, H-6 α), 4.14 (dddd, *J* = 31.8, 12.1, 5.1, 2.3 Hz, 1H, C*H*HCH_2F β), 4.04 (td, *J* = 9.9, 4.9 Hz, 1H, H-5 α), 3.95 – 3.85 (m, 2H, CH*H*CH_2F α, CH*H*CH_2F β), 3.84 – 3.76 (m, 2H, H-6 β, CH*H*CH_2F α), 3.76 – 3.69 (m, 4H, H-2 α, H-4 α, H-6 α, H-4 β), 3.61 (dd, *J* = 9.1, 7.5 Hz, 1H, H-2 β), 3.58 (td, *J* = 9.8, 5.0 Hz, 1H, H-5 β); $^{13}C\{^{1}H\}$ NMR (214 MHz, $CDCl_3$, HSQC, HMBC, HMBC-Gated) δ 165.6 ($C_{q\text{-benzoyl}}$ β), 165.5 ($C_{q\text{-benzoyl}}$ α), 145.6, 137.7, 137.6, 137.0, 136.9 ($C_{q\text{-arom}}$), 133.1, 133.0, 131.2jjjj, 130.2, 130.0 (CH_{arom}), 130.0, 129.9 ($C_{q\text{-arom}}$), 129.4, 129.1, 129.0, 128.5, 128.5, 128.4, 128.4, 128.3, 128.3, 128.2, 128.1, 128.0, 127.8, 126.2, 126.2, 124.9 (CH_{arom}), 104.4 (C-1 β), 101.5 (CHPh α), 101.4 (CHPh βf), 98.0 (C-1 α), 82.5 (d, *J* = 170.3 Hz, CH_2F β), 82.8 (d, *J* = 170.0 Hz, CH_2F α), 79.6 (C-2 α), 79.1 (C-2 β), 78.8 (C-4 β), 77.4 (C-4 α), 74.4 (CH_2 Bn β), 73.1 (C-3 β), 72.7(CH_2 Bn α), 71.1 (C-3 α), 69.4 (d, *J* = 19.9 Hz, CH_2CH_2F β), 69.0 (C-6 α), 68.8 (C-6 β), 67.5 (d, *J* = 20.1 Hz, CH_2CH_2F α), 66.3 (C-5 β), 62.7 (C-5 α); HRMS (ESI) M/Z: $[M + Na]^+$ Calcd for $C_{29}H_{29}FO_7Na$ 531.1790; Found 531.1788.

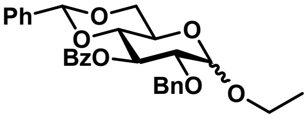

Ethyl 3-*O*-benzoyl-2-*O*-benzyl-4,6-*O*-benzylidene-ᴅ-glucopyranoside (S12). The title compound was prepared according the general glycosylation protocol, using **2** as the donor and ethanol as the acceptor. Flash column chromatography (100:0 → 90:10, pentane:EtOAc v:v) yielded the title compound (quant, α:β; 25:75) as a colorless oil. TLC: R_f 0.21, (90:10, pentane:EtOAc, v:v); Spectroscopic data for the major anomer: ^{1}H NMR (850 MHz, $CDCl_3$, HH-COSY, HSQC, HMBC, HMBC-Gated) δ 8.14 – 7.03 (m, 15H, CH_{arom}), 5.58 (t, *J* = 9.5 Hz, 1H, H-3), 5.47 (s, 1H, C*H*Ph), 4.82 (d, *J* = 11.7 Hz, 1H, C*H*H Bn), 4.68 – 4.65 (m, 2H, H-1, C*H*H Bn), 4.63, 4.37 (dd, *J* = 10.6, 5.0 Hz, 1H, H-6), 4.06 – 3.99 (m, 1H, C*H*HCH_3), 3.80 (t, *J* = 10.3 Hz, 1H, H-6), 3.78 – 3.67 (m, 2H, H-4, H-5), 3.59 – 3.55 (m, 2H, H-2, CH*H*CH_3), 1.32 (t, *J* = 7.1 Hz, 3H, CH_3); $^{13}C\{^{1}H\}$ NMR (214 MHz, $CDCl_3$, HSQC, HMBC, HMBC-Gated) δ 165.6 ($C_{q\text{-benzoyl}}$), 137.7, 137.0 ($C_{q\text{-arom}}$), 133.1 (CH_{arom}), 130.1 ($C_{q\text{-arom}}$), 130.0, 129.1, 128.4, 128.4, 128.3, 128.3, 127.7, 126.2 (CH_{arom}), 104.2 (C-1), 101.4 (CHPh), 79.4 (C-2), 79.0 (C-4), 74.4 (CH_2 Bn), 73.2 (C-3), 68.9 (C-6), 66.3 (C-5), 66.3 (CH_2CH_3), 15.5 (CH_3); Spectroscopic data for the minor anomer: ^{1}H NMR (850 MHz, $CDCl_3$, HH-COSY, HSQC, HMBC, HMBC-Gated) δ 8.14 – 7.03 (m, 15H, CH_{arom}), 5.86 (t, *J* = 9.7 Hz, 1H, H-3), 5.47 (s, 1H, C*H*Ph), 4.86 (d, *J* = 3.6 Hz, 1H, H-1), 4.64 (d, *J* = 12.5 Hz, 1H, C*H*H Bn), 4.60 (d, *J* = 12.5 Hz, 1H, CH*H* Bn), 4.28 (dd, *J* = 10.4, 4.9 Hz, 1H, H-6), 4.06 – 3.99 (m, 1H, H-5), 3.78 – 3.67 (m, 4H, H-2, H-4, H-6, C*H*HCH_3), 3.53 (dq, *J* = 9.7, 7.0 Hz, 1H, C*H*HCH_3), 1.30 (t, *J* = 7.1 Hz, 3H, CH_3); $^{13}C\{^{1}H\}$ NMR (214 MHz, $CDCl_3$, HSQC, HMBC-Gated) δ 165.5 ($C_{q\text{-benzoyl}}$), 137.8, 137.1, 133.1 ($C_{q\text{-arom}}$), 133.0 (CH_{arom}), 130.3 ($C_{q\text{-arom}}$), 129.9, 129.0, 128.5, 128.4, 128.2, 128.0, 126.2 (CH_{arom}), 101.5 (CHPh), 97.5 (C-1), 79.9 (C-2), 77.5 (C-4), 72.7 (CH_2 Bn), 71.3 (C-3), 69.1 (C-6), 64.0 (CH_2CH_3), 62.6 (C-5), 15.5 (CH_3); HRMS (ESI) M/Z: $[M + Na]^+$ Calcd for $C_{29}H_{30}O_7Na$ 513.1884; Found 513.1880.

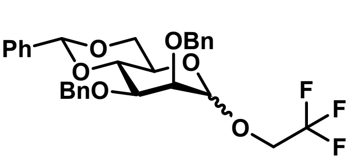

2,2,2-Trifluoroethyl 2,3-di-*O*-benzyl-4,6-*O*-benzylidene-ᴅ-mannopyranoside (S13). The title compound was prepared according the general glycosylation protocol, using **3** as the donor and 2,2,2-trifluoroethanol as the acceptor. Flash column chromatography (100:0 → 90:10, pentane:EtOAc v:v) yielded the title compound (27 mg,

0.051 mmol, 51%, α:β; 23:77) as a colorless oil. TLC: R_f 0.52, (90:10, pentane:EtOAc, v:v); ^{1}H NMR (850 MHz, $CDCl_3$, HH-COSY, HSQC, HMBC, HMBC-Gated) and $^{13}C\{^1H\}$ NMR (214 MHz, $CDCl_3$, HSQC, HMBC-Gated) were in accordance to literature[16]; HRMS (ESI) M/Z: $[M + Na]^+$ Calcd for $C_{29}H_{29}F_3O_6Na$ 553.1808; Found 553.1805.

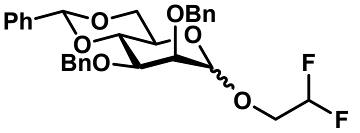

2,2-Difluoroethyl 2,3-di-*O*-benzyl-4,6-*O*-benzylidene-ᴅ-mannopyranoside (S14). The title compound was prepared according the general glycosylation protocol, using **3** as the donor and 2,2-difluoroethanol as the acceptor. Flash column chromatography (100:0 → 85:15, pentane:EtOAc v:v) yielded the title compound (30 mg, 0.059 mmol, 59%, α:β; 18:82) as a colorless oil. TLC: R_f 0.34, (90:10, pentane:EtOAc, v:v); ^{1}H NMR (850 MHz, $CDCl_3$, HH-COSY, HSQC, HMBC, HMBC-Gated) and $^{13}C\{^1H\}$ NMR (214 MHz, $CDCl_3$, HSQC, HMBC-Gated) were in accordance to literature[16]; HRMS (ESI) M/Z: $[M + Na]^+$ Calcd for $C_{29}H_{30}F_2O_6Na$ 535.1903; Found 535.1902.

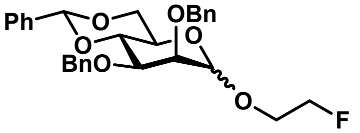

2-Fluoroethyl 2,3-di-*O*-benzyl-4,6-*O*-benzylidene-ᴅ-mannopyranoside (S15). The title compound was prepared according the general glycosylation protocol, using **3** as the donor and 2-fluoroethanol as the acceptor. Flash column chromatography (95:5 → 80:20, pentane:EtOAc v:v) yielded the title compound (30 mg, 0.061 mmol, 61%, α:β; 17:83) as a colorless oil. TLC: R_f 0.17, (90:10, pentane:EtOAc, v:v); ^{1}H NMR (850 MHz, $CDCl_3$, HH-COSY, HSQC, HMBC, HMBC-Gated) and $^{13}C\{^1H\}$ NMR (214 MHz, $CDCl_3$, HSQC, HMBC-Gated) were in accordance to literature[16]; HRMS (ESI) M/Z: $[M + Na]^+$ Calcd for $C_{29}H_{31}FO_6Na$ 517.1997; Found 517.1997.

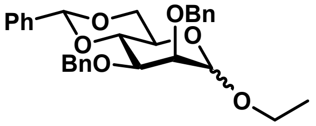

Ethyl 2,3-di-*O*-benzyl-4,6-*O*-benzylidene-ᴅ-mannopyranoside (S16). The title compound was prepared according the general glycosylation protocol, using **3** as the donor and ethanol as the acceptor. Flash column chromatography (100:0 → 90:10, pentane:EtOAc v:v) yielded the title compound (26 mg, 0.055 mmol, 55%, α:β; 19:81) as a colorless oil. TLC: R_f 0.42, (90:10, pentane:EtOAc, v:v); ^{1}H NMR (850 MHz, $CDCl_3$, HH-COSY, HSQC, HMBC, HMBC-Gated) and $^{13}C\{^1H\}$ NMR (214 MHz, $CDCl_3$, HSQC, HMBC-Gated) were in accordance to literature[16]; HRMS (ESI) M/Z: $[M + Na]^+$ Calcd for $C_{29}H_{32}O_6Na$ 499.2091; Found 499.2088.

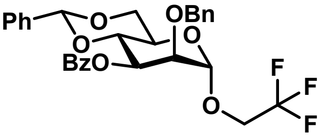

2,2,2-Trifluoroethyl 3-*O*-benzoyl-2-*O*-benzyl-4,6-*O*-benzylidene-ᴅ-mannopyranoside (S17). The title compound was prepared according the general glycosylation protocol, using **4** as the donor and 2,2,2-trifluoroethanol as the acceptor. Flash column chromatography (100:0 → 90:10, pentane:EtOAc v:v) yielded the title compound (45 mg, 0.083 mmol, 83%, α:β; >98:2) as a colorless oil. TLC: R_f 0.46, (90:10, pentane:EtOAc, v:v); ^{1}H NMR (850 MHz, $CDCl_3$, HH-COSY, HSQC, HMBC, HMBC-Gated) δ 8.09 – 7.19 (m, 15H, CH_{arom}), 5.62 (s, 1H, C*H*Ph), 5.56 (dd, J = 10.4, 3.6 Hz, 1H, H-3), 4.94 (d, J = 1.6 Hz, 1H, H-1), 4.65 (d, J = 12.0 Hz, 1H, C*H*H Bn), 4.62 (d, J = 11.9 Hz, 1H, CH*H* Bn), 4.37 (dd, J = 10.5, 9.4 Hz, 1H, H-4), 4.29 (dd, J = 10.2, 4.7 Hz, 1H, H-6), 4.18 (dd, J = 3.6, 1.6 Hz, 1H, H-2), 4.01 – 3.95 (m, 2H, H-5, C*H*HCF$_3$), 3.95 – 3.88 (m, 2H, CH*H*CF$_3$); $^{13}C\{^1H\}$ NMR (214 MHz, $CDCl_3$, HSQC, HMBC, HMBC-Gated) δ 165.8 ($C_{q\text{-}benzoyl}$), 137.2, 137.2 ($C_{q\text{-}arom}$), 133.3 (CH_{arom}), 130.0 ($C_{q\text{-}arom}$), 129.9, 128.7, 128.7, 128.6, 128.6, 128.5, 128.5, 128.4, 128.3, 128.2, 128.2, 128.1, 128.0, 126.2 (CH_{arom}), 123.6 (q, *J* = 278.6 Hz, CF_3), 101.9 (CHPh), 99.6 (C-1), 76.1 (C-4), 75.9 (C-2), 74.2 (CH_2 Bn), 70.7 (C-3), 68.6, (C-6) 64.9 (C-5), 64.55 (q, *J* = 35.2 Hz, CH_2CF_3);

^{13}C-GATED NMR (214 MHz, CDCl3) δ 99.6 (J_{H1-C1} = 172 Hz, α); HRMS (ESI) M/Z: [M + Na]$^+$ Calcd for $C_{29}H_{27}F_3O_7Na$ 567.1601; Found 567.1601.

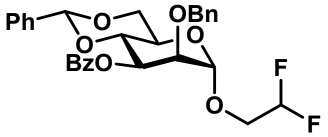

2,2-Difluoroethyl 3-*O*-benzoyl-2-*O*-benzyl-4,6-*O*-benzylidene-ᴅ-mannopyranoside (S18). The title compound was prepared according the general glycosylation protocol, using **4** as the donor and 2,2-difluoroethanol as the acceptor. Flash column chromatography (100:0 → 85:15, pentane:EtOAc v:v) yielded the title compound (38 mg, 0.072 mmol, 72%, α:β; >98:2) as a colorless oil. TLC: R_f 0.35, (90:10, pentane:EtOAc, v:v); ^{1}H NMR (850 MHz, $CDCl_3$, HH-COSY, HSQC, HMBC, HMBC-Gated) δ 8.10 – 7.15 (m, 15H, CH_{arom}), 5.92 (tdd, *J* = 55.4, 4.7, 3.6 Hz, 1H, CHF_2), 5.62 (s, 1H), 5.54 (dd, *J* = 10.4, 3.5 Hz, 1H, C*H*Ph), 4.91 (d, *J* = 1.6 Hz, 1H, H-3), 4.67 – 4.60 (m, 2H, C*H*H Bn, CH*H* Bn), 4.36 (dd, *J* = 10.5, 9.4 Hz, 1H, H-4), 4.29 (dd, *J* = 10.2, 4.7 Hz, 1H, H-6), 4.15 (dd, *J* = 3.5, 1.7 Hz, 1H, H-2), 3.97 (td, *J* = 9.9, 4.7 Hz, 1H, H-5), 3.91 (t, *J* = 10.3 Hz, 1H, H-6), 3.85 (dddd, *J* = 16.2, 12.9, 11.7, 3.7 Hz, 1H, C*H*HCHF$_2$), 3.72 (tdd, *J* = 12.7, 11.7, 4.7 Hz, 1H, CH*H*CHF$_2$); ^{13}C{^{1}H} NMR (214 MHz, $CDCl_3$, HSQC, HMBC, HMBC-Gated) δ 165.9 ($C_{q\text{-benzoyl}}$), 137.3, 137.3 ($C_{q\text{-arom}}$), 133.3, 130.0 (CH_{arom}), 129.9 ($C_{q\text{-arom}}$), 129.1, 128.6, 128.5, 128.3, 128.2, 128.1, 126.2 (CH_{arom}), 114.0 (t, *J* = 241.4 Hz, CHF_2), 101.8 (CHPh), 99.6 (C-1), 76.2 (C-4), 76.0 (C-2), 74.1 (CH_2 Bn), 70.9 (C-3), 68.7 (C-6), 66.8 (t, J = 28.6 Hz, CH_2CHF_2), 64.6 (C-5); ^{13}C-GATED NMR (214 MHz, CDCl3) δ 99.6 (J_{H1-C1} = 173 Hz, α); HRMS (ESI) M/Z: [M + Na]$^+$ Calcd for $C_{29}H_{28}F_2O_7Na$ 549.1695; Found 549.1697.

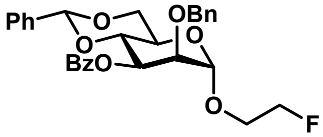

2-Fluoroethyl 3-*O*-benzoyl-2-*O*-benzyl-4,6-*O*-benzylidene-ᴅ-mannopyranoside (S19). The title compound was prepared according the general glycosylation protocol, using **4** as the donor and 2-fluoroethanol as the acceptor. Flash column chromatography (95:5 → 80:20, pentane:EtOAc v:v) yielded the title compound (48 mg, 0.094 mmol, 94%, α:β; >98:2) as a colorless oil. TLC: R_f 0.20, (90:10, pentane:EtOAc, v:v); ^{1}H NMR (850 MHz, $CDCl_3$, HH-COSY, HSQC, HMBC, HMBC-Gated) δ 8.15 – 7.17 (m, 15H, CH_{arom}), 5.62 (s, 1H, C*H*Ph), 5.60 (dd, *J* = 10.4, 3.5 Hz, 1H, H-3), 4.94 (d, *J* = 1.7 Hz, 1H, H-1), 4.66 – 4.63 (m, 2H, C*H*H Bn), 4.60 (dtdt, *J* = 10.6, 7.8, 4.9, 2.6 Hz, 1H, C*H*HF), 4.58 – 4.52 (m, 1H, CH*H*F), 4.36 (dd, *J* = 10.5, 9.4 Hz, 1H, H-4), 4.29 (dd, *J* = 10.3, 4.8 Hz, 1H, H-6), 4.15 (dd, *J* = 3.5, 1.7 Hz, 1H, H-2), 4.02 (td, *J* = 9.9, 4.8 Hz, 1H, H-5), 3.95 – 3.86 (m, 2H, H-6, C*H*HCH$_2$F), 3.75 (dddd, *J* = 27.0, 12.2, 6.3, 2.8 Hz, 1H, C*H*HCH$_2$F); ^{13}C{^{1}H} NMR (214 MHz, $CDCl_3$, HSQC, HMBC, HMBC-Gated) δ ^{13}C{^{1}H} NMR (214 MHz, $CDCl_3$) δ 165.9 ($C_{q\text{-benzoyl}}$), 145.6, 137.5 ($C_{q\text{-arom}}$), 137.3, 133.2, 131.2, 130.0 (CH_{arom}), 130.0 ($C_{q\text{-arom}}$), 129.4, 129.0, 128.5, 128.5, 128.3, 128.0, 128.0, 126.2, 124.9 (CH_{arom}), 101.8 (CHPh), 99.3 (C-1), 82.5 (d, *J* = 170.1 Hz, CH_2F), 76.4 (C-4), 76.4 (C-2), 73.9 (CH_2 Bn), 71.1 (C-3), 68.8 (C-6), 66.9 (d, *J* = 20.0 Hz, CH_2CH_2F), 64.3 (C-5); ^{13}C-GATED NMR (214 MHz, CDCl3) δ 99.3 (J_{H1-C1} = 171 Hz, α); HRMS (ESI) M/Z: [M + Na]$^+$ Calcd for $C_{29}H_{29}FO_7Na$ 531.1790; Found 531.1791.

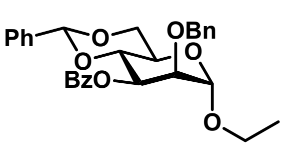

Ethyl 3-*O*-benzoyl-2-*O*-benzyl-4,6-*O*-benzylidene-ᴅ-mannopyranoside (S20). The title compound was prepared according the general glycosylation protocol, using **4** as the donor and ethanol as the acceptor. Flash column chromatography (100:0 → 90:10, pentane:EtOAc v:v) yielded the title compound (36 mg, 0.073 mmol, 73%, α:β; >98:2) as a colorless oil. TLC: R_f 0.48, (90:10, pentane:EtOAc, v:v); ^{1}H NMR (850 MHz, $CDCl_3$, HH-COSY, HSQC, HMBC, HMBC-Gated) δ 8.16 – 7.15 (m, 15H, CH_{arom}), 5.62 (s, 1H, C*H*Ph), 5.60 (dd, *J* = 10.4, 3.5 Hz, 1H, H-3), 4.87 (d, *J* = 1.7 Hz, 1Hz, H-1), 4.65 (s, 2H, CH_2 Bn), 4.34 (dd, *J* = 10.5, 9.4 Hz, 1H, H-4), 4.29 (dd, *J* = 10.3, 4.7 Hz, 1H, H-6), 4.08 (dd, *J* = 3.6, 1.7 Hz, 1H, H-2), 4.00 (td, *J* = 9.9, 4.7 Hz, 1H, H-5), 3.91 (t, *J* = 10.3 Hz, 1H, H-6), 3.77 (dq, *J* = 9.6, 7.1 Hz, 1H, C*H*HCH$_3$), 3.48 (dq, *J* = 9.6, 7.0 Hz, 1H, CH*H*CH$_3$), 1.23 (t, *J* = 7.1 Hz, 3H, CH_3); ^{13}C{^{1}H} NMR (214

MHz, $CDCl_3$, HSQC, HMBC, HMBC-Gated) δ 165.9 ($C_{q\text{-benzoyl}}$), 137.7, 137.4 ($C_{q\text{-arom}}$), 133.2 (CH_{arom}), 130.1 ($C_{q\text{-arom}}$), 129.0, 128.5, 128.5, 128.3, 128.0, 128.0, 126.2 (CH_{arom}), 101.8 (CHPh), 98.9 (C-1), 76.7 (C-2), 76.6 (C-4), 73.9 (CH_2 Bn), 71.3 (C-3), 69.0 (C-6), 64.1 (C-5), 63.5 (CH_2CH_3), 15.1 (CH_3); ^{13}C-GATED NMR (214 MHz, $CDCl_3$) δ 98.9 ($J_{H1\text{-}C1}$ = 170 Hz, α); HRMS (ESI) M/Z: $[M + Na]^+$ Calcd for $C_{29}H_{30}O_7Na$ 513.1884; Found 513.1884.

Preparation of the IR donors 5-6

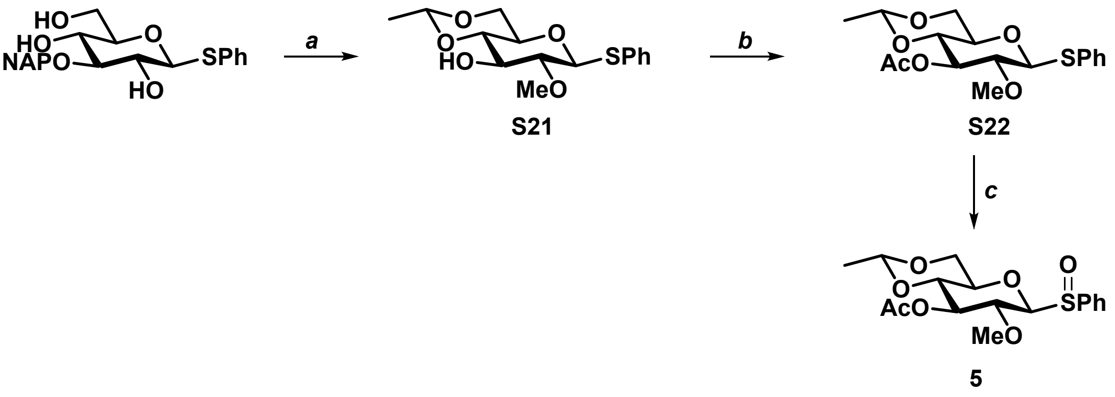

Supplementary scheme S3. Glucoside donor **5** synthesis. *Reagents and conditions:* a) 1. 1,1-dimethoxyethane, camphorsulfonic acid, acetonitril, RT, 2. DDQ, DCM:H_2O, **S21**: 66%; b) acetic anhydride, pyridine, RT, **S22**: 63%; c) meta-chloroperoxybenzoic acid, DCM, **5**: quant.

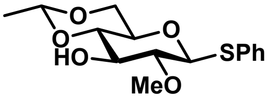

Phenyl 4,6-*O*-ethylidene-2-*O*-methyl-1-thio-β-D-glucopyranose (S21). To a solution of phenyl 2-O-methyl-3-O-(2-methyl)naphthyl-1-thio-β-d-glucopyranoside (50 mg, 0.12 mmol) in dry acetonitrile (1.2 ml, 0.1M) and 1,1-dimethoxyethane (0.033 ml, 0.35 mmol, 3 eq.), CSA (2.7 mg, 0.012 mmol, 0.1 eq.) was added. The mixture stirred under argon atmosphere for 16 hours before being quenched by addition of triethylamine (0.10 ml). The resulting solution was concentrated *in vacuo*, diluted with EtOAc and washed with sat. aq. $NaHCO_3$ and brine. The organic layer was dried over $MgSO_4$, filtered and concentrated *in vacuo*. To a solution of the crude phenyl 2-*O*-methyl-3-*O*-(2-methyl)naphthyl-4,6-*O*-ethylidene-1-thio-β-d-glucopyranoside (20 mg, 0.044 mmol) in DCM/H_2O (7/1, 0.5 ml, 0.1M) was added DDQ (15 mg, 0.066 mmol, 1.5 eq.) and the reaction mixture was stirred in the dark to completion. The reaction mixture was diluted using DCM (5 mL). The organic phase was washed twice using an aqueous mixture of ascorbic acid (0.7%), citric acid (1.5%) and NaOH (0.9%) (w/v). The organic layer way dried over $MgSO_4$, filtered and concentrated *in vacuo*. Flash column chromatography (75:0 → 70:30, pentane:*n*-heptane v:v) yielded the title compound (14 mg, 0.044 mmol, 66%) as a white amorphous solid. TLC: R_f 0.28, (50:50, *n*-heptane:EtOAc, v:v); ^1H NMR (500 MHz, $CDCl_3$) δ 7.56 – 7.46 (m, 2H, CH_{arom}), 7.37 – 7.26 (m, 3H, CH_{arom}), 4.72 (q, *J* = 5.0 Hz, 1H, CH_3CH), 4.61 (d, *J* = 9.8 Hz, 1H, H-1), 4.26 – 4.10 (m, 1H, H-6), 3.76 (ddt, *J* = 8.7, 5.8, 2.3 Hz, 1H, H-3), 3.67 (s, 1H, OCH_3), 3.63 – 3.44 (m, 1H, H-6), 3.35 – 3.20 (m, 2H, H-4, H-5), 3.11 (dd, *J* = 9.7, 8.4 Hz, 1H, H-2), 2.70 (d, *J* = 2.5 Hz, 1H, 3-O*H*), 1.36 (d, *J* = 5.0 Hz, 3H, CH_3CH); $^{13}C\{^1H\}$ NMR (126 MHz, $CDCl_3$) δ 133.2, 132.2, 129.1, 128.0 (CH_{arom}), 99.8 (CH_3CH), 87.8 (C-1), 82.8 (C-2), 79.7 (C-4), 75.5 (C-3), 70.3 (C-5), 68.3 (C-6), 61.5 (OCH_3), 20.4 (CH_3CH); HRMS (ESI): $[M + Na]^+$ Calcd. for $C_{15}H_{20}O_5SNa$ 335.0924, found 335.09332.

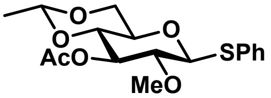

Phenyl 3-*O*-acetyl-4,6-*O*-ethylidene-2-*O*-methyl-1-thio-β-D-glucopyranose (S22). To a solution of **S21** (14 mg, 1 Eq, 45 µmol) pyridine (0.1 mL, 1 mmol) were added acetic anhydride (0.42 ml, 0.45 mmol, 10 eq.) and DMAP (0.55 mg, 4.5 µmol, 0.1 eq.). The reaction mixture was stirred at room temperature until TLC showed complete conversion after which it was concentrated *in vacuo.* The crude product was then dissolved in EtOAc and washed with sat. aq. $CuSO_4$. The organic layer was dried over $MgSO_4$, filtered and concentrated *in vacuo.* The crude material was then purified using silica gel flash column chromatography (100:0 → 70:30 *n*-heptane:EtOAc, v:v)

to obtain phenyl 3-*O*-acetyl-2-*O*-methyl-4,6-*O*-ethylidene-1-thio-β-D-glucopyranose (10 mg, 0.028 mmol, 63 %) as a waxy solid. TLC: R_f 0.46 (EtOAc/*n*-heptane, 1:1 v/v); ^{1}H NMR (400 MHz, $CDCl_3$, HH-COSY, HSQC) δ 7.55 – 7.46 (m, 2H, CH_{arom}), 7.36 – 7.29 (m, 3H, CH_{arom}), 5.27 – 5.13 (m, 1H, H-3), 4.70 – 4.62 (m, 2H, H-1, CH_3CH), 4.21 – 4.13 (m, 1H, H-6), 3.53 (s, 4H, H-6, OCH_3), 3.40 – 3.30 (m, 2H, H-4, H-5), 3.19 (dd, *J* = 9.8, 8.8 Hz, 1H, H-2), 2.12 (s, 3H, CH_3 Ac), 1.31 (d, *J* = 5.1 Hz, 3H, CH_3CH). $^{13}C\{^1H\}$ NMR (101 MHz, $CDCl_3$) δ 170.0 ($C_{q\text{-acetyl}}$), 133.0, 132.5, 129.2, 128.1 (CH_{arom}), 99.7 (CH_3CH), 88.0 (C-1), 81.0 (C-2), 78.2 (C-4), 74.6 (C-3), 70.5 (C-5), 68.2 (C-6), 60.7 (OCH_3), 21.2 (CH_3 Ac), 20.4 (CH_3CH); HRMS (ESI): $[M + Na]^+$ Calcd for $C_{17}H_{22}O_6SNa$ 377.1029; found 377.1046.

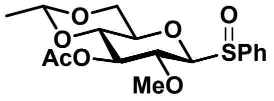

Phenyl 3-*O*-acetyl-4,6-*O*-ethylidene-2-*O*-methyl-1-thiosulfinyl-β-ᴅ-glucopyranose (5). A solution of **S22** (10 mg, 0.028 mmol) in DCM (1.0 ml, 0.05M) was cooled to –78 °C under inert atmosphere and then meta-chloroperoxybenzoic acid (1.1 eq., 75 wt%) was added. The reaction was stirred for 3 h, diluted with DCM and washed with 10% aq. $Na_2S_2O_3$ solution, sat. aq. $NaHCO_3$ and brine. The organic layer was dried over $MgSO_4$, filtered, and concentrated *in vacuo*. The resulting crude mixture was used directly for IRMPD experiments. HRMS (ESI): $[M + Na]^+$ Calcd for $C_{17}H_{22}O_7SNa$, 393.0978; found, 393.0993.

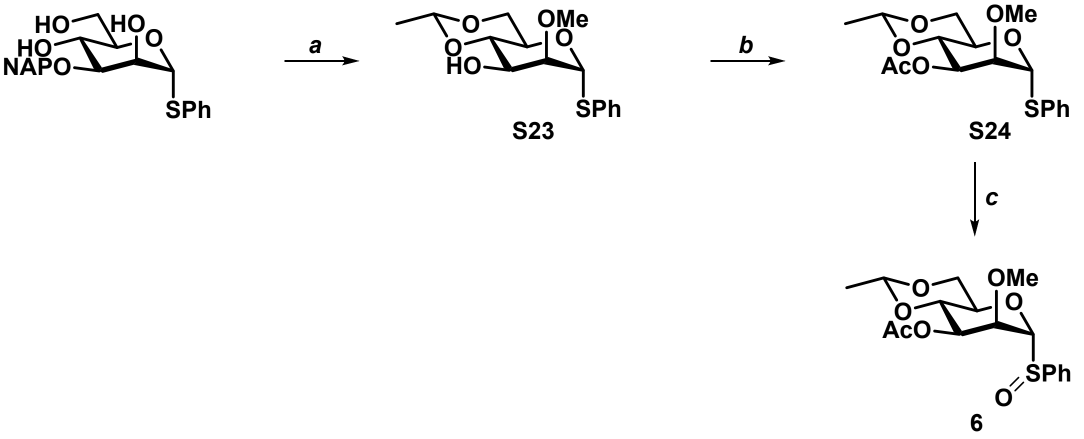

Supplementary scheme S4. Mannoside donor **6** synthesis. *Reagents and conditions:* a) 1. 1,1-dimethoxyethane, camphorsulfonic acid, acetonitril, RT; 2. DDQ, DCM:H_2O, **S23**: 66%; b) acetic anhydride, pyridine, RT, **S24**: 63%; c) meta-chloroperoxybenzoic acid, DCM, **6**: quant.

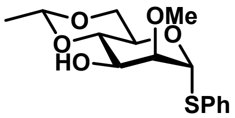

Phenyl 4,6-*O*-ethylidene-2-*O*-methyl-1-thio-α-ᴅ-mannopyranose (S23). To a solution of phenyl 2-O-methyl-3-O-(2-methyl)naphthyl-1-thio-α-ᴅ-mannopyranoside (250 mg, 0.59 mmol) in dry acetonitrile (5.9 ml, 0.1M) and 1,1-dimethoxyethane (1.76 mmol, 3 eq.), CSA (14 mg, 0.059 mmol, eq.) was added. The mixture stirred under argon atmosphere for 18 hours before being quenched by addition of triethylamine (0.10 mL). The resulting solution was concentrated *in vacuo*, diluted with EtOAc and washed with sat. aq. $NaHCO_3$ and brine. The organic layer was dried over $MgSO_4$, filtered and concentrated *in vacuo*. To a solution of the crude phenyl 2-*O*-methyl-3-*O*-(2-methyl)naphthyl-4,6-*O*-ethylidene-1-thio-α-ᴅ-glucopyranoside (200 mg, 0.44 mmol) in DCM/H_2O (7/1, 5 ml, 0.1M) was added DDQ (150 mg, 0.66 mmol, 1.5 eq.) and the reaction mixture was stirred in the dark to completion. The reaction mixture was diluted with DCM. The organic phase was washed twice using an aqueous mixture of ascorbic acid (0.7%), citric acid (1.5%) and NaOH (0.9%) (w/v). The organic layer way dried over $MgSO_4$, filtered and concentrated *in vacuo*. Flash column chromatography (75:0 → 70:30, pentane:*n*-heptane v:v) yielded the title compound (120 mg, 0.38 mmol, 87%) as a white waxy solid. TLC: R_f 0.21, (50:50, *n*-heptane:EtOAc, v:v); ^{1}H NMR (500 MHz, $CDCl_3$) δ 7.52 – 7.42 (m, 2H, CH_{arom}), 7.39 – 7.24 (m, 3H, CH_{arom}), 5.62 (d, *J* = 1.2 Hz, 1H, H-1), 4.77 (q, *J* = 5.0 Hz, 1H, CH_3CH), 4.15 (td, *J* = 9.8, 4.9 Hz, 1H, H-5), 4.03 (dd, *J* = 10.3, 4.9 Hz, 1H, H-6), 4.00 (d, *J* =

8.6 Hz, 1H, H-3), 3.82 (dd, *J* = 3.7, 1.3 Hz, 1H, H-2), 3.67 (t, *J* = 9.7 Hz, 1H, H-4), 3.60 (t, *J* = 10.3 Hz, 1H, H-6), 3.50 (s, 3H, OCH_3), 2.46 (bs, 1H, 3-O*H*), 1.39 (d, *J* = 5.1 Hz, 3H, CH_3CH); $^{13}C\{^1H\}$ NMR (126 MHz, $CDCl_3$) δ 133.8, 131.7, 129.3, 127.9 (CH_{arom}), 100.1 (*C*H ethylidene), 85.1 (C-1), 82.2 (C-2), 79.1 (C-4), 69.1 (C-3), 68.1 (C-6), 64.7 (C-5), 58.8 (OCH_3), 20.7 (CH_3CH); HRMS (ESI): $[M + Na]^+$ Calcd. for $C_{15}H_{20}O_5SNa$ 335.0924, found 335.0937.

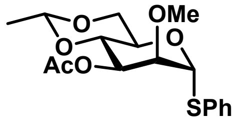

Phenyl 3-*O*-acetyl-4,6-*O*-ethylidene-2-*O*-methyl-1-thio-α-ᴅ-mannopyranose (S24). To a solution of **S23** (120 mg, 0.38 mmol) in pyridine (1.0 mL, 0.38M) were added acetic anhydride (0.5 ml, 0.45 mmol, 5 eq.) and DMAP (4.69 mg, 0.038 mmol, 0.1 eq.). The reaction mixture was stirred at room temperature for two hours after which it was concentrated *in vacuo.* The crude product was then dissolved in EtOAc and washed with sat. aq. $CuSO_4$ and brine. The organic layer was dried over $MgSO_4$, filtered and concentrated *in vacuo.* The crude material was then purified using silica gel flash column chromatography (100:0 → 80:20 *n*-heptane:EtOAc, v:v) to obtain phenyl 3-*O*-acetyl-2-*O*-methyl-4,6-*O*-ethylidene-1-thio-β-ᴅ-mannopyranose (15 mg, 0.042 mmol, 11 %) as a waxy solid. TLC: R_f 0.42 (EtOAc/*n*-heptane, 1:1 v/v); ^1H NMR (500 MHz, $CDCl_3$) δ 7.52 – 7.42 (m, 2H, CH_{arom}), 7.40 – 7.16 (m, 3H, CH_{arom}), 5.56 (d, *J* = 1.4 Hz, 1H, H-1), 5.18 (dd, *J* = 10.5, 3.3 Hz, 1H, H-3), 4.75 (q, *J* = 5.0 Hz, 1H, CH_3CH), 4.25 (td, *J* = 9.9, 4.8 Hz, 1H, H-5), 4.04 (dd, *J* = 10.4, 4.9 Hz, 1H, H-6), 3.98 (dd, *J* = 3.4, 1.5 Hz, 1H, H-2), 3.93 (dd, *J* = 10.5, 9.4 Hz, 1H, H-4), 3.63 (t, *J* = 10.3 Hz, 1H, H-6), 3.44 (s, 3H, OCH_3), 2.15 (s, 3H, CH_3 Ac), 1.35 (d, *J* = 5.0 Hz, 3H, CH_3CH); $^{13}C\{^1H\}$ NMR (126 MHz, $CDCl_3$) δ 170.4 ($C_{q\text{-}acetyl}$), 133.9, 131.6, 129.3, 127.8 (CH_{arom}), 100.0 (CH_3CH), 85.8 (C-1), 80.2 (C-2), 75.8 (C-4), 70.9 (C-3), 68.1 (C-6), 65.2 (C-5), 59.2 (OCH_3), 21.2 (CH_3 Ac), 20.5 (CH_3CH); HRMS (ESI): $[M + Na]^+$ Calcd for $C_{17}H_{22}O_6SNa$ 377.1029; found 377.1510.

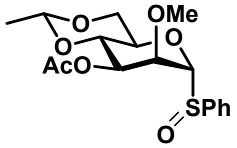

Phenyl 3-*O*-acetyl-4,6-*O*-ethylidene-2-*O*-methyl-1-thiosulfinyl-α-ᴅ-mannopyranose (6). A solution of **S23** (14.5 mg, 0.041 mmol) in DCM (1.0 ml, 0.05M) was cooled to –78 °C under inert atmosphere and then meta-chloroperoxybenzoic acid (1.1 eq., 75 wt%) was added. The reaction was stirred for 3 h, diluted with DCM and washed with 10% aq. $Na_2S_2O_3$ solution, sat. aq. $NaHCO_3$ and brine. The organic layer was dried over $MgSO_4$, filtered, and concentrated *in vacuo*. The resulting crude mixture was used directly for IRMPD experiments. HRMS (ESI): $[M + Na]^+$ Calcd for $C_{17}H_{22}O_7SNa$, 393.0978; found, 393.0996.

NMR spectra of new and selected compounds

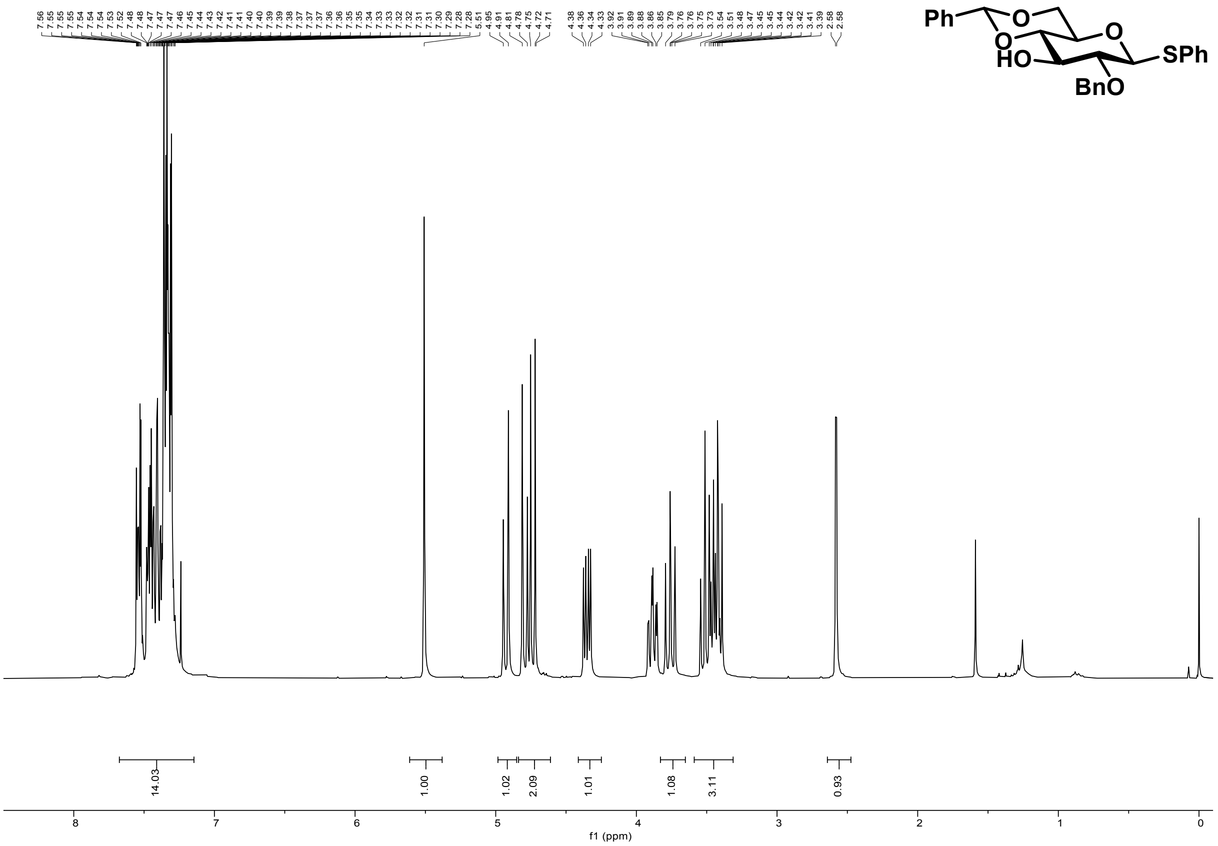

Supplementary Figure S7. ^{1}H NMR, 300 MHz, $CDCl_3$ of compound **S1**

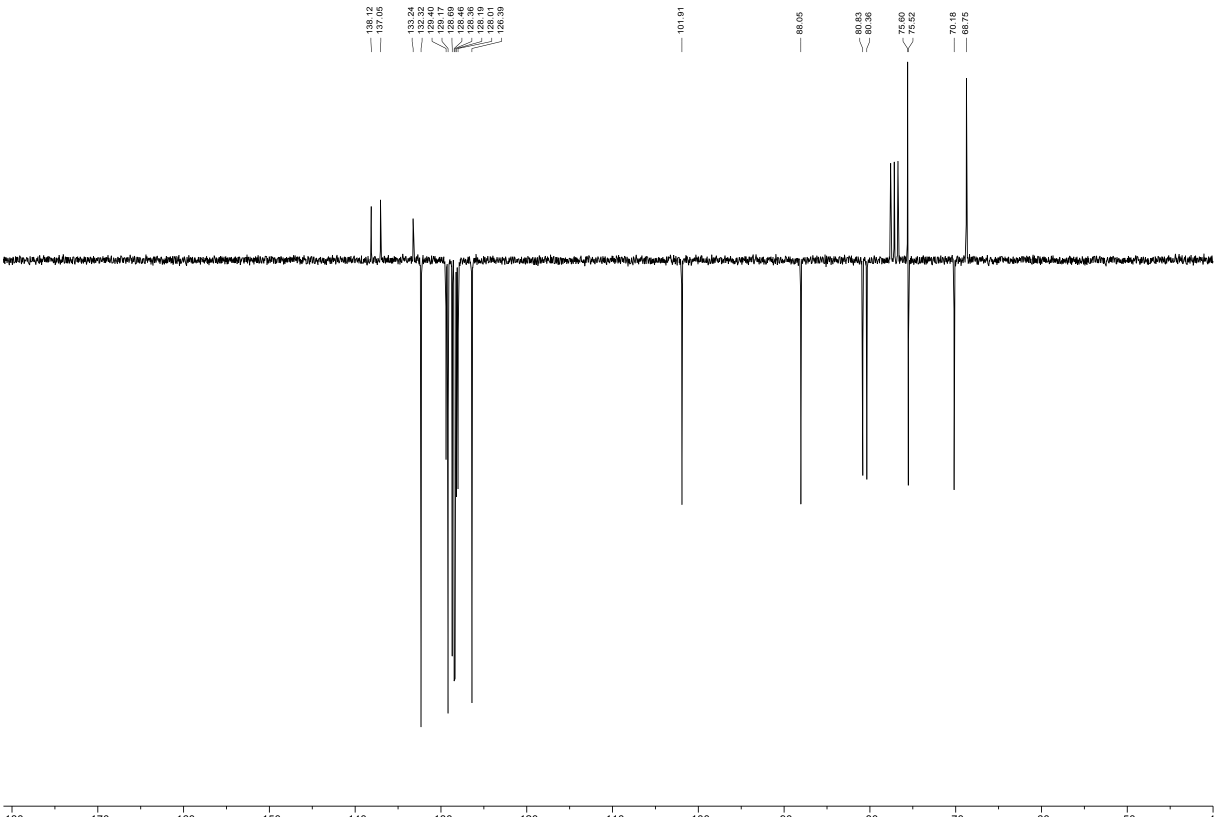

Supplementary Figure S8. $^{13}C\{^{1}H\}$ NMR, 75 MHz, $CDCl_3$ of compound **S1**

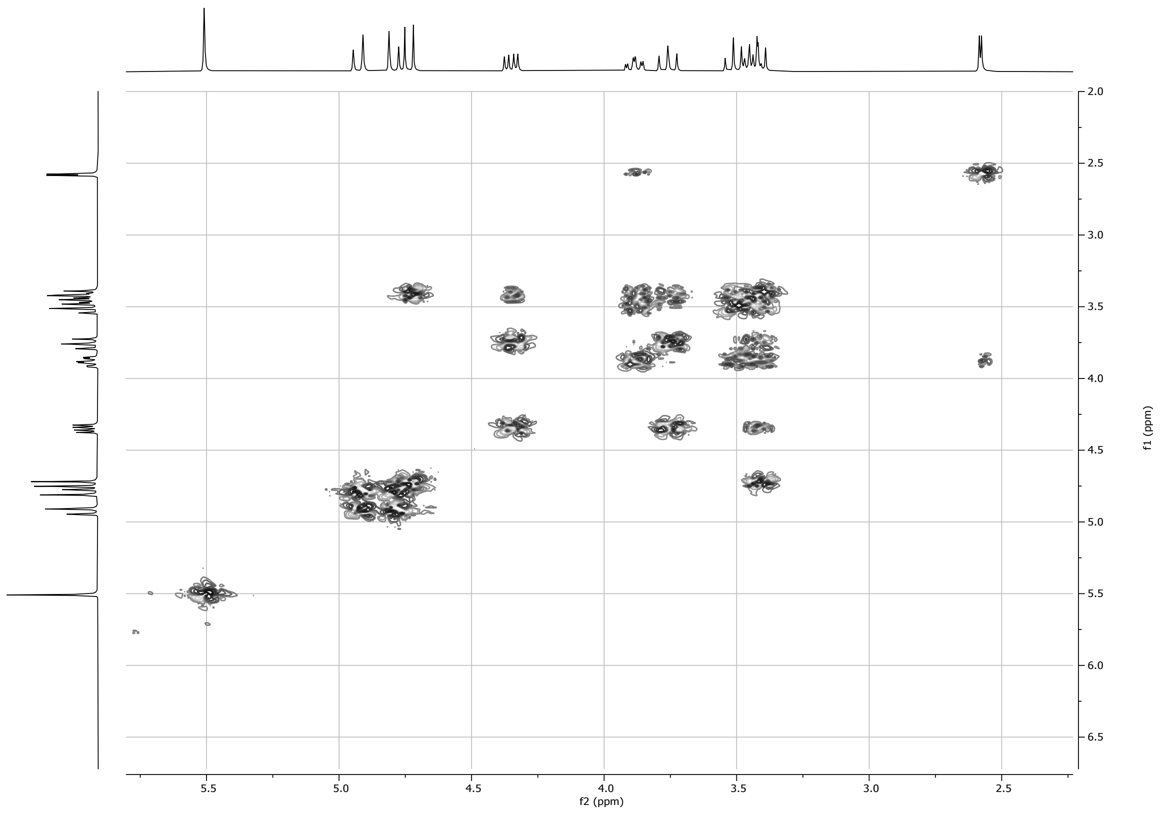

Supplementary Figure S9. HH-COSY NMR, $CDCl_3$ of compound **S1**

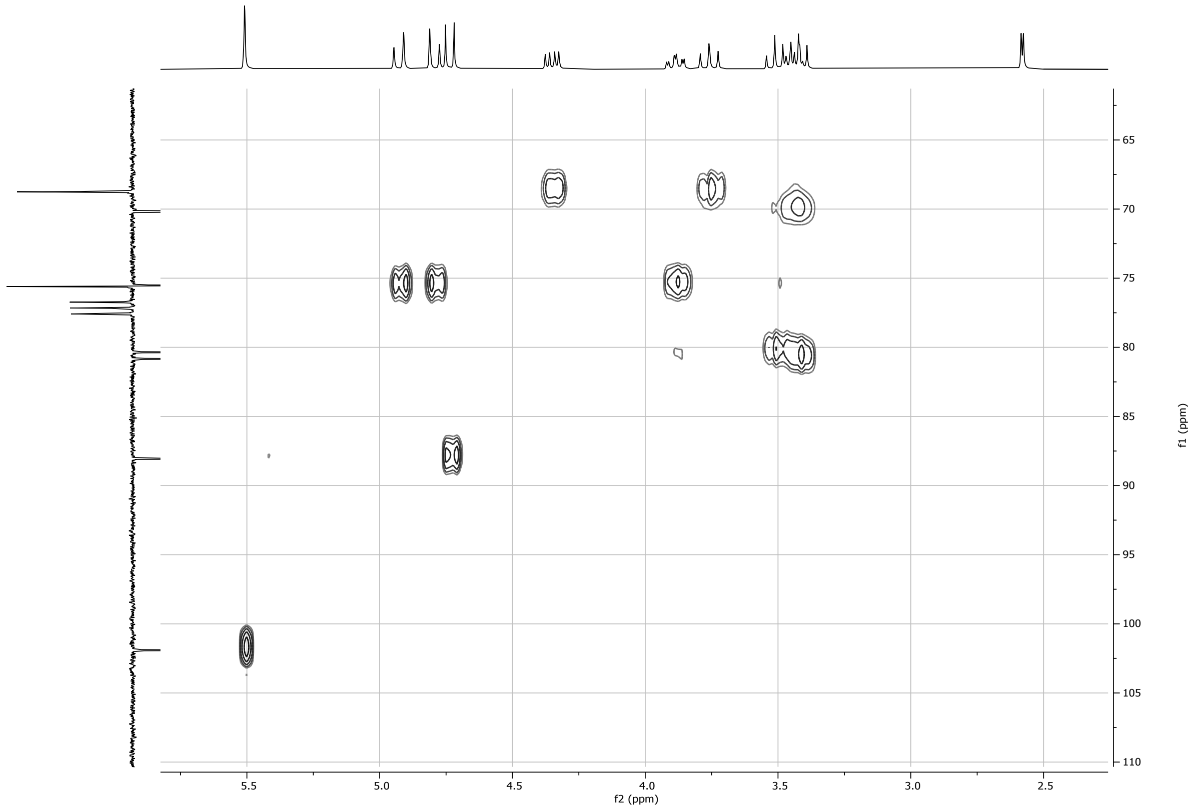

Supplementary Figure S10. HSQC{^{1}H} NMR, $CDCl_3$ of compound **S1**

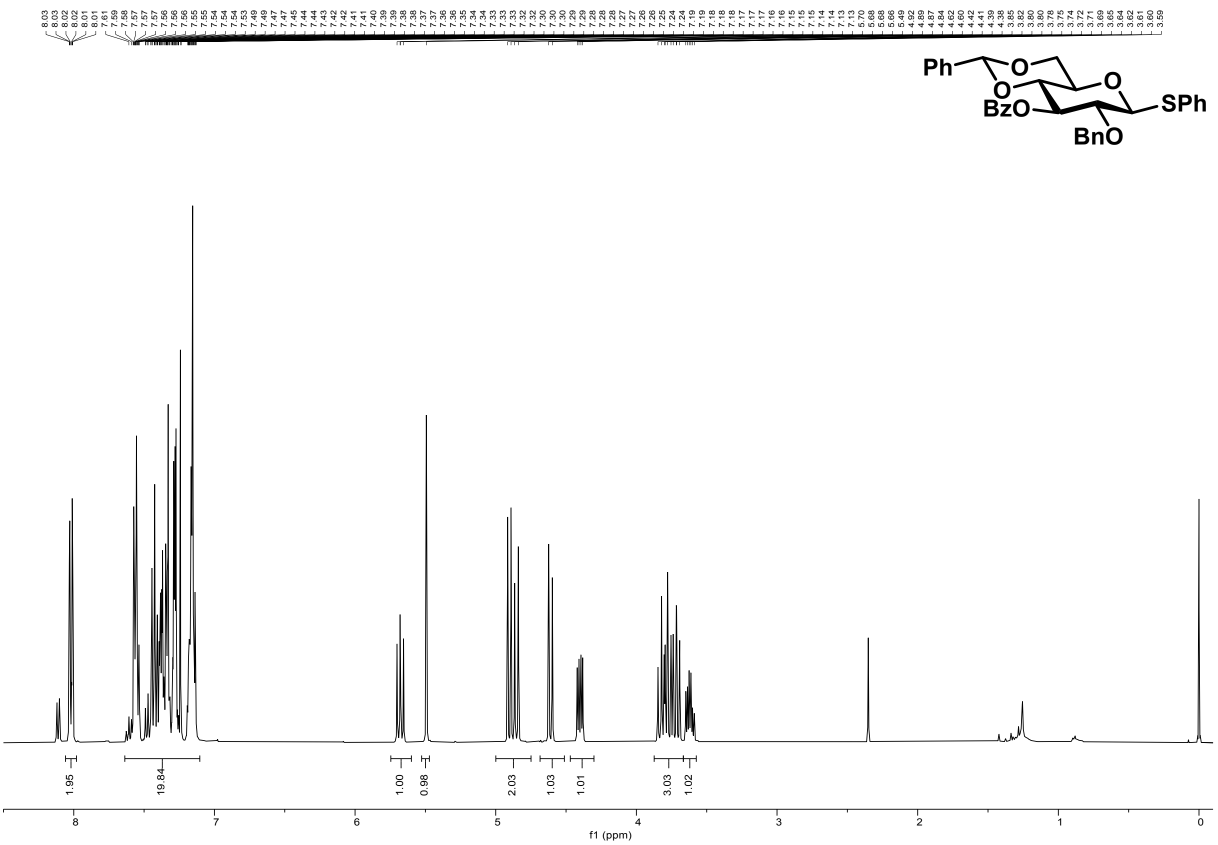

Supplementary Figure S11. ^{1}H NMR, 400 MHz, $CDCl_3$ of compound **2**

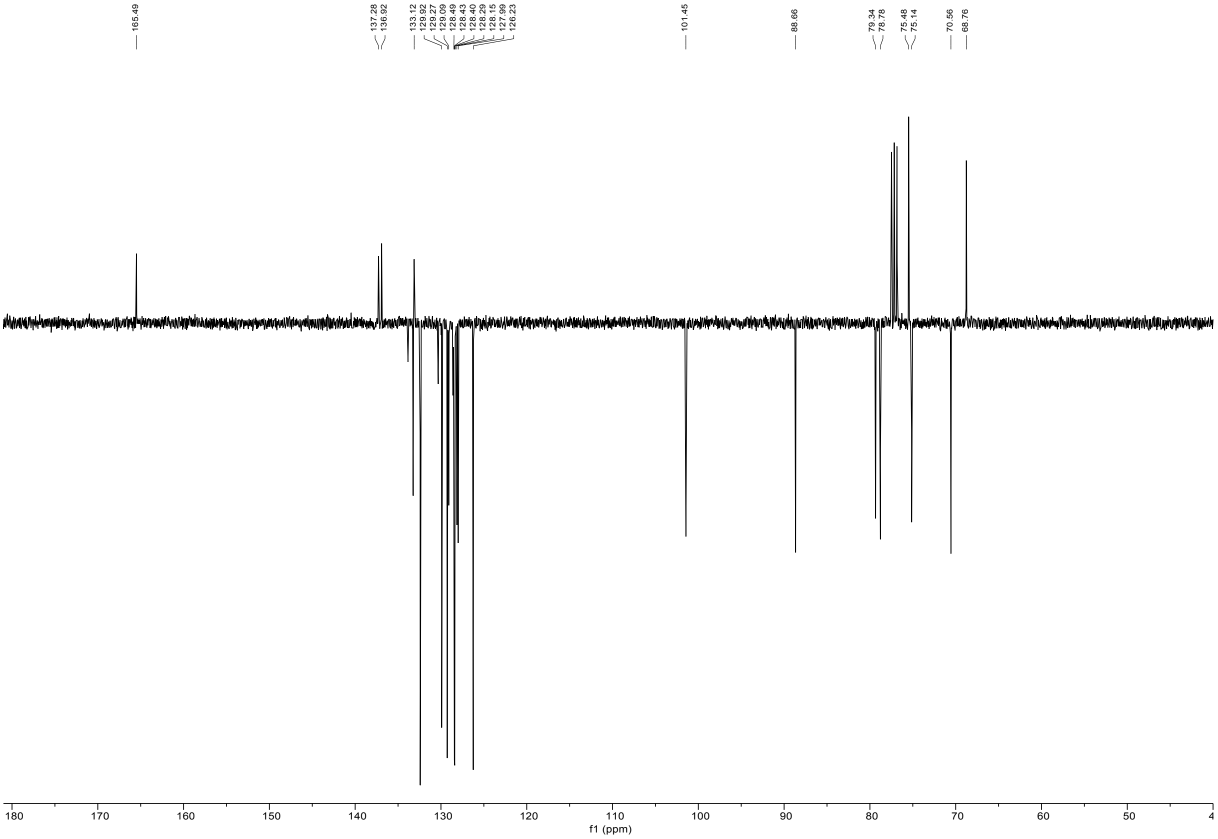

Supplementary Figure S12. $^{13}C\{^1H\}$ NMR, 101 MHz, $CDCl_3$ of compound **2**

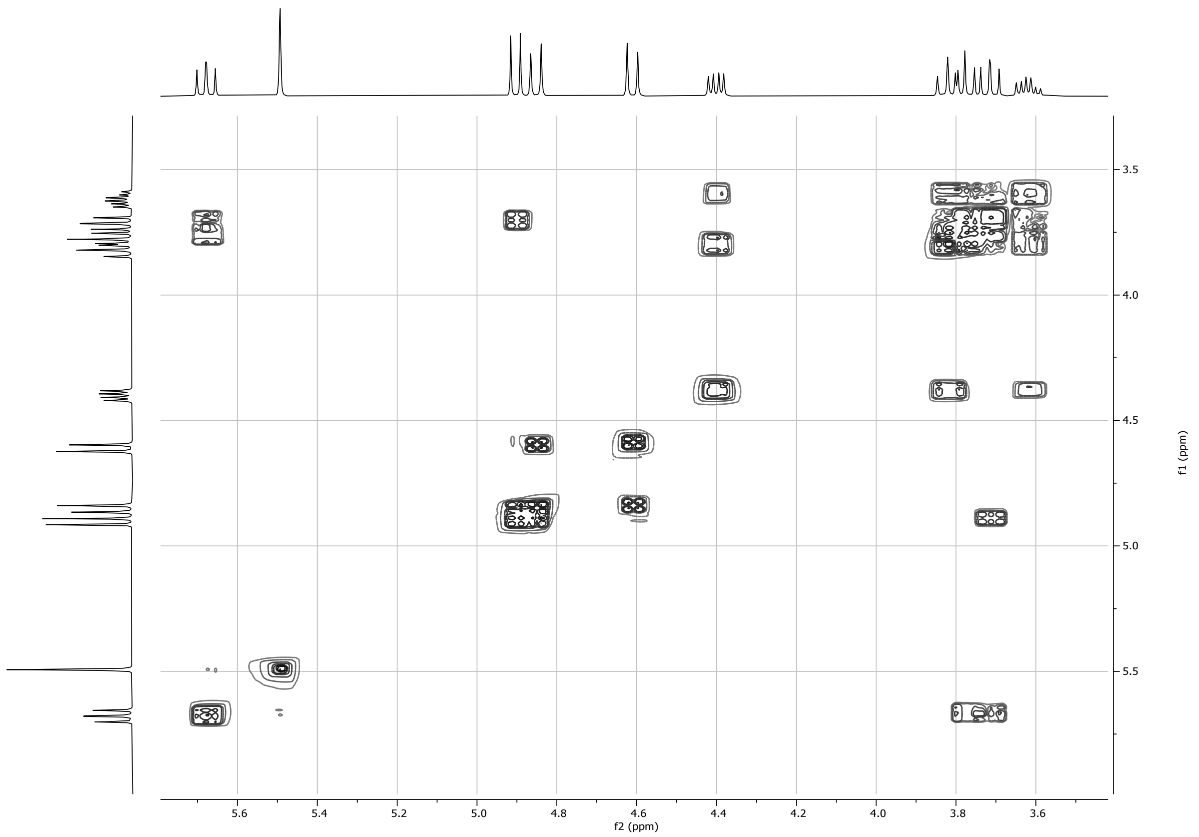

Supplementary Figure S13. HH-COSY NMR, $CDCl_3$ of compound **2**

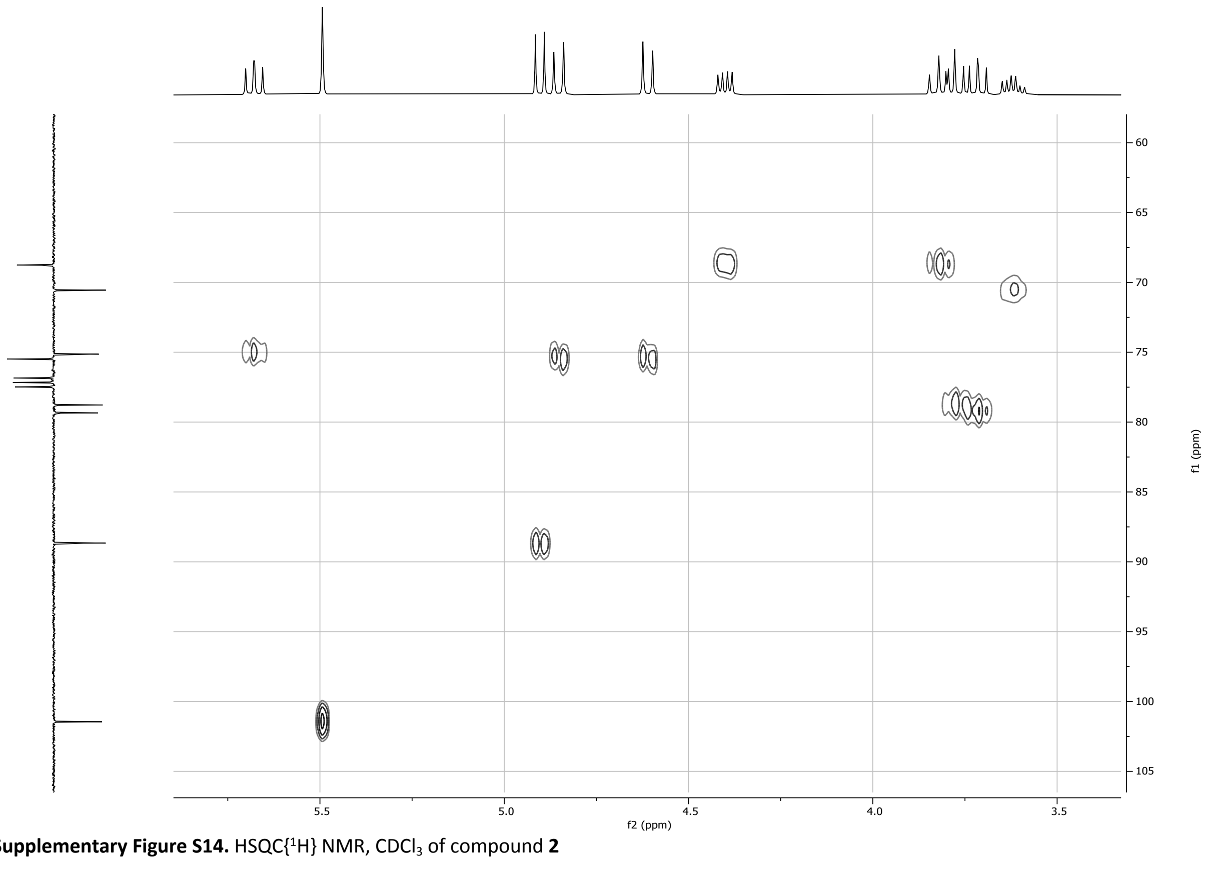

Supplementary Figure S14. HSQC{^{1}H} NMR, $CDCl_3$ of compound **2**

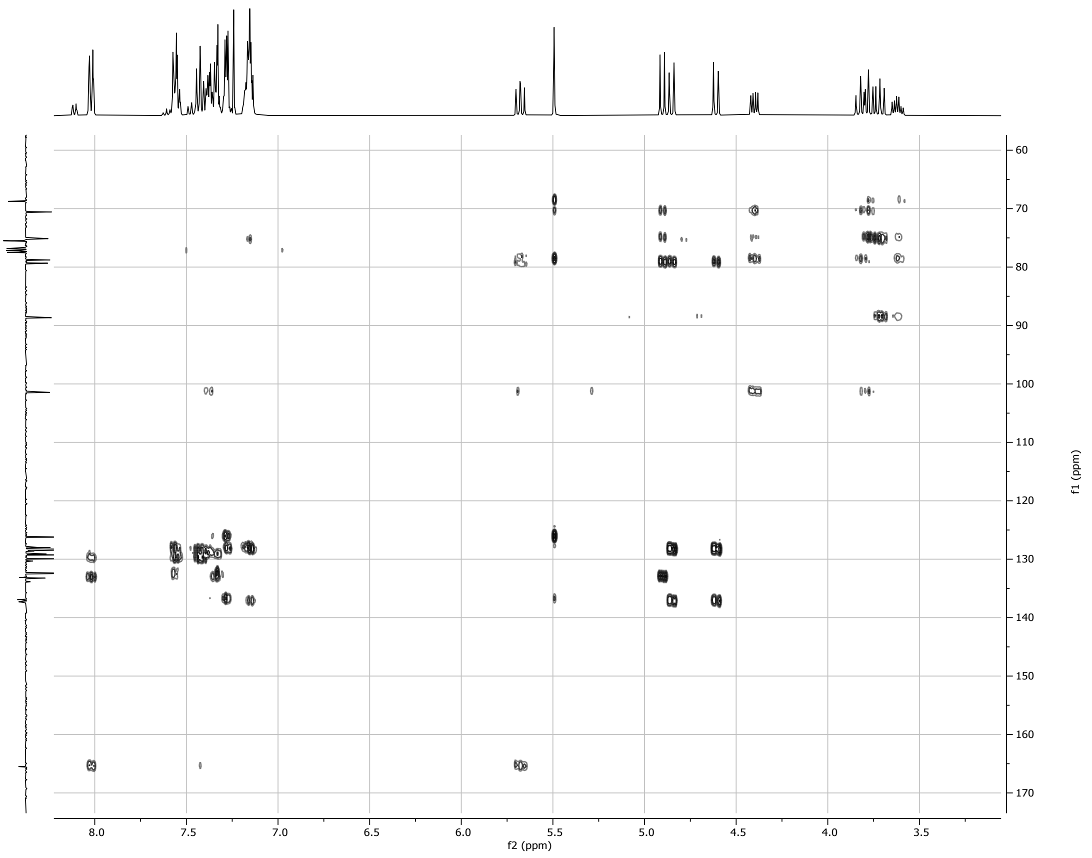

Supplementary Figure S15. HMBC NMR, $CDCl_3$ of compound **2**

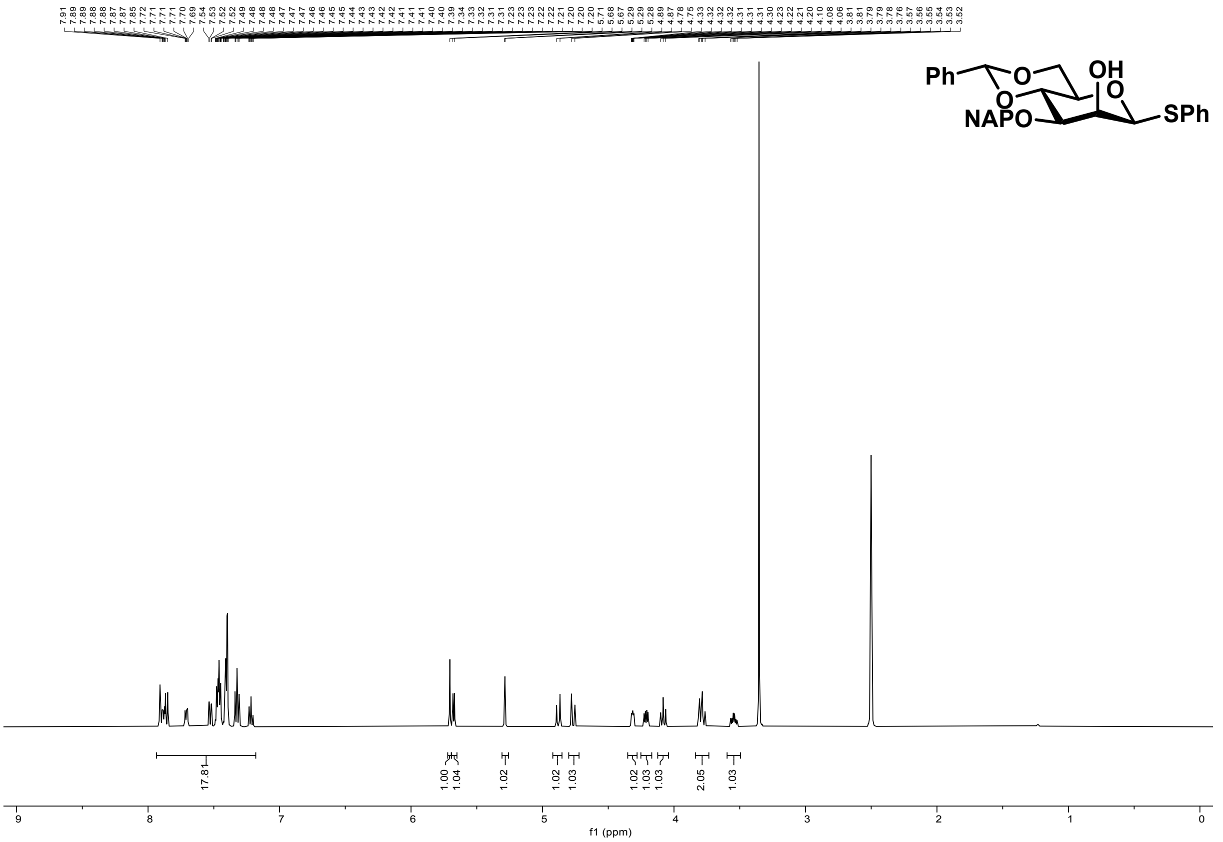

Supplementary Figure 16. ^{1}H NMR, 500 MHz, $(CD_3)_2SO$ of compound **S2**

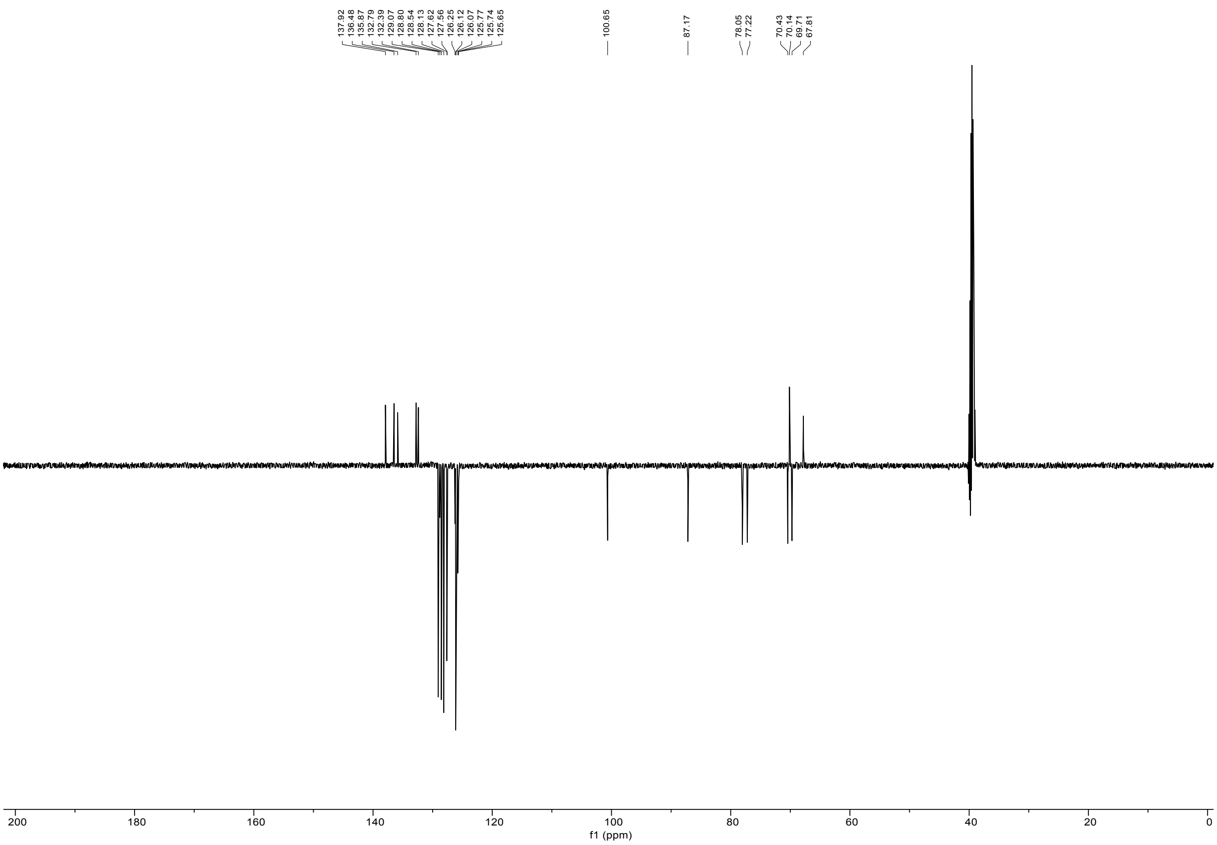

Supplementary Figure 17. $^{13}C\{^{1}H\}$ NMR, 126 MHz, $(CD_3)_2SO$ of compound **S2**

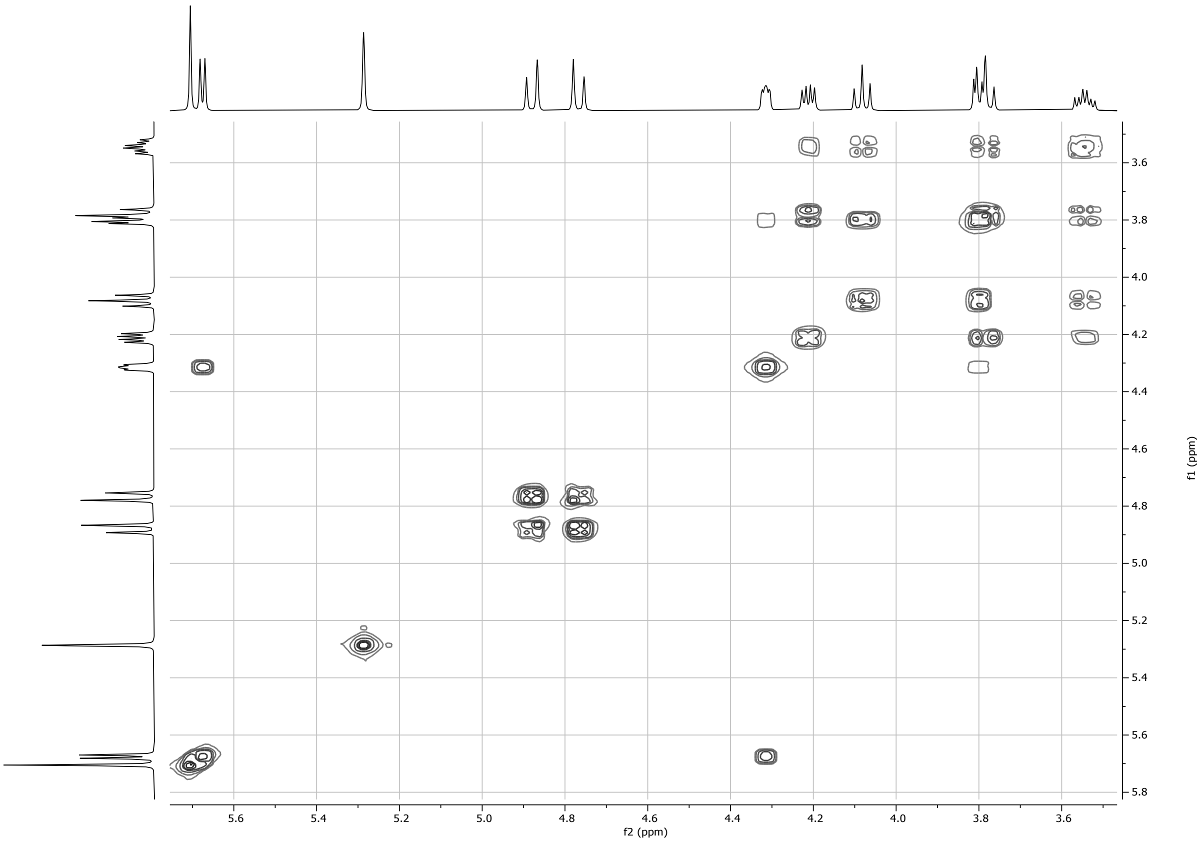

Supplementary Figure 18. HH-COSY NMR, $(CD_3)_2SO$ of compound **S2**

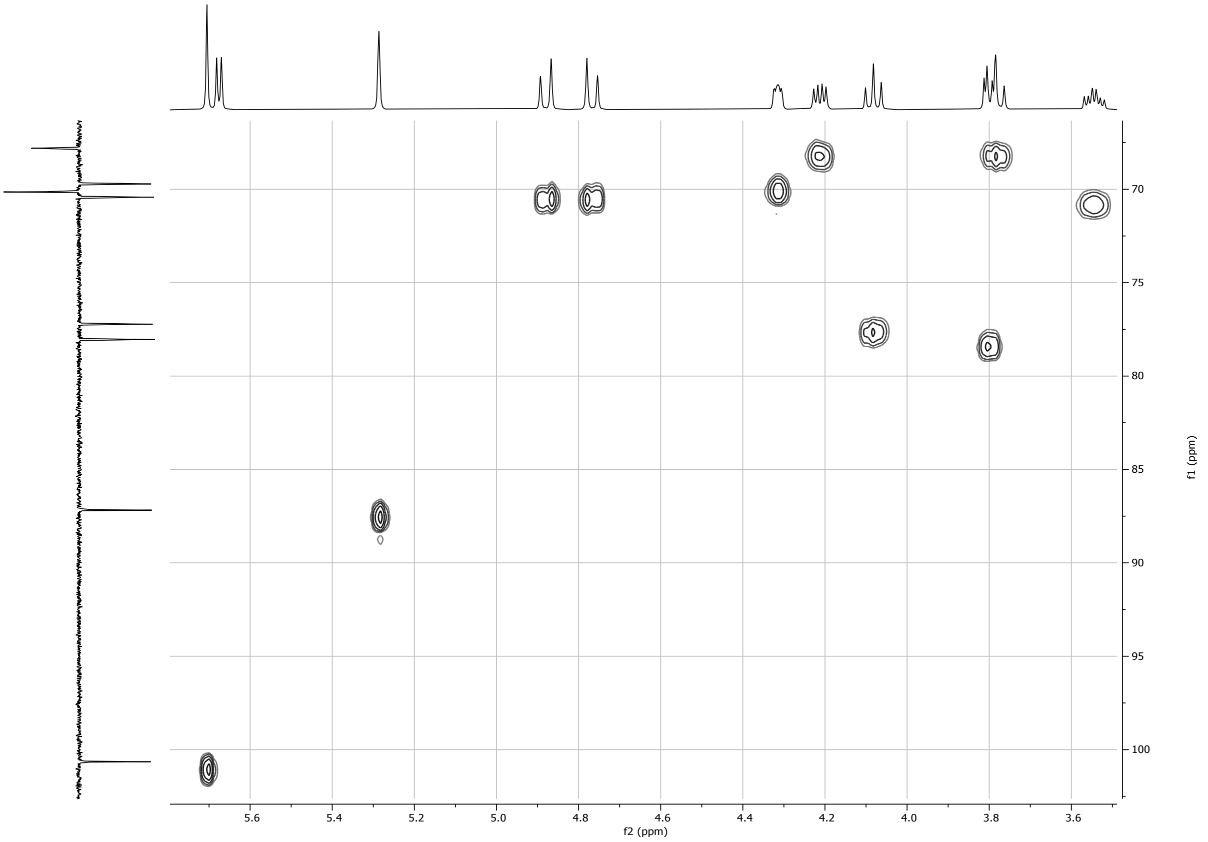

Supplementary Figure 19. HSQC{^{1}H} NMR, $(CD_3)_2SO$ of compound **S2**

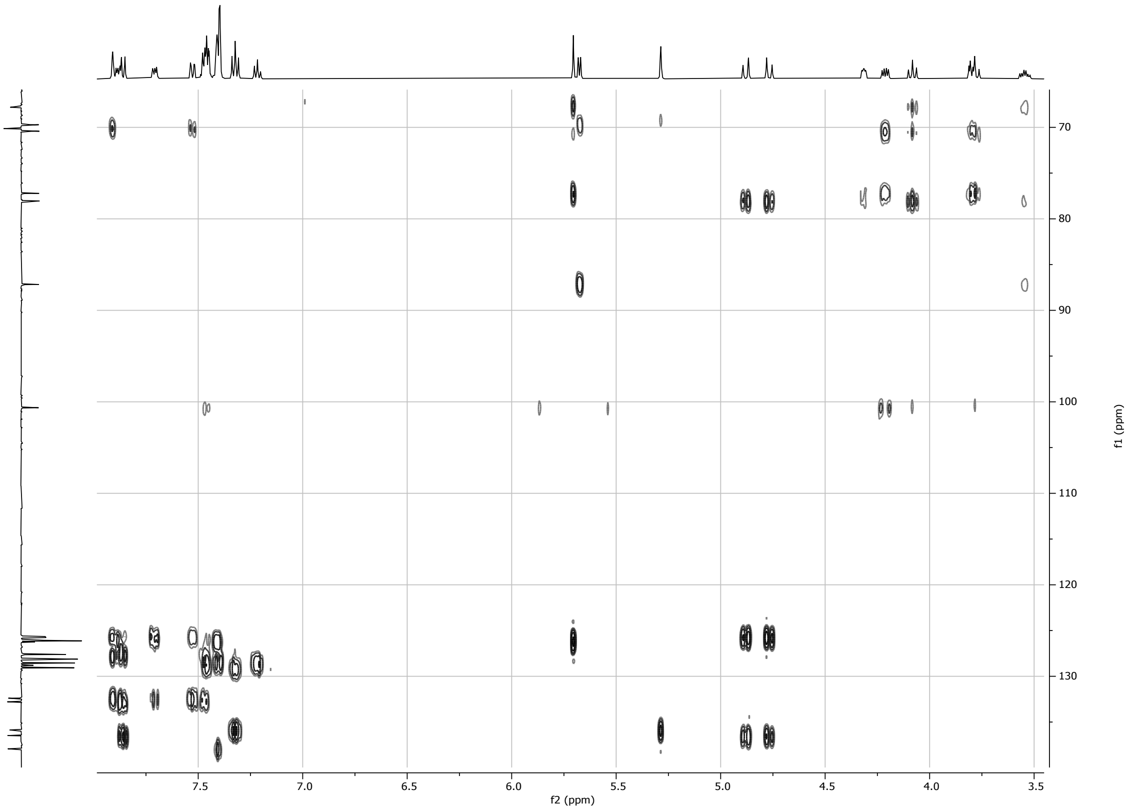

Supplementary Figure 20. HMBC NMR, $(CD_3)_2SO$ of compound **S2**

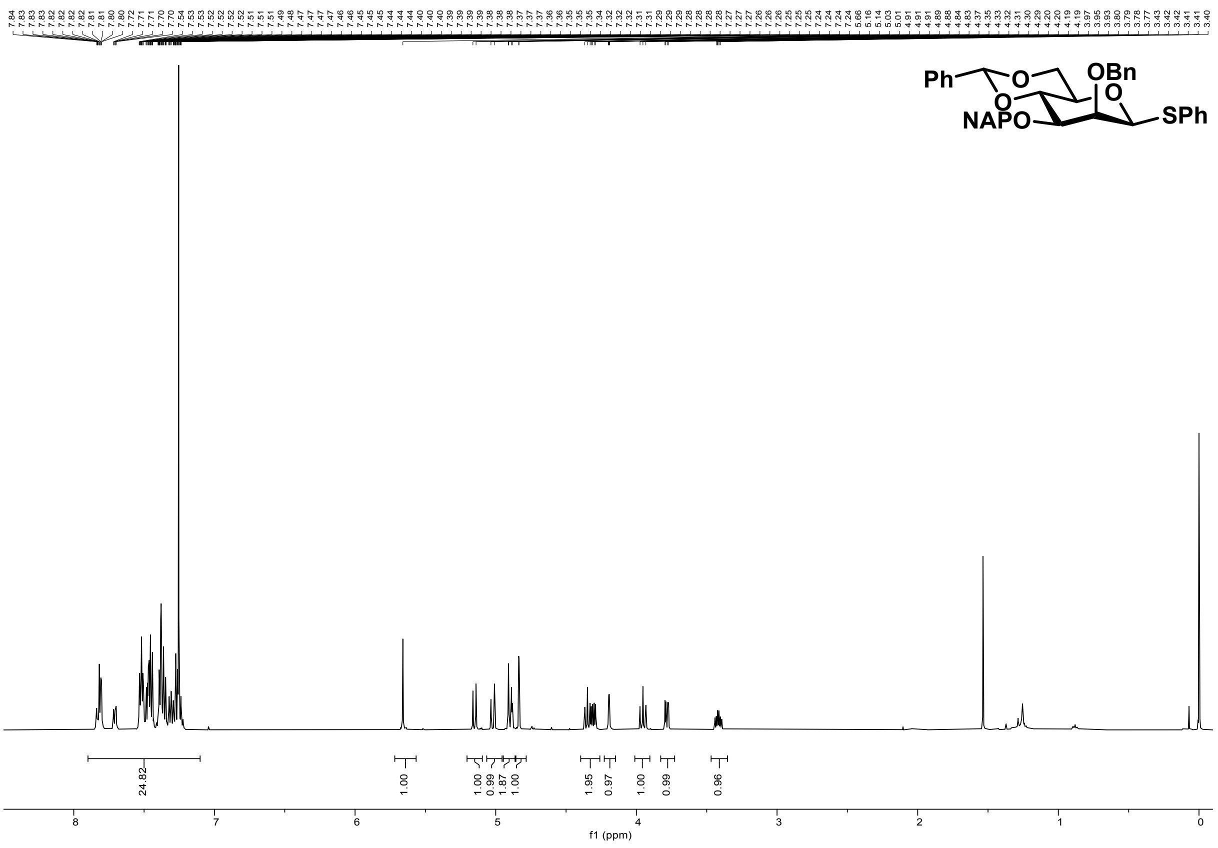

Supplementary Figure S21. ^{1}H NMR, 400 MHz, $CDCl_3$ of compound **S3**

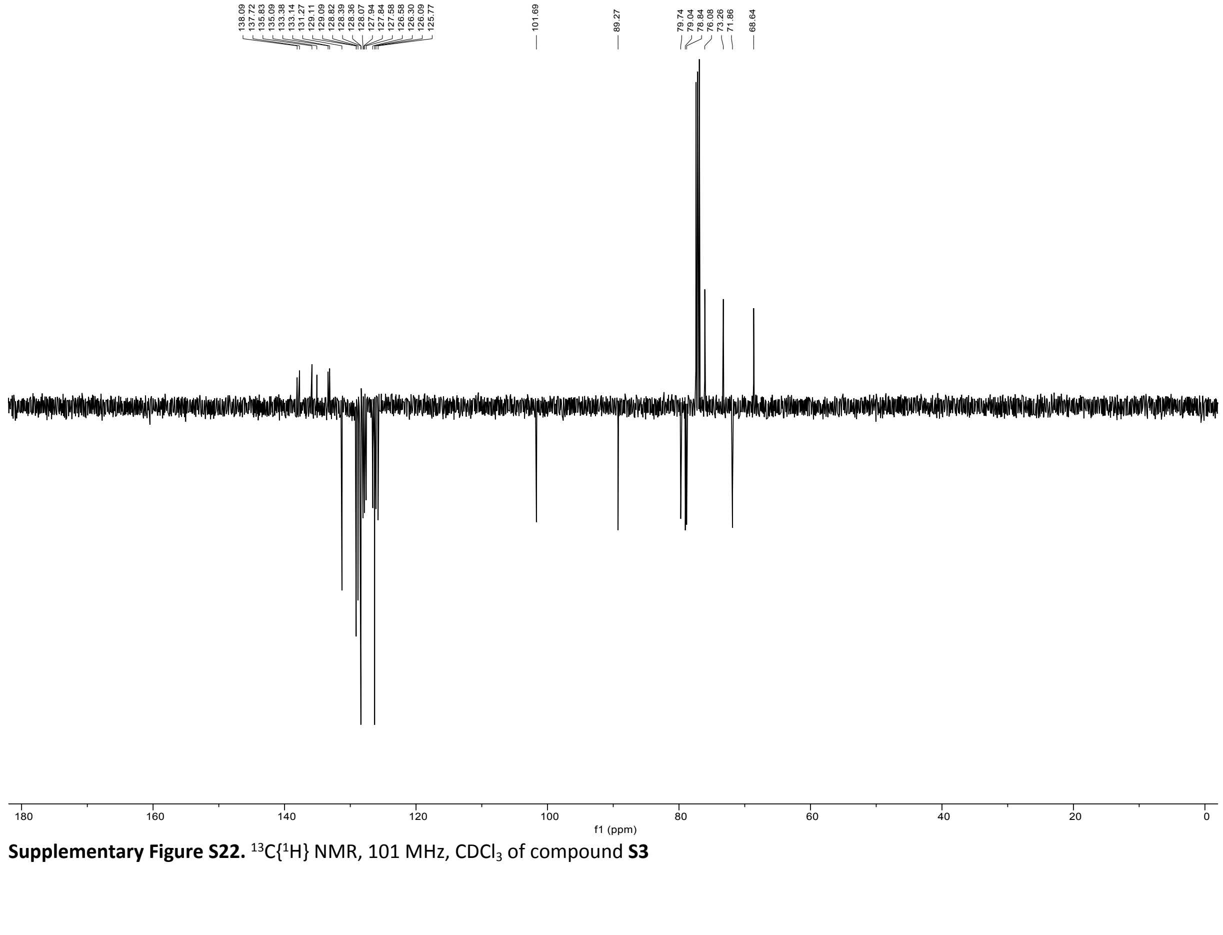

Supplementary Figure S22. $^{13}C\{^{1}H\}$ NMR, 101 MHz, $CDCl_3$ of compound **S3**

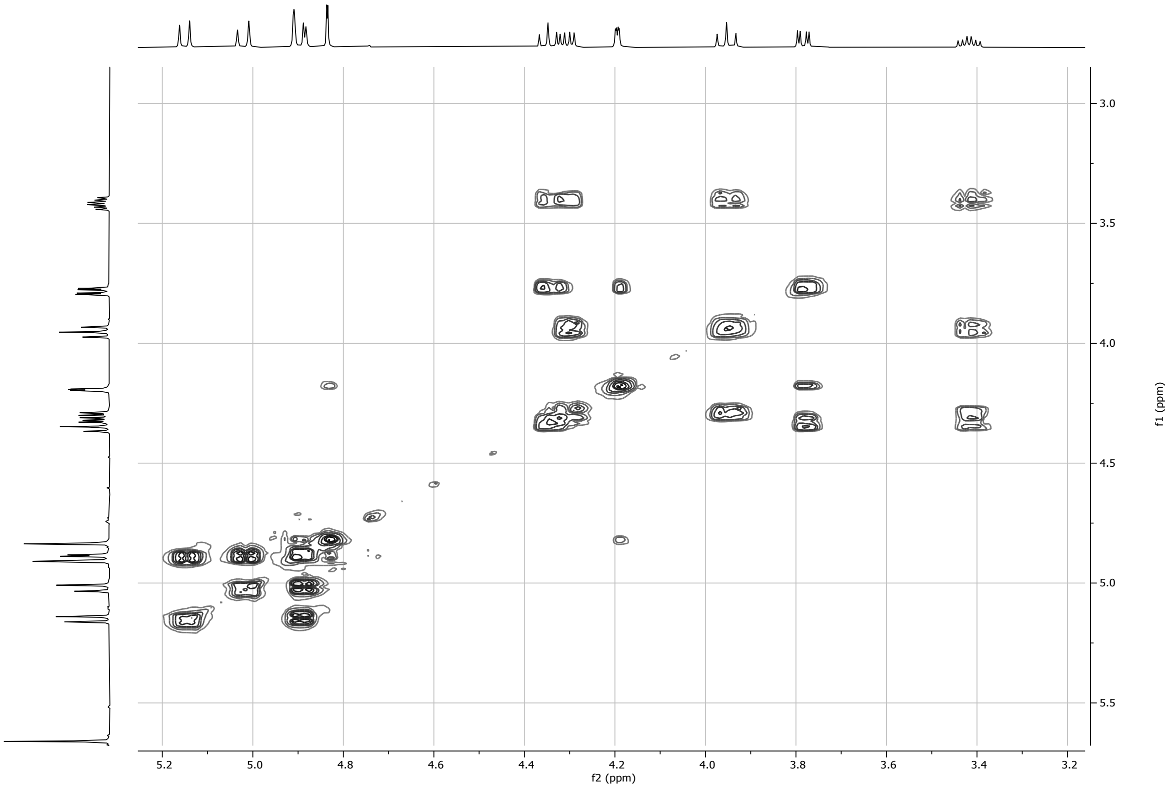

Supplementary Figure S23. HH-COSY NMR, $CDCl_3$ of compound **S3**

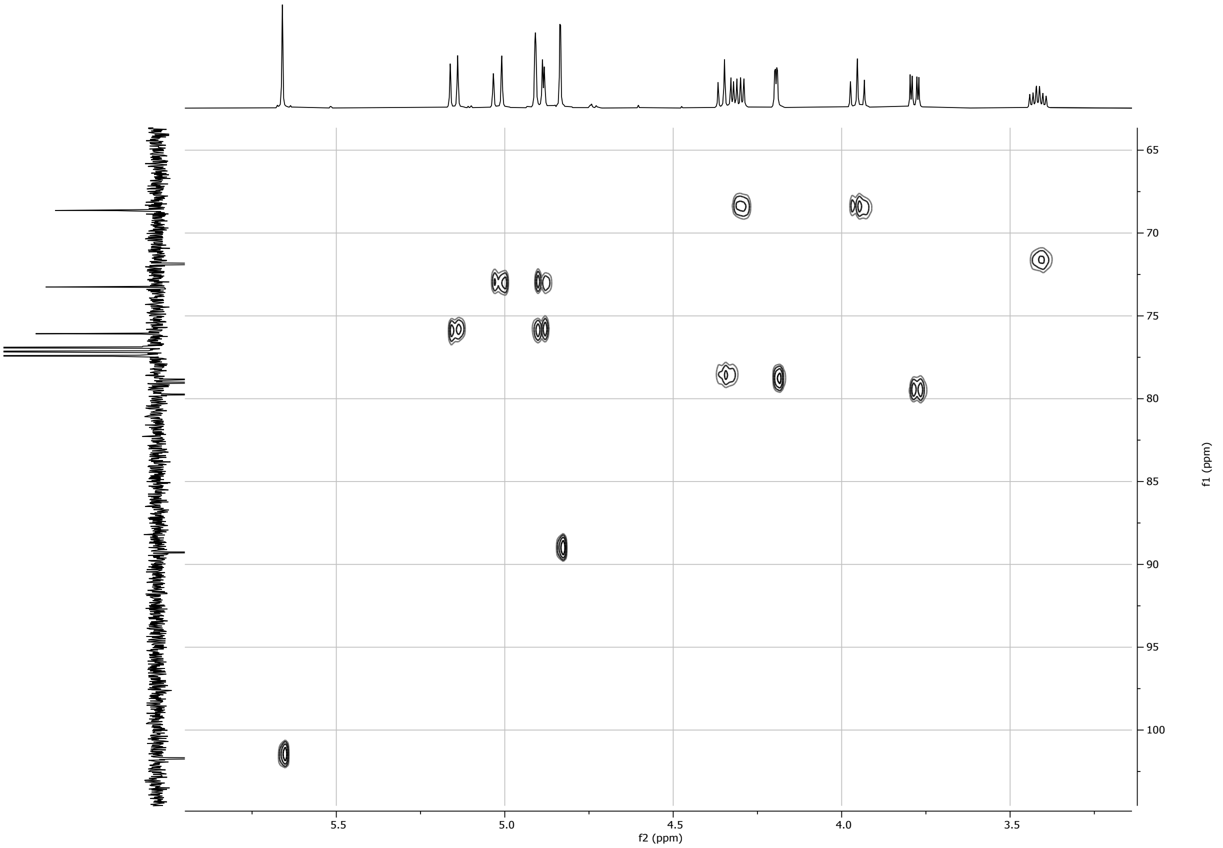

Supplementary Figure S24. HSQC{^{1}H} NMR, $CDCl_3$ of compound **S3**

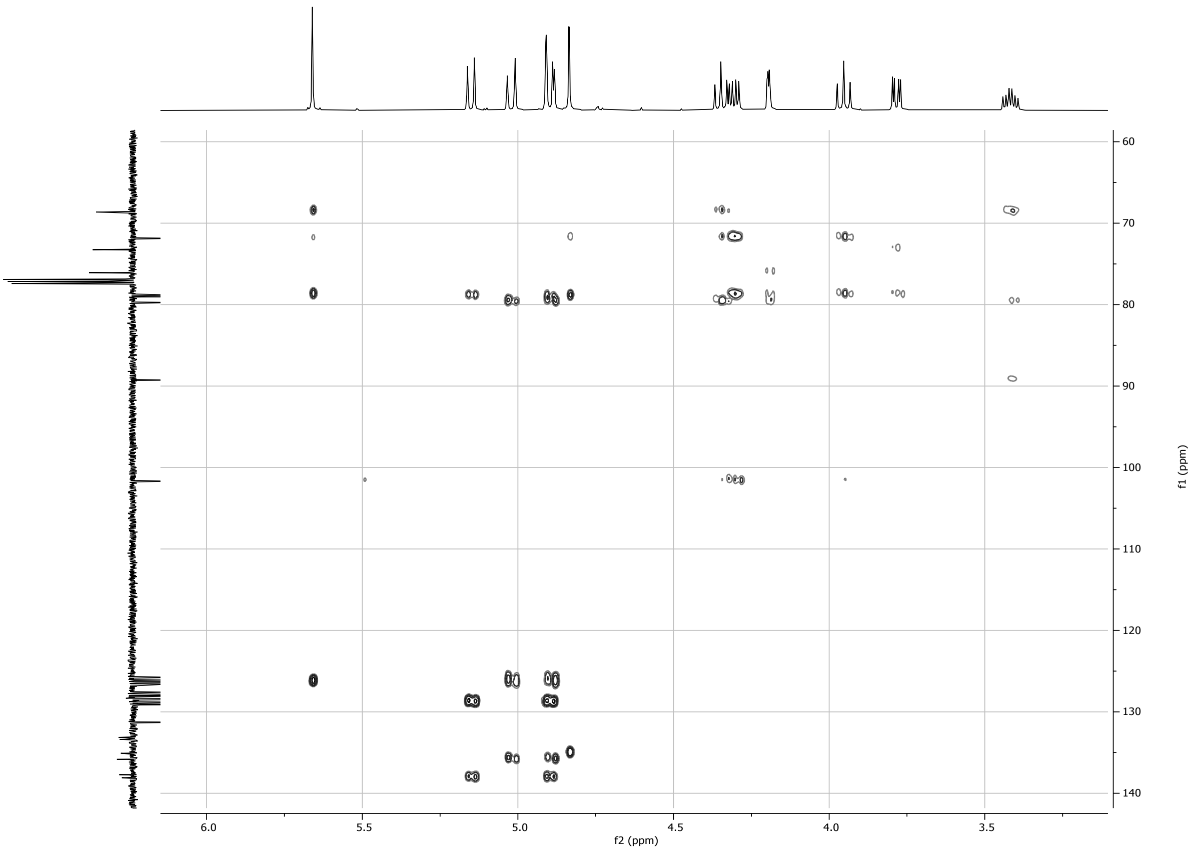

Supplementary Figure S25. HMBC NMR, $CDCl_3$ of compound **S3**

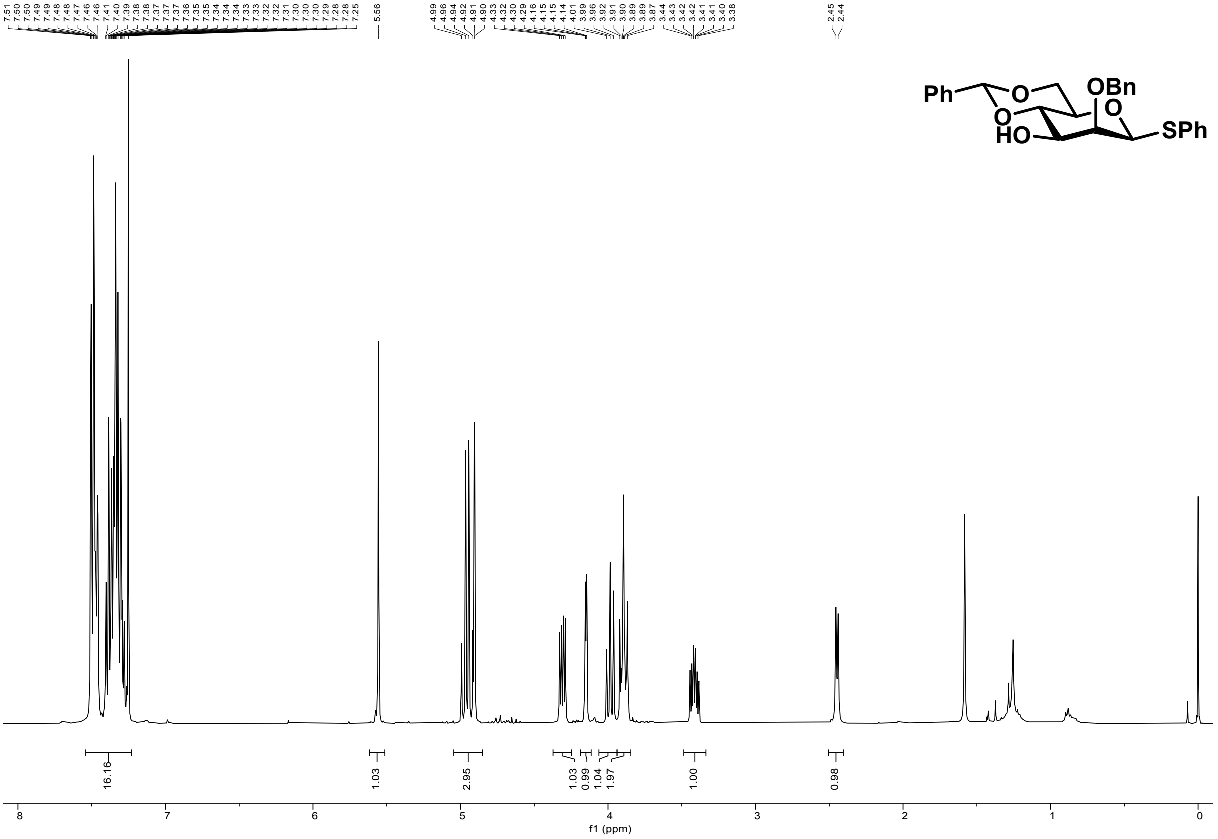

Supplementary Figure S26. ^{1}H NMR, 400 MHz, $CDCl_3$ of compound **S4**

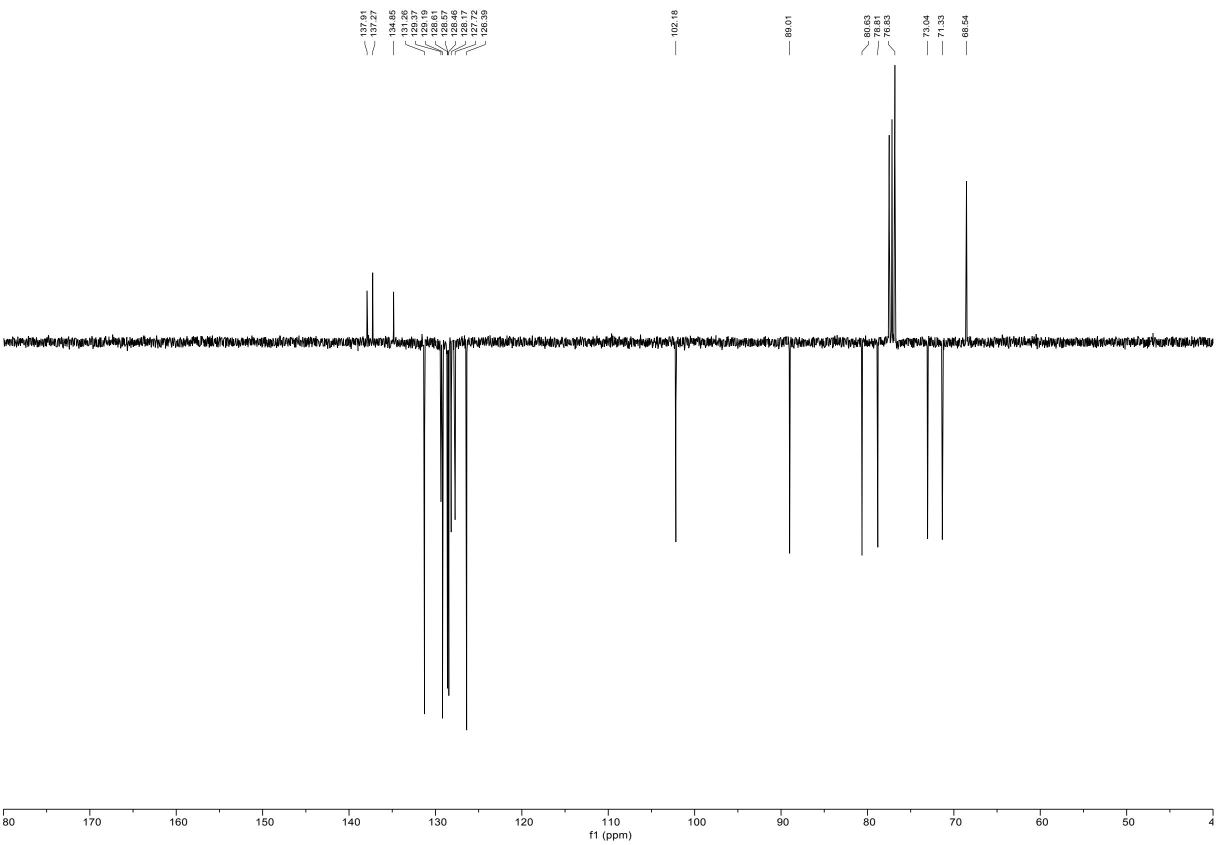

Supplementary Figure S27. $^{13}C\{^{1}H\}$ NMR, 101 MHz, $CDCl_3$ of compound **S4**

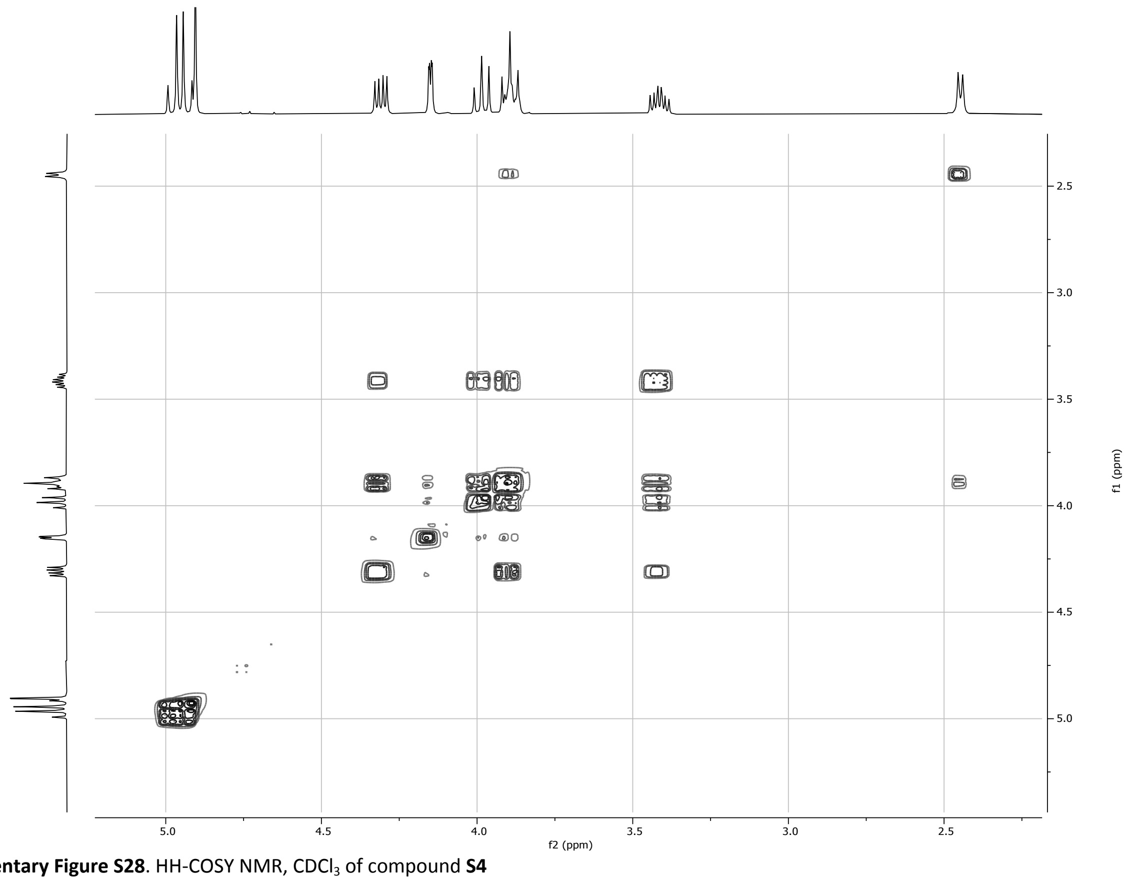

Supplementary Figure S28. HH-COSY NMR, $CDCl_3$ of compound **S4**

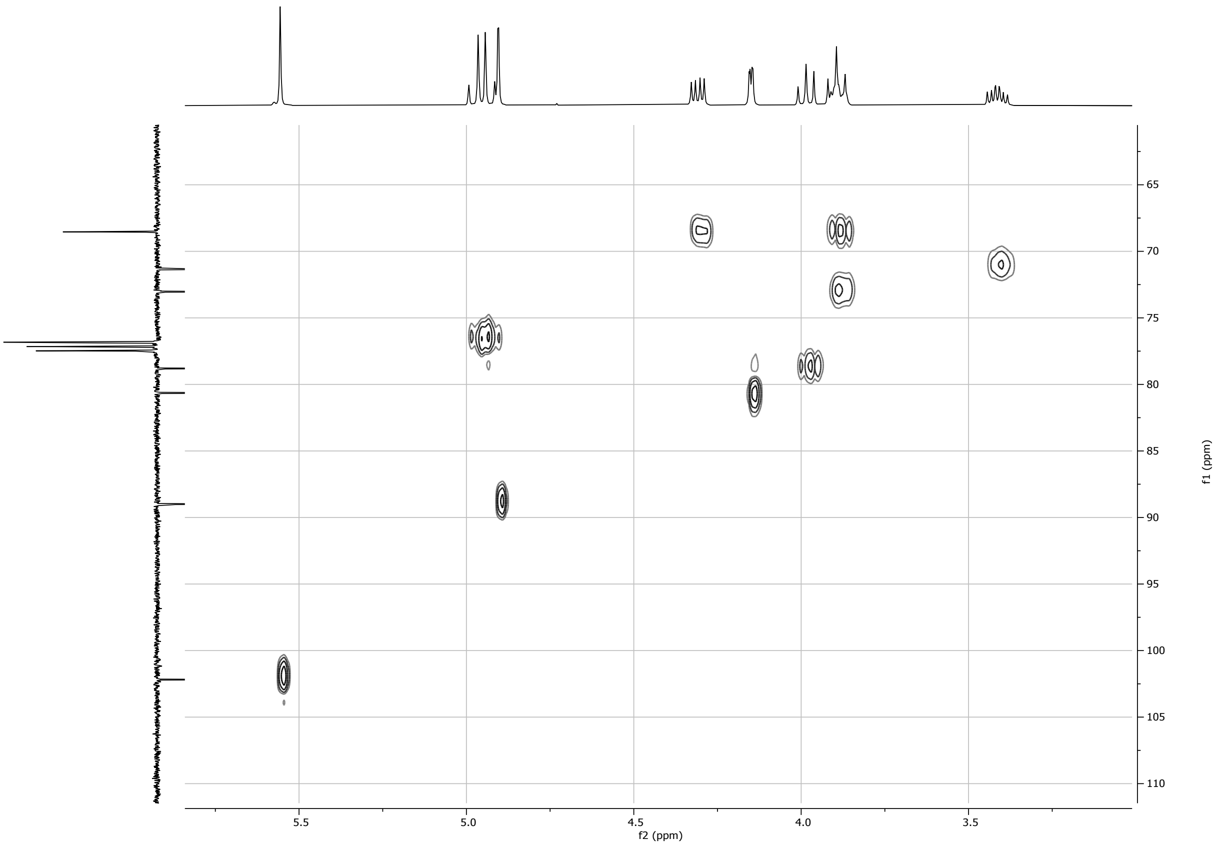

Supplementary Figure S29. HSQC{^{1}H} NMR, $CDCl_3$ of compound **S4**

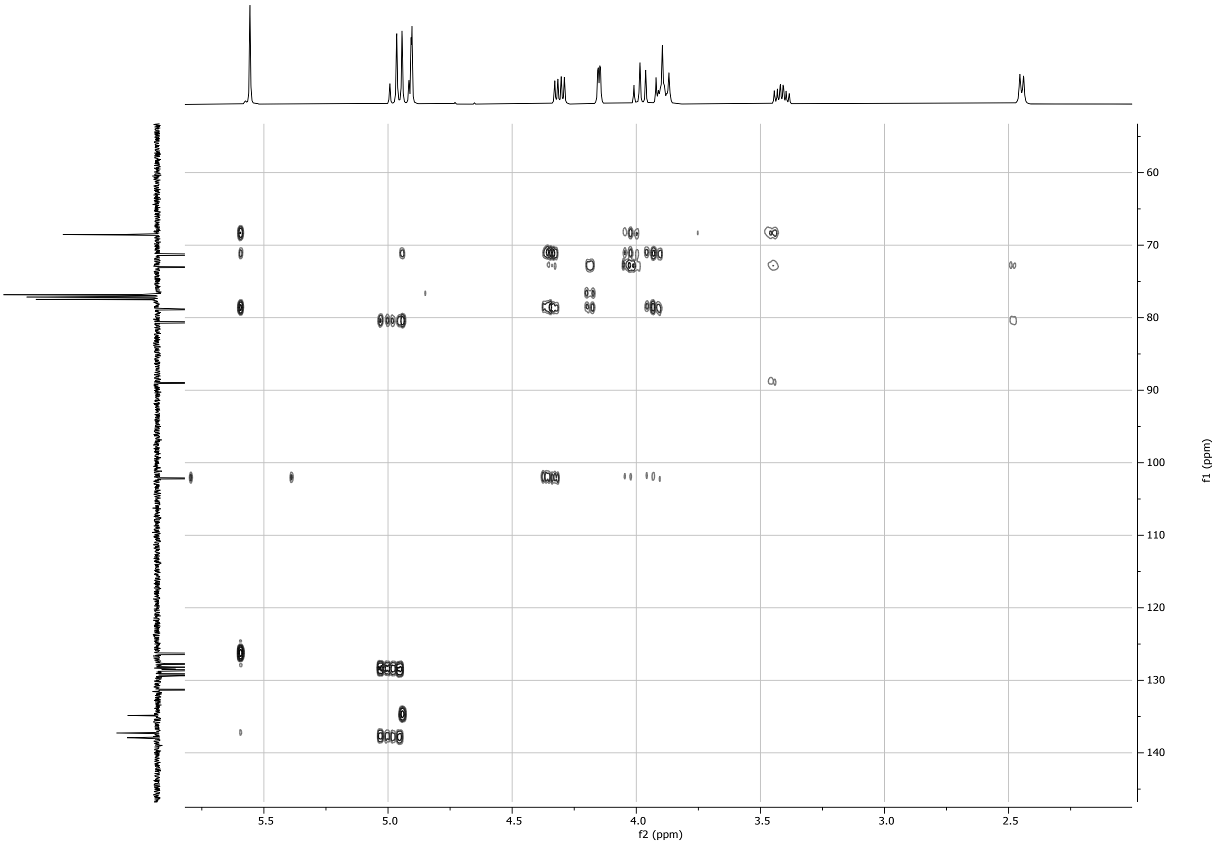

Supplementary Figure S30. HMBC NMR, $CDCl_3$ of compound **S4**

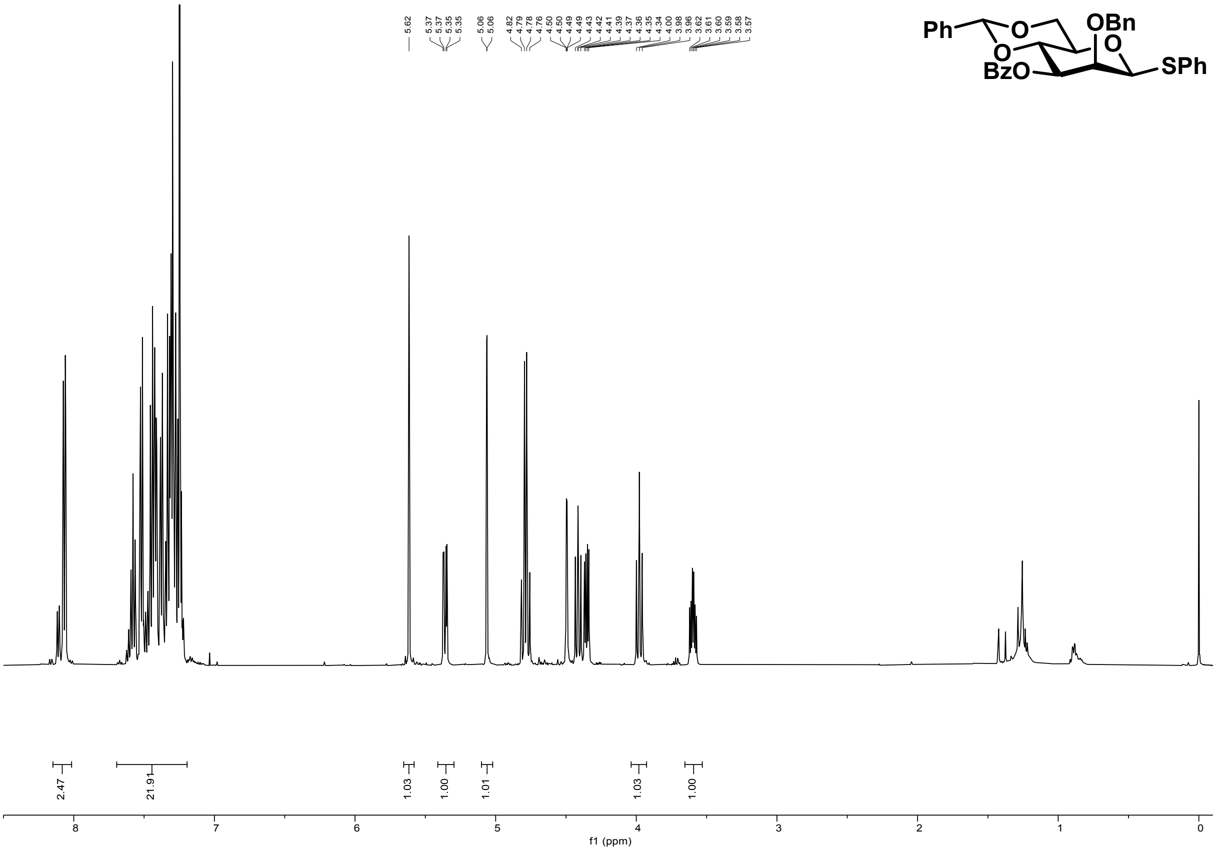

Supplementary Figure S31. ^{1}H NMR, 400 MHz, $CDCl_3$ of compound **4**

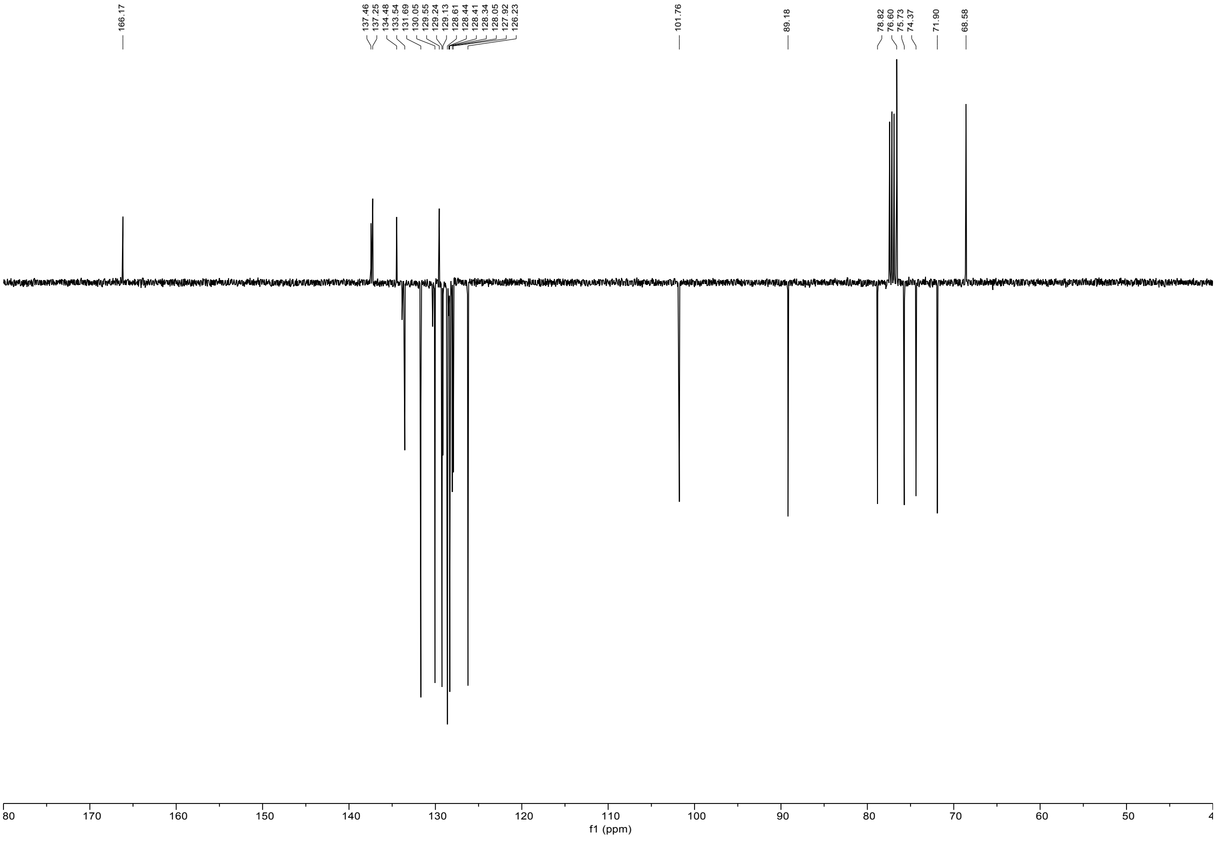

Supplementary Figure S32. $^{13}C\{^{1}H\}$ NMR, 101 MHz, $CDCl_3$ of compound **4**

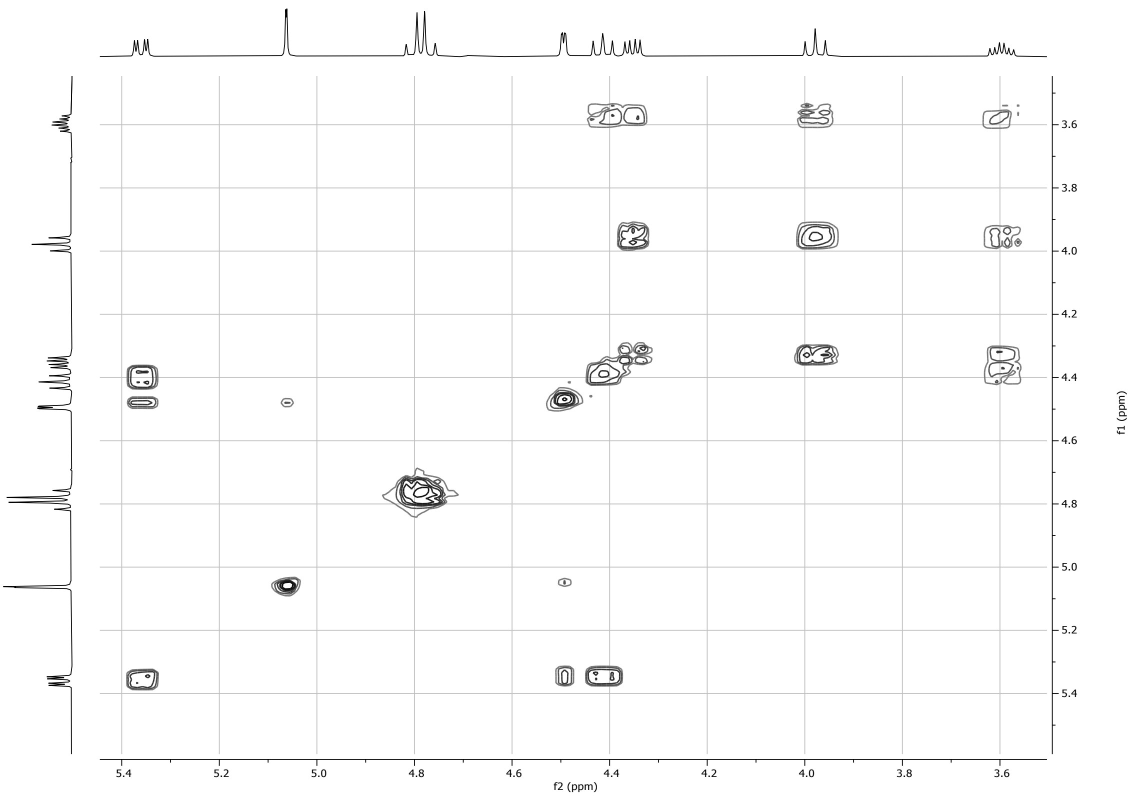

Supplementary Figure S33. HH-COSY NMR, $CDCl_3$ of compound **4**

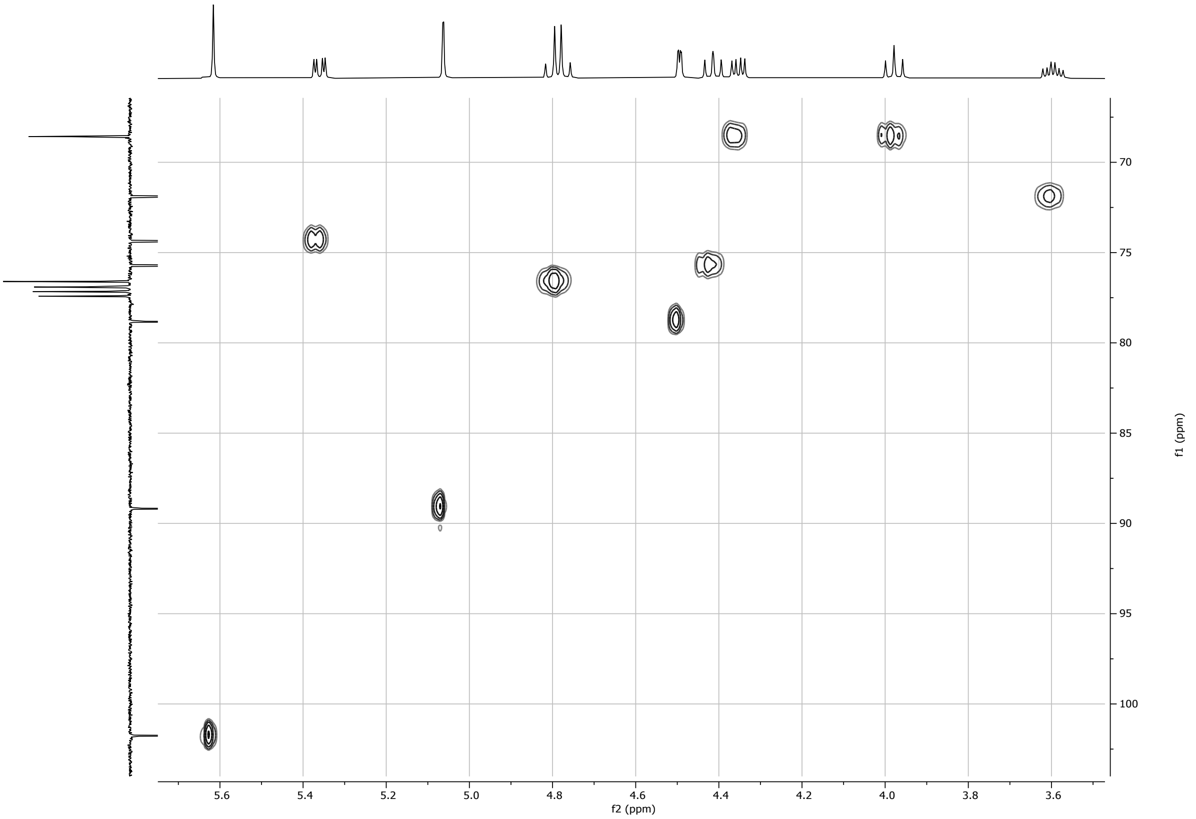

Supplementary Figure S34. HSQC{^{1}H} NMR, $CDCl_3$ of compound **4**

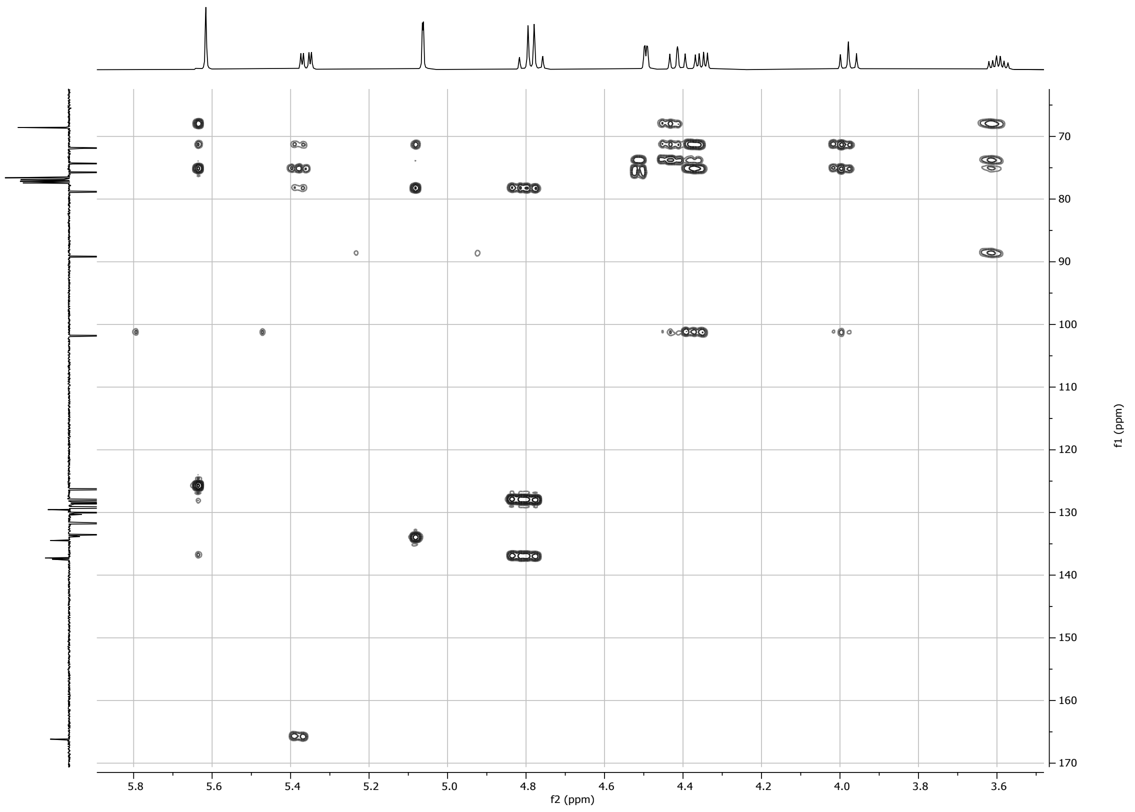

Supplementary Figure S35. HMBC NMR, $CDCl_3$ of compound **4**

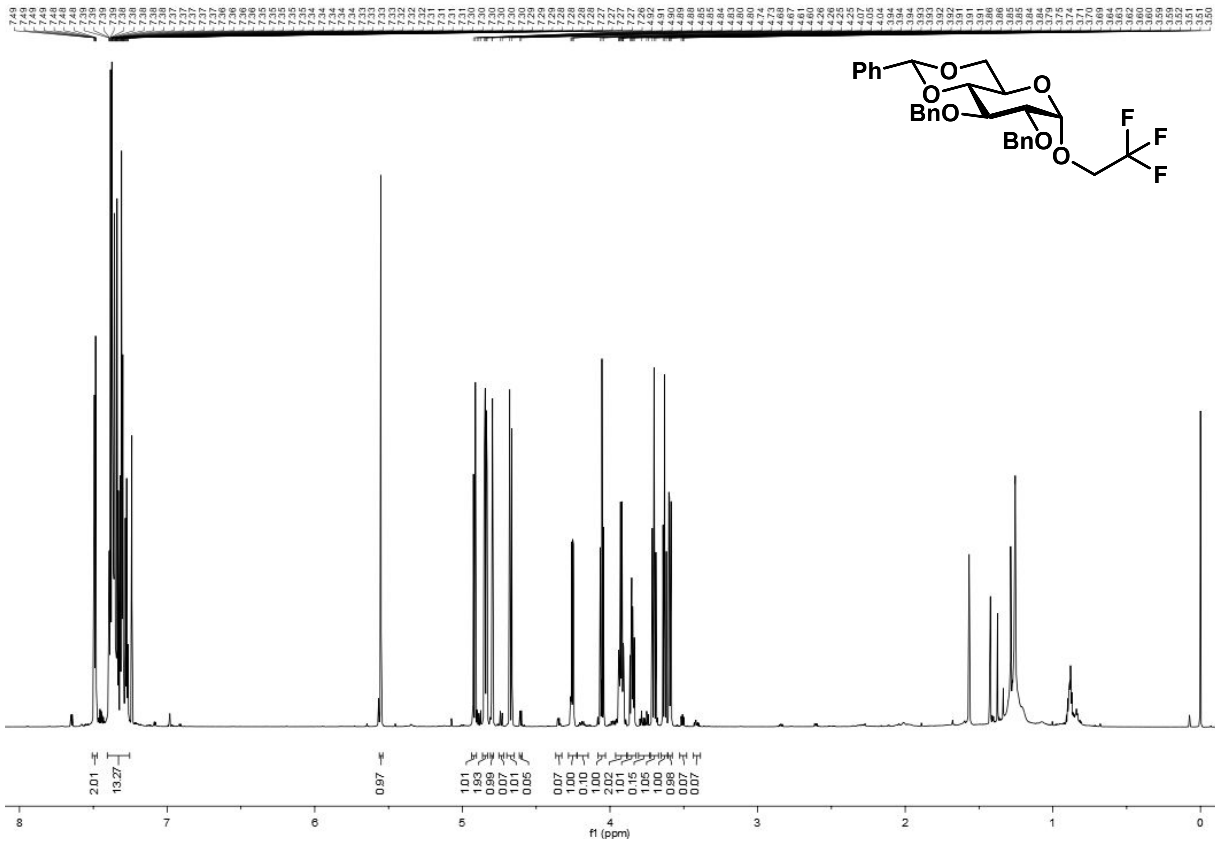

Supplementary Figure S36. ^{1}H NMR, 850 MHz, $CDCl_3$ of compound **S5**

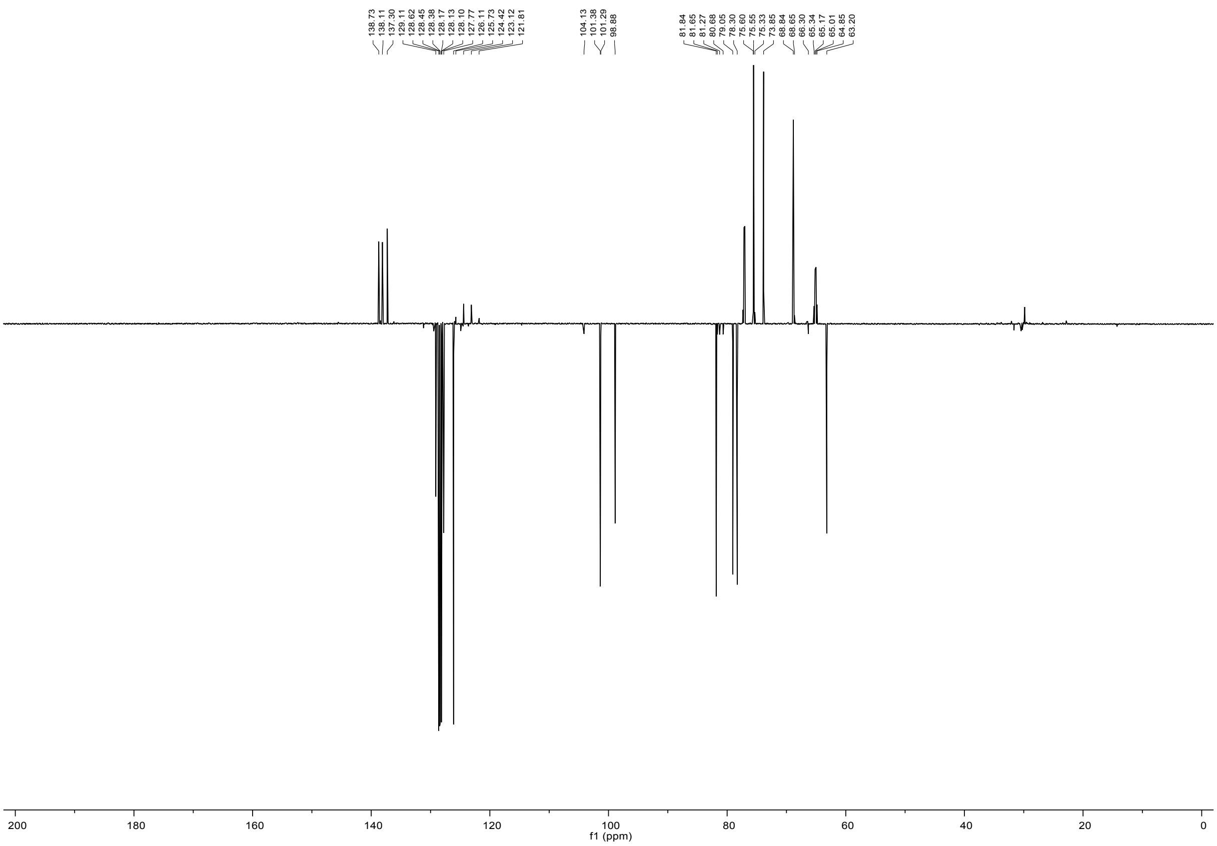

Supplementary Figure S37. $^{13}C\{^{1}H\}$ NMR, 214 MHz, $CDCl_3$ of compound **S5**

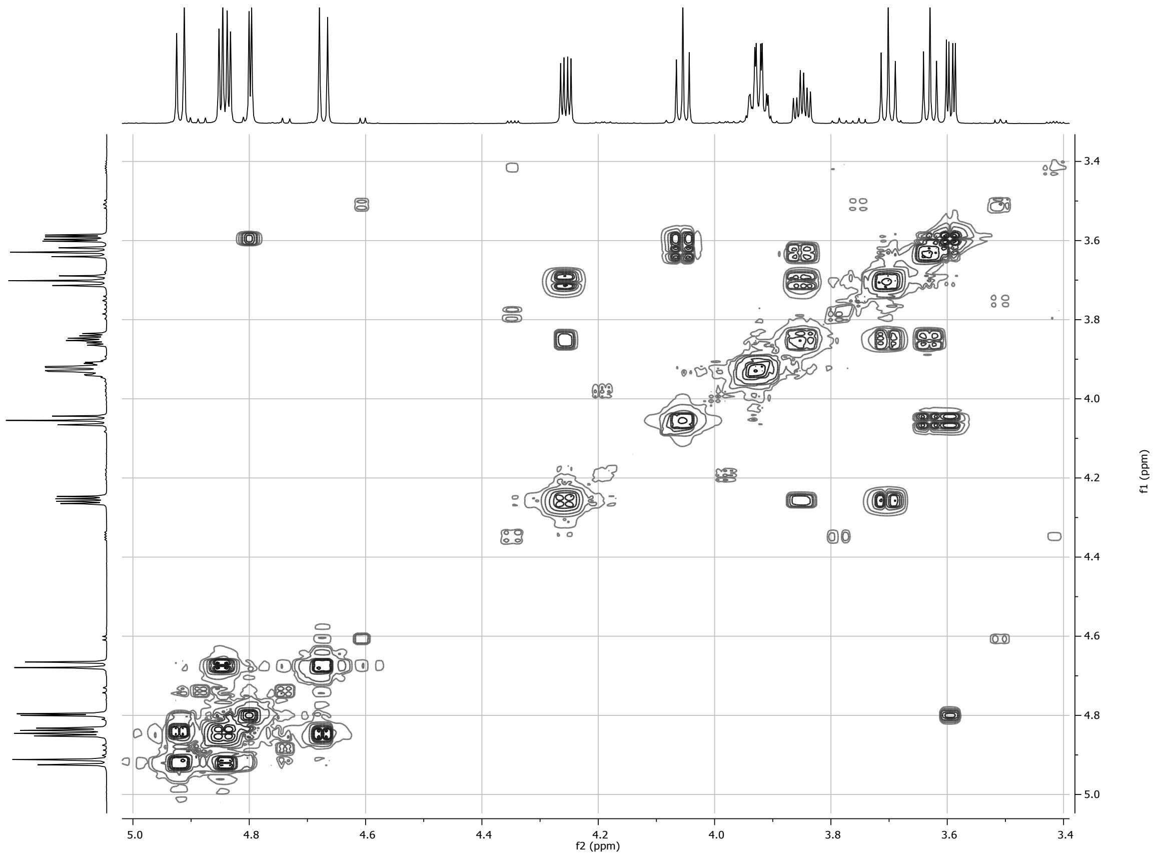

Supplementary Figure S38. HH-COSY NMR, $CDCl_3$ of compound **S5**

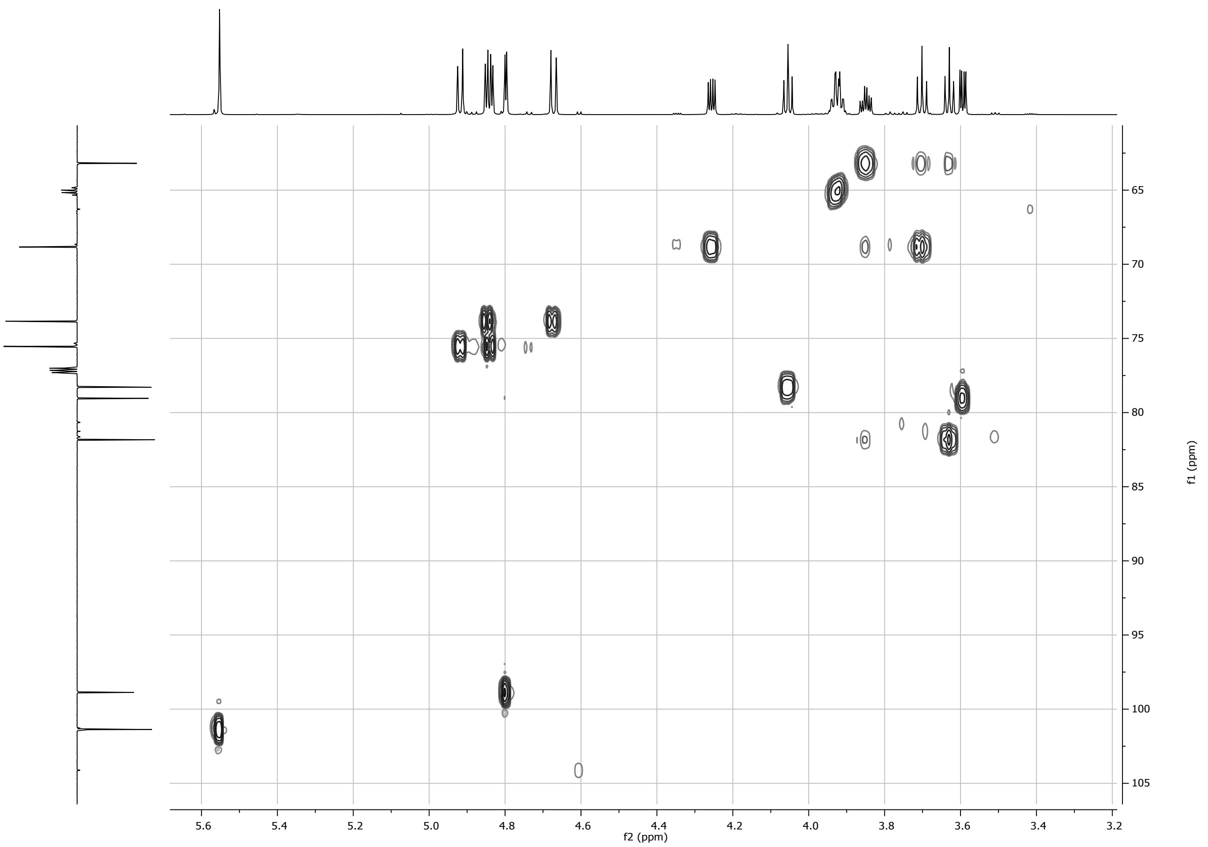

Supplementary Figure S39. HSQC{^{1}H} NMR, $CDCl_3$ of compound **S5**

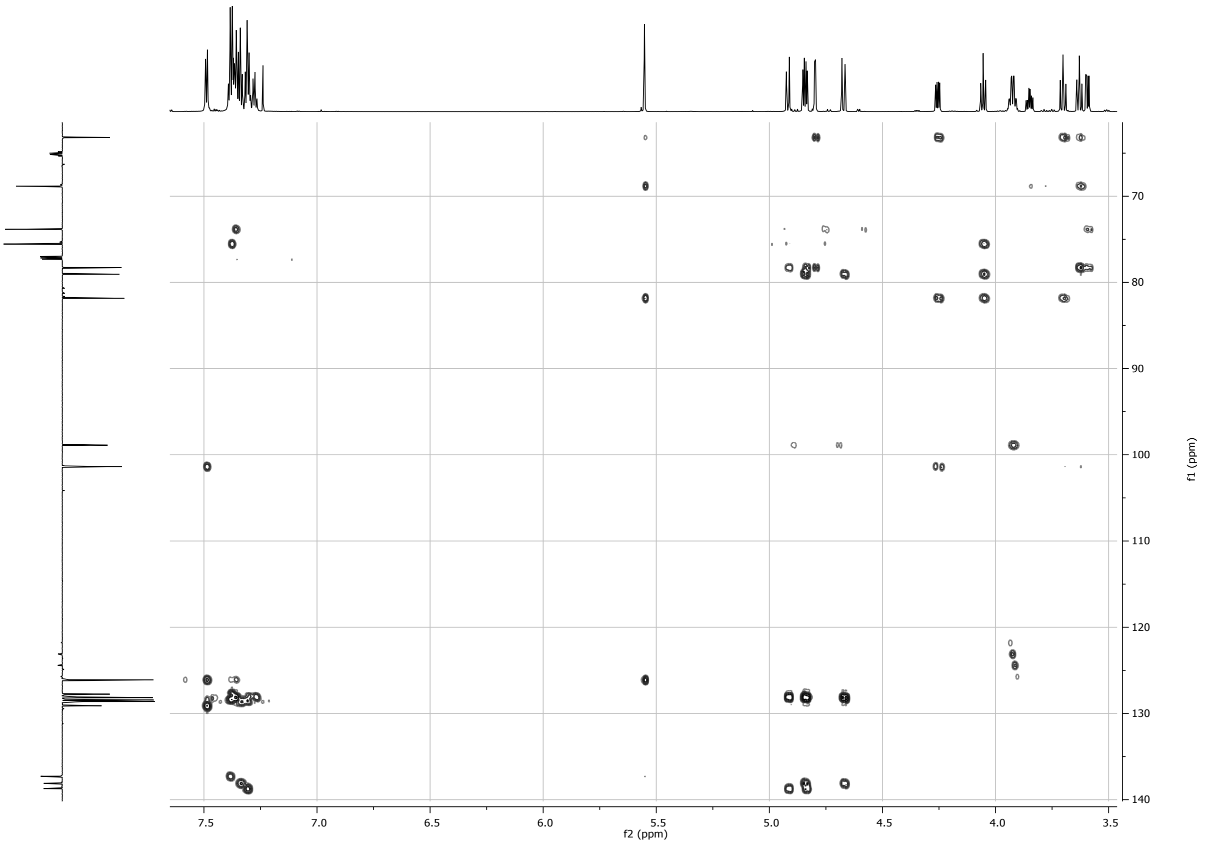

Supplementary Figure S40. HMBC NMR, $CDCl_3$ of compound **S5**

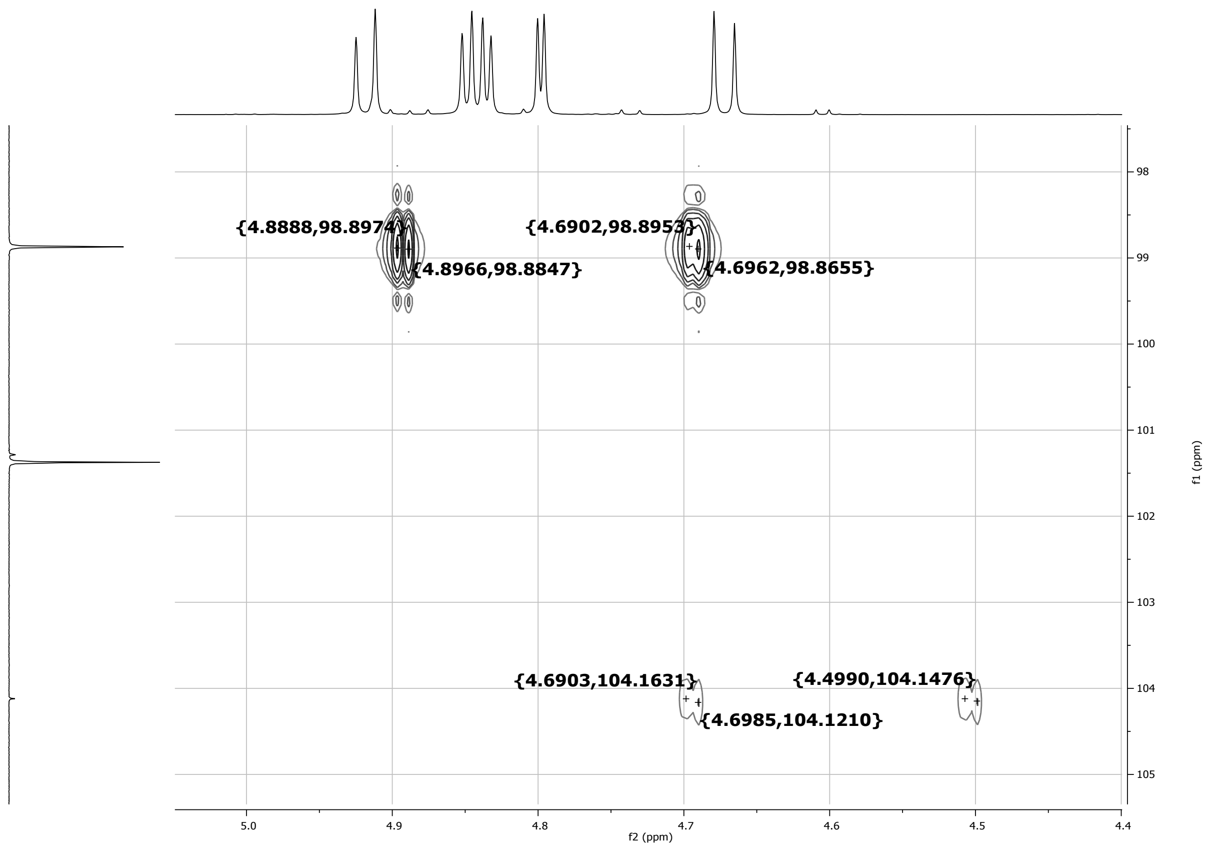

Supplementary Figure S41. HMBC-Gated NMR, $CDCl_3$ of compound **S5**

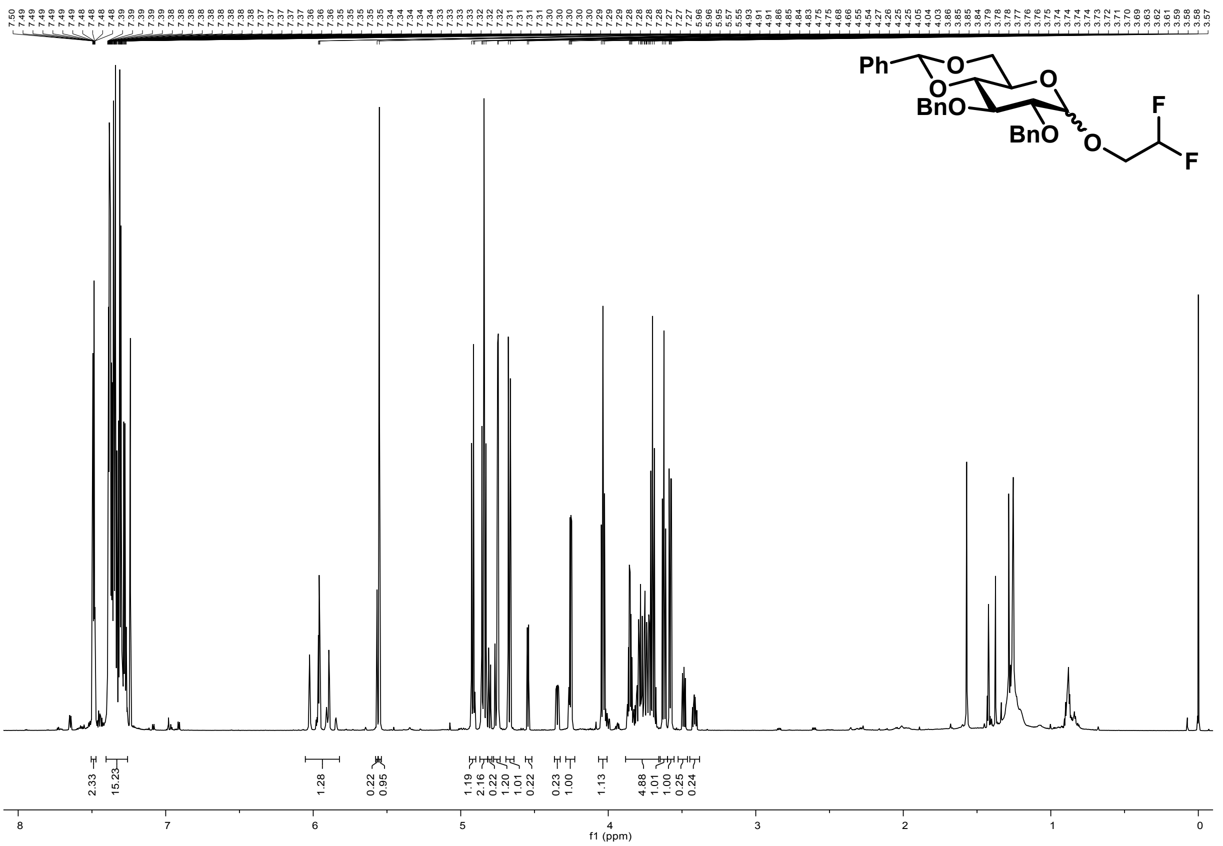

Supplementary Figure S42. ^{1}H NMR, 850 MHz, $CDCl_3$ of compound **S6**

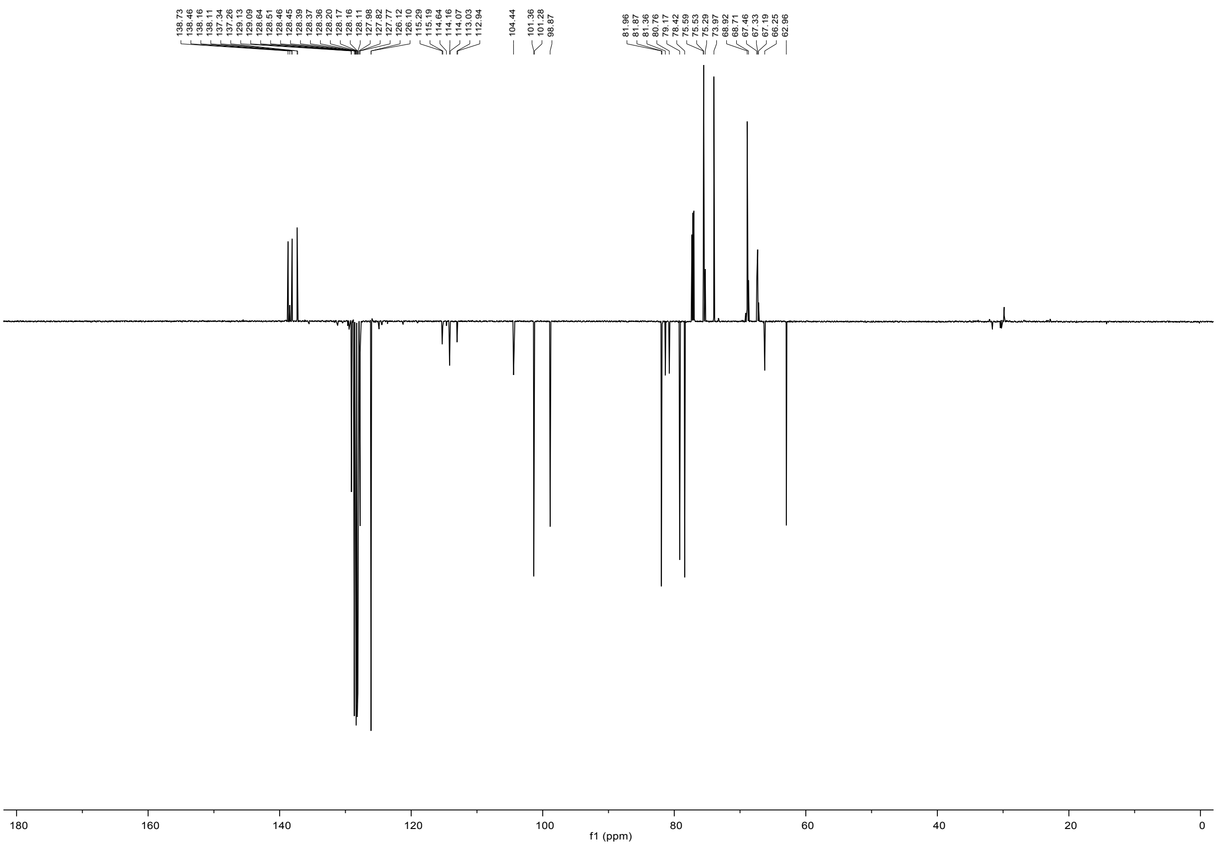

Supplementary Figure S43. $^{13}C\{^{1}H\}$ NMR, 214 MHz, $CDCl_3$ of compound **S6**

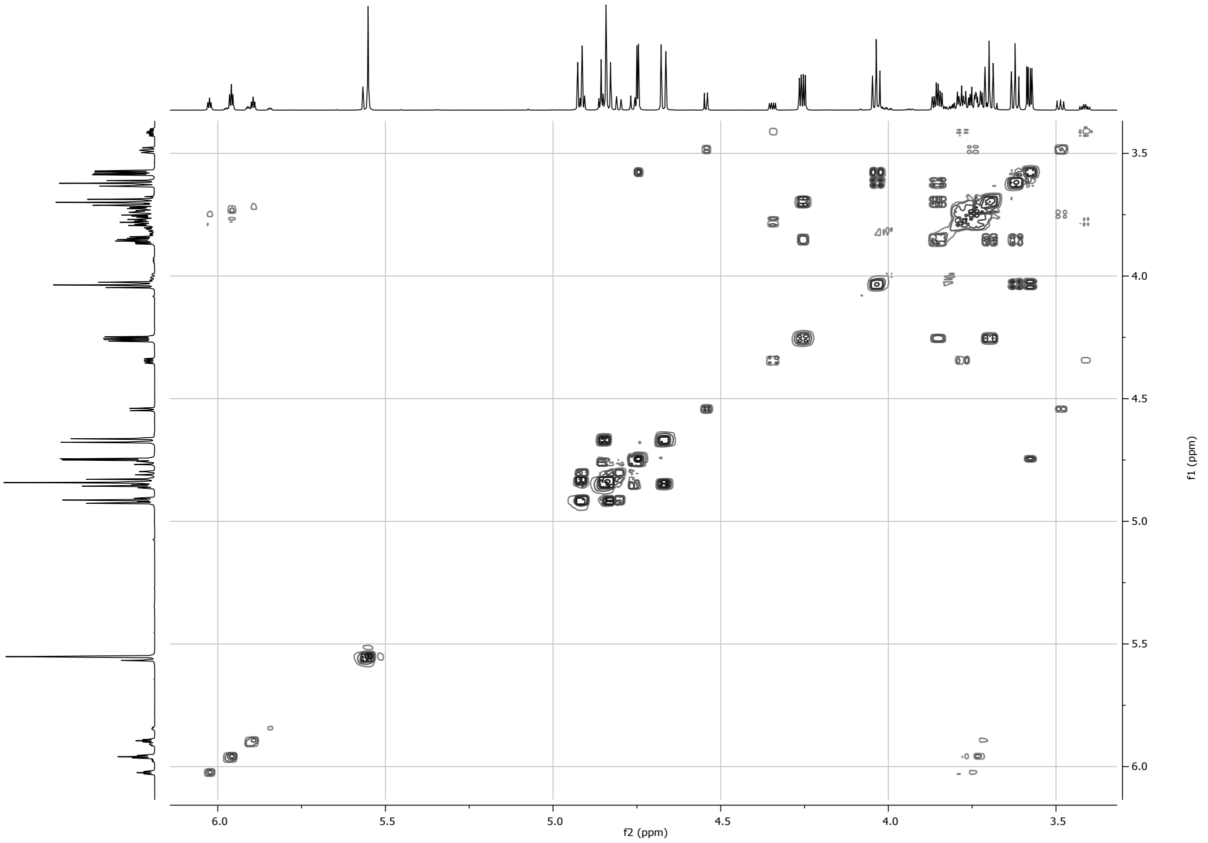

Supplementary Figure S44. HH-COSY NMR, $CDCl_3$ of compound **S6**

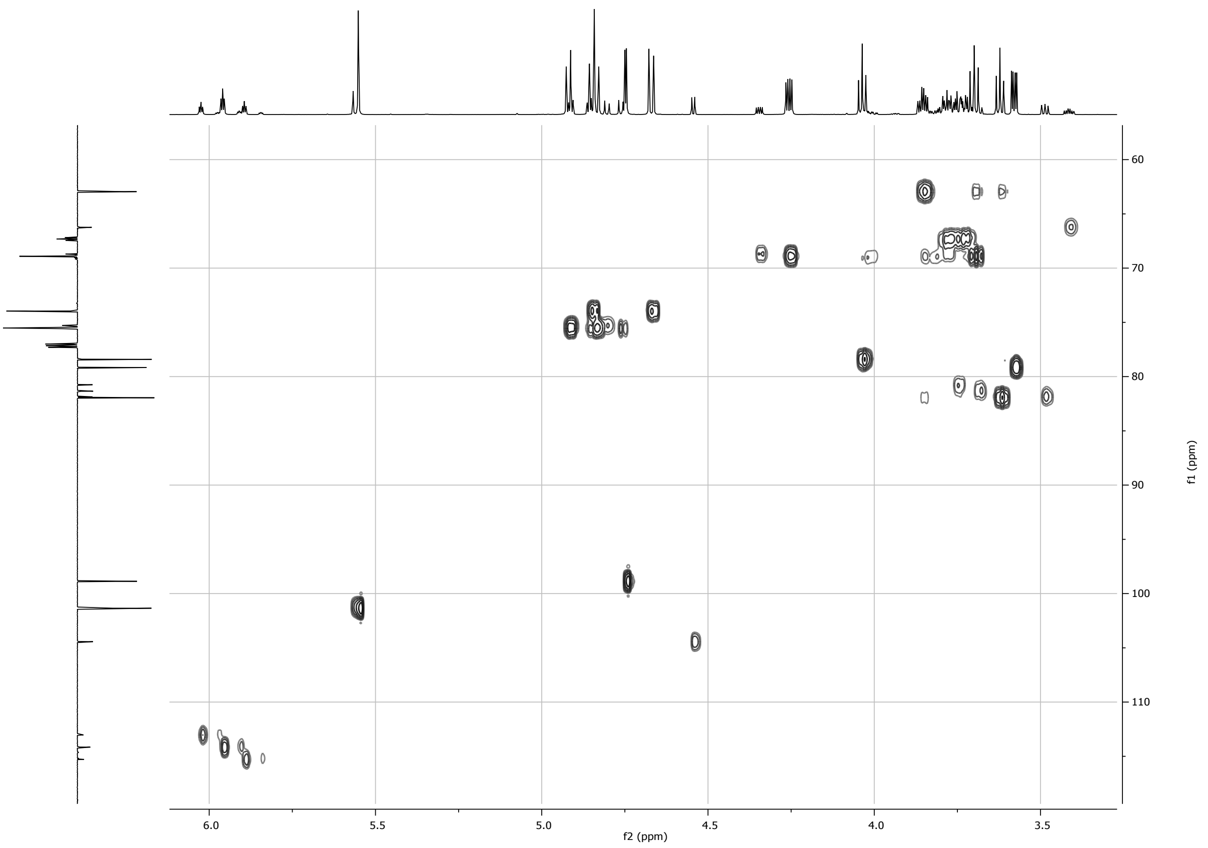

Supplementary Figure S45. HSQC{^{1}H} NMR, $CDCl_3$ of compound **S6**

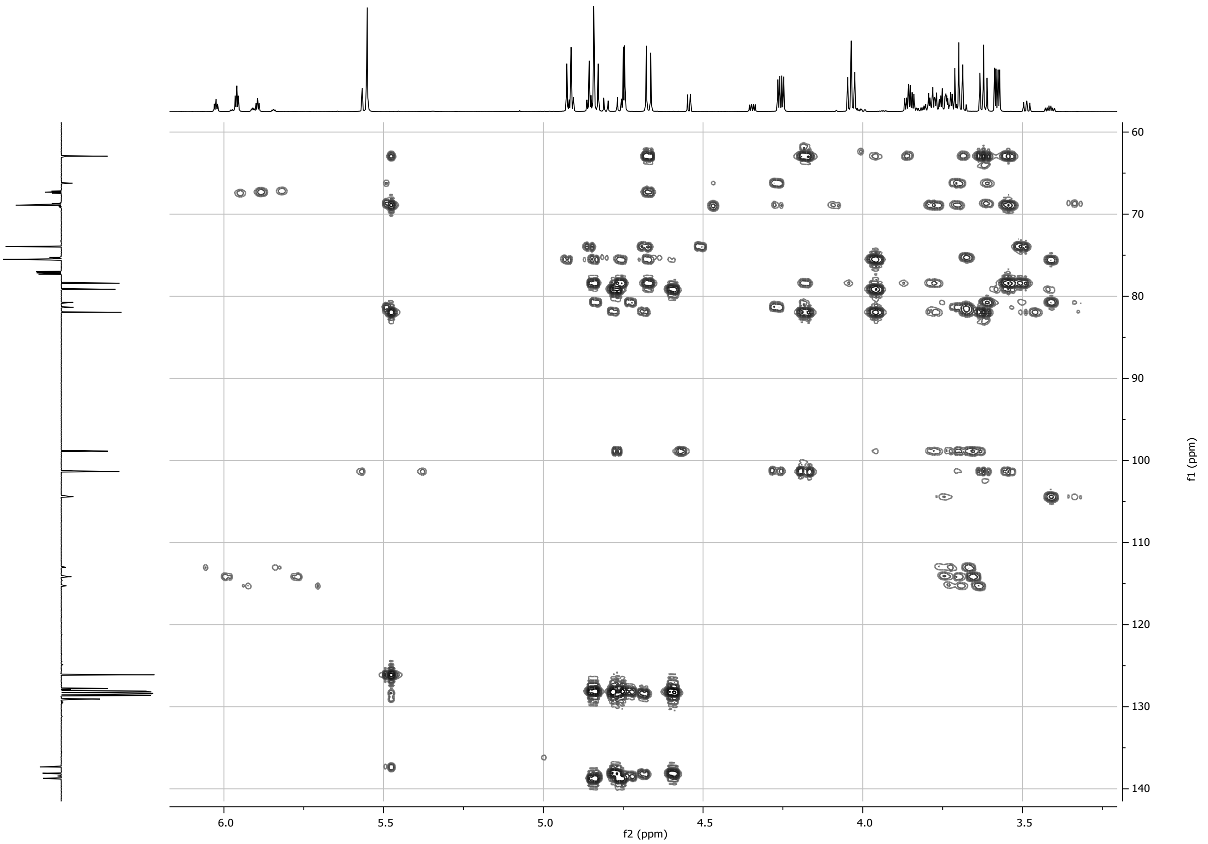

Supplementary Figure S46. HMBC NMR, $CDCl_3$ of compound **S6**

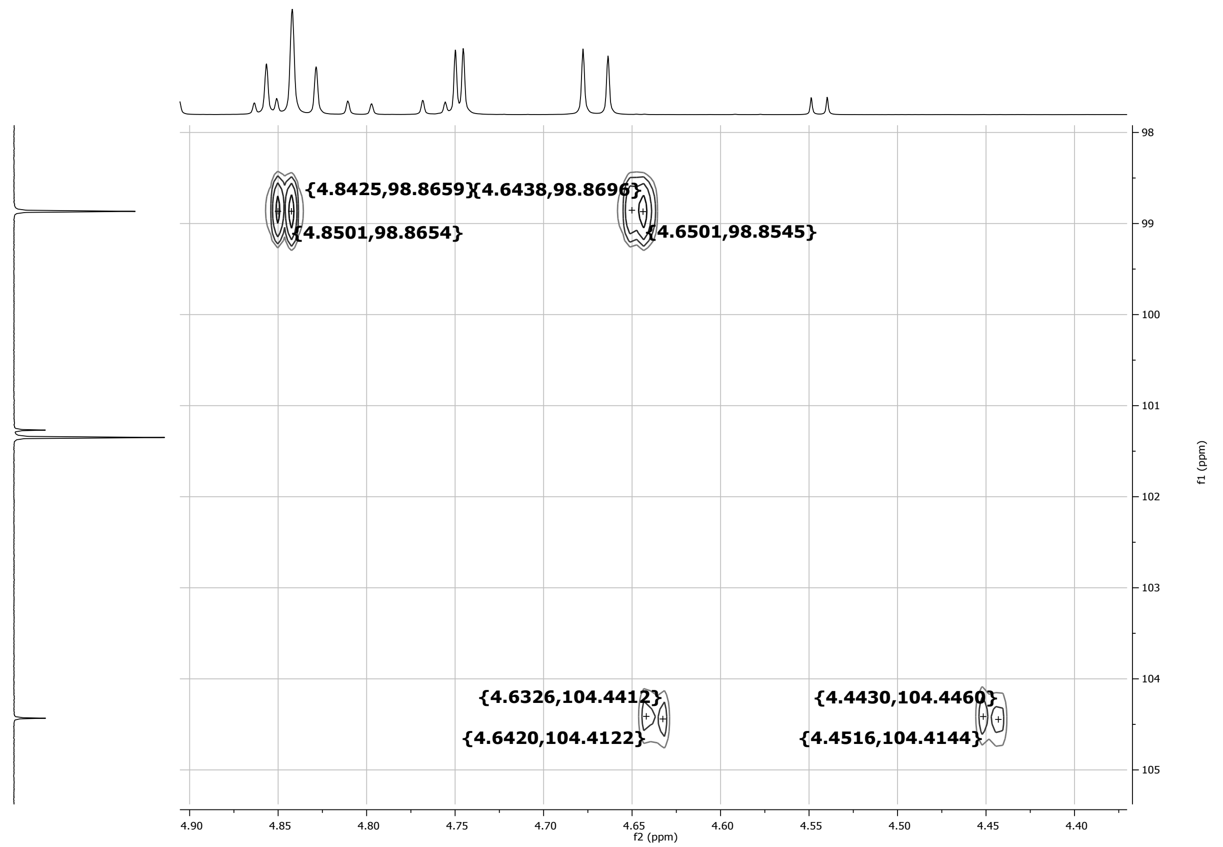

Supplementary Figure S47. HMBC-Gated NMR, $CDCl_3$ of compound **S6**

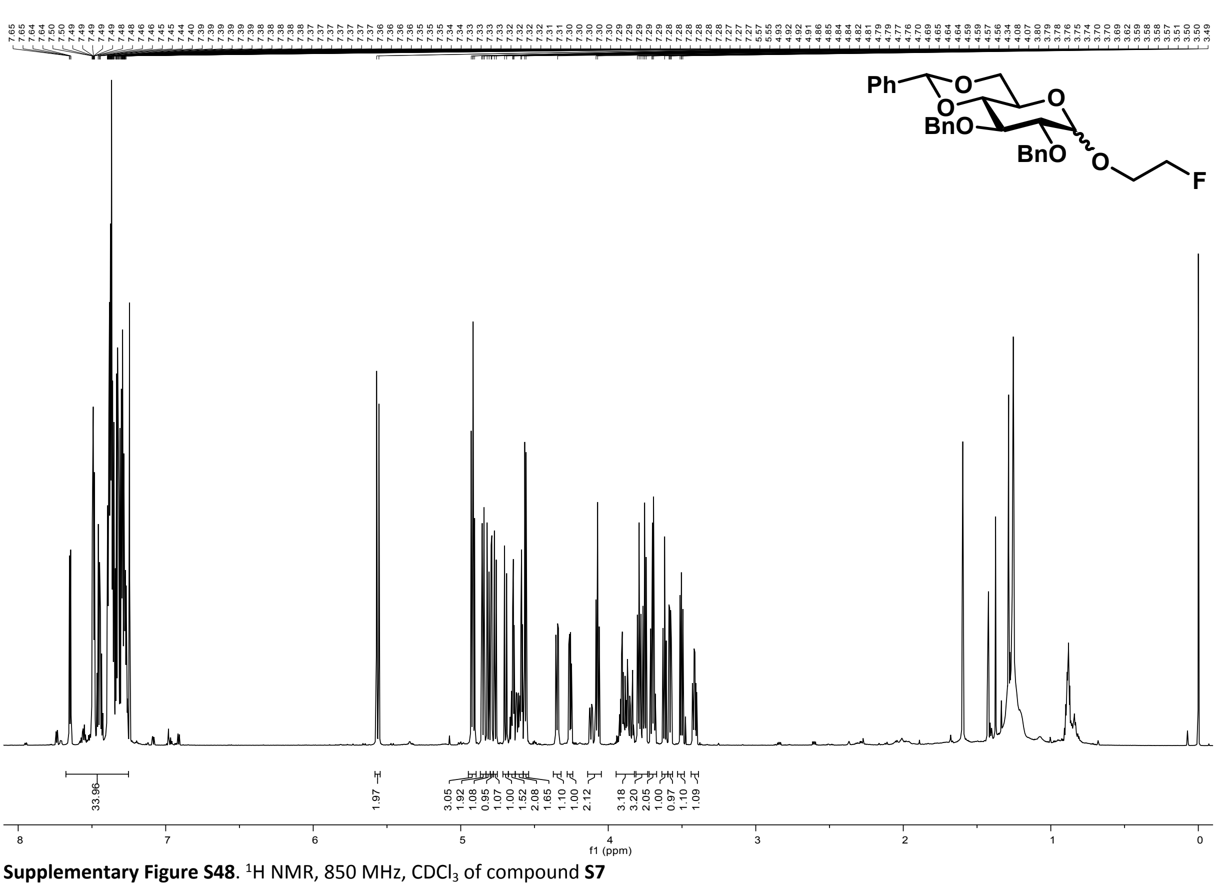

Supplementary Figure S48. ^{1}H NMR, 850 MHz, $CDCl_3$ of compound **S7**

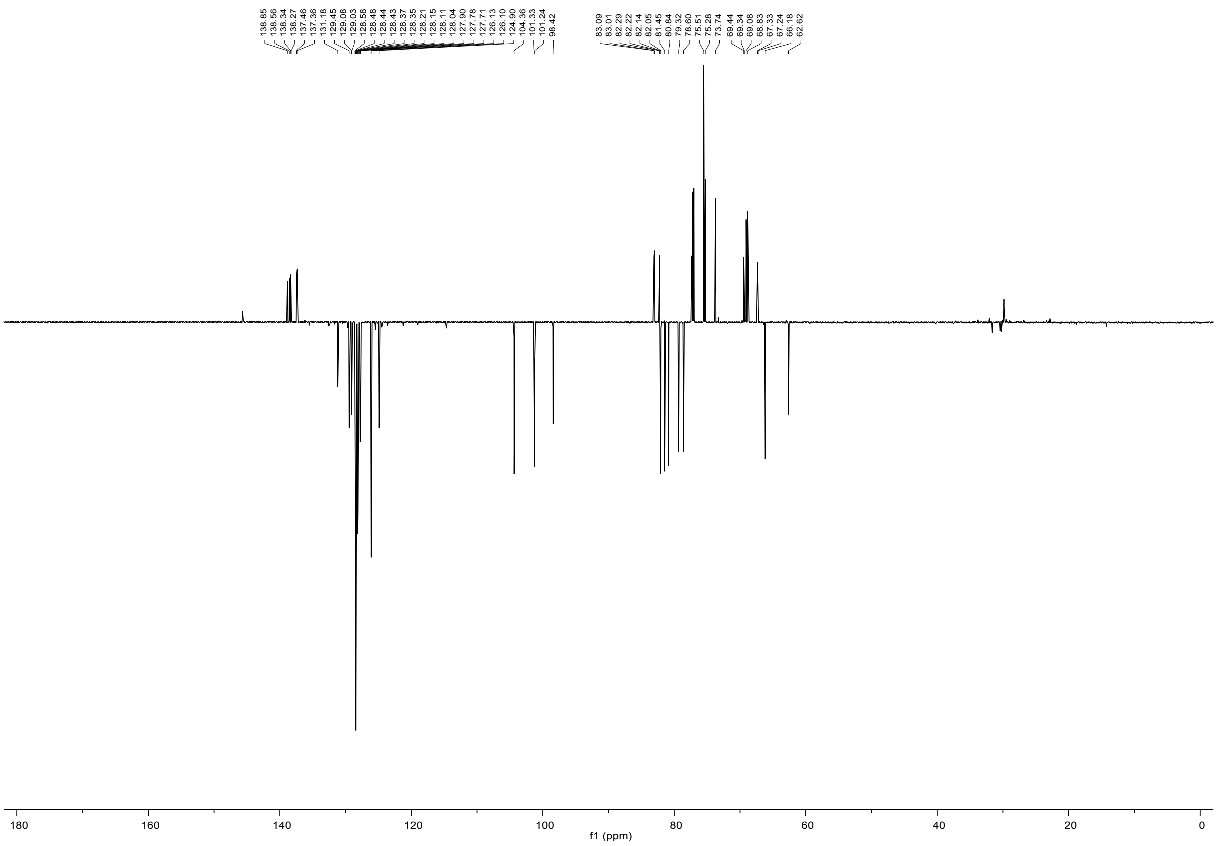

Supplementary Figure S49. $^{13}C\{^{1}H\}$ NMR, 214 MHz, $CDCl_3$ of compound **S7**

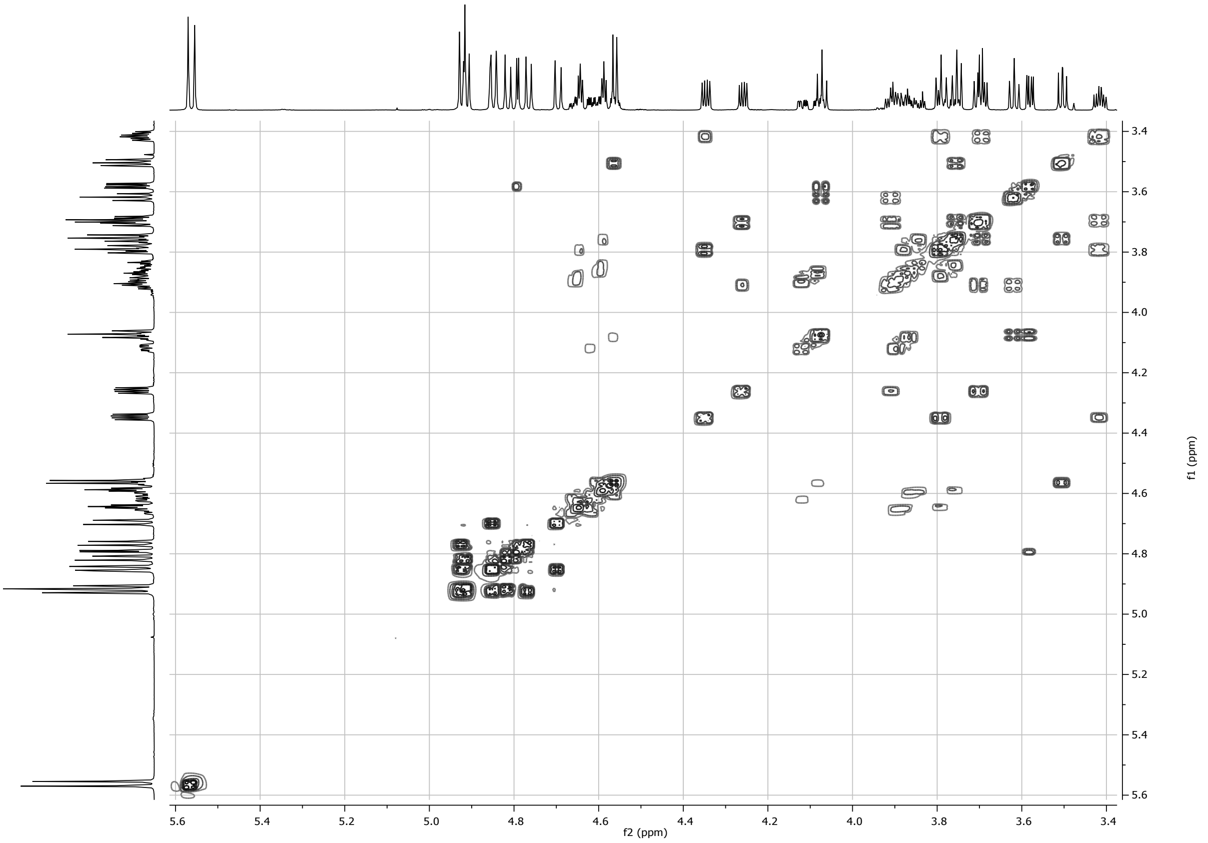

Supplementary Figure S50. HH-COSY NMR, $CDCl_3$ of compound **S7**

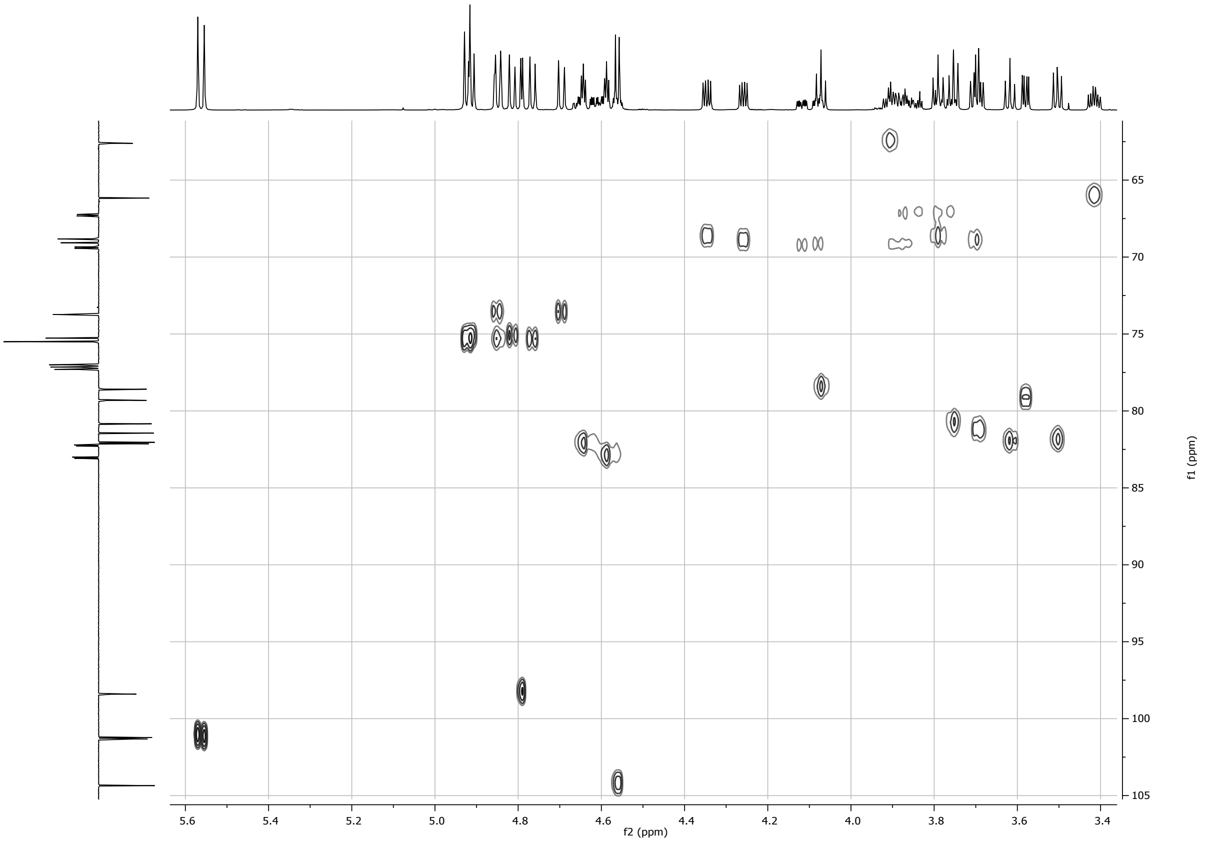

Supplementary Figure S51. HSQC{^{1}H} NMR, $CDCl_3$ of compound **S7**

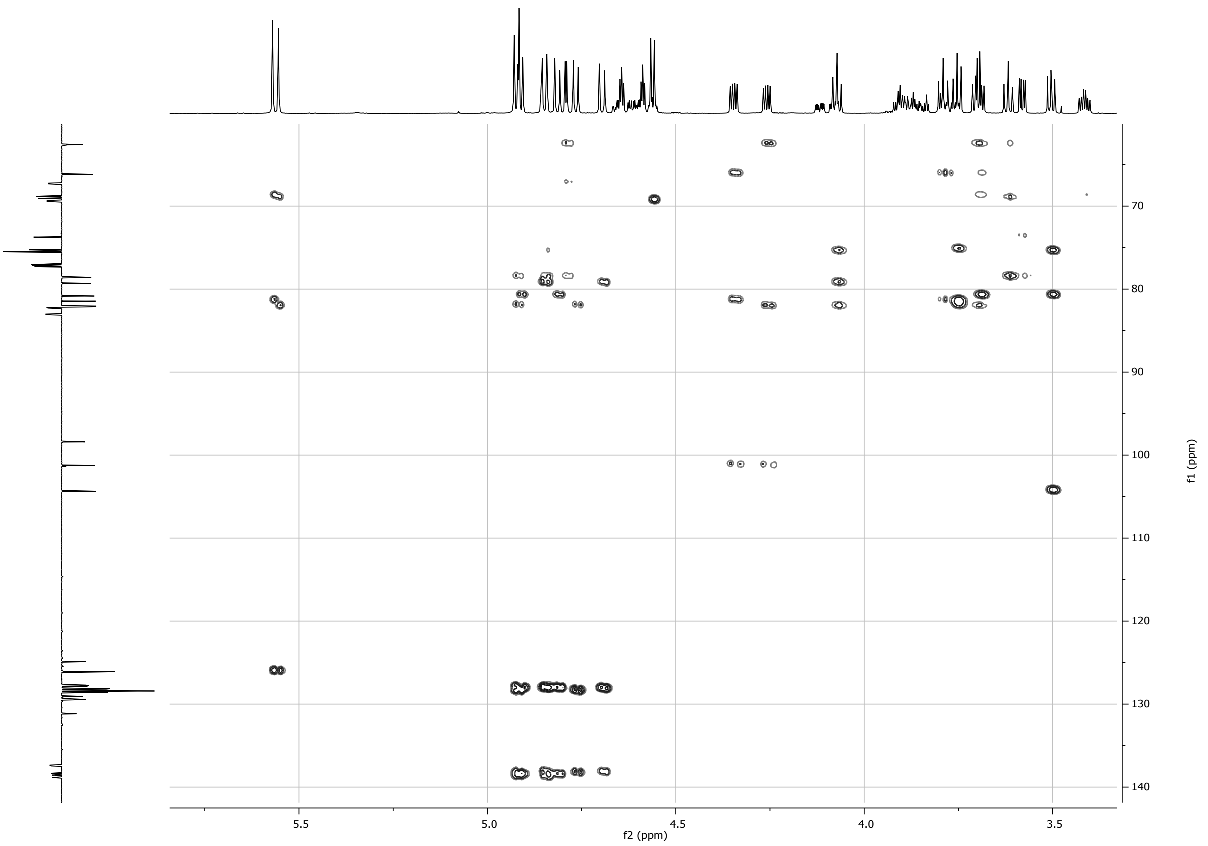

Supplementary Figure S52. HMBC NMR, $CDCl_3$ of compound **S7**

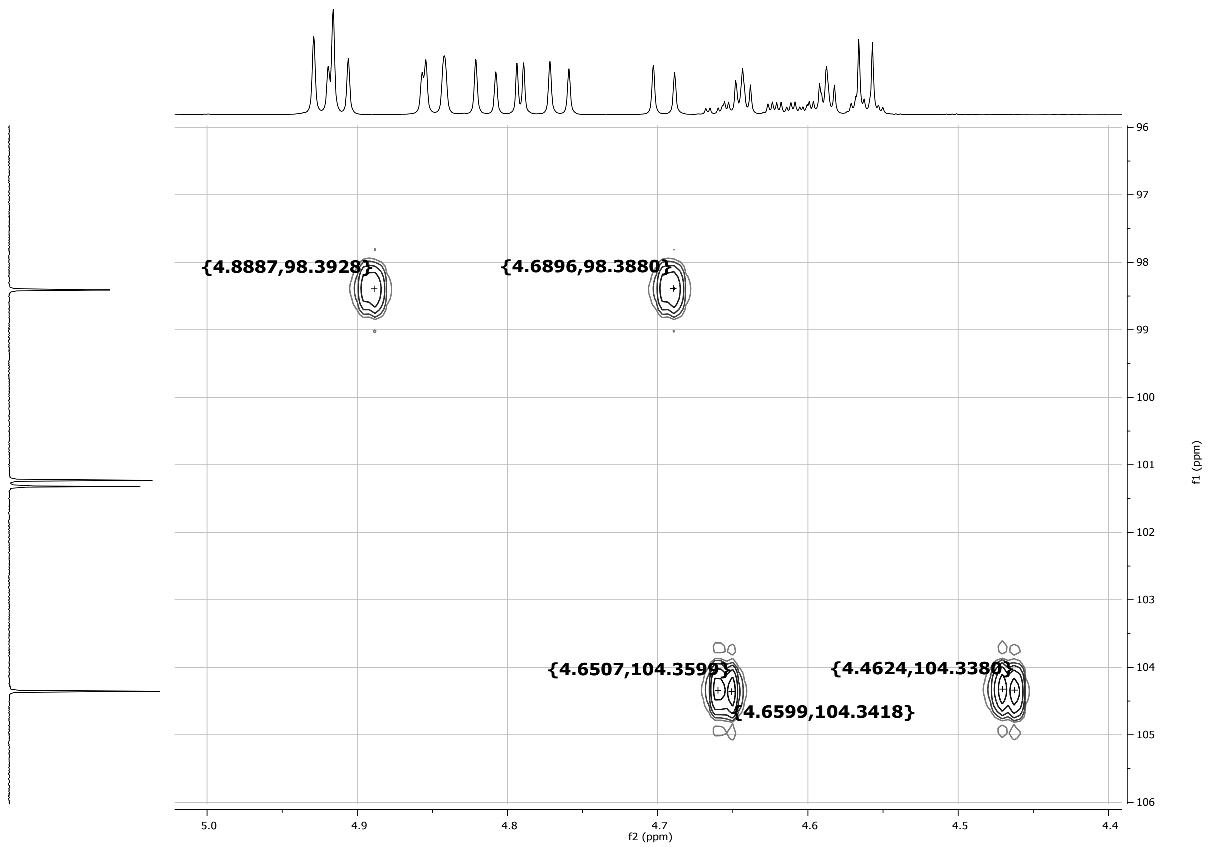

Supplementary Figure S53. HMBC-Gated NMR, $CDCl_3$ of compound **S7**

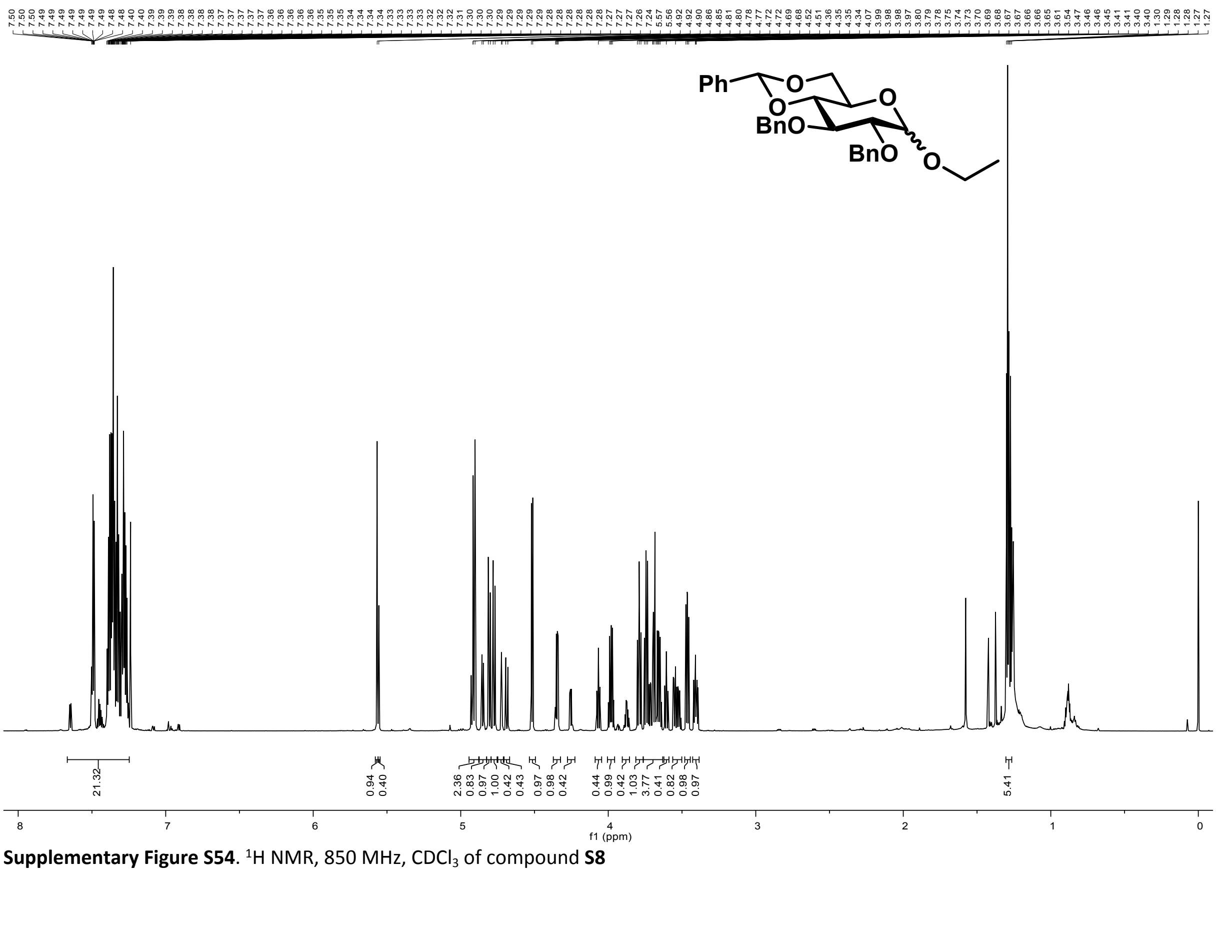

Supplementary Figure S54. ^1H NMR, 850 MHz, $CDCl_3$ of compound **S8**

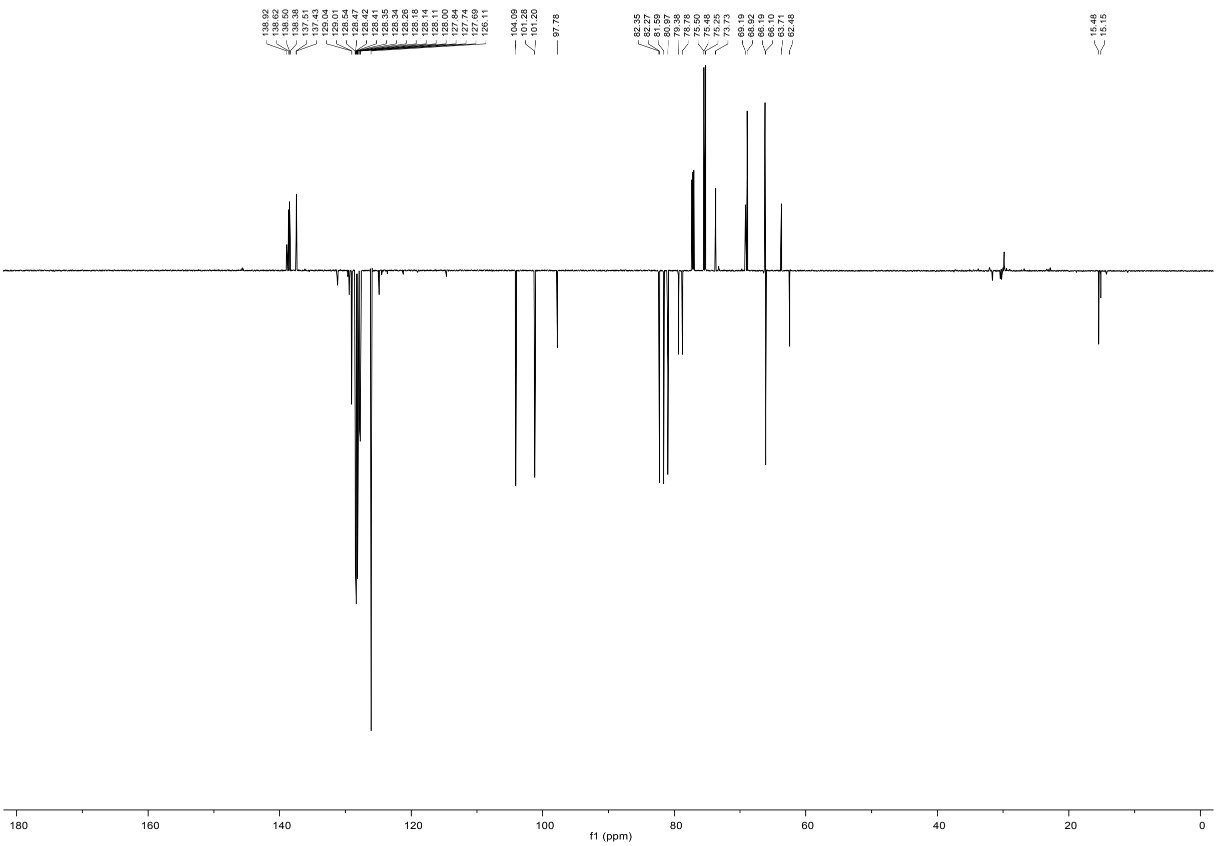

Supplementary Figure S55. $^{13}C\{^1H\}$ NMR, 214 MHz, $CDCl_3$ of compound **S8**

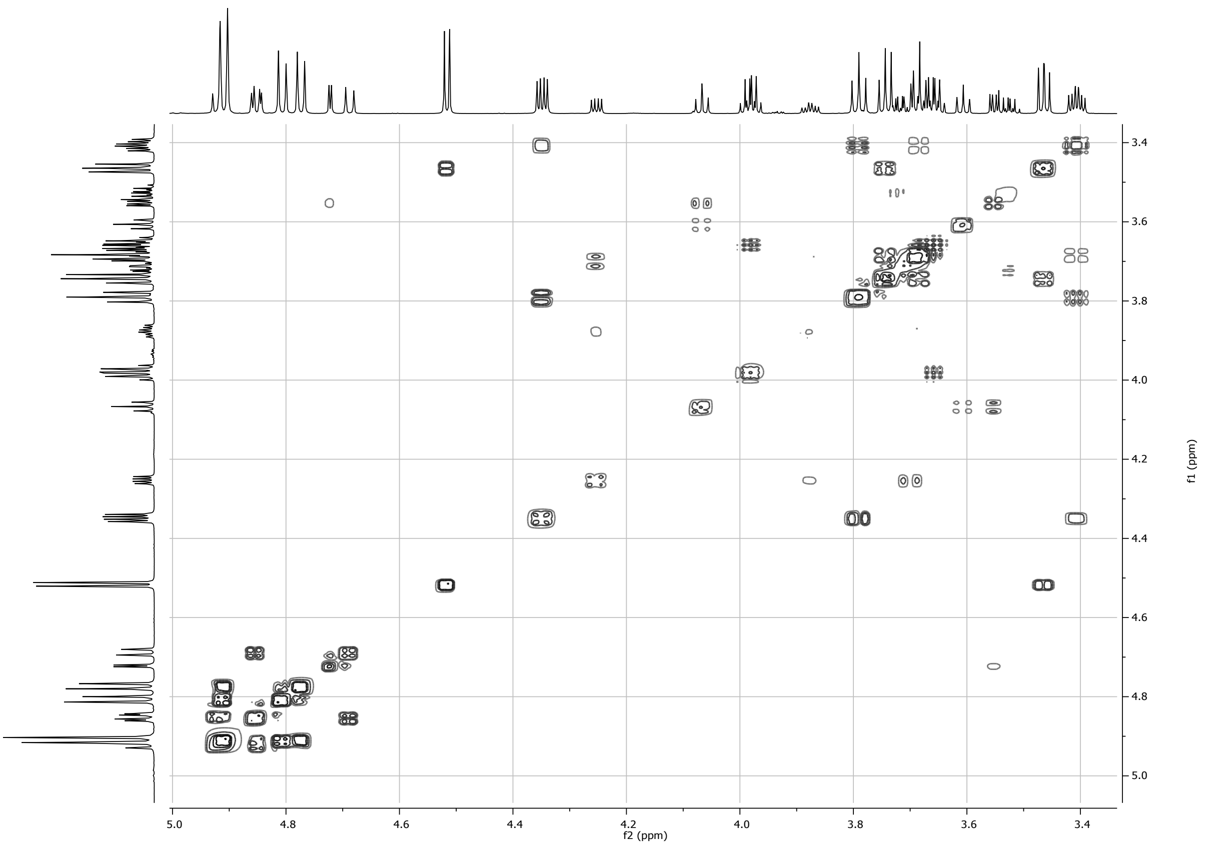

Supplementary Figure S56. HH-COSY NMR, $CDCl_3$ of compound **S8**

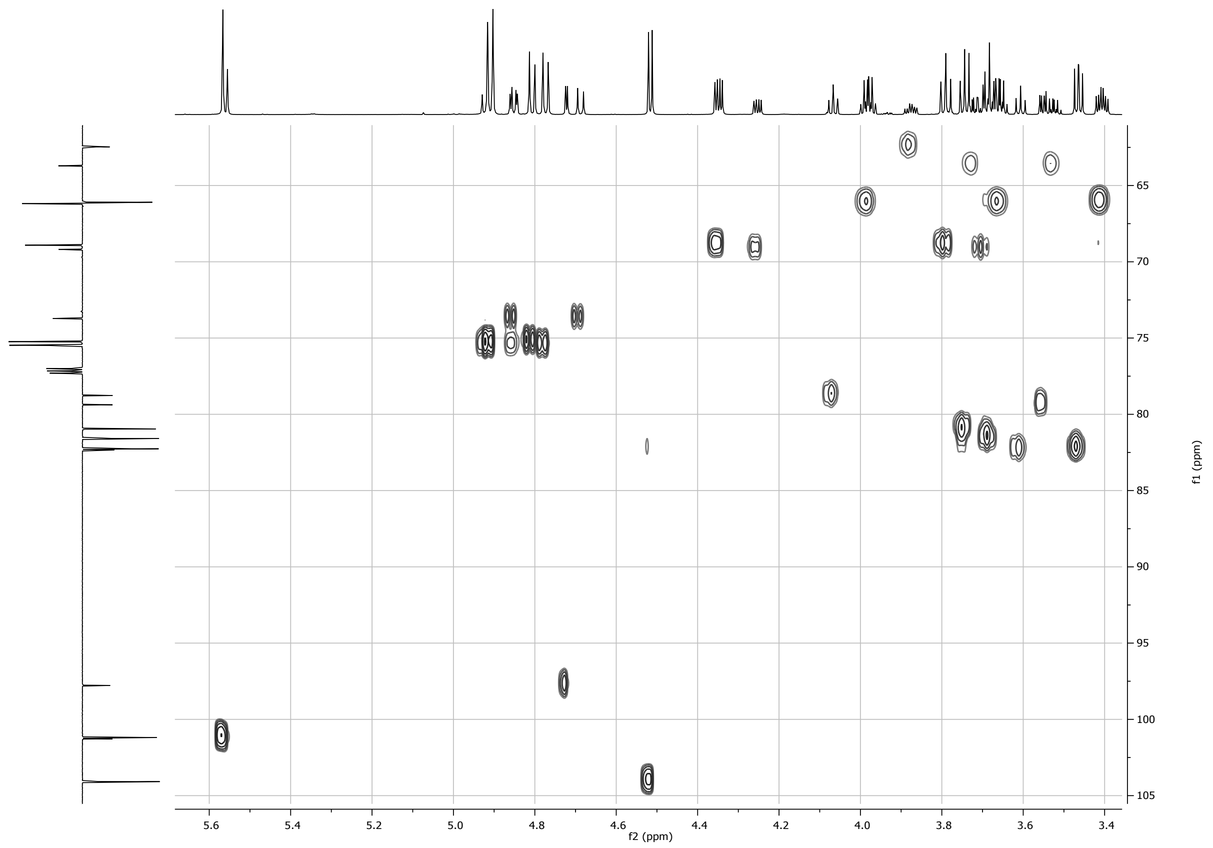

Supplementary Figure S57. HSQC{¹H} NMR, $CDCl_3$ of compound **S8**

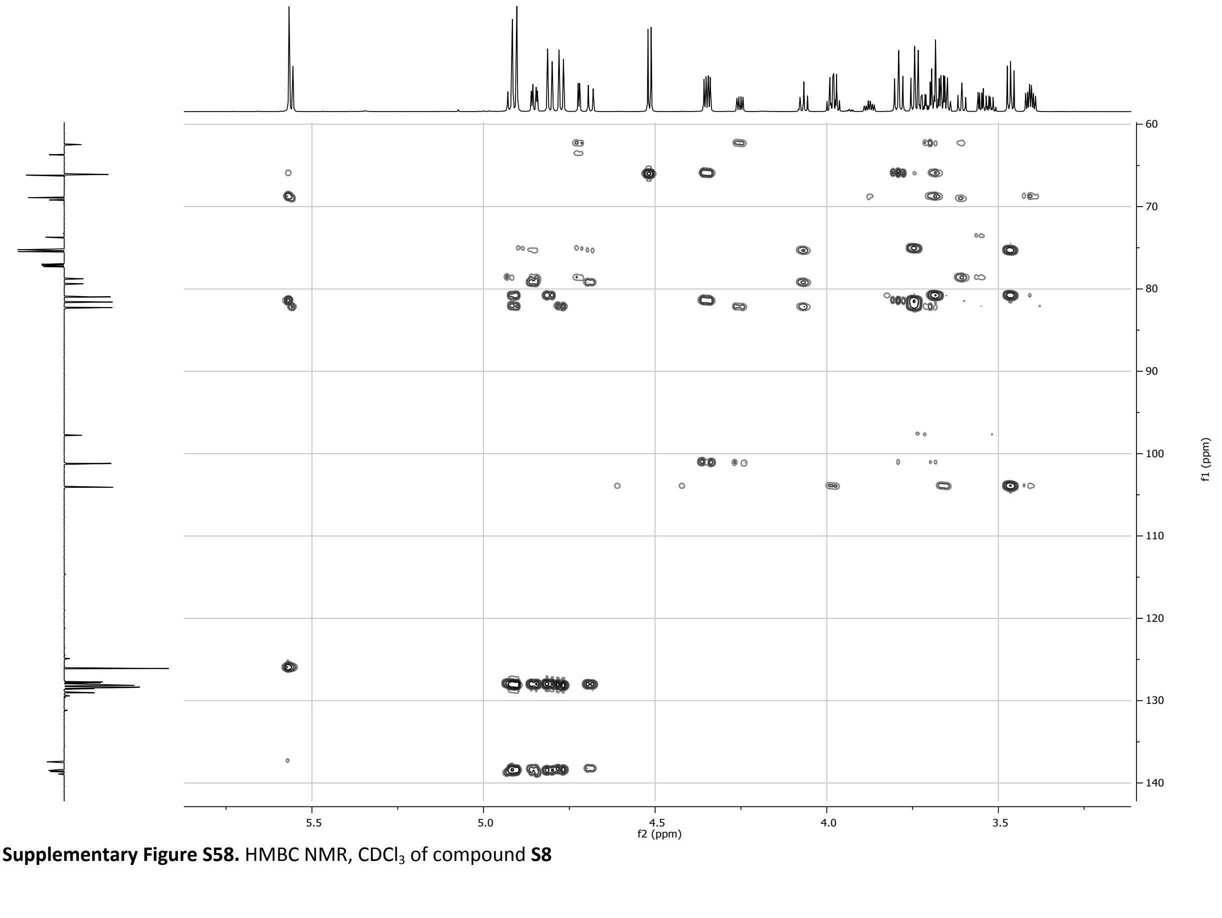

Supplementary Figure S58. HMBC NMR, $CDCl_3$ of compound **S8**

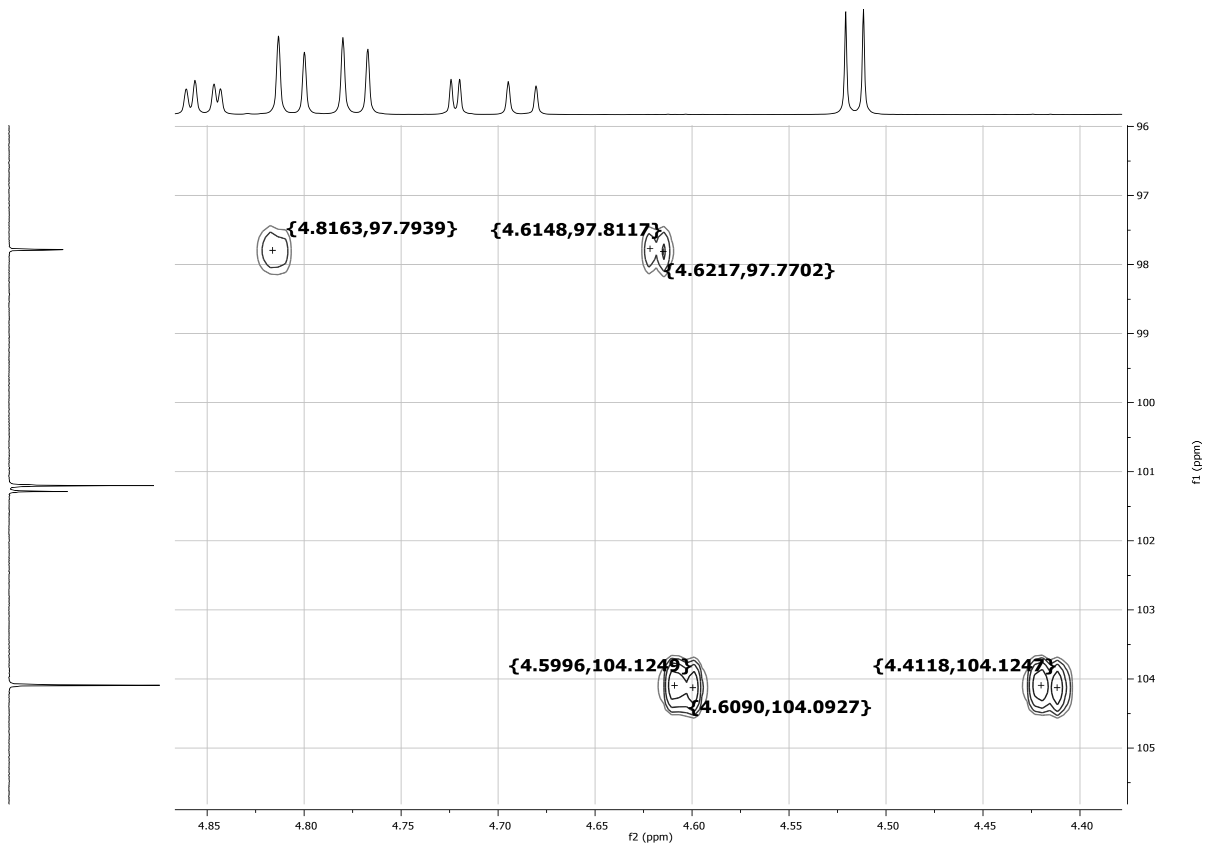

Supplementary Figure S59. HMBC-Gated NMR, $CDCl_3$ of compound **S8**

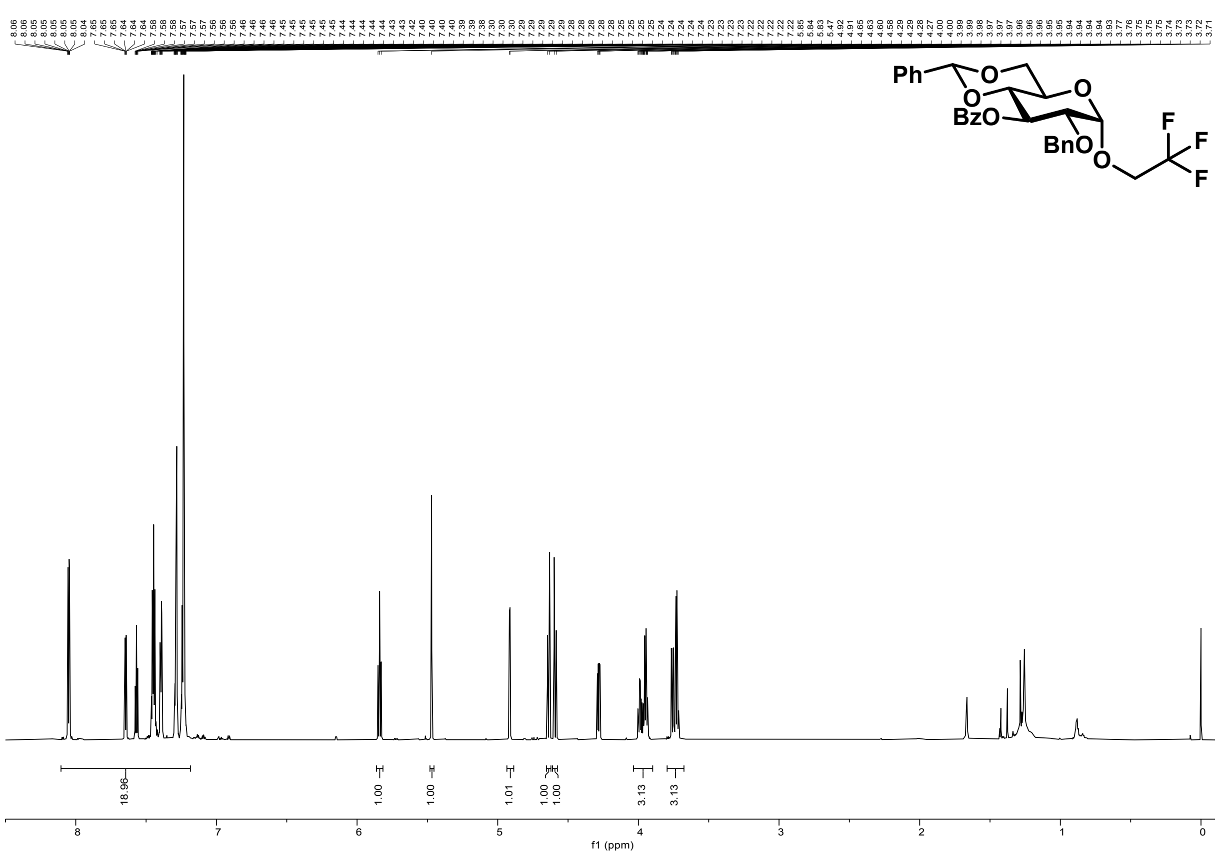

sSupplementary Figure S60. ^{1}H NMR, 850 MHz, $CDCl_3$ of compound **S9**

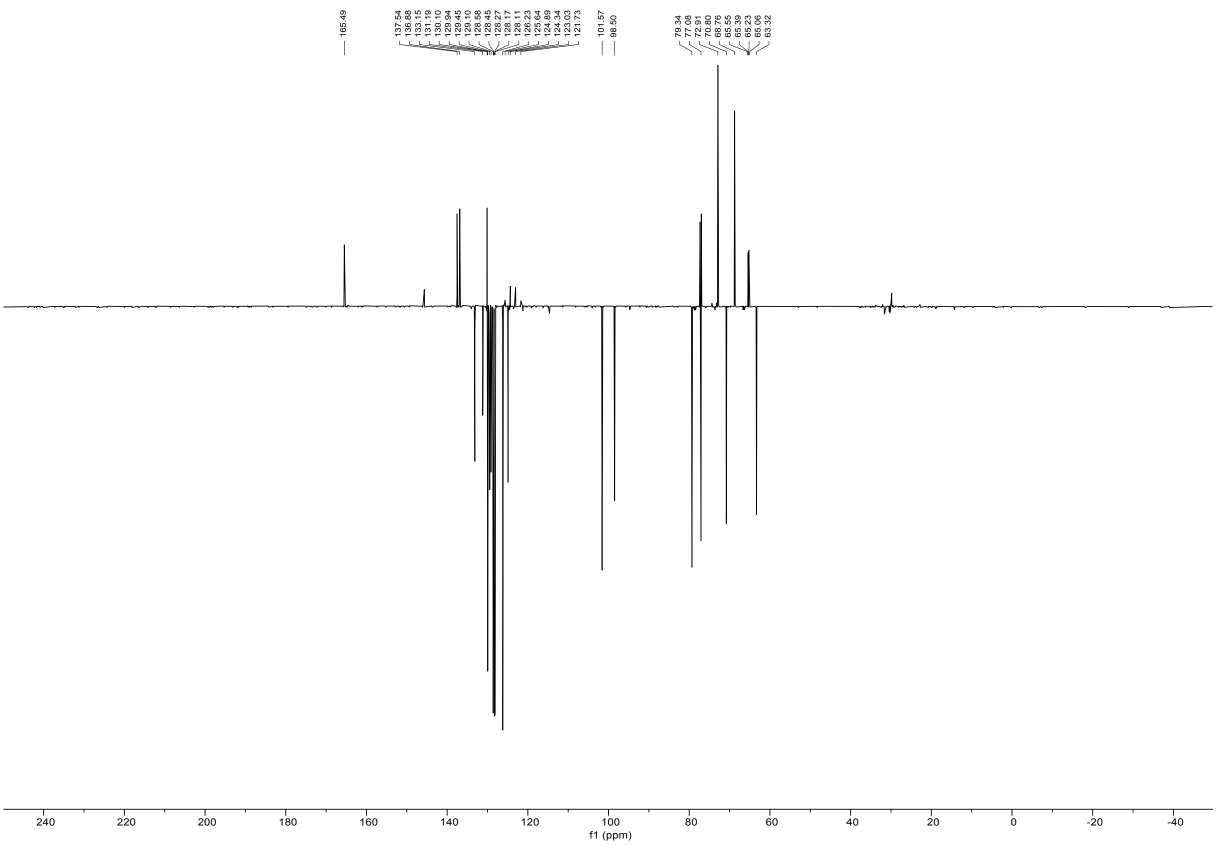

Supplementary Figure S61. $^{13}C\{^{1}H\}$ NMR, 214 MHz, $CDCl_3$ of compound **S9**

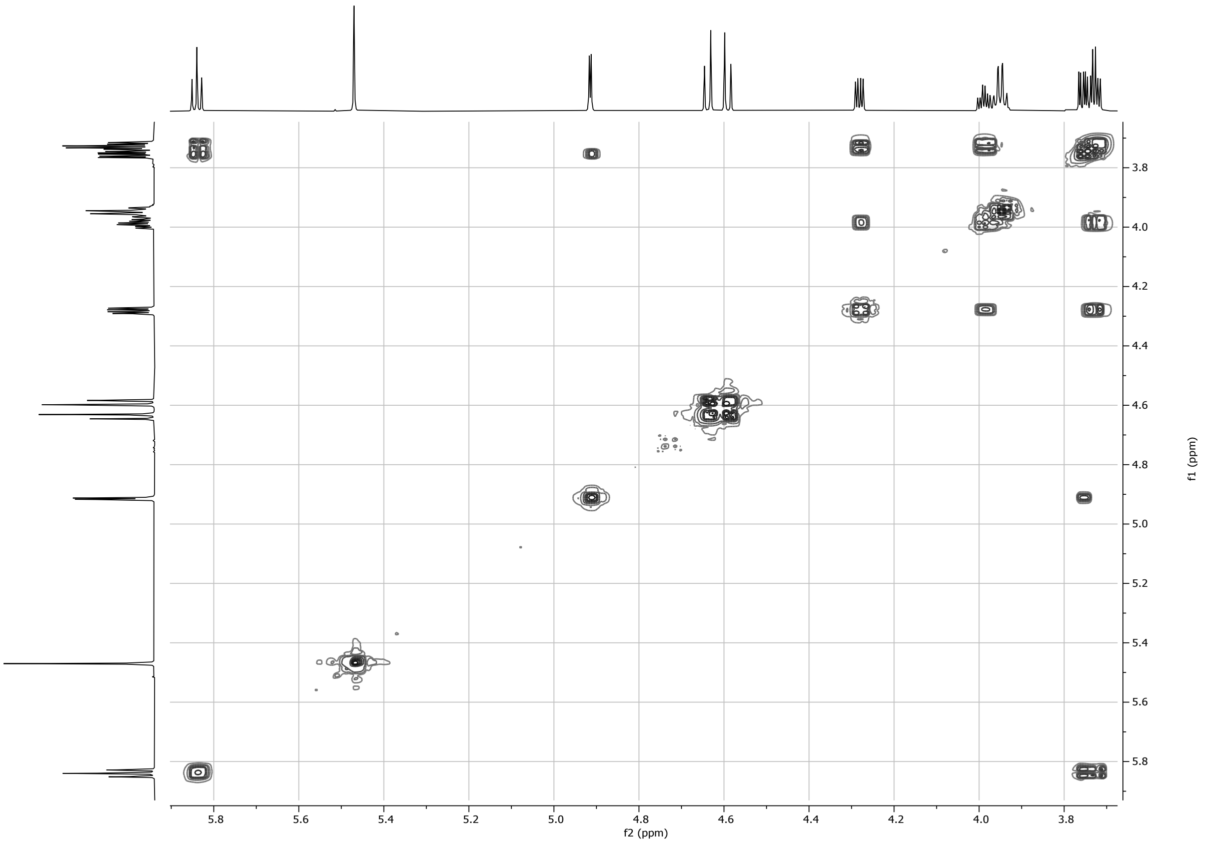

Supplementary Figure S62. HH-COSY NMR, $CDCl_3$ of compound **S9**

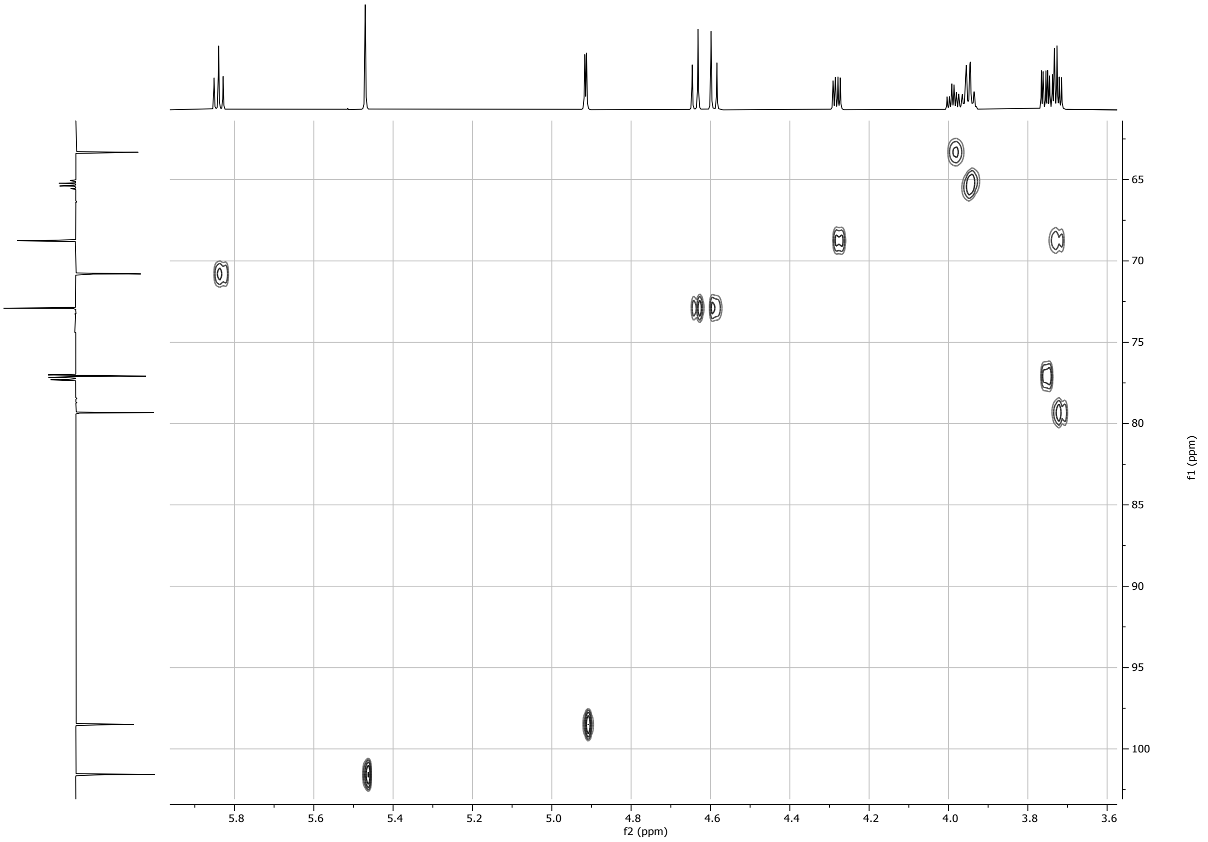

Supplementary Figure S63. HSQC{^{1}H} NMR, $CDCl_3$ of compound S**9**

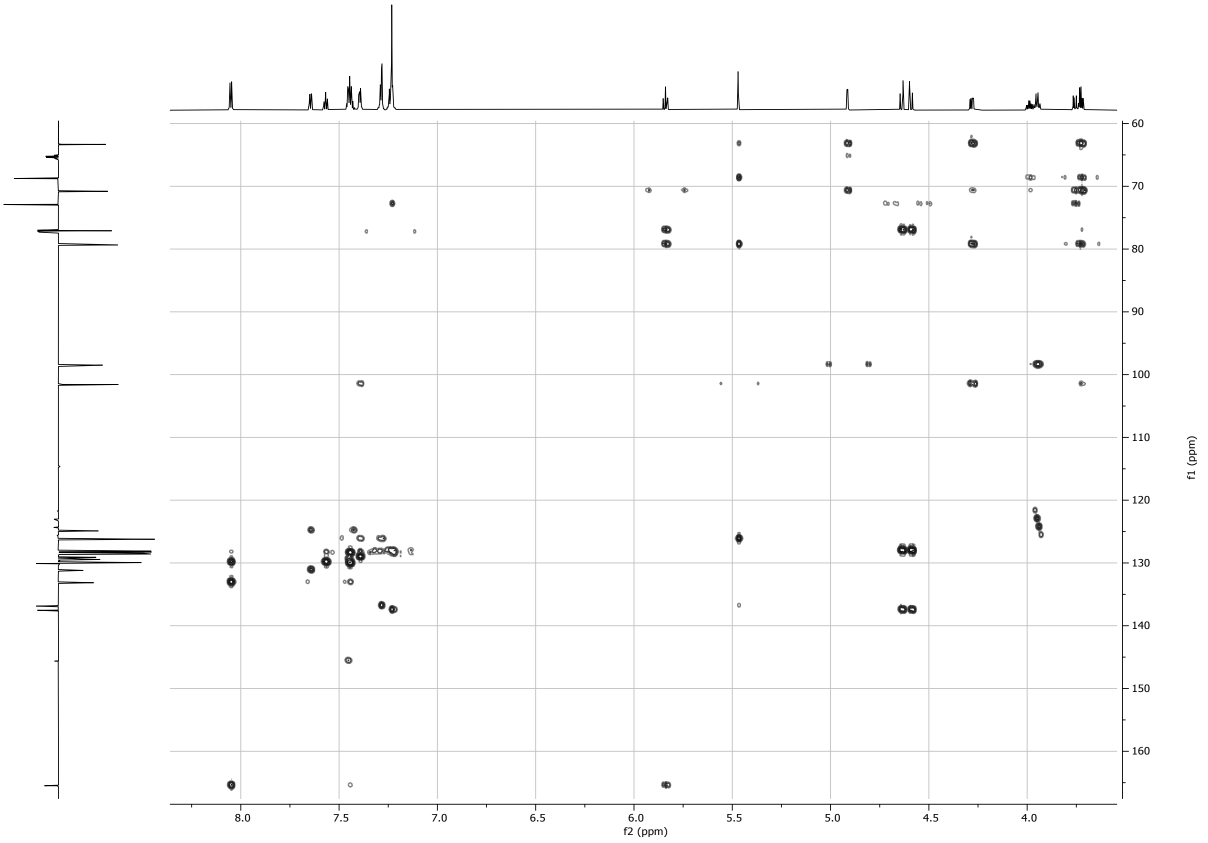

Supplementary Figure S64. HMBC NMR, $CDCl_3$ of compound **S9**

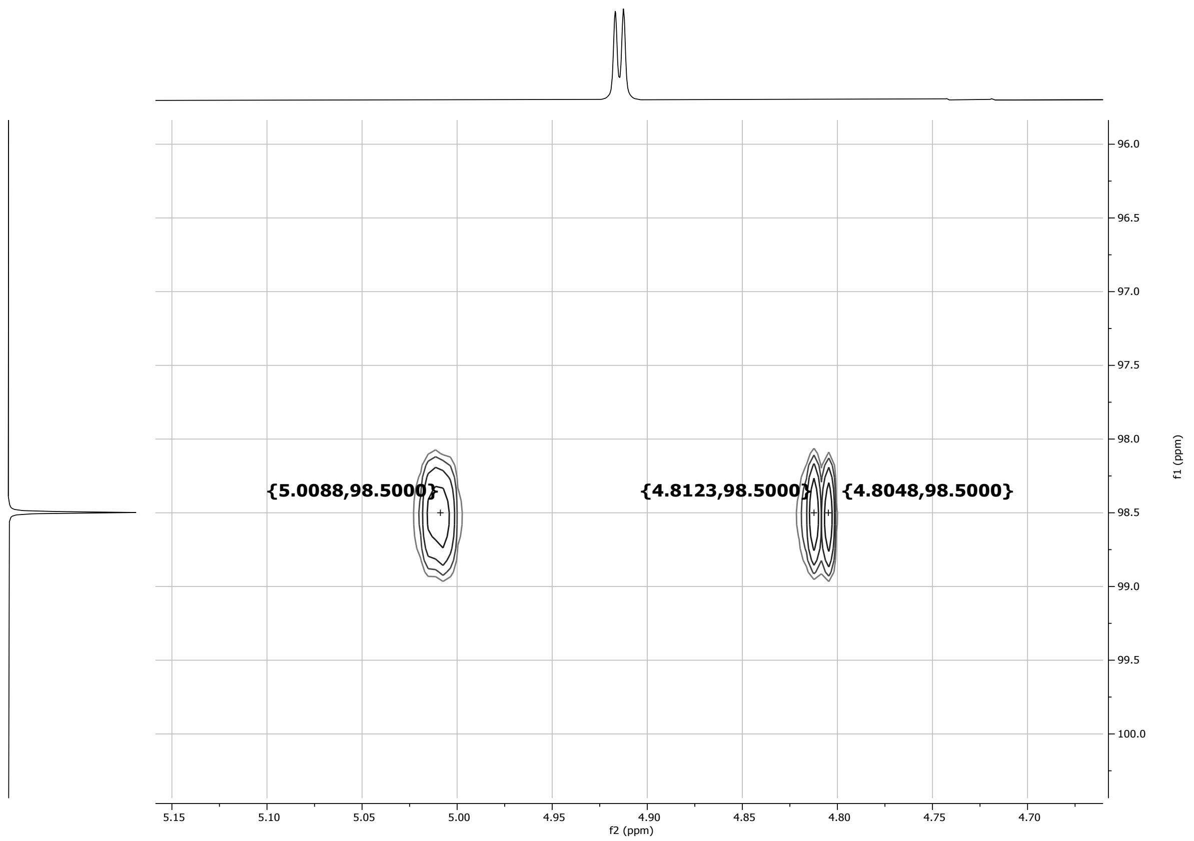

Supplementary Figure S65. HMBC-Gated NMR, $CDCl_3$ of compound **S9**

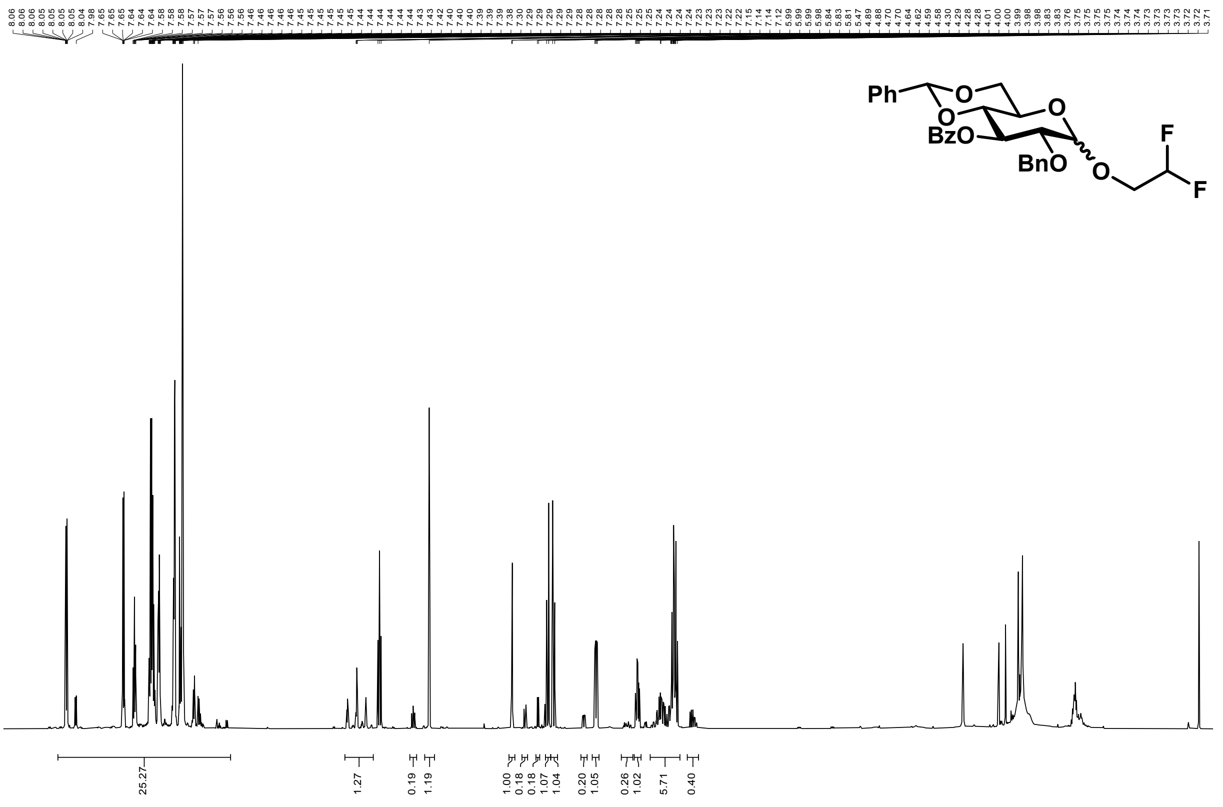

Supplementary Figure S66. ^1H NMR, 850 MHz, $CDCl_3$ of compound **S10**

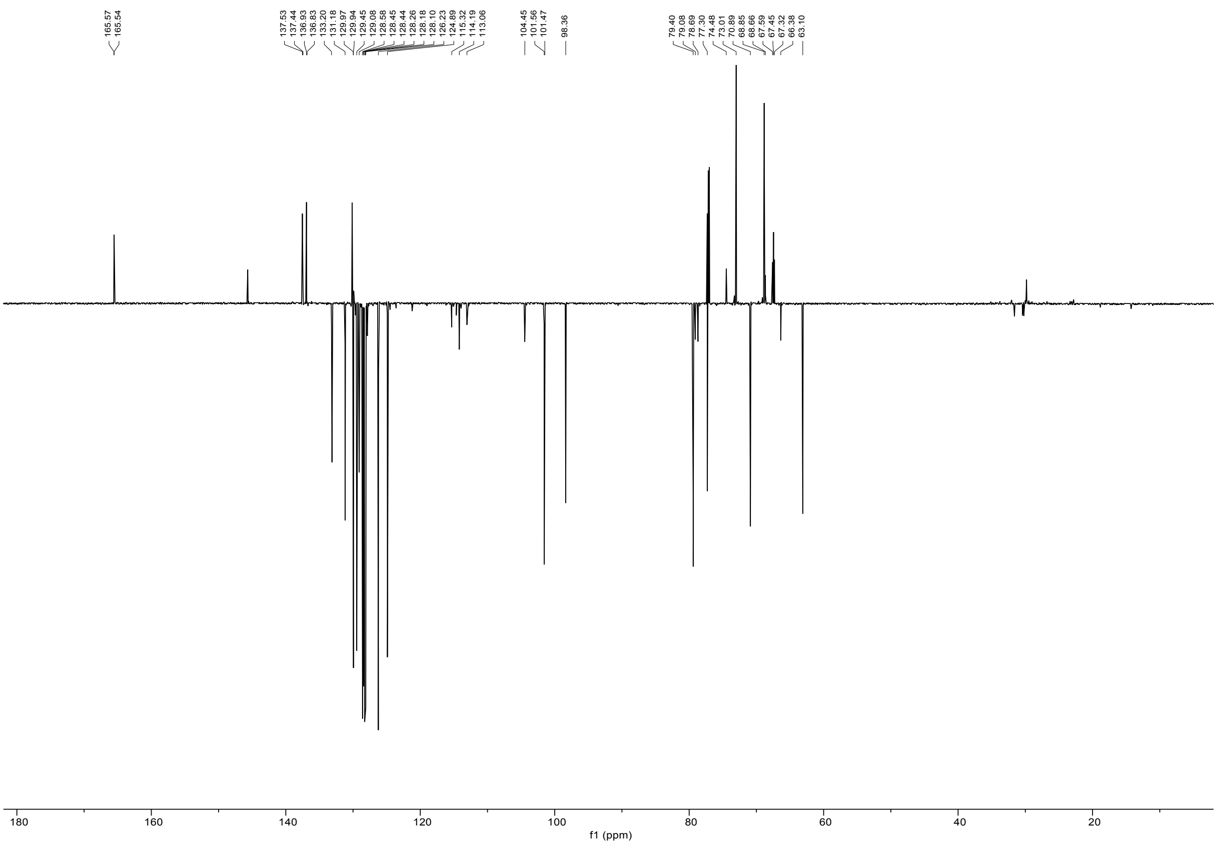

Supplementary Figure S67. $^{13}C\{^{1}H\}$ NMR, 214 MHz, $CDCl_3$ of compound **S10**

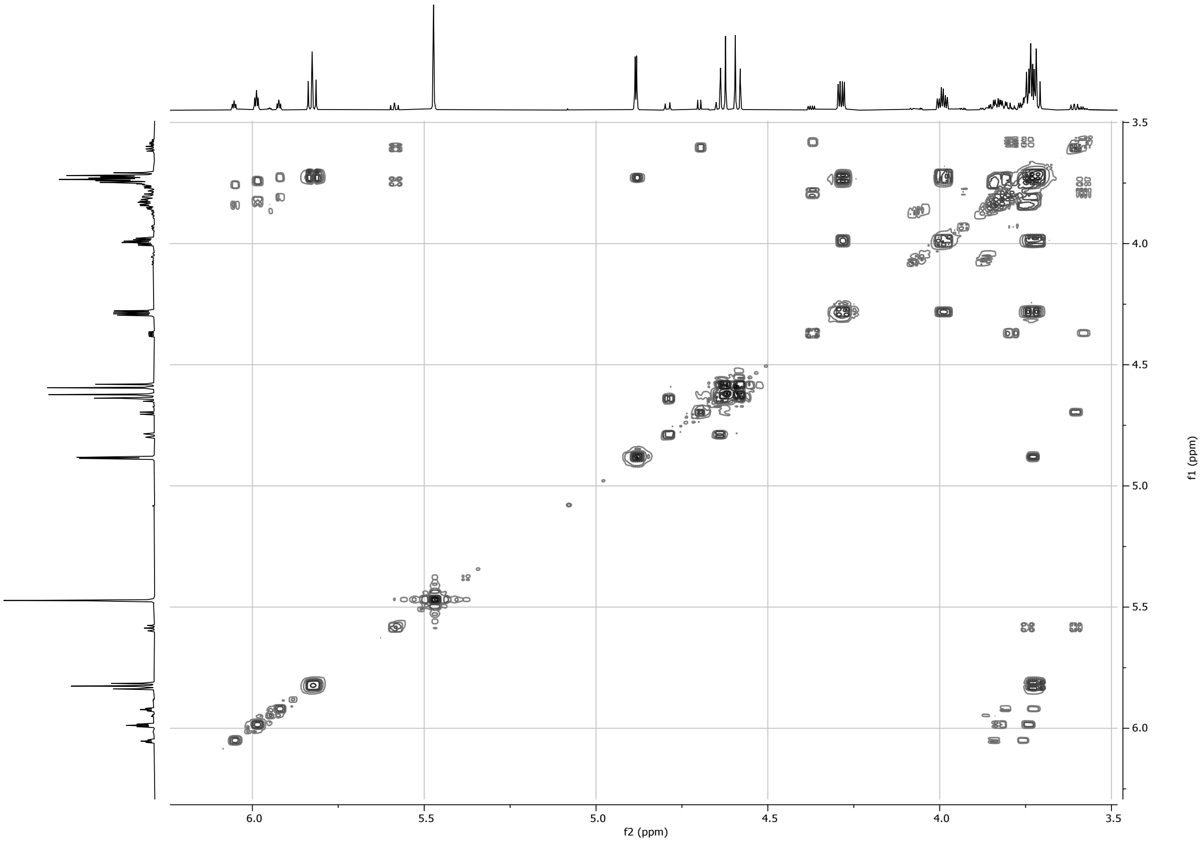

Supplementary Figure S68. HH-COSY NMR, $CDCl_3$ of compound **S10**

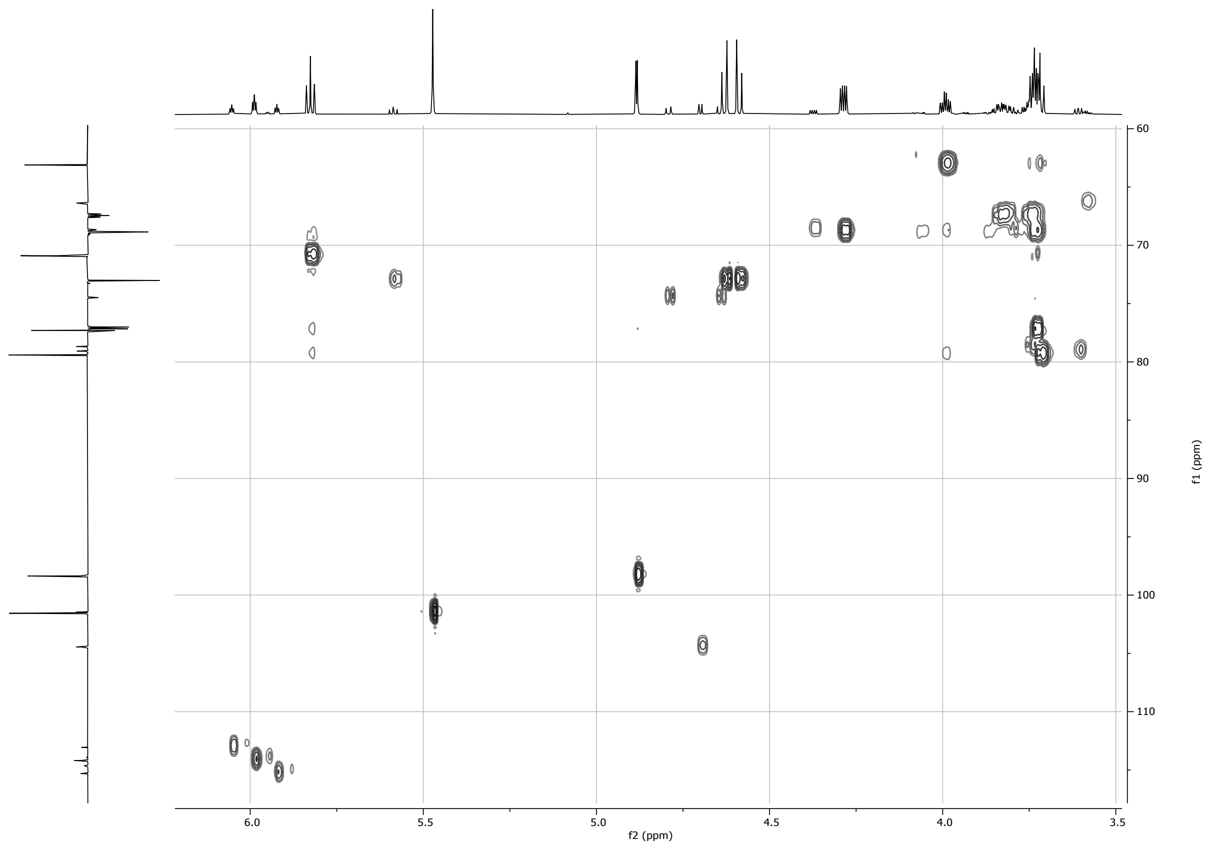

Supplementary Figure S69. HSQC{^{1}H} NMR, $CDCl_3$ of compound **S10**

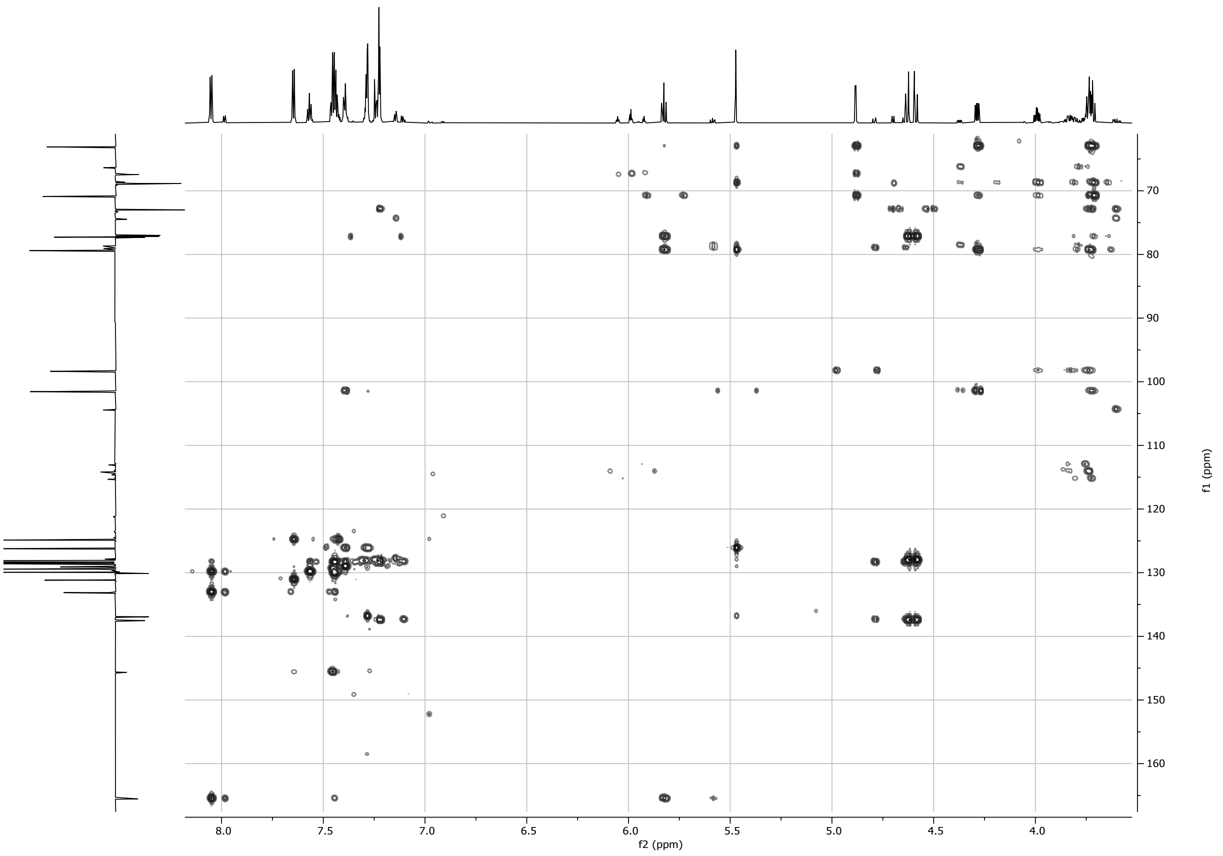

Supplementary Figure S70. HMBC NMR, $CDCl_3$ of compound **S10**

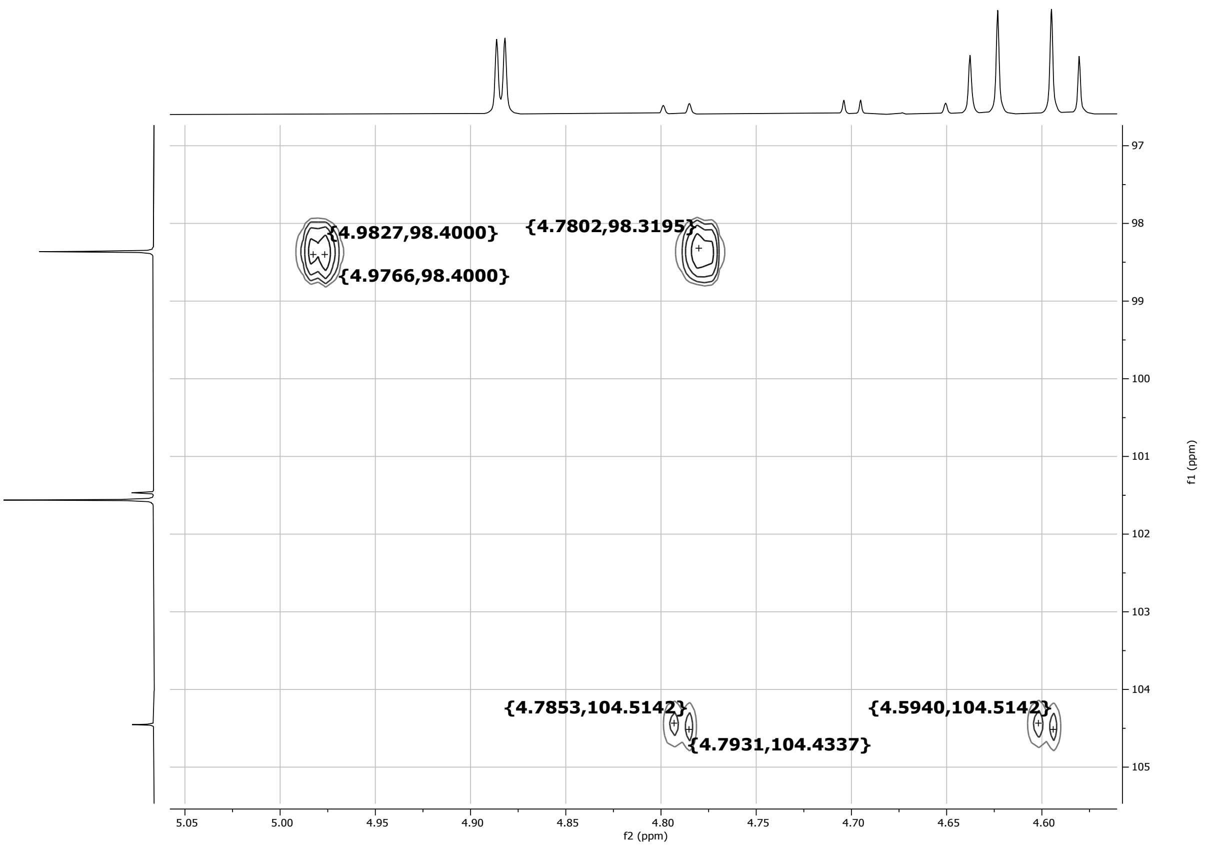

Supplementary Figure S71. HMBC-Gated NMR, $CDCl_3$ of compound **S10**

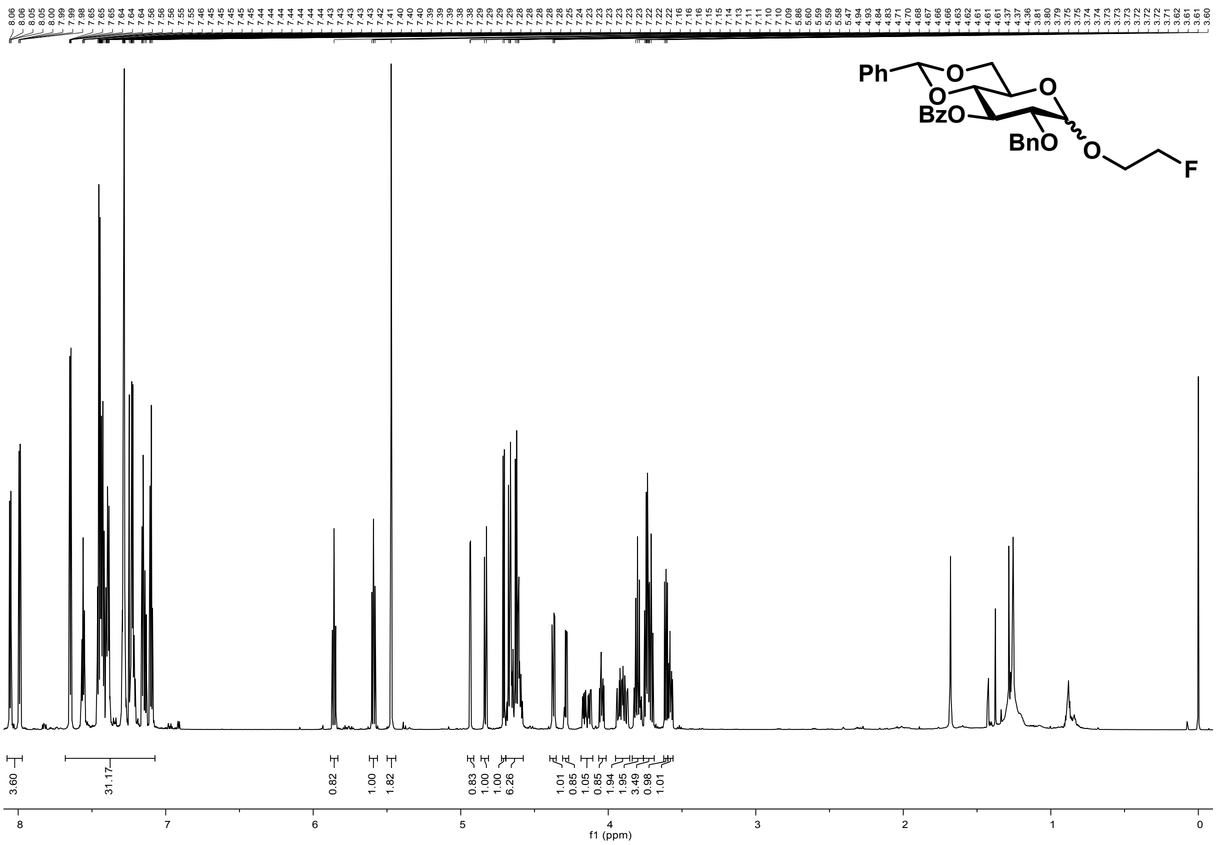

Supplementary Figure S72. ^{1}H NMR, 850 MHz, $CDCl_3$ of compound **S11**

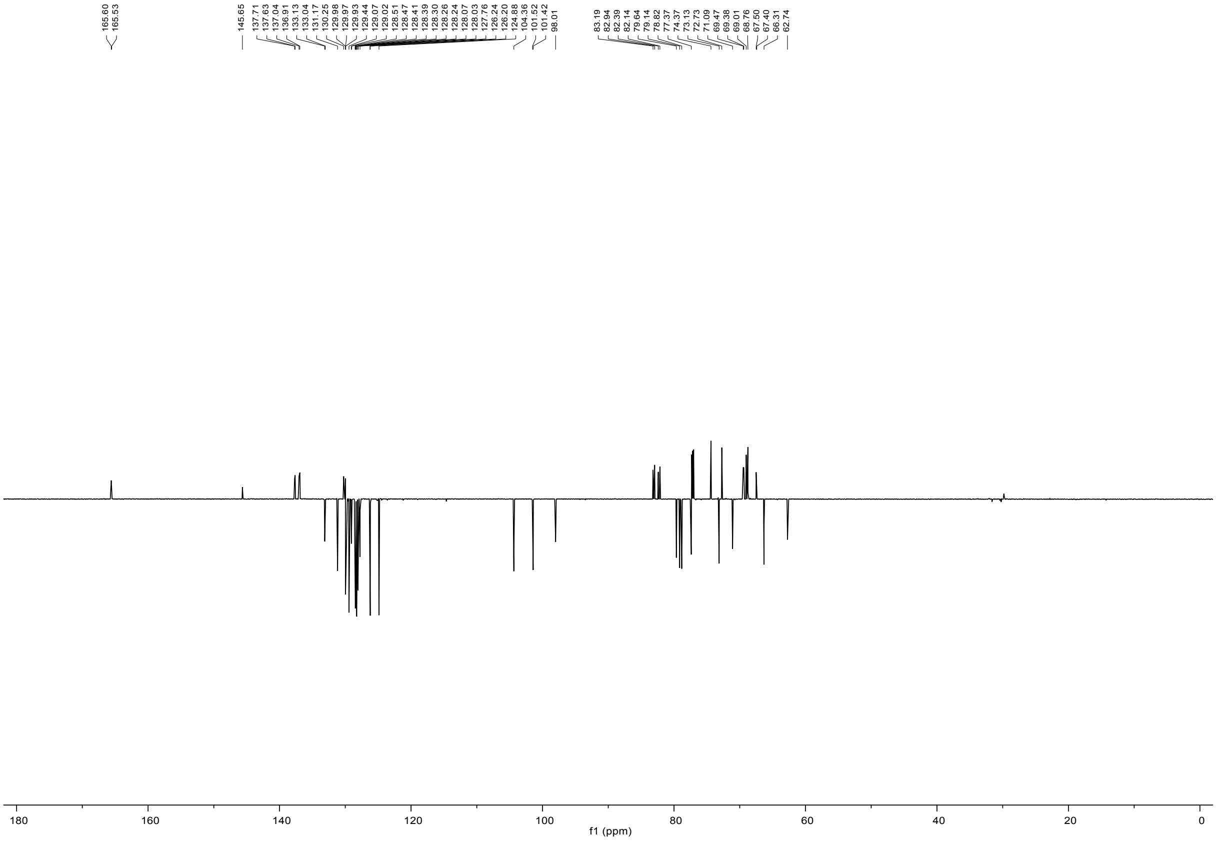

Supplementary Figure S73. $^{13}C\{^{1}H\}$ NMR, 214 MHz, $CDCl_3$ of compound **S11**

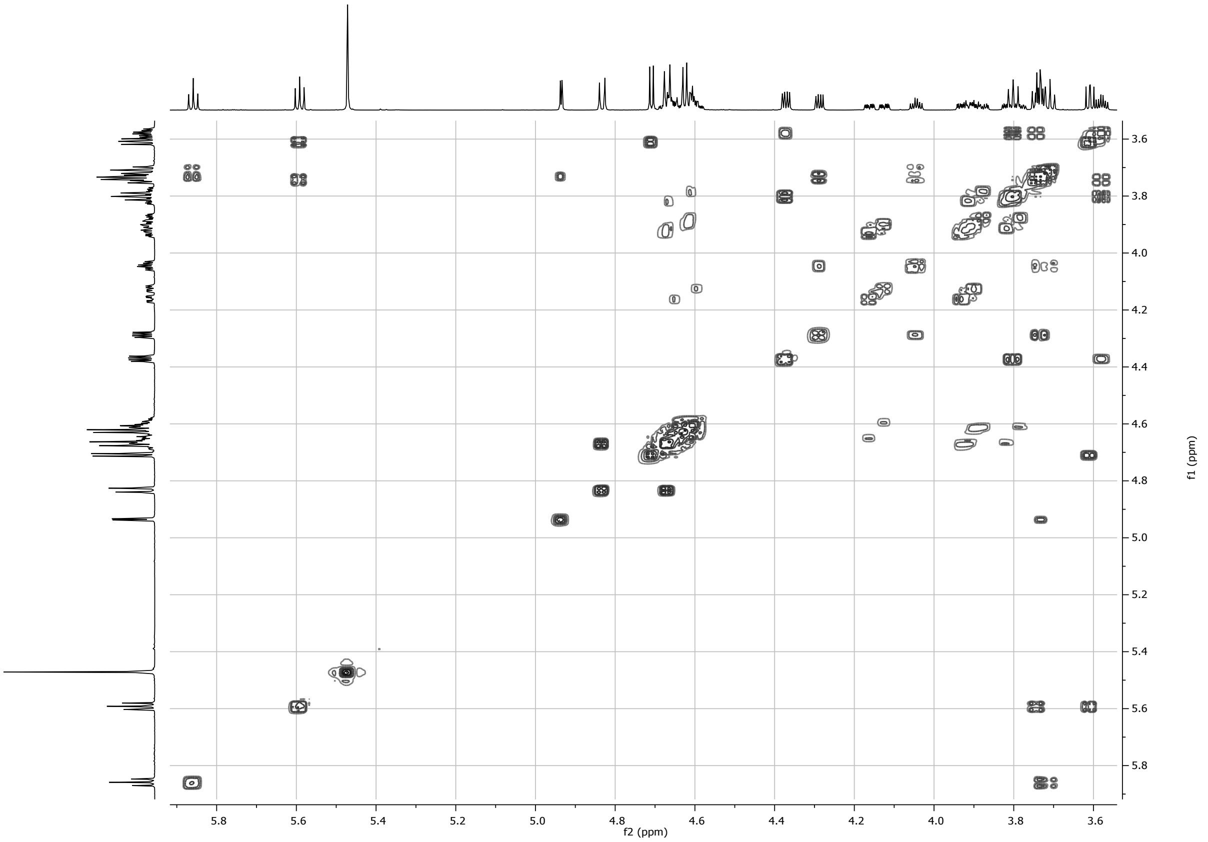

Supplementary Figure S74. HH-COSY NMR, $CDCl_3$ of compound **S11**

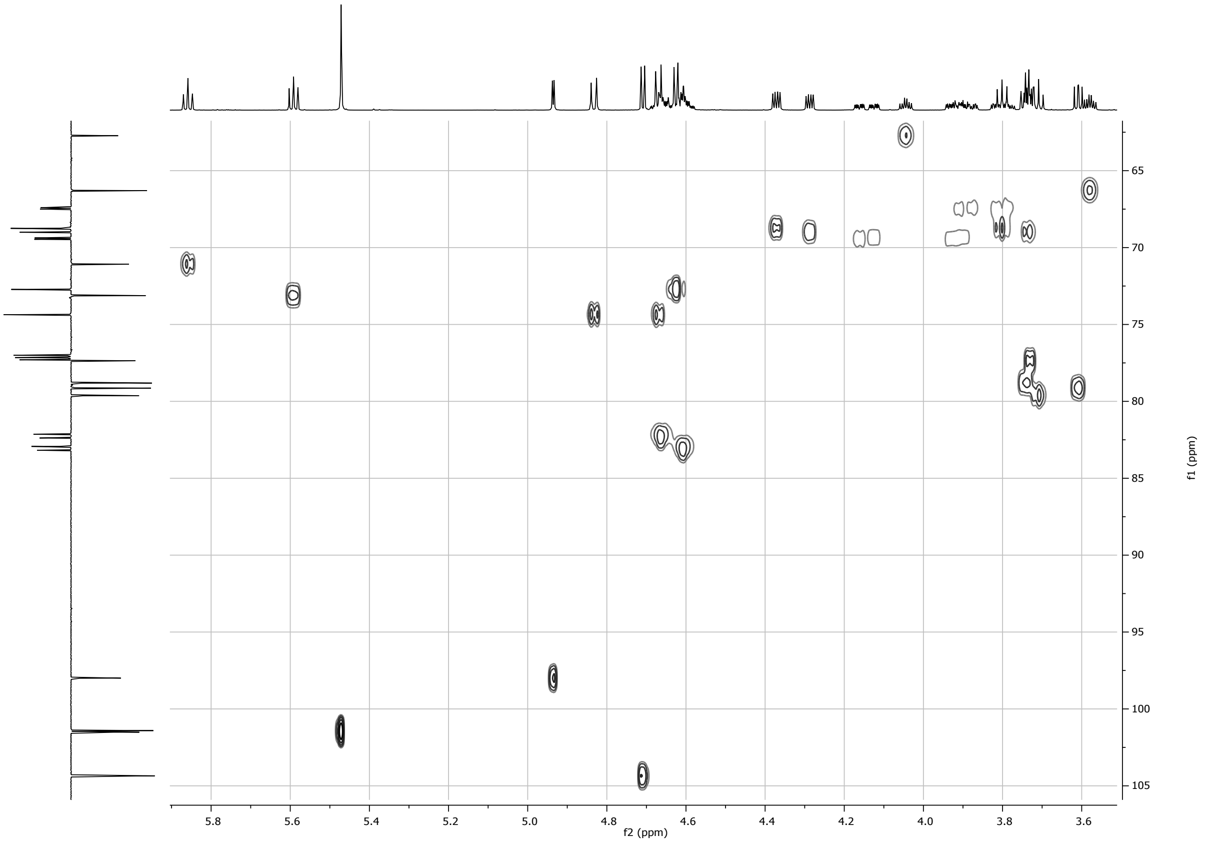

Supplementary Figure S75. HSQC{^{1}H} NMR, $CDCl_3$ of compound **S11**

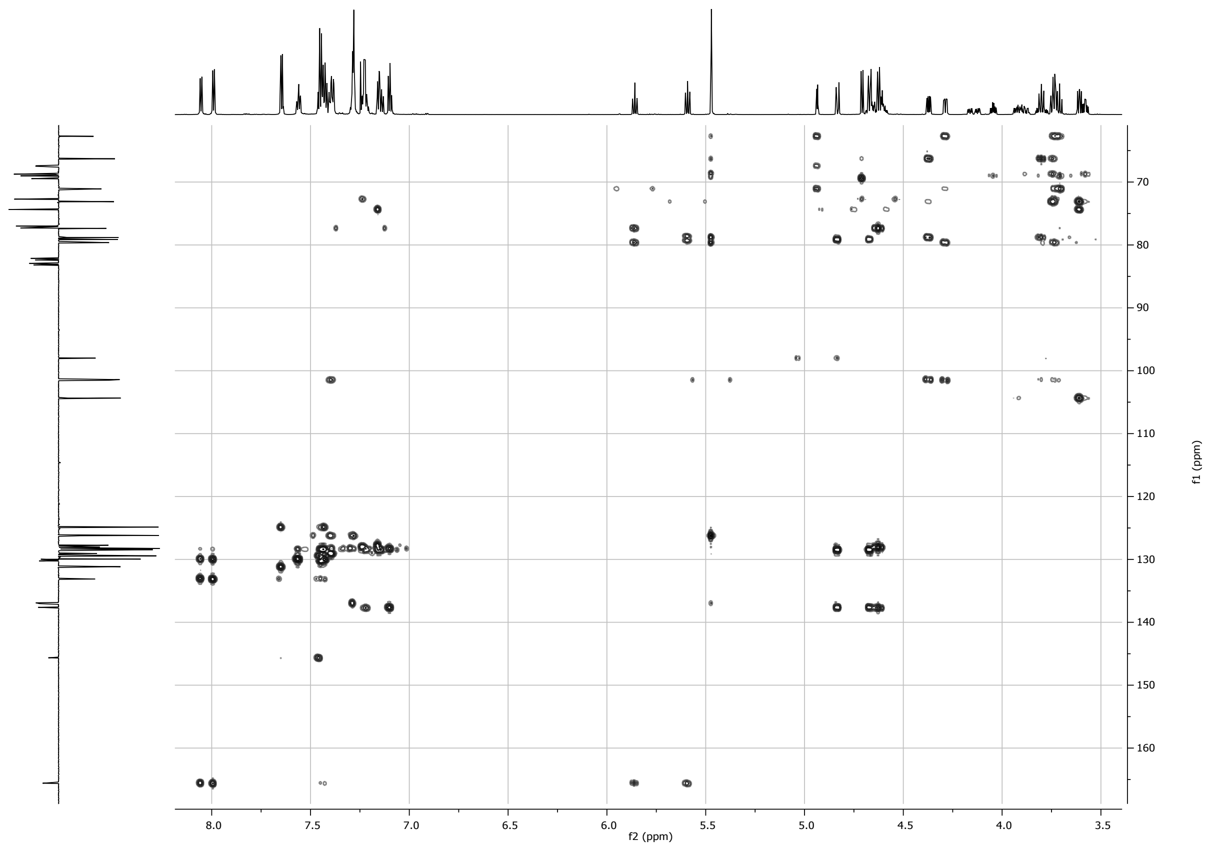

Supplementary Figure S76. HMBC NMR, $CDCl_3$ of compound **S11**

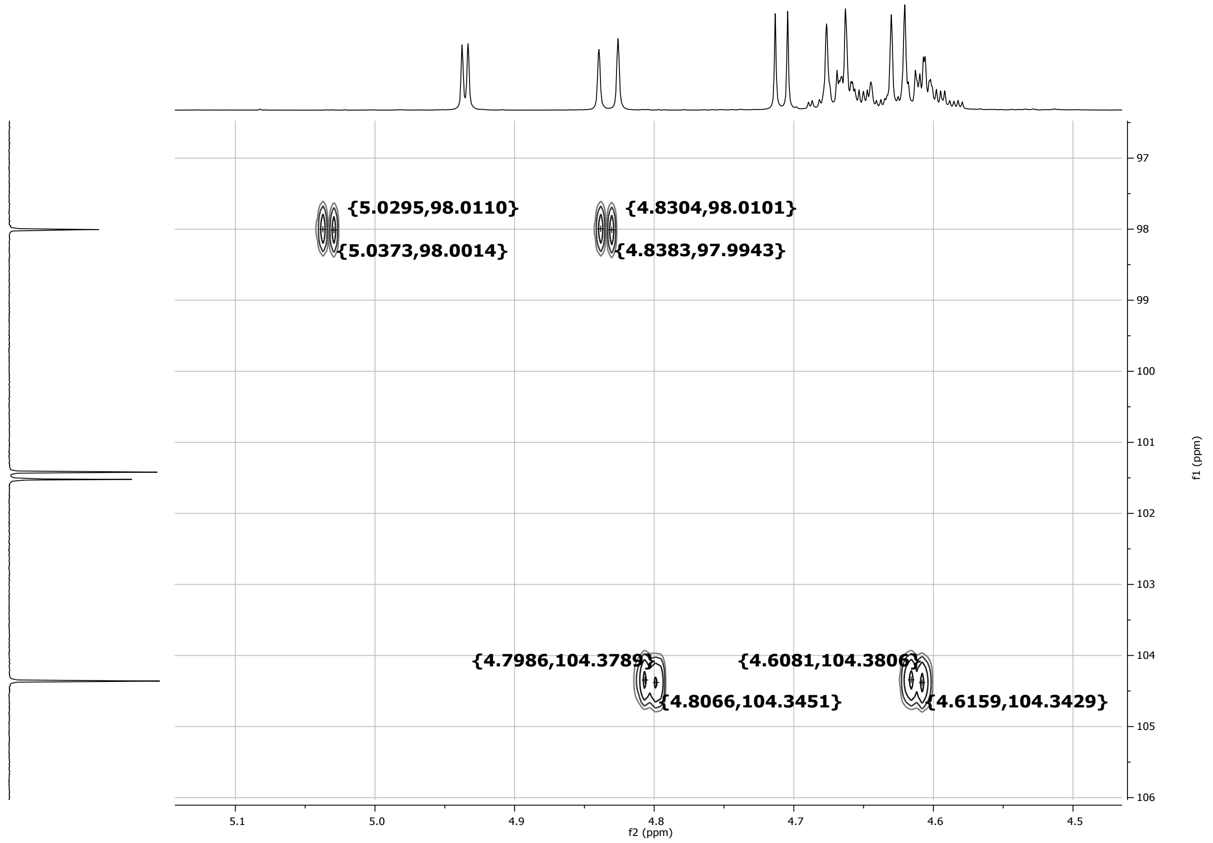

Supplementary Figure S77. HMBC-Gated NMR, $CDCl_3$ of compound **S11**

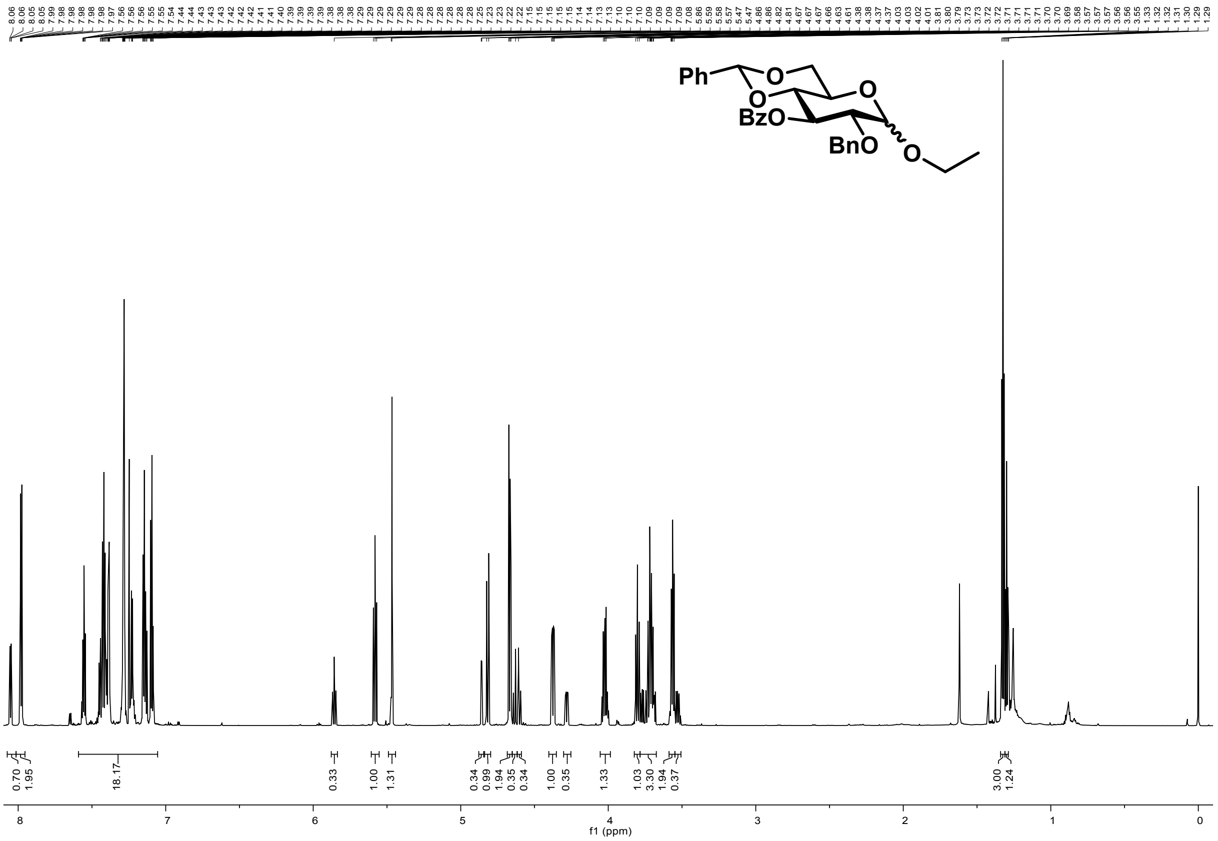

Supplementary Figure S78. ^{1}H NMR, 850 MHz, $CDCl_3$ of compound **S12**

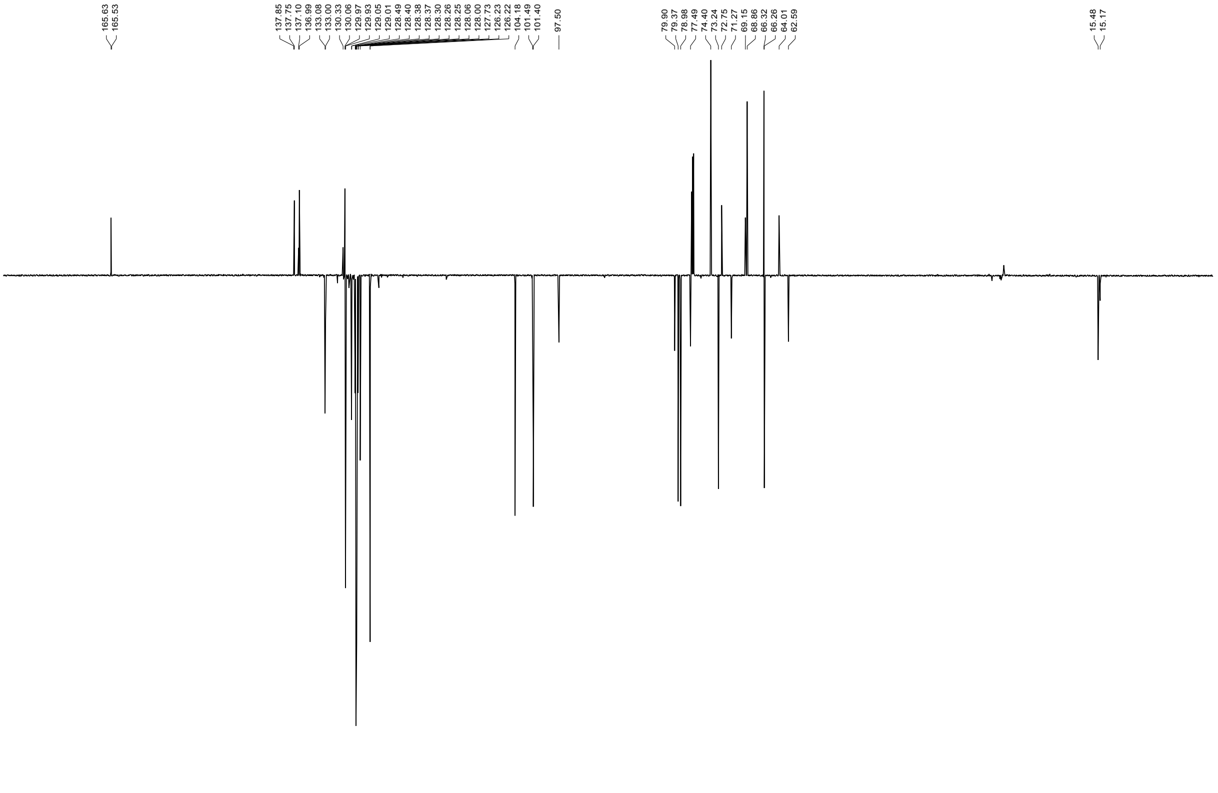

Supplementary Figure S79. $^{13}C\{^{1}H\}$ NMR, 214 MHz, $CDCl_3$ of compound **S12**

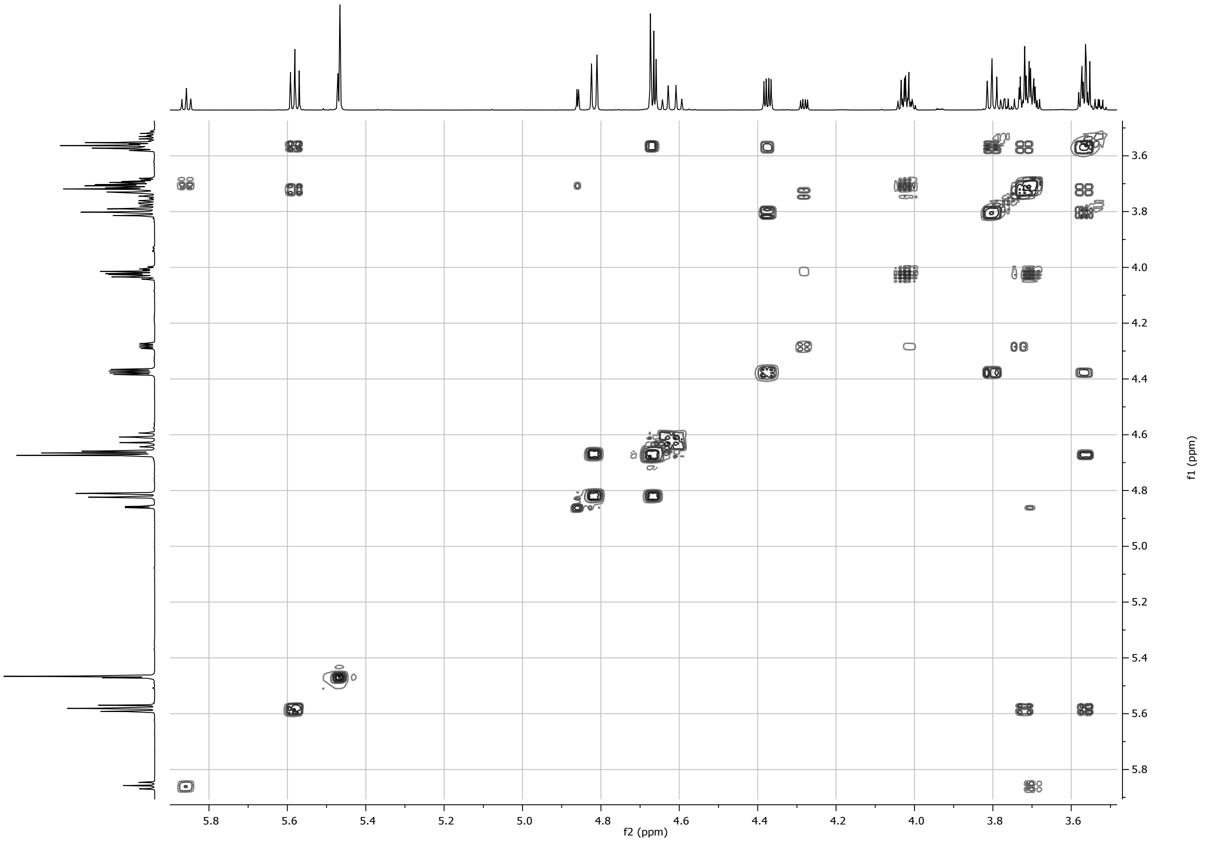

Supplementary Figure S80. HH-COSY NMR, $CDCl_3$ of compound **S12**

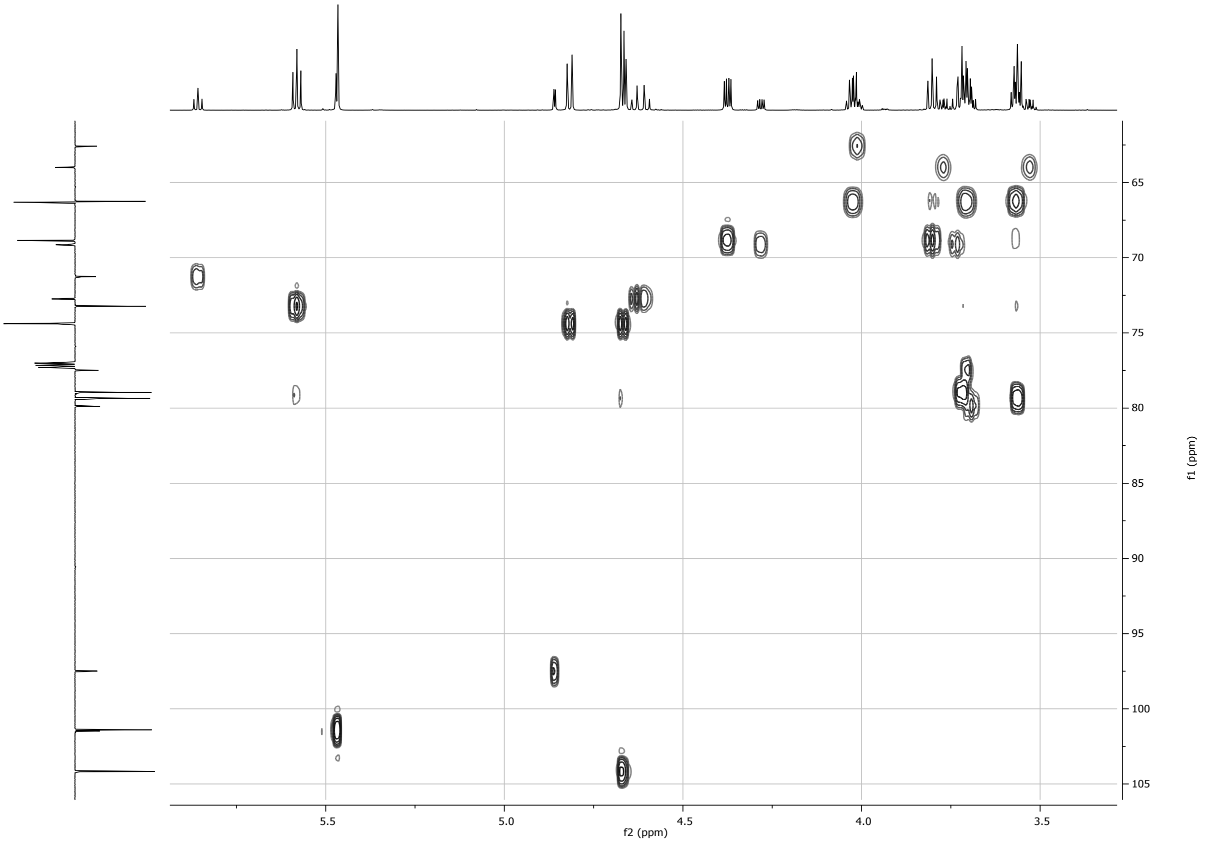

Supplementary Figure S81. HSQC{^{1}H} NMR, $CDCl_3$ of compound **S12**

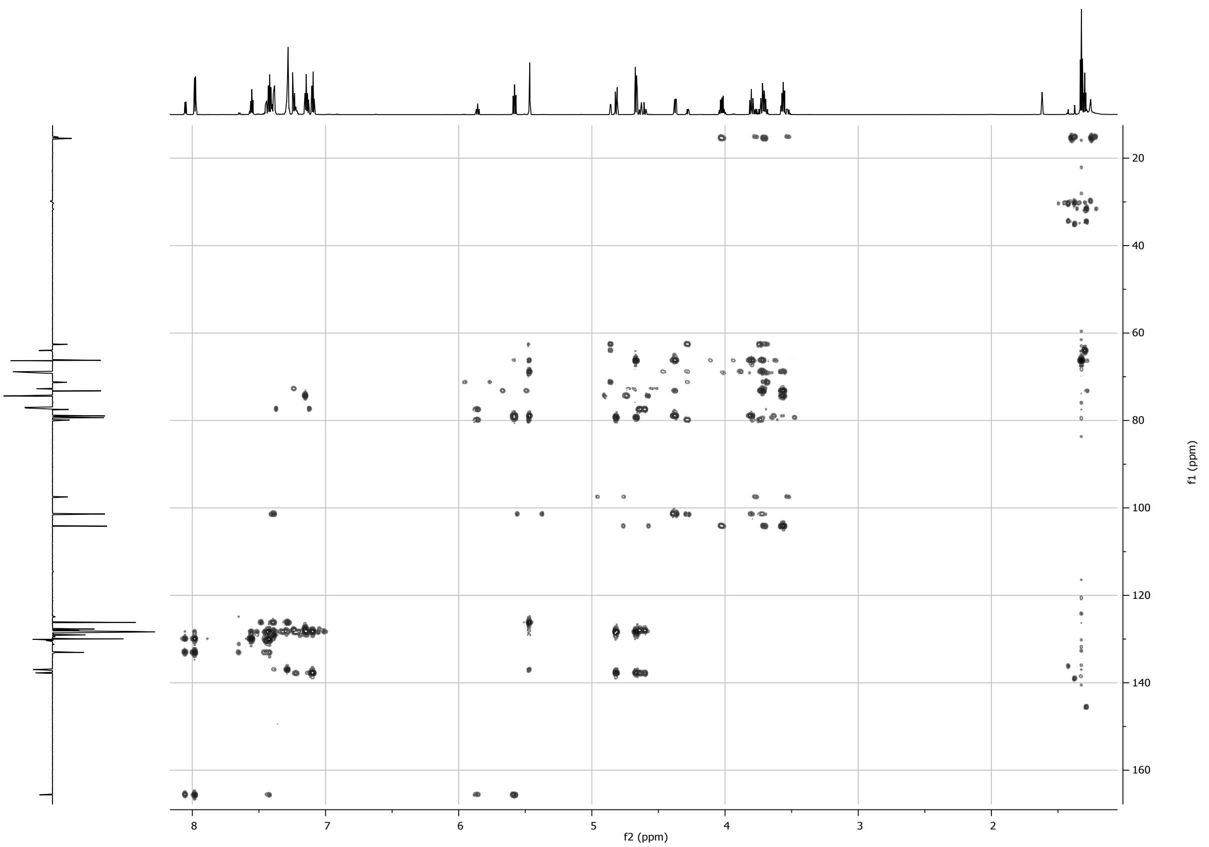

Supplementary Figure S82. HMBC NMR, $CDCl_3$ of compound **S12**

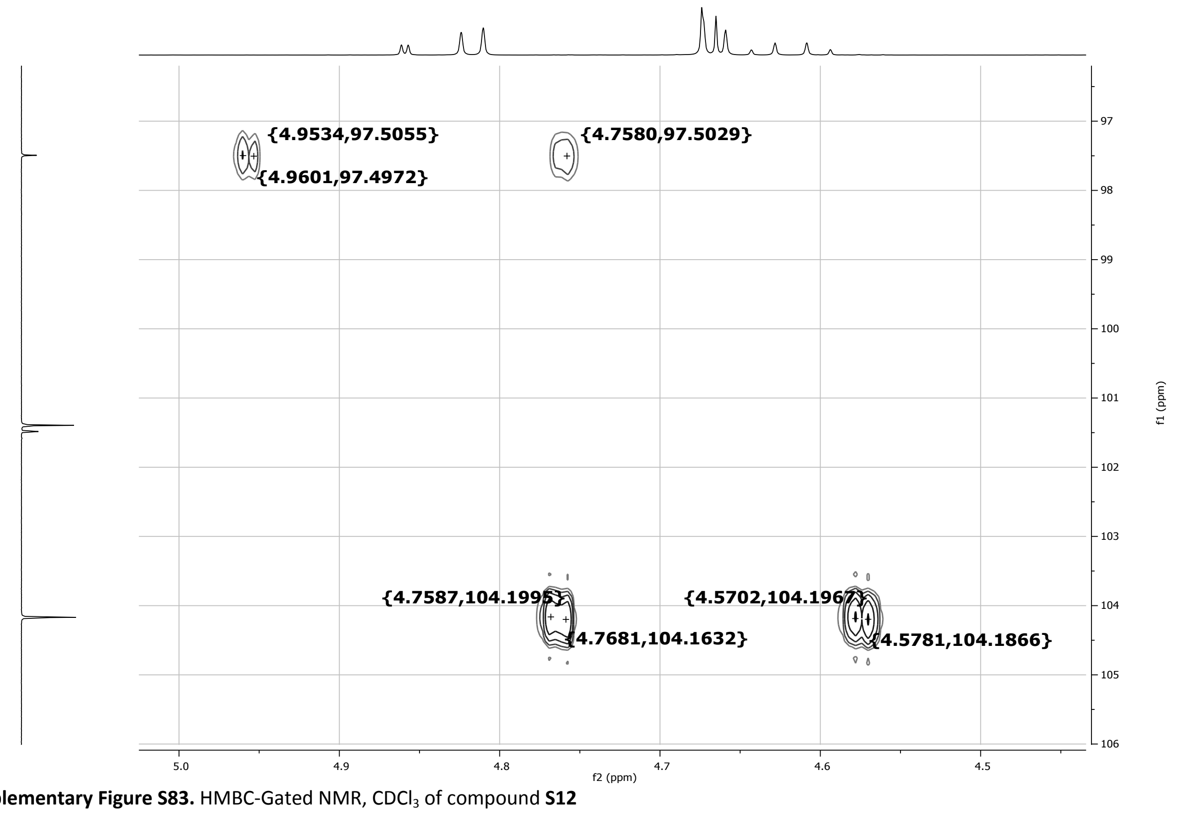

Supplementary Figure S83. HMBC-Gated NMR, $CDCl_3$ of compound **S12**

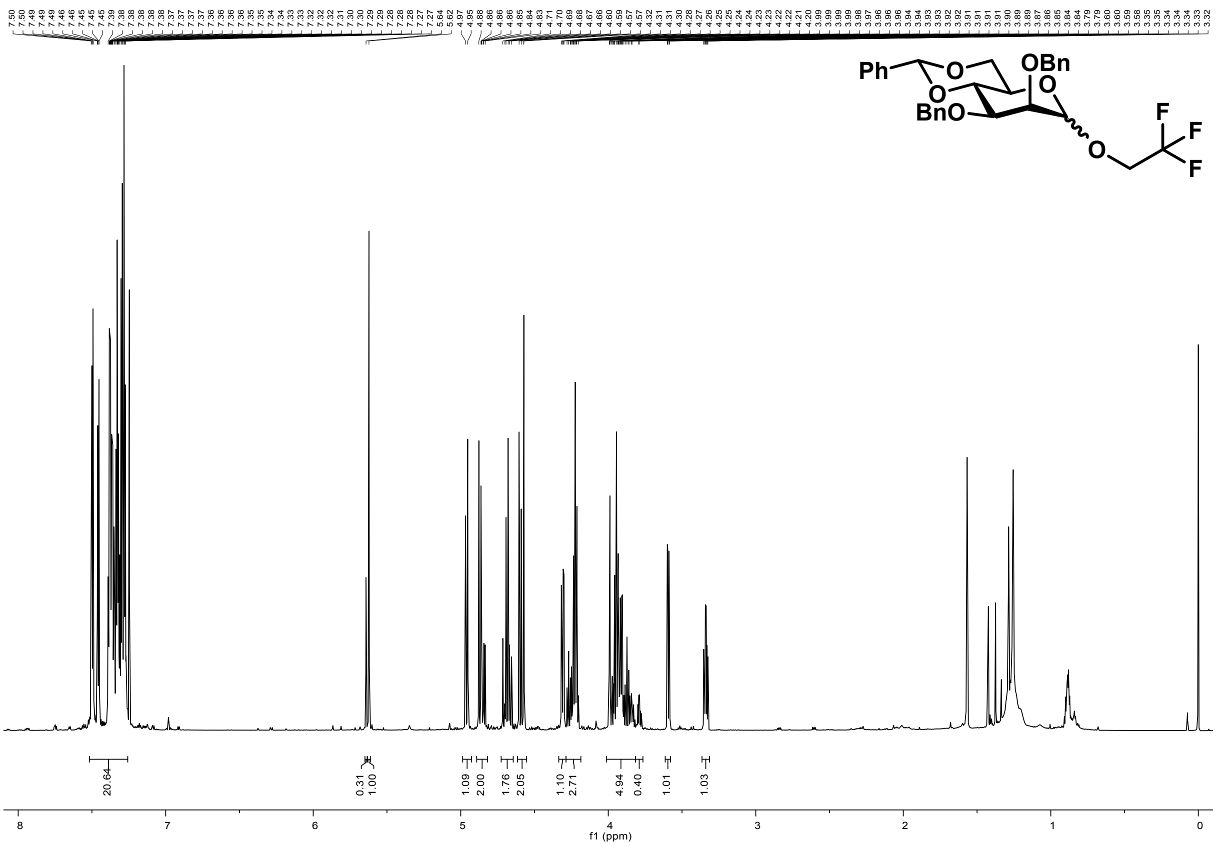

Supplementary Figure S84. ^{1}H NMR, 850 MHz, $CDCl_3$ of compound **S13**

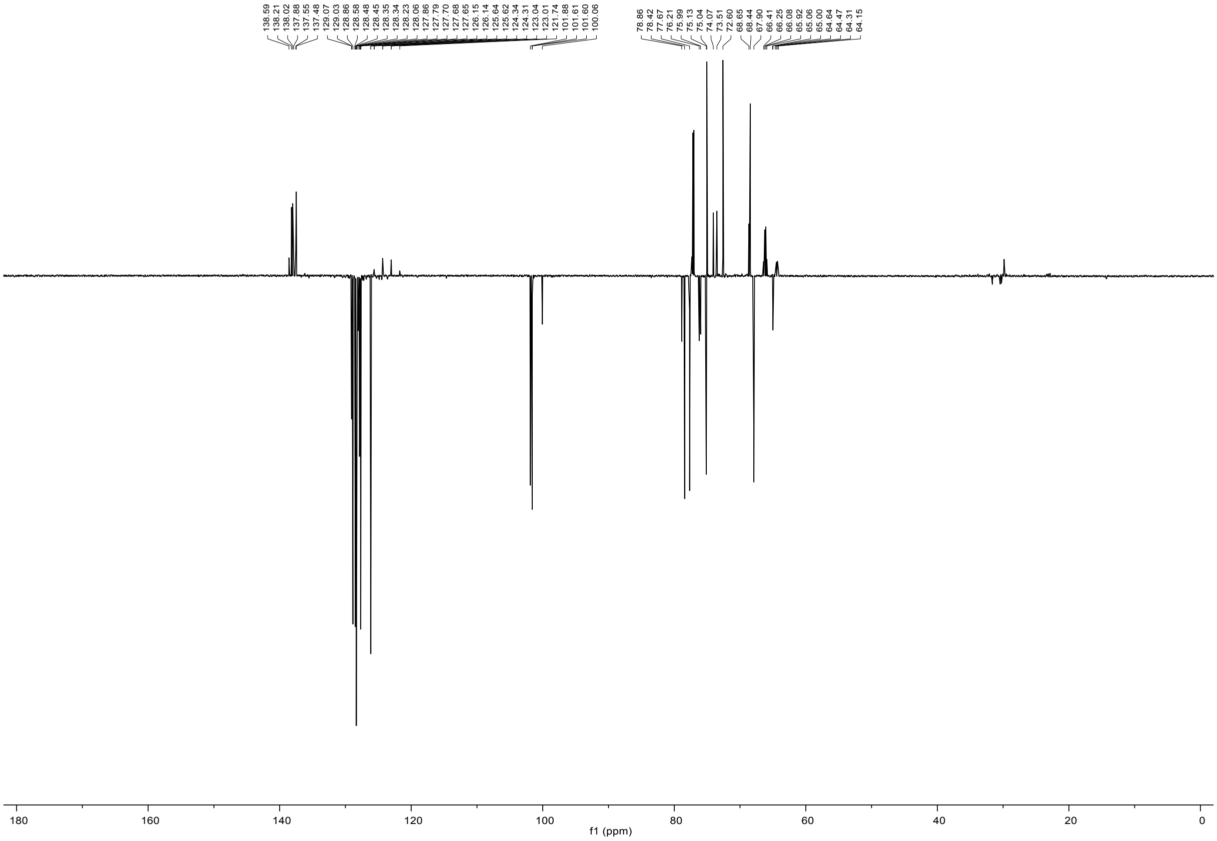

Supplementary Figure S85. $^{13}C\{^{1}H\}$ NMR, 214 MHz, $CDCl_3$ of compound **S13**

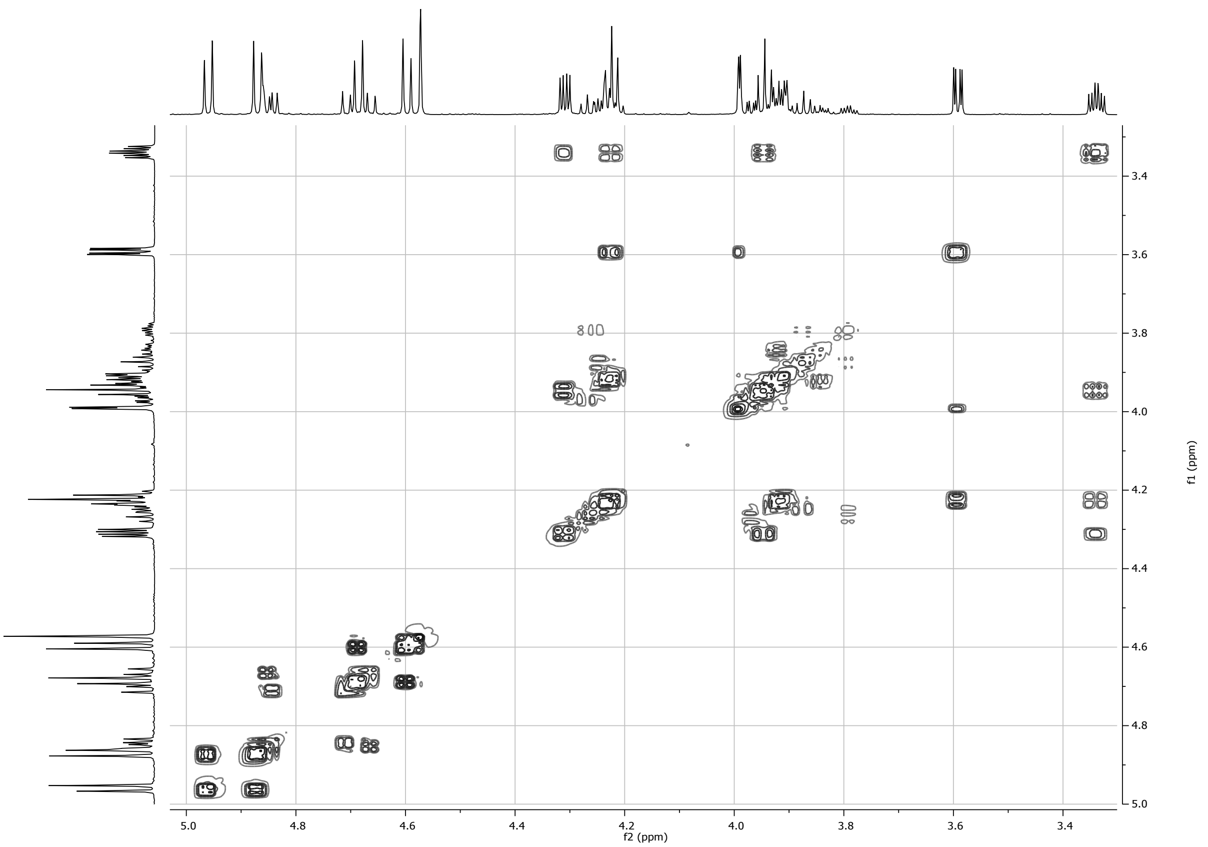

Supplementary Figure S86. HH-COSY NMR, $CDCl_3$ of compound **S13**

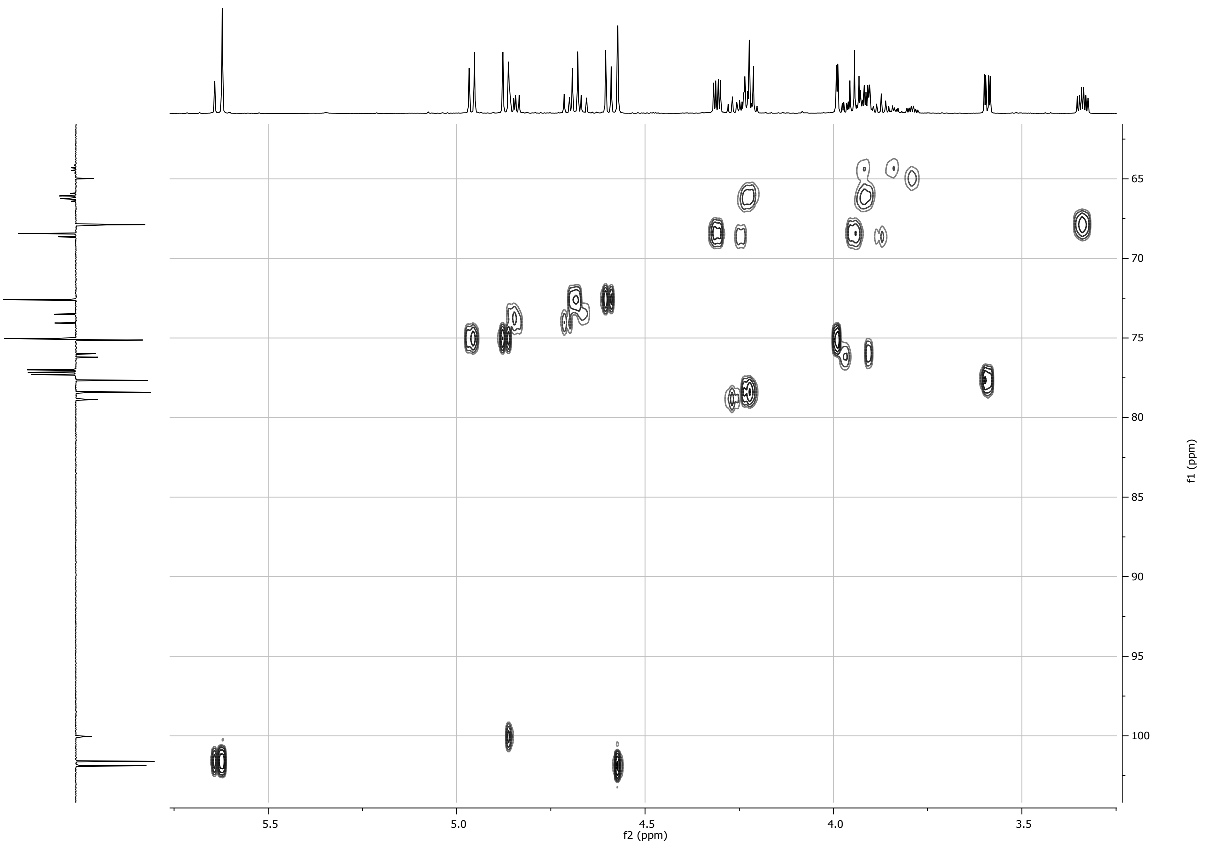

Supplementary Figure S87. HSQC{^{1}H} NMR, $CDCl_3$ of compound **S13**

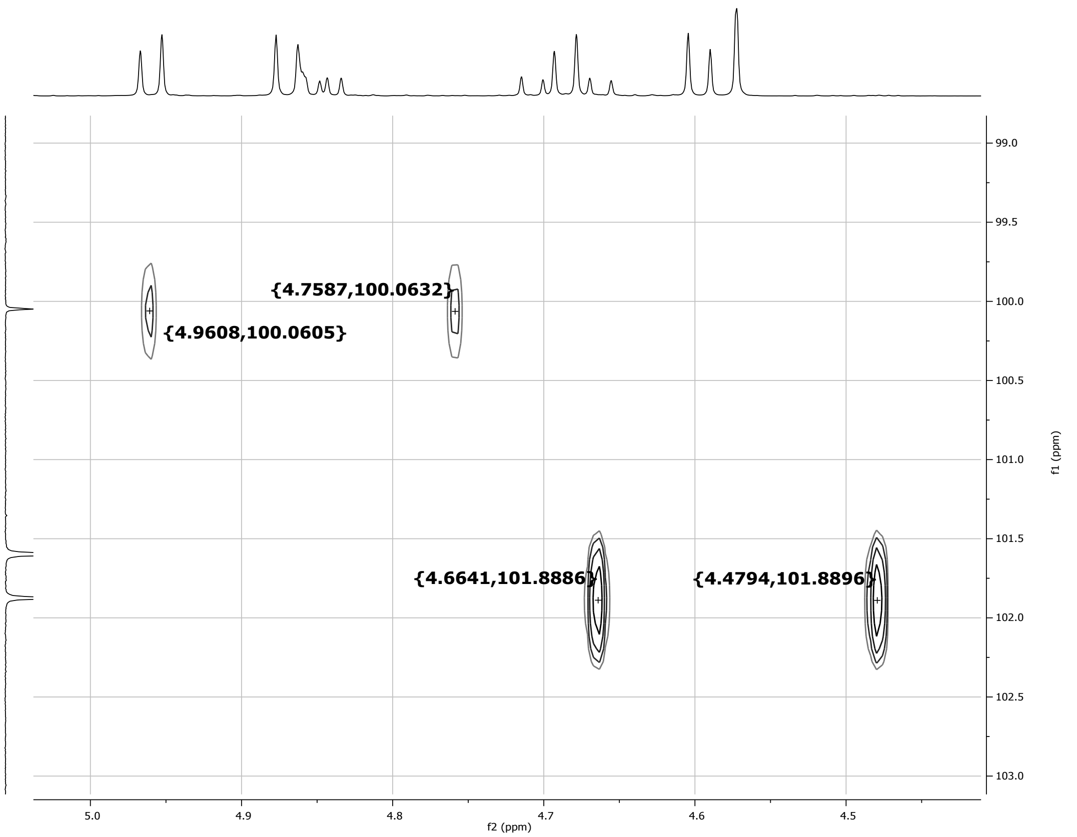

Supplementary Figure S88. HMBC-Gated NMR, $CDCl_3$ of compound **S13**

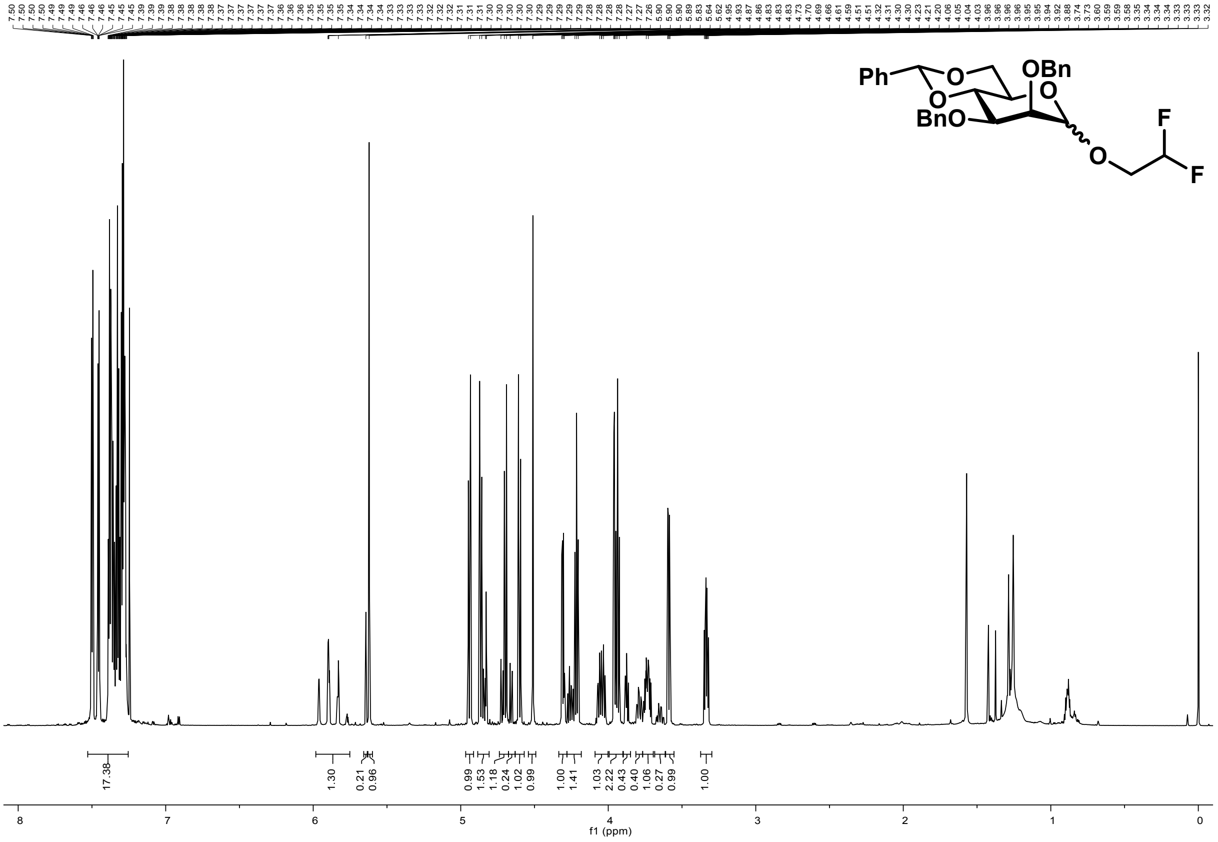

Supplementary Figure S89. ^1H NMR, 850 MHz, $CDCl_3$ of compound **S14**

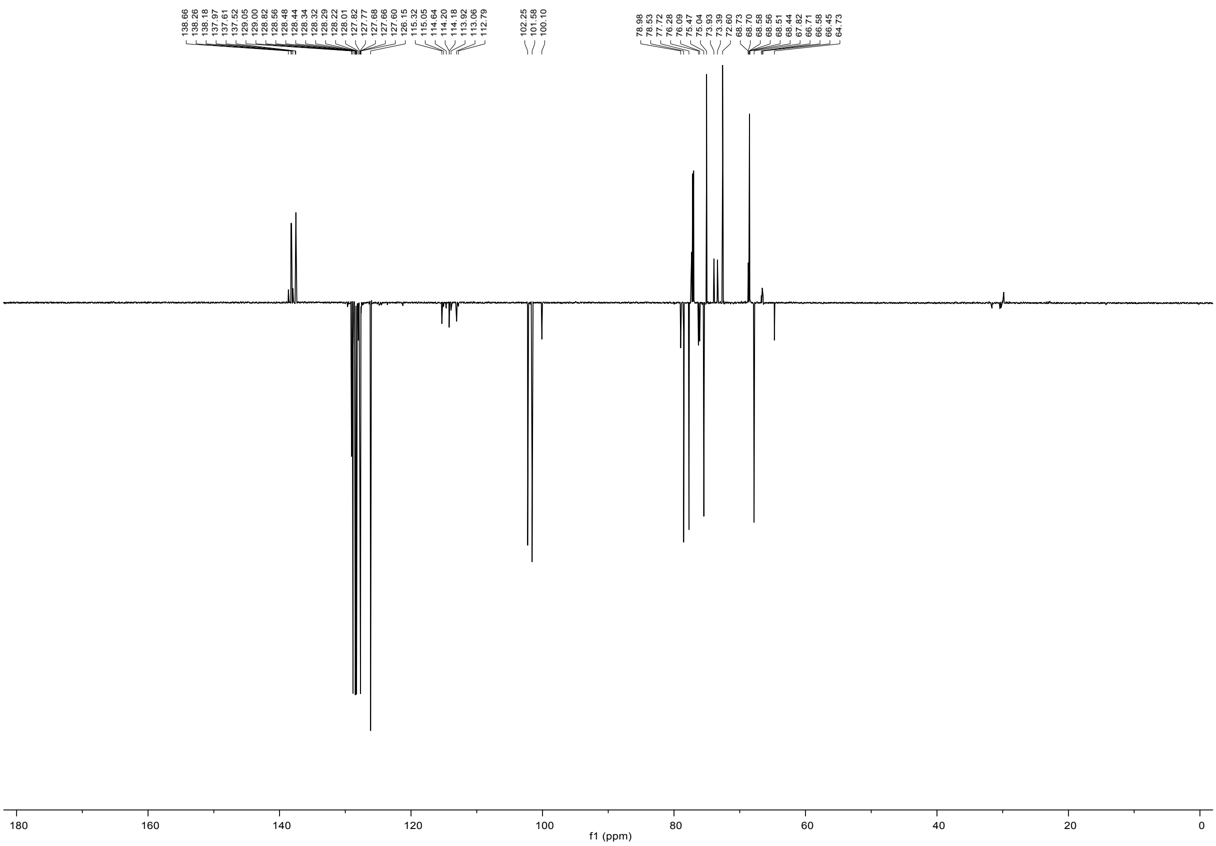

Supplementary Figure S90. $^{13}C\{^{1}H\}$ NMR, 214 MHz, $CDCl_3$ of compound **S14**

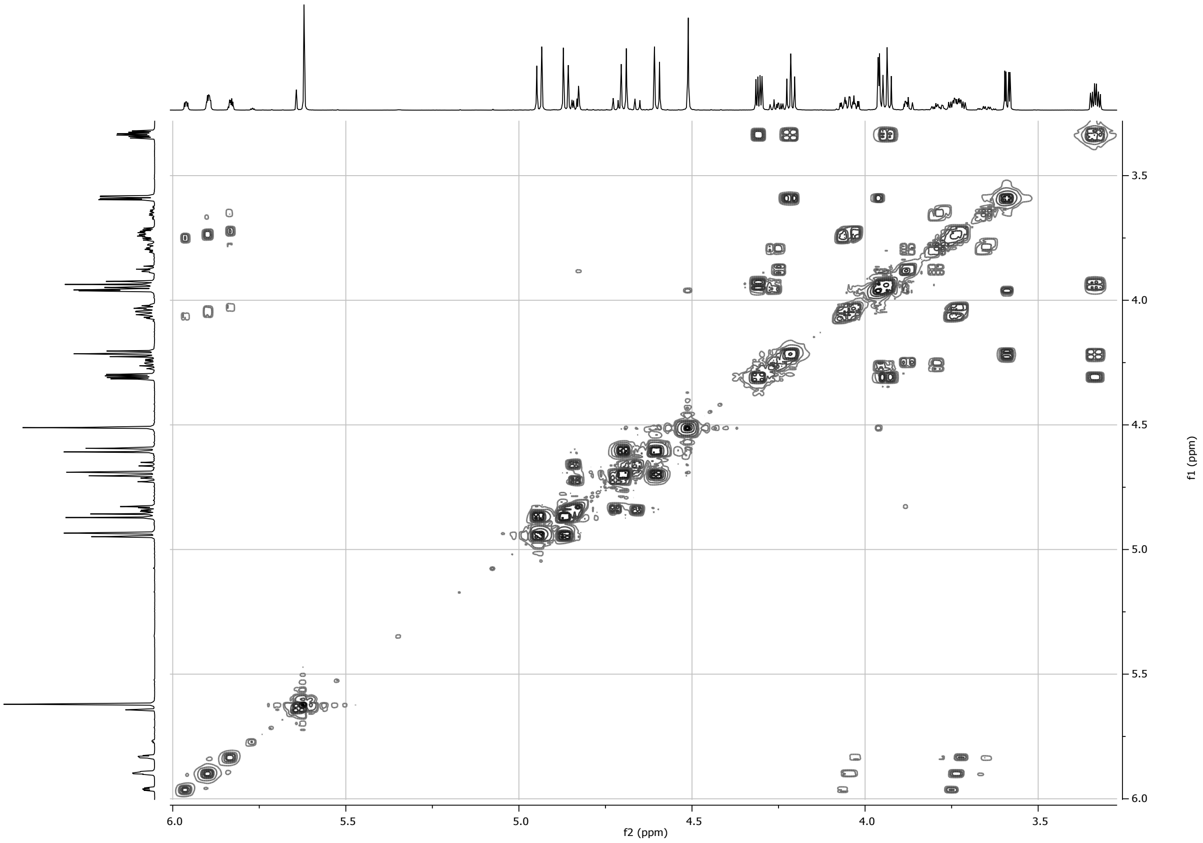

Supplementary Figure S91. HH-COSY NMR, $CDCl_3$ of compound **S14**

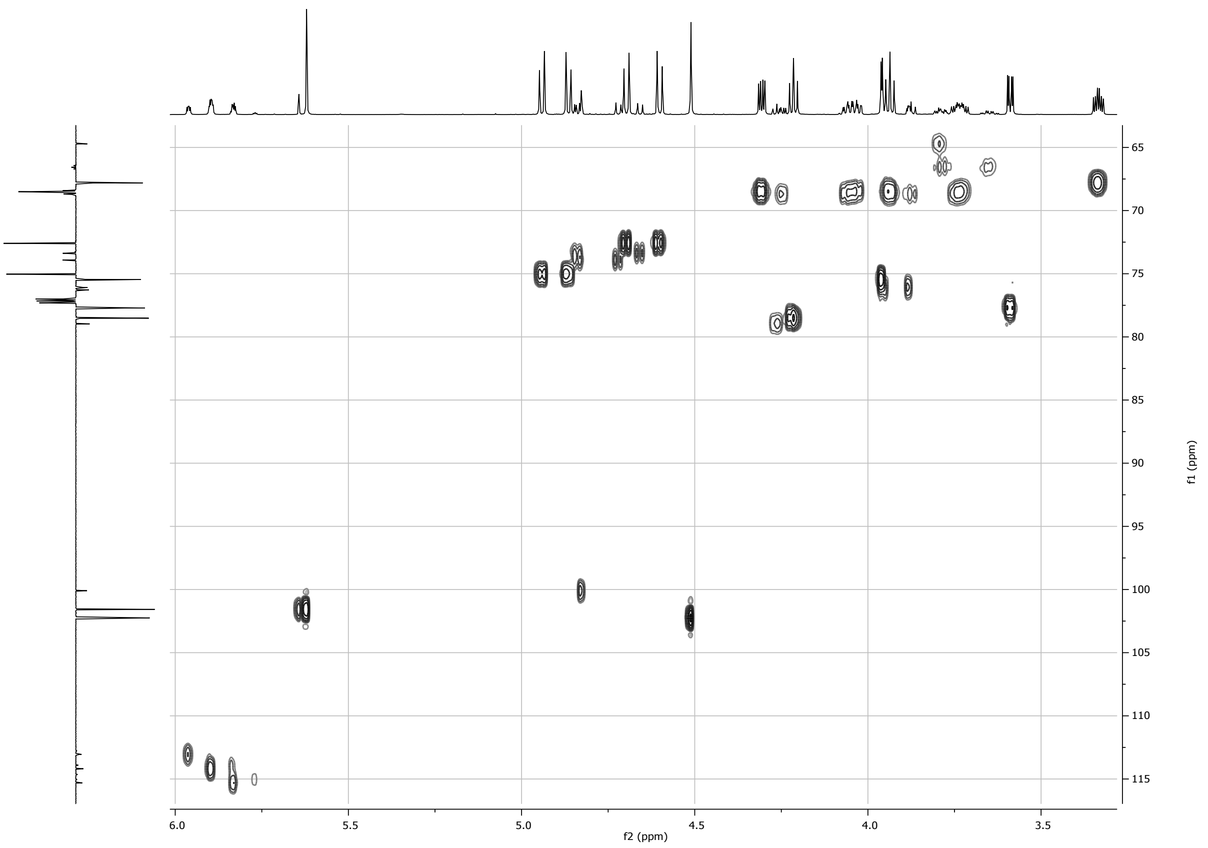

Supplementary Figure S92. HSQC{^{1}H} NMR, $CDCl_3$ of compound **S14**

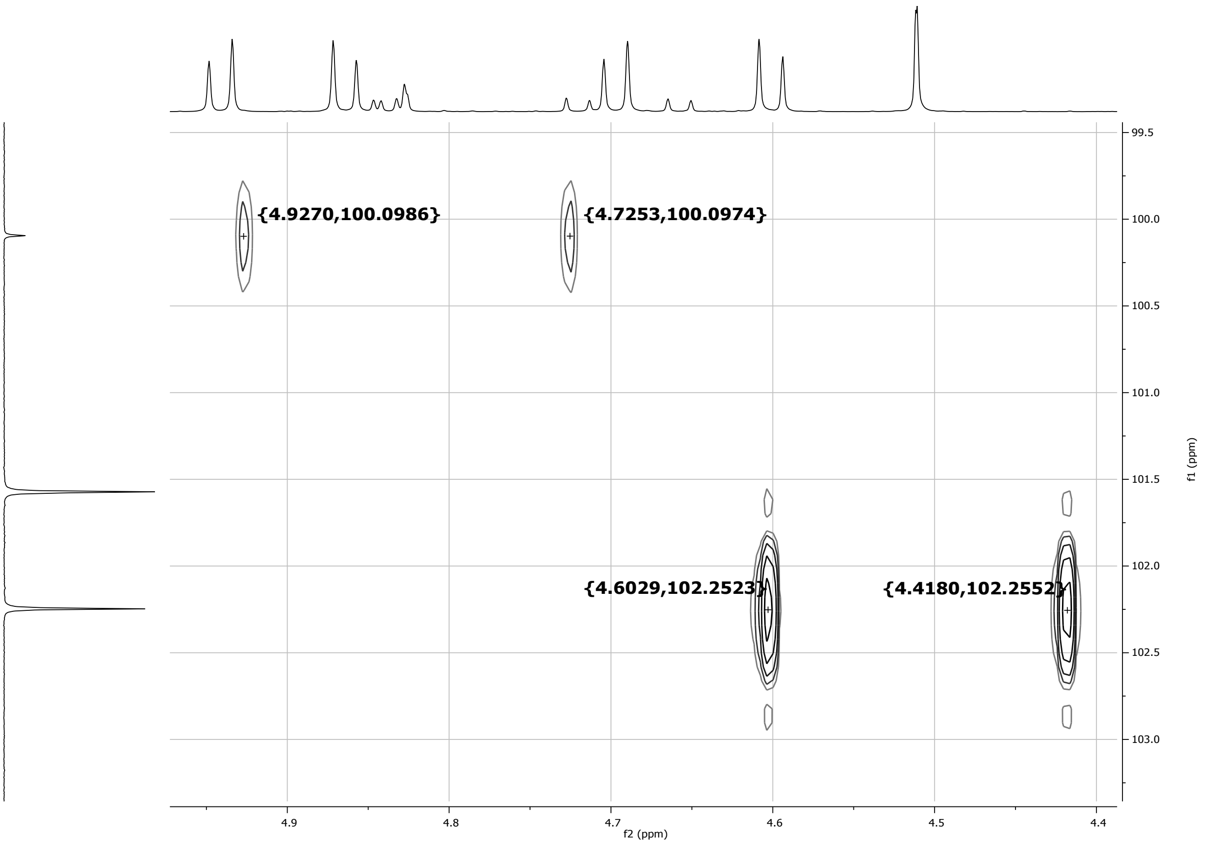

Supplementary Figure S93. HMBC-Gated NMR, $CDCl_3$ of compound **S14**

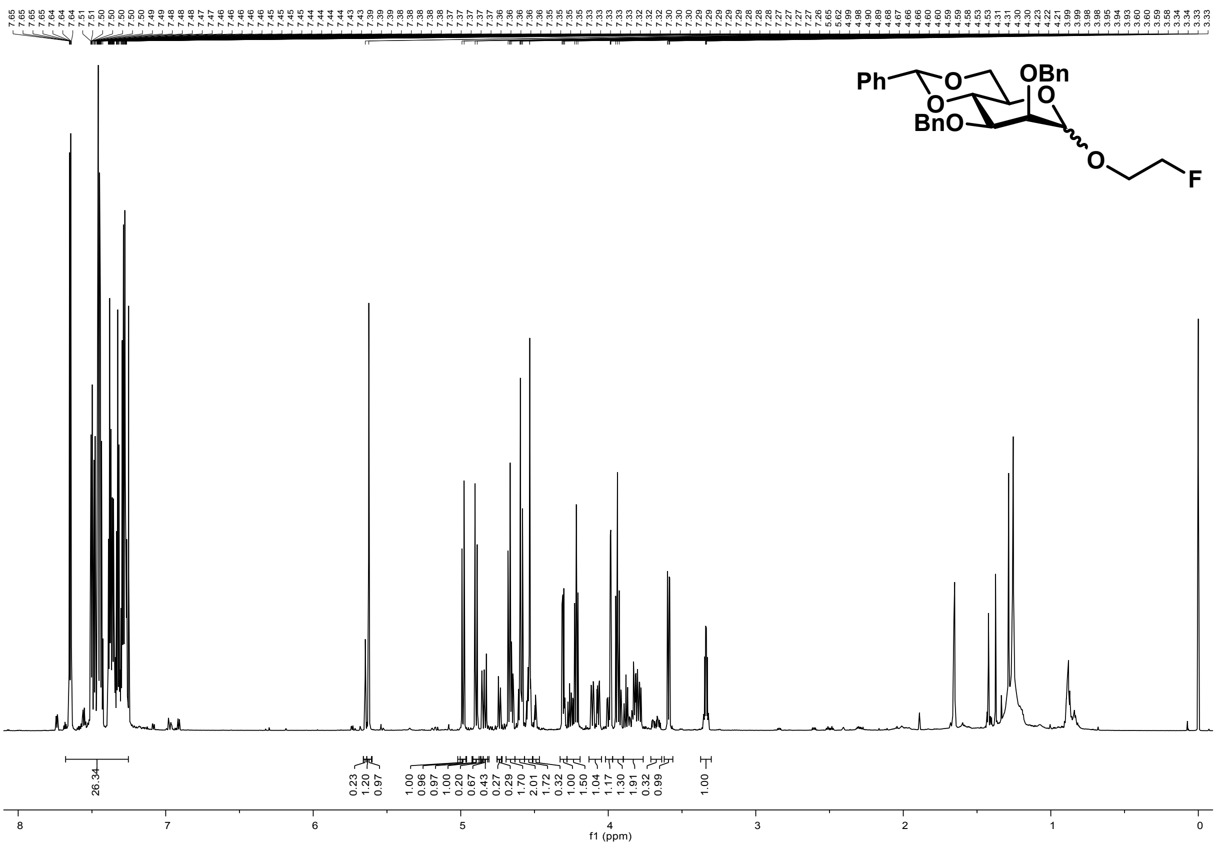

Supplementary Figure S94. ^{1}H NMR, 850 MHz, $CDCl_3$ of compound **S15**

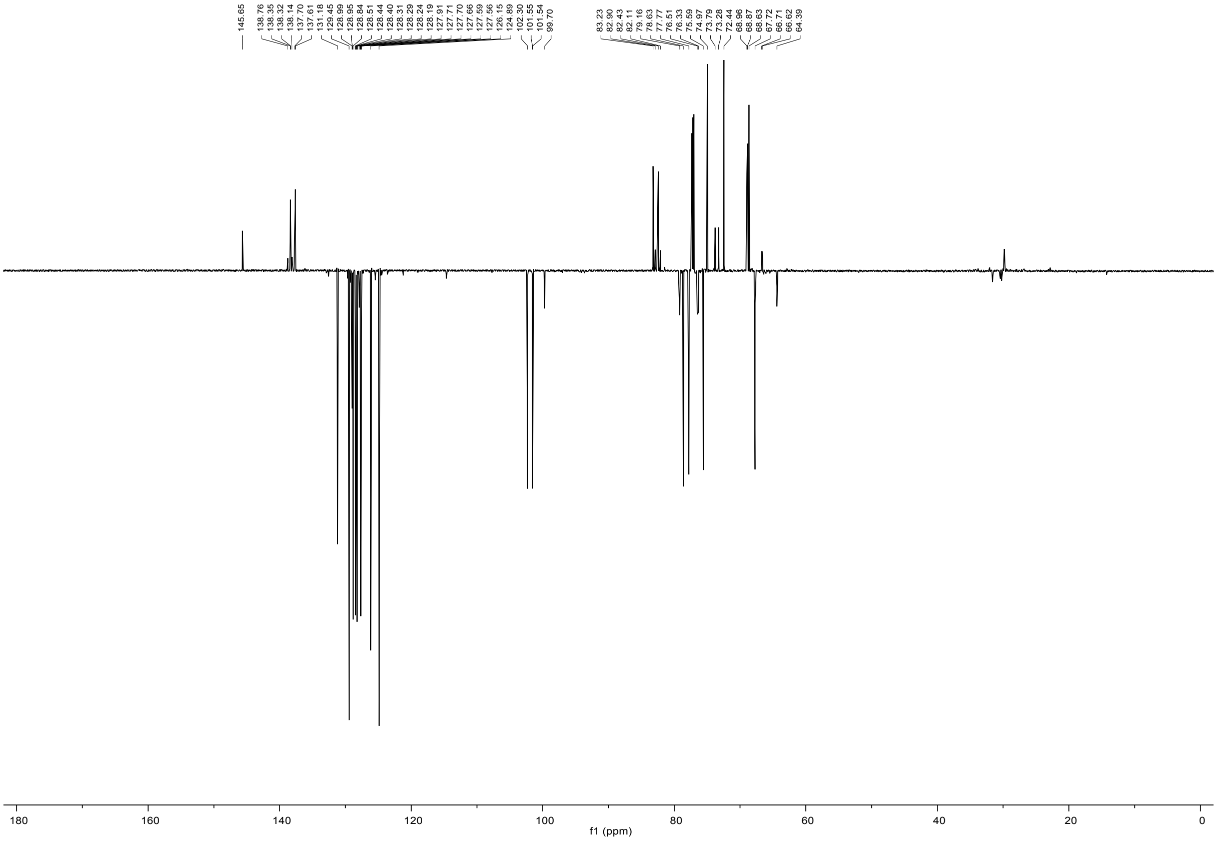

Supplementary Figure S95. $^{13}C\{^{1}H\}$ NMR, 214 MHz, $CDCl_3$ of compound **S15**

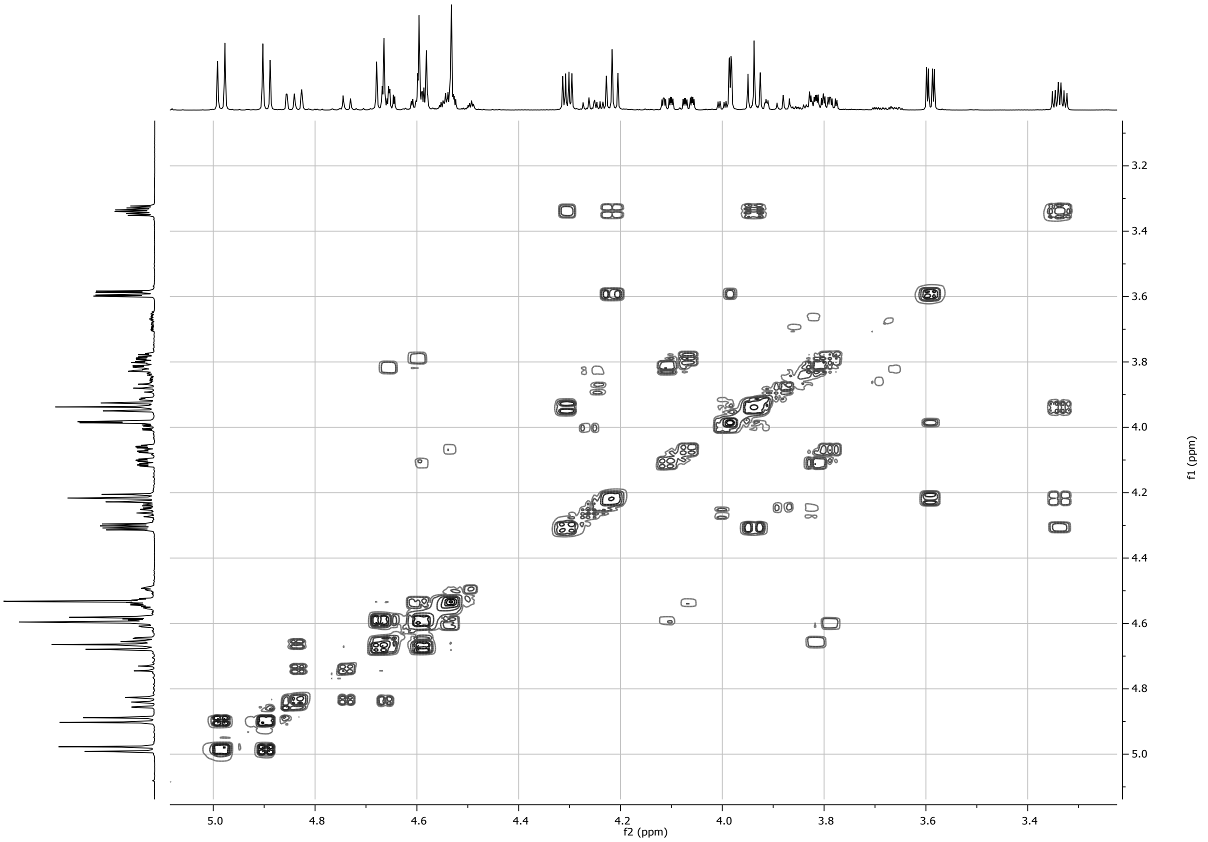

Supplementary Figure S96. HH-COSY NMR, $CDCl_3$ of compound **S15**

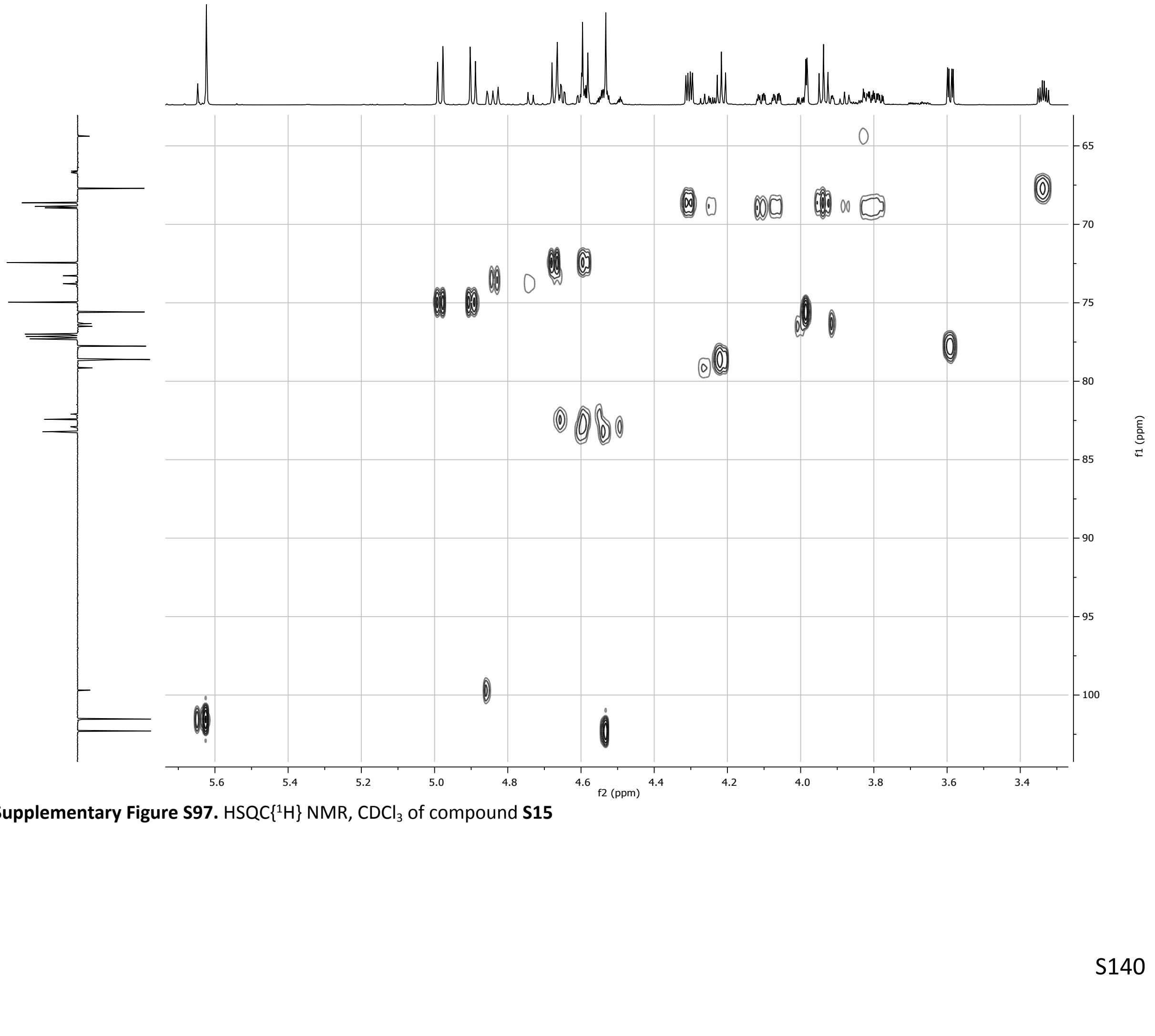

Supplementary Figure S97. HSQC{^{1}H} NMR, $CDCl_3$ of compound **S15**

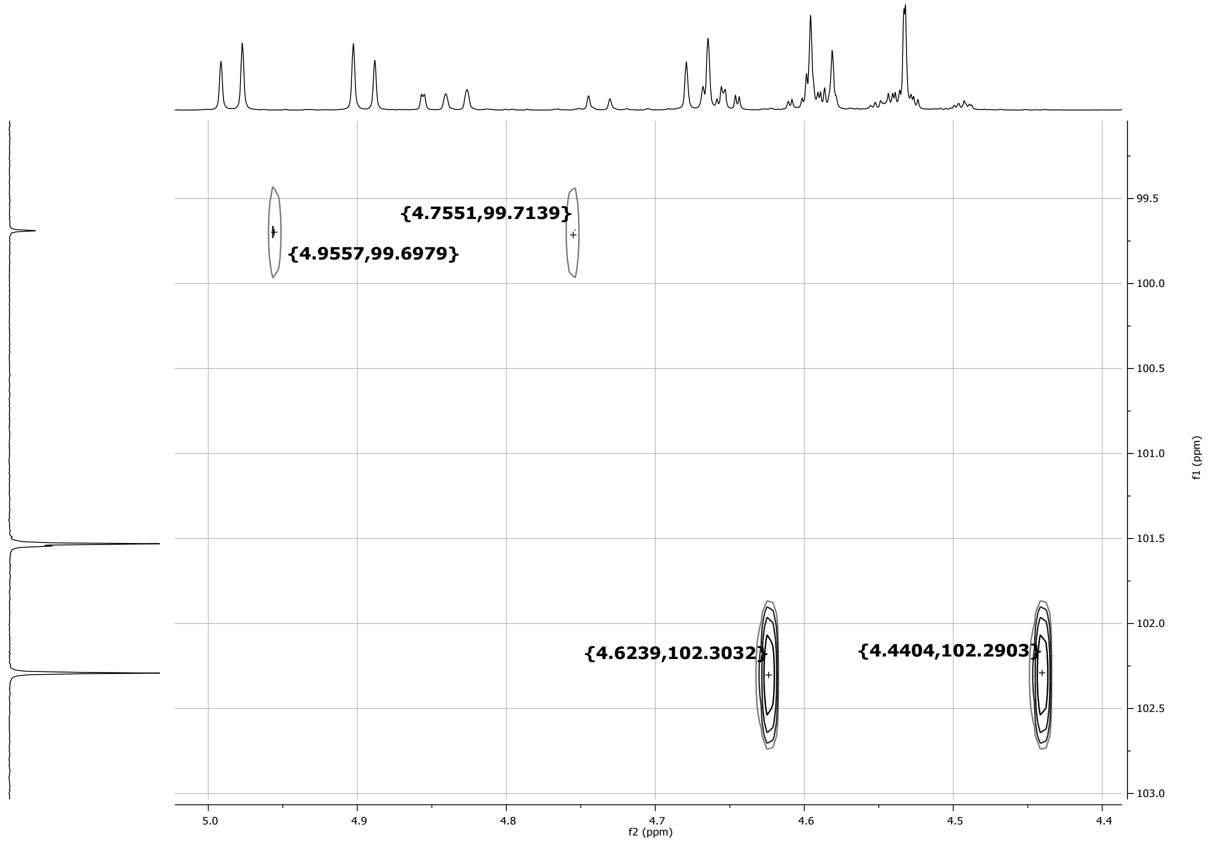

Supplementary Figure S98. HMBC-Gated NMR, $CDCl_3$ of compound **S15**

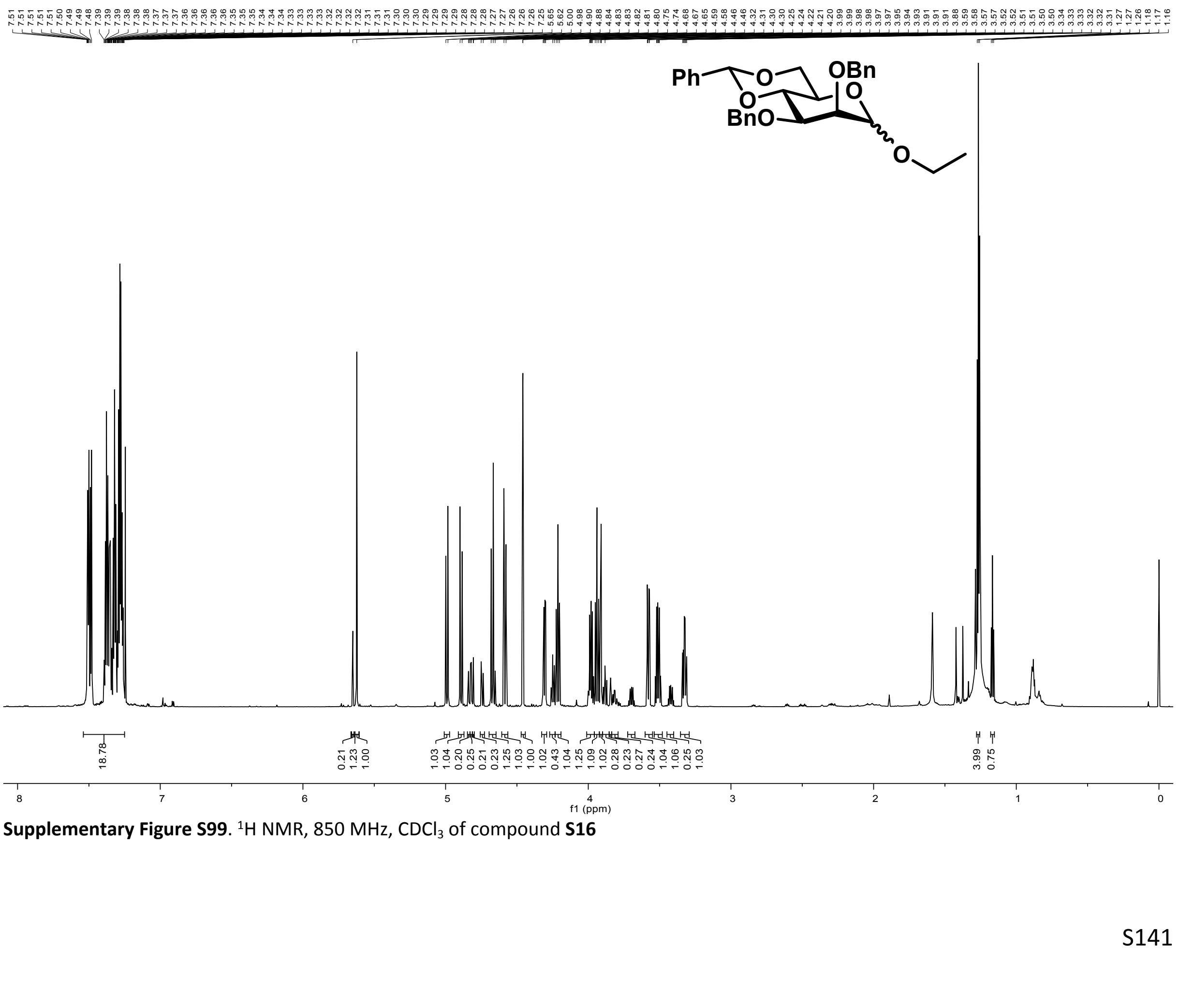

Supplementary Figure S99. ^1H NMR, 850 MHz, $CDCl_3$ of compound **S16**

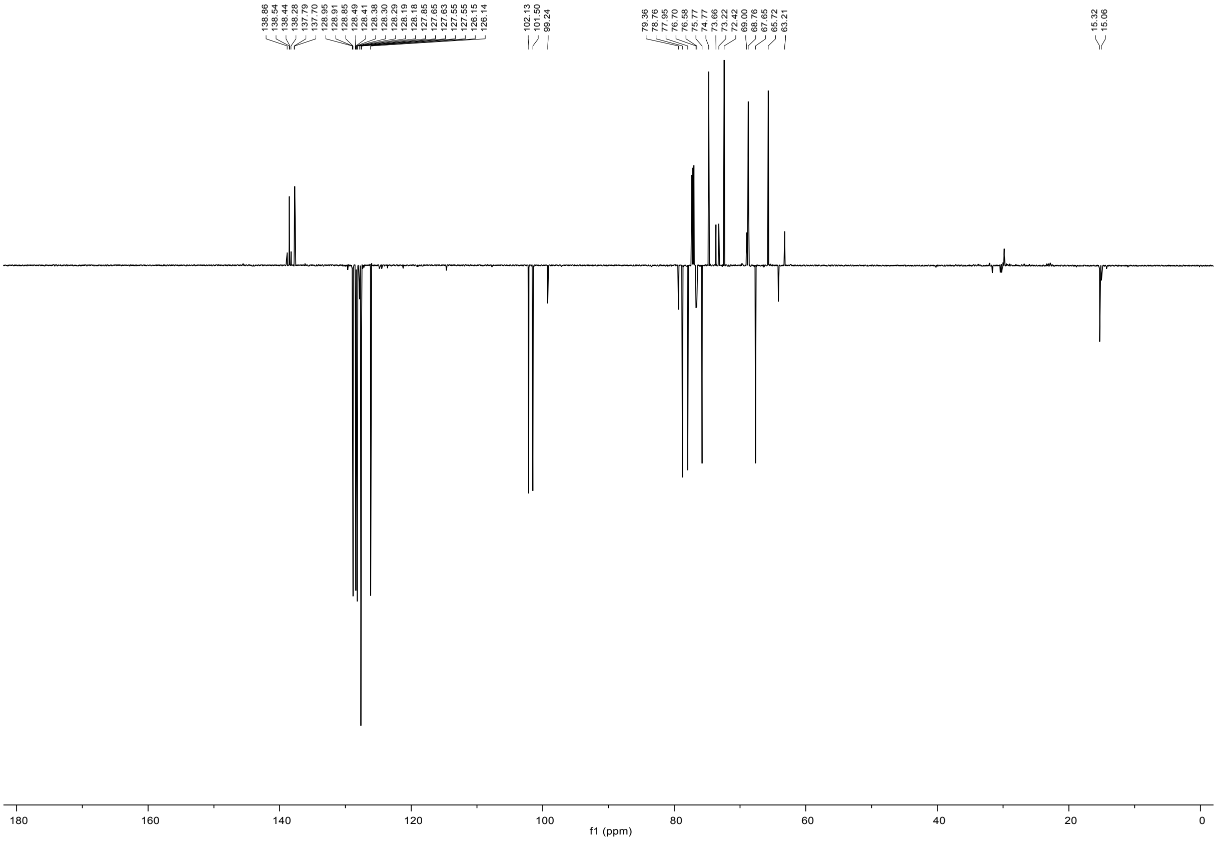

Supplementary Figure S100. $^{13}C\{^{1}H\}$ NMR, 214 MHz, $CDCl_3$ of compound **S16**

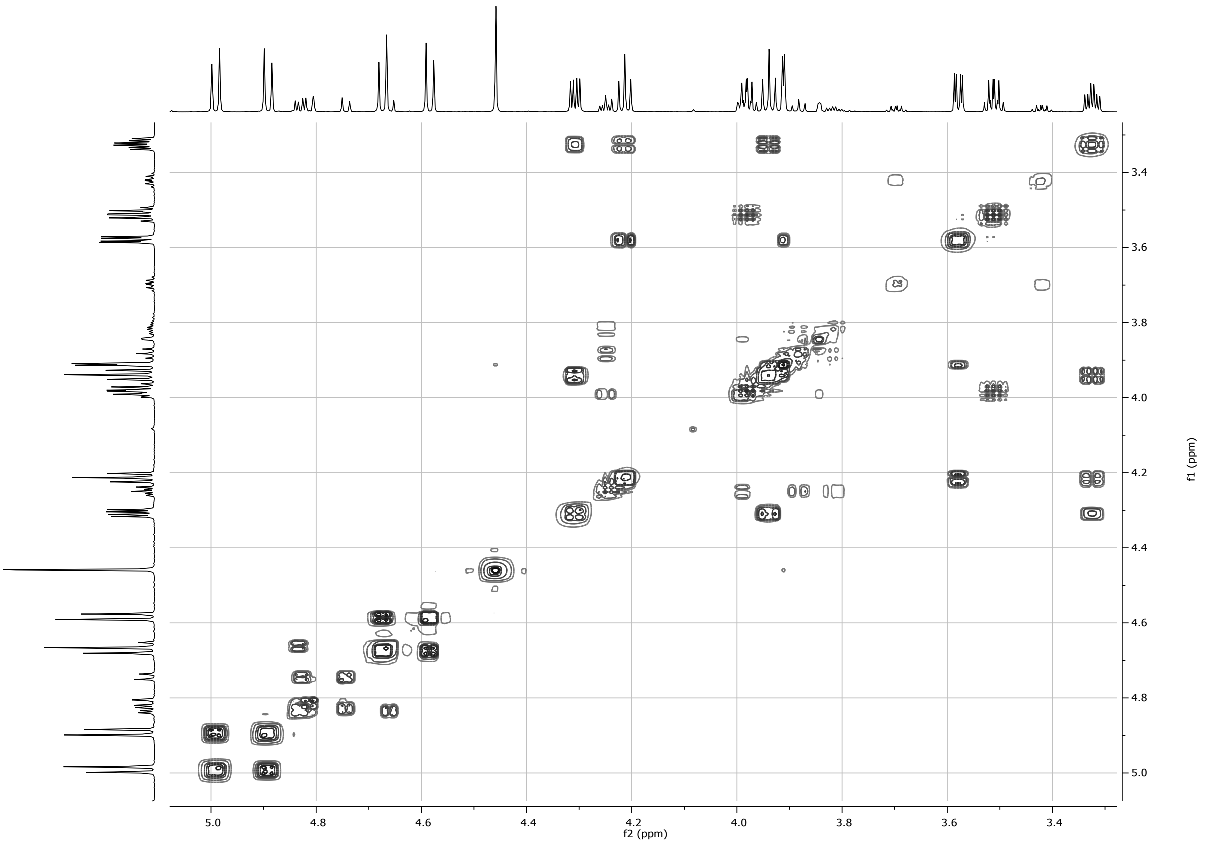

Supplementary Figure S101. HH-COSY NMR, $CDCl_3$ of compound **S16**

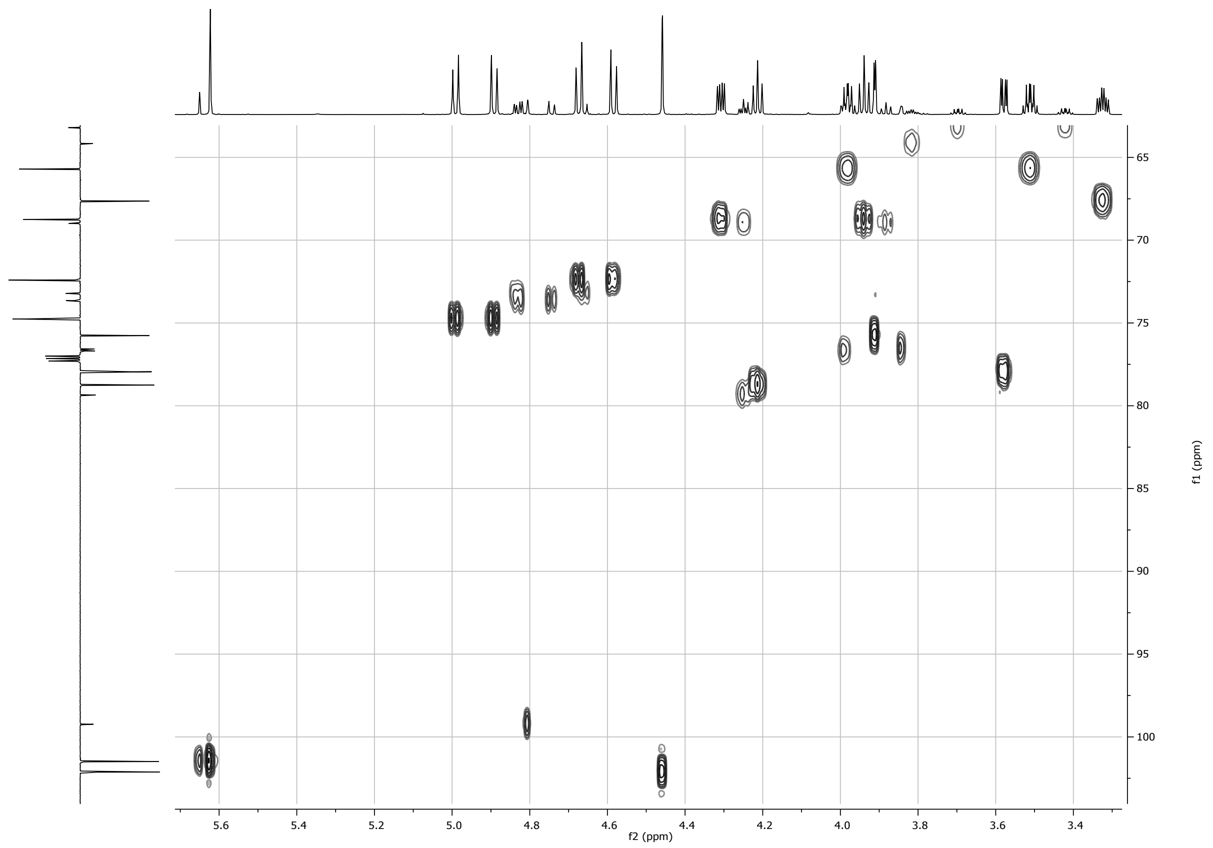

Supplementary Figure S102. HSQC{1H} NMR, $CDCl_3$ of compound **S16**

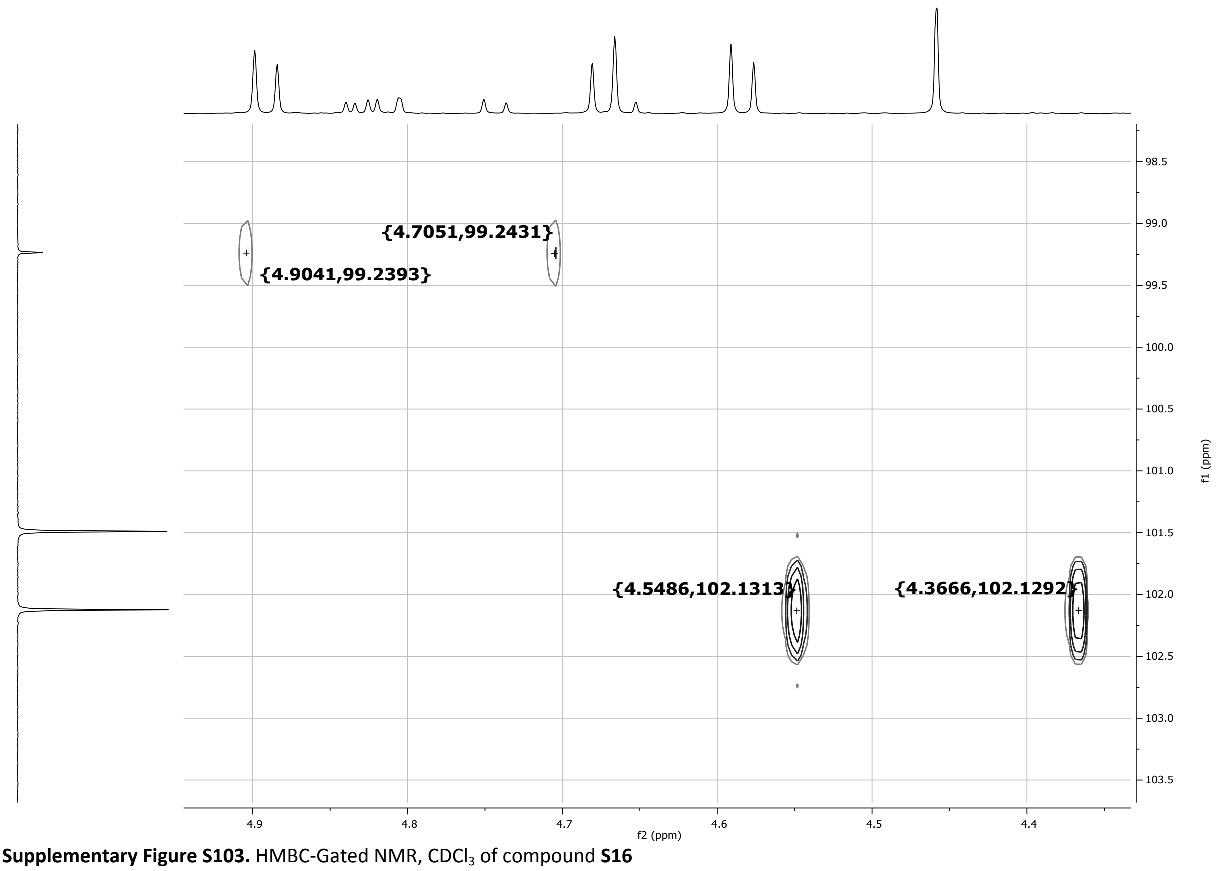

Supplementary Figure S103. HMBC-Gated NMR, $CDCl_3$ of compound **S16**

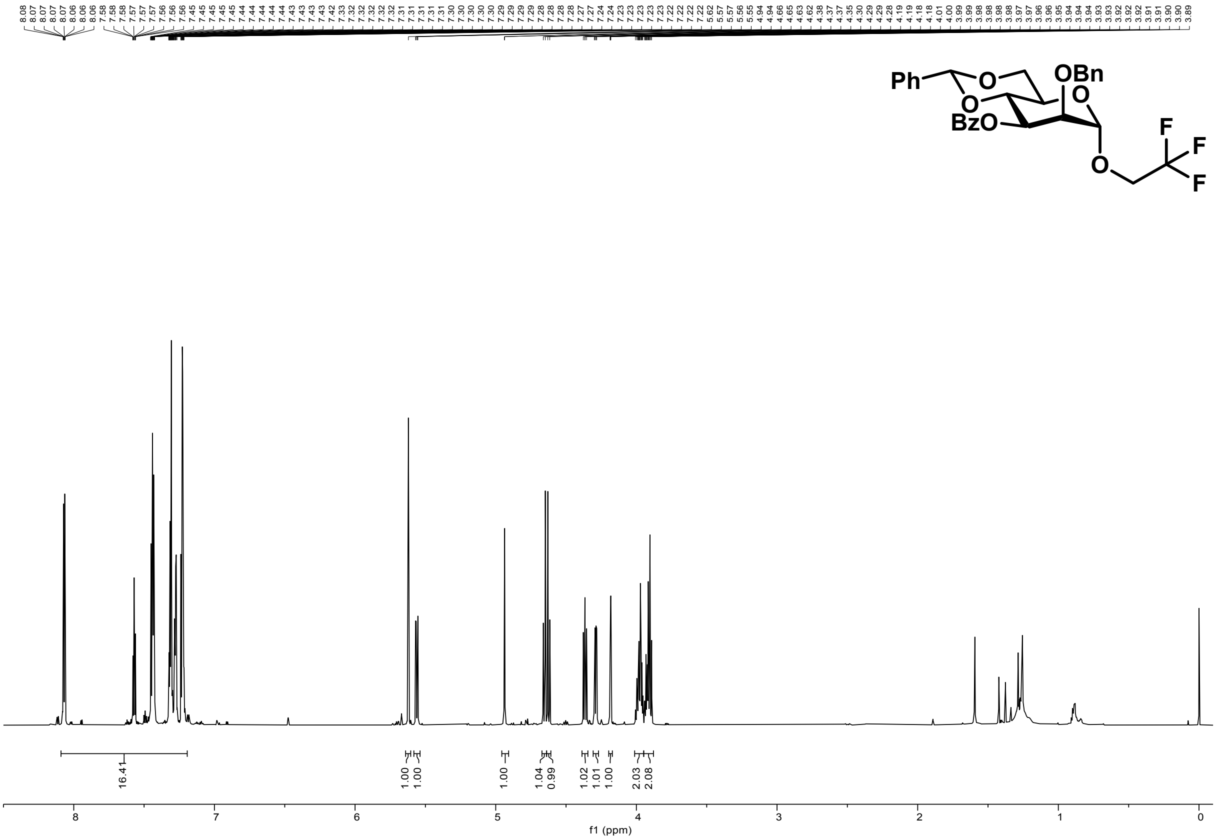

Supplementary Figure S104. ^{1}H NMR, 850 MHz, $CDCl_3$ of compound **S17**

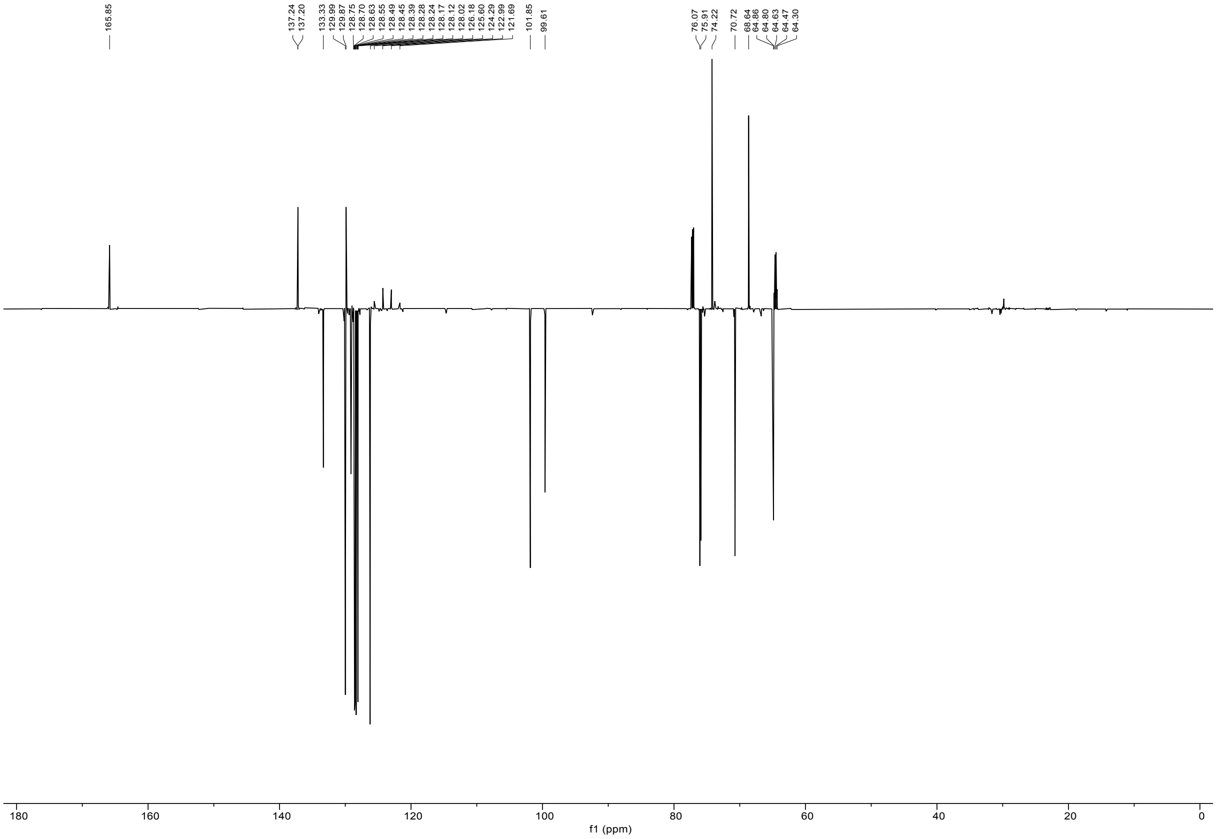

Supplementary Figure S105. $^{13}C\{^{1}H\}$ NMR, 214 MHz, $CDCl_3$ of compound **S17**

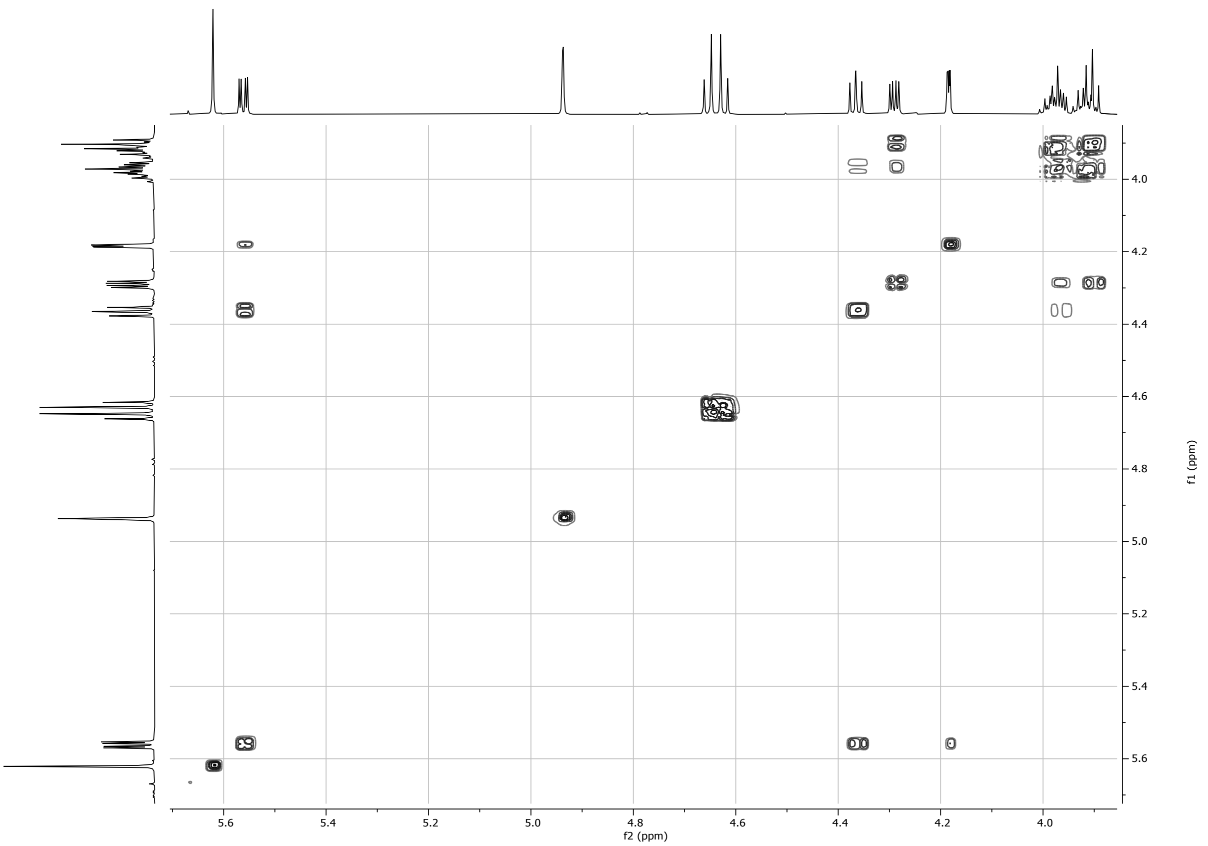

Supplementary Figure S106. HH-COSY NMR, $CDCl_3$ of compound **S17**

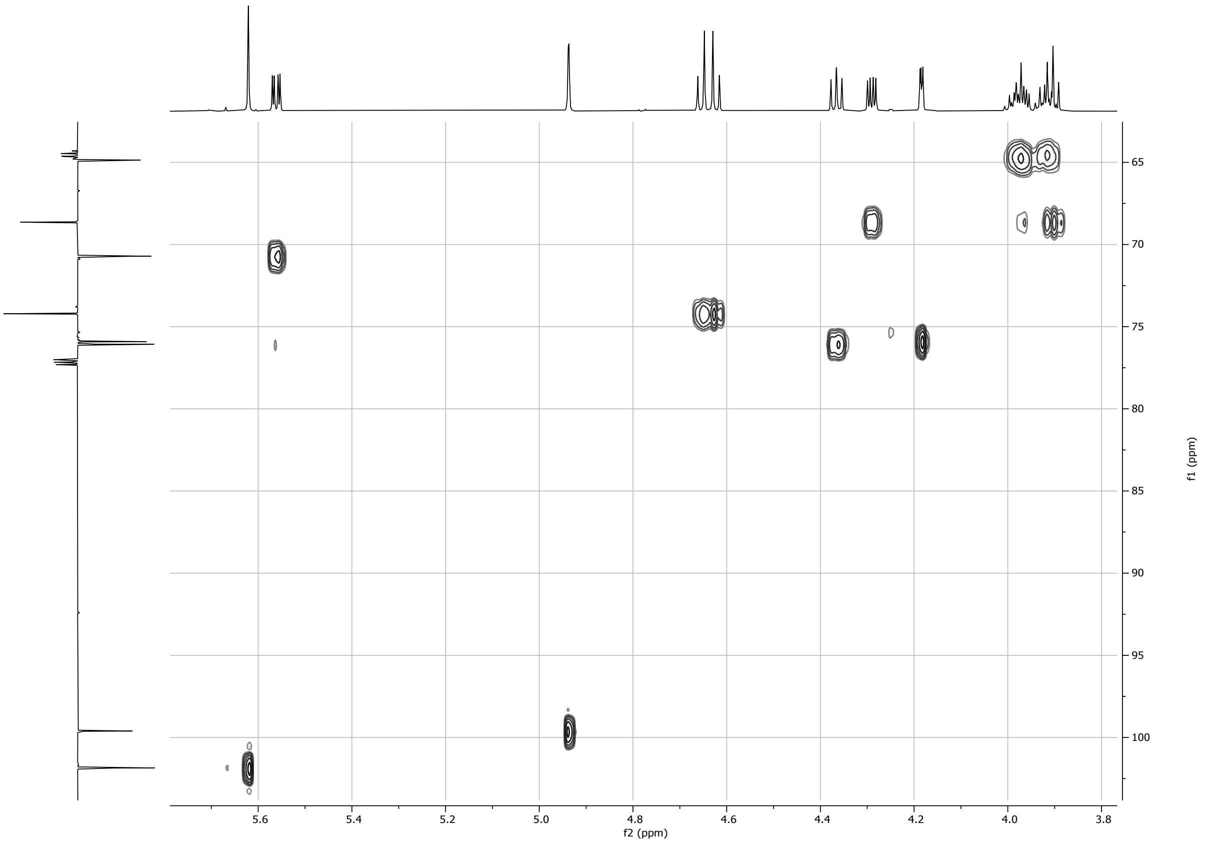

Supplementary Figure S107. HSQC{^{1}H} NMR, $CDCl_3$ of compound **S17**

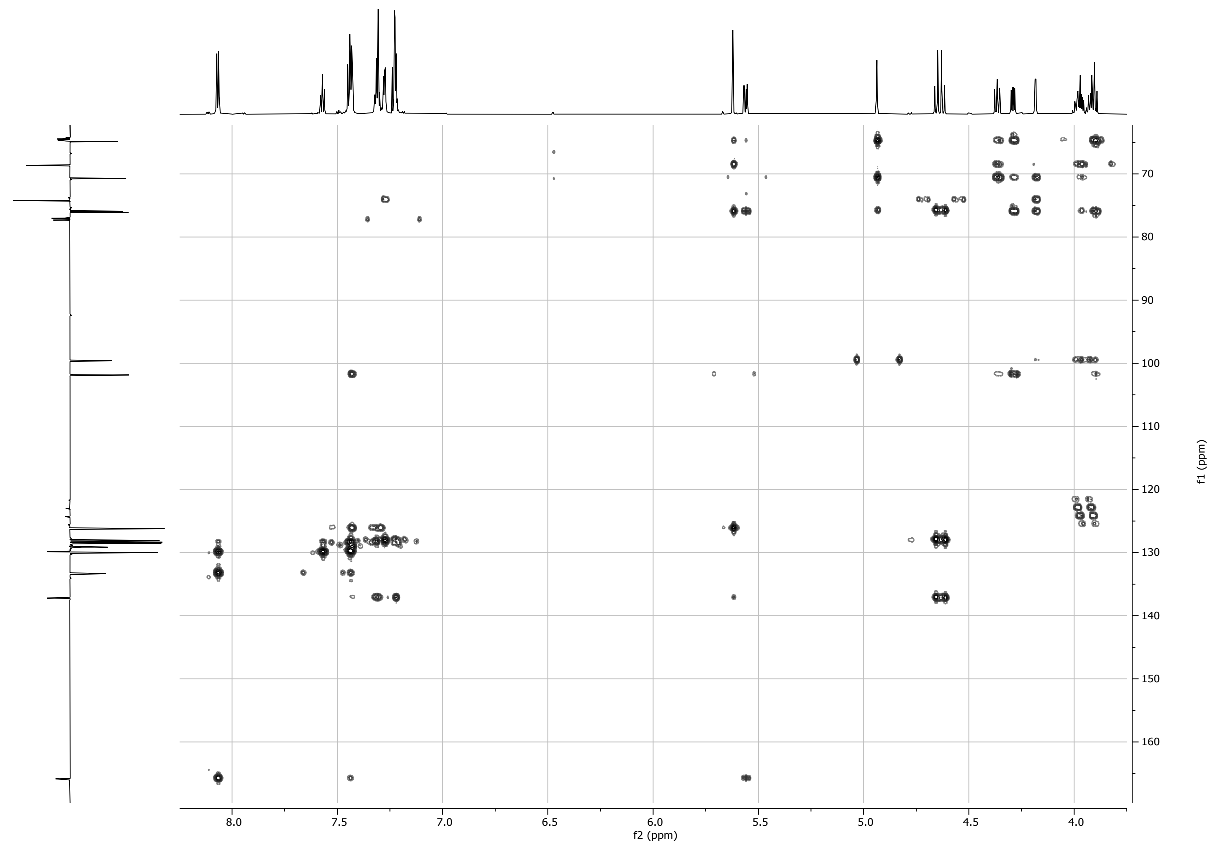

Supplementary Figure S108. HMBC NMR, $CDCl_3$ of compound **S17**

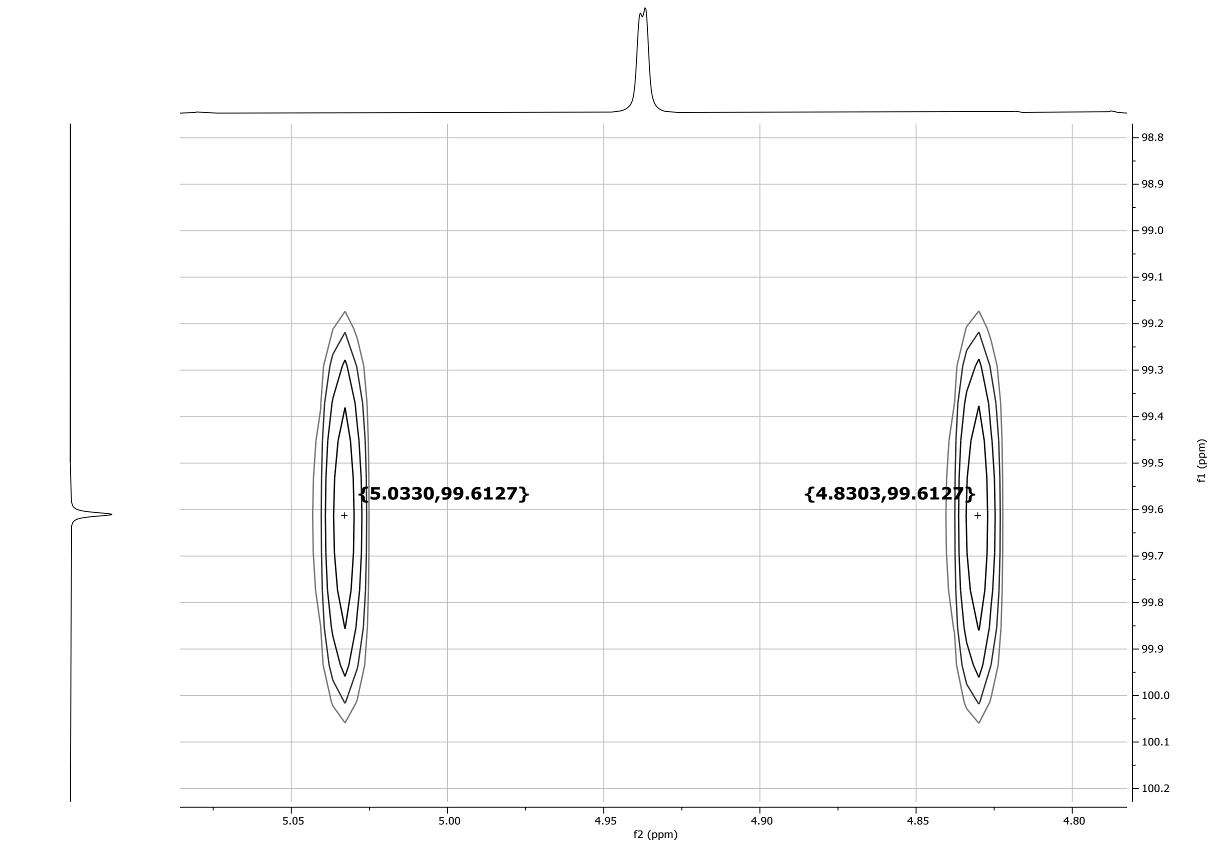

Supplementary Figure S109. HMBC-Gated NMR, $CDCl_3$ of compound **S17**

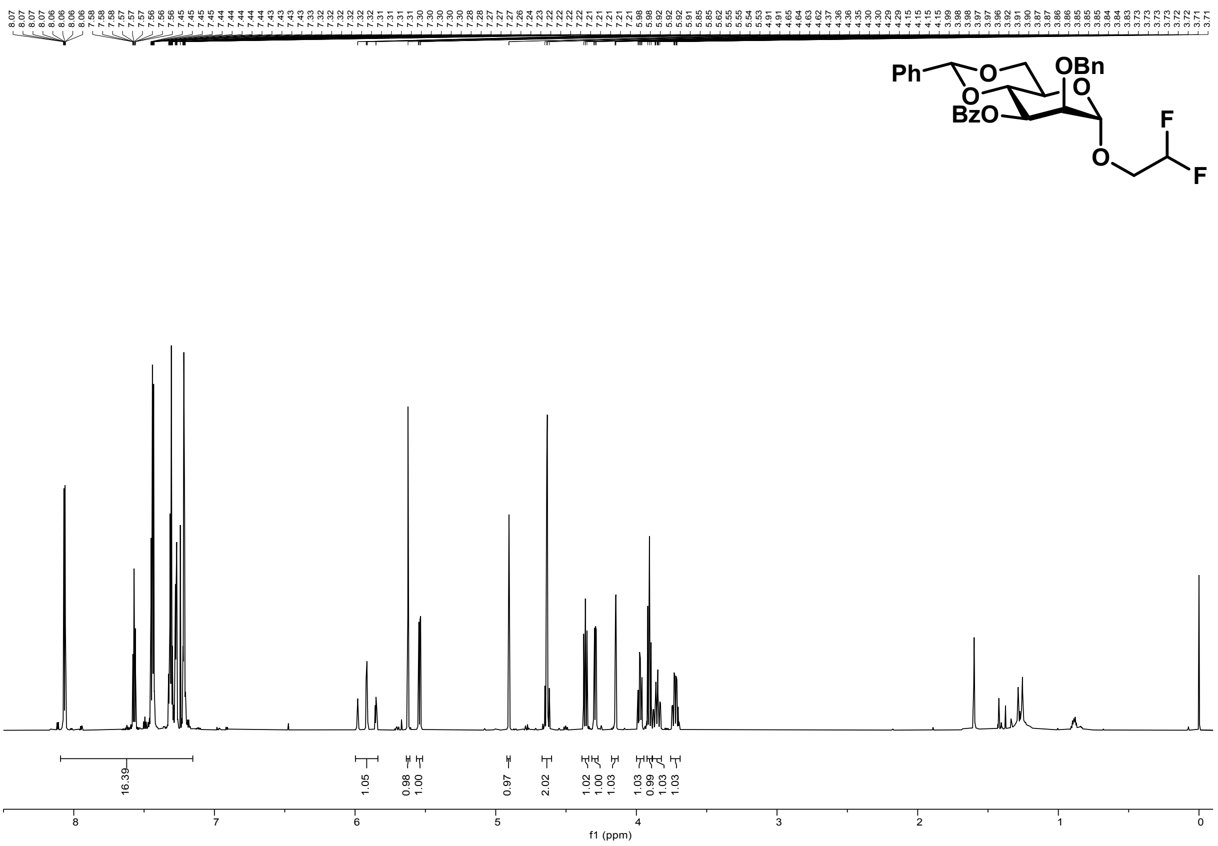

Supplementary Figure S110. ^{1}H NMR, 850 MHz, $CDCl_3$ of compound **S18**

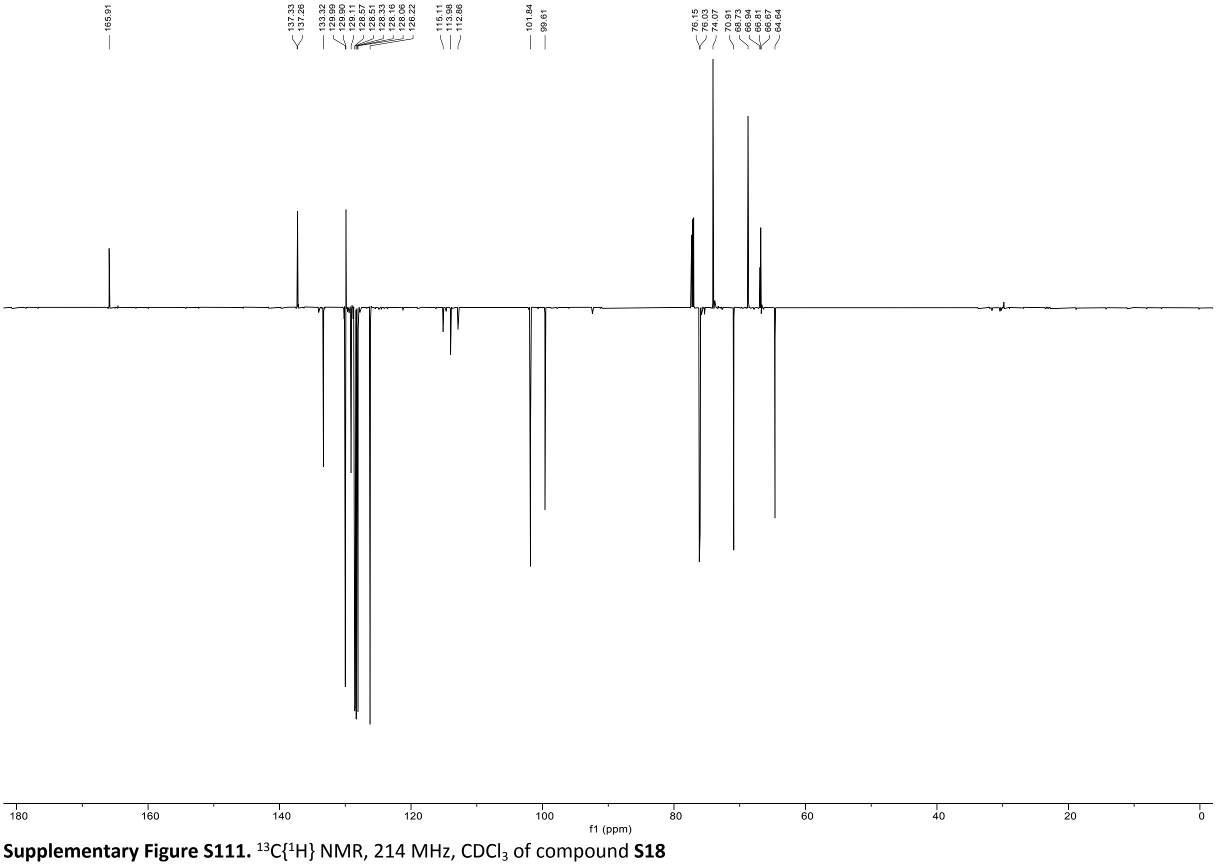

Supplementary Figure S111. $^{13}C\{^{1}H\}$ NMR, 214 MHz, $CDCl_3$ of compound **S18**

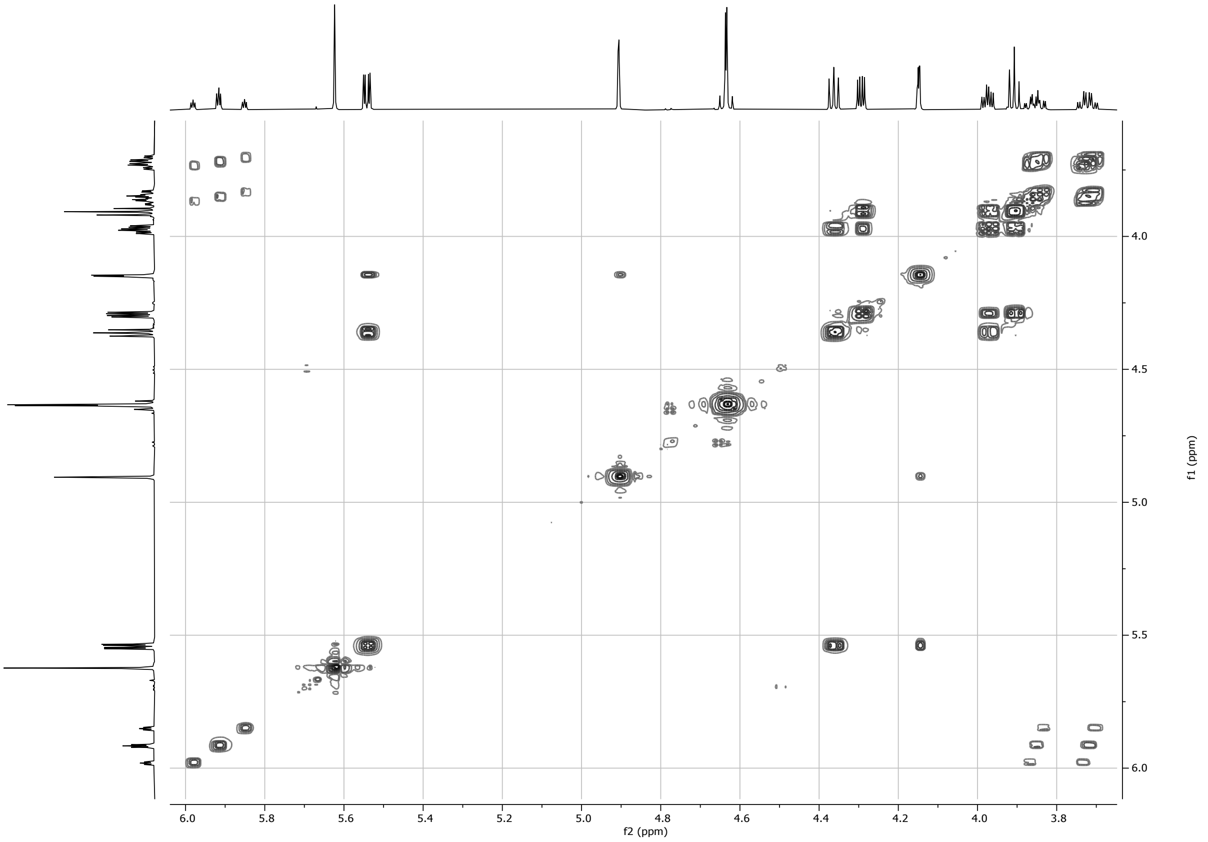

Supplementary Figure S112. HH-COSY NMR, $CDCl_3$ of compound **S18**

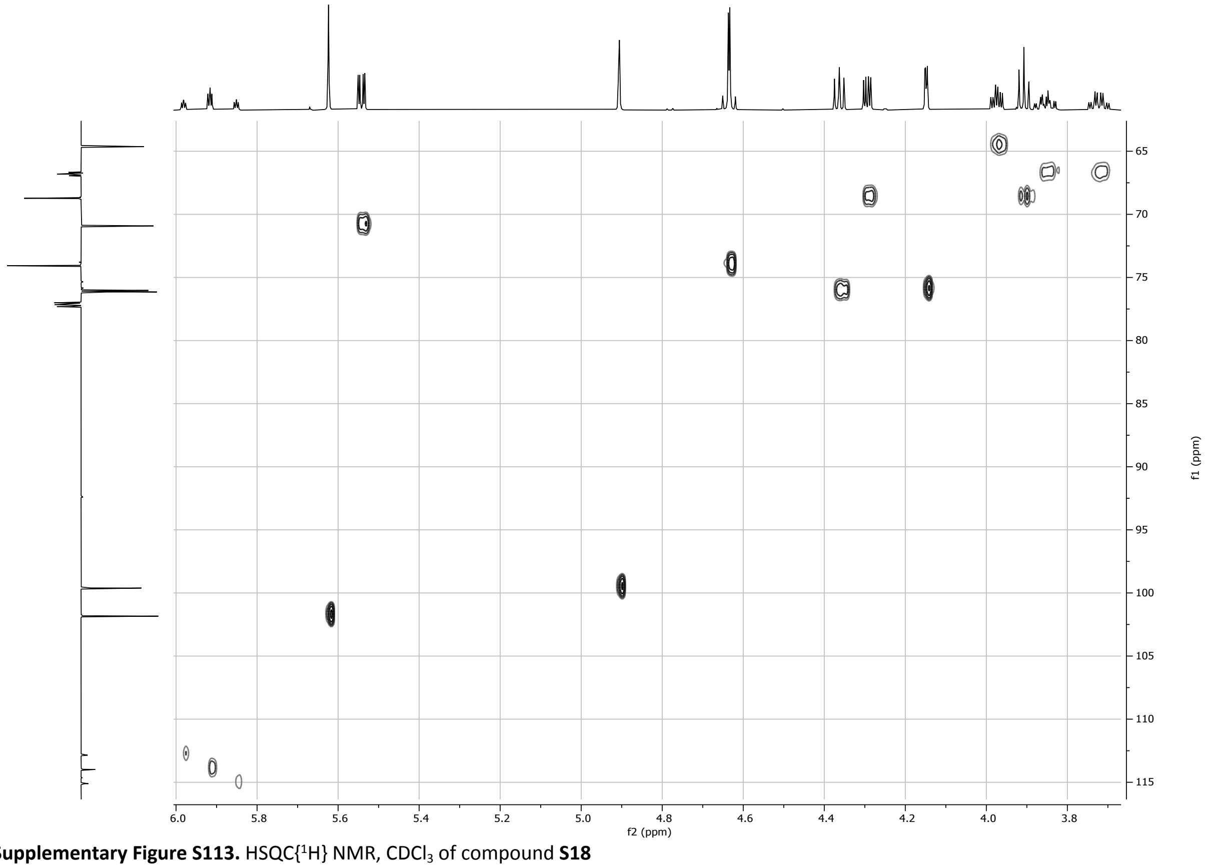

Supplementary Figure S113. HSQC{^{1}H} NMR, $CDCl_3$ of compound **S18**

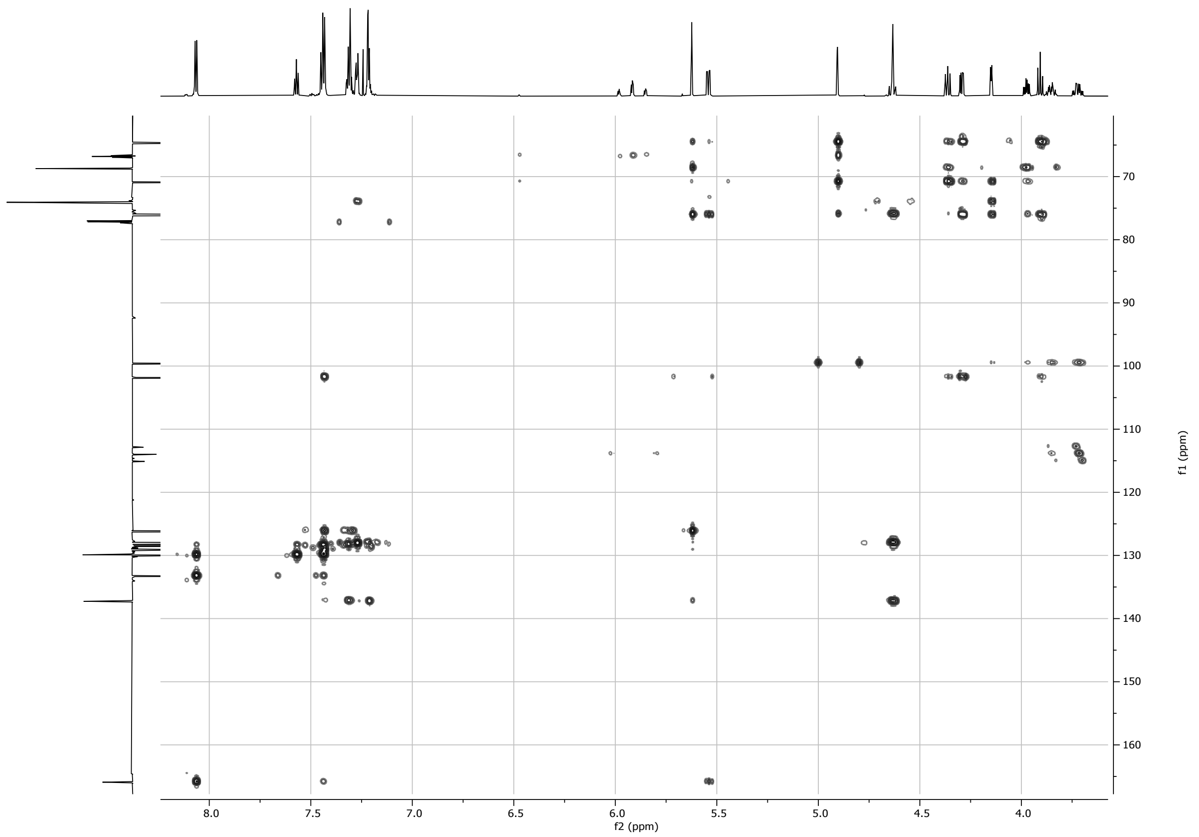

Supplementary Figure S114. HMBC NMR, $CDCl_3$ of compound **S18**

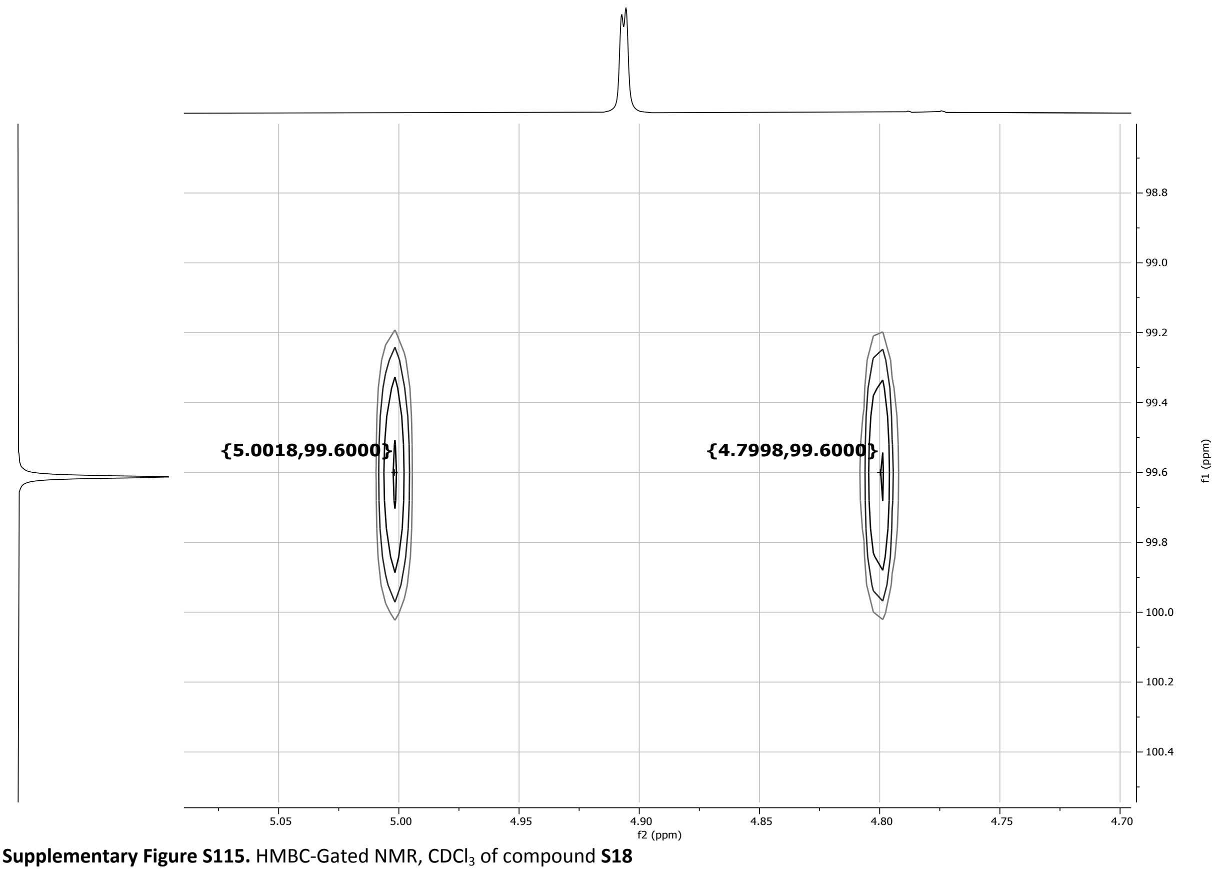

Supplementary Figure S115. HMBC-Gated NMR, $CDCl_3$ of compound **S18**

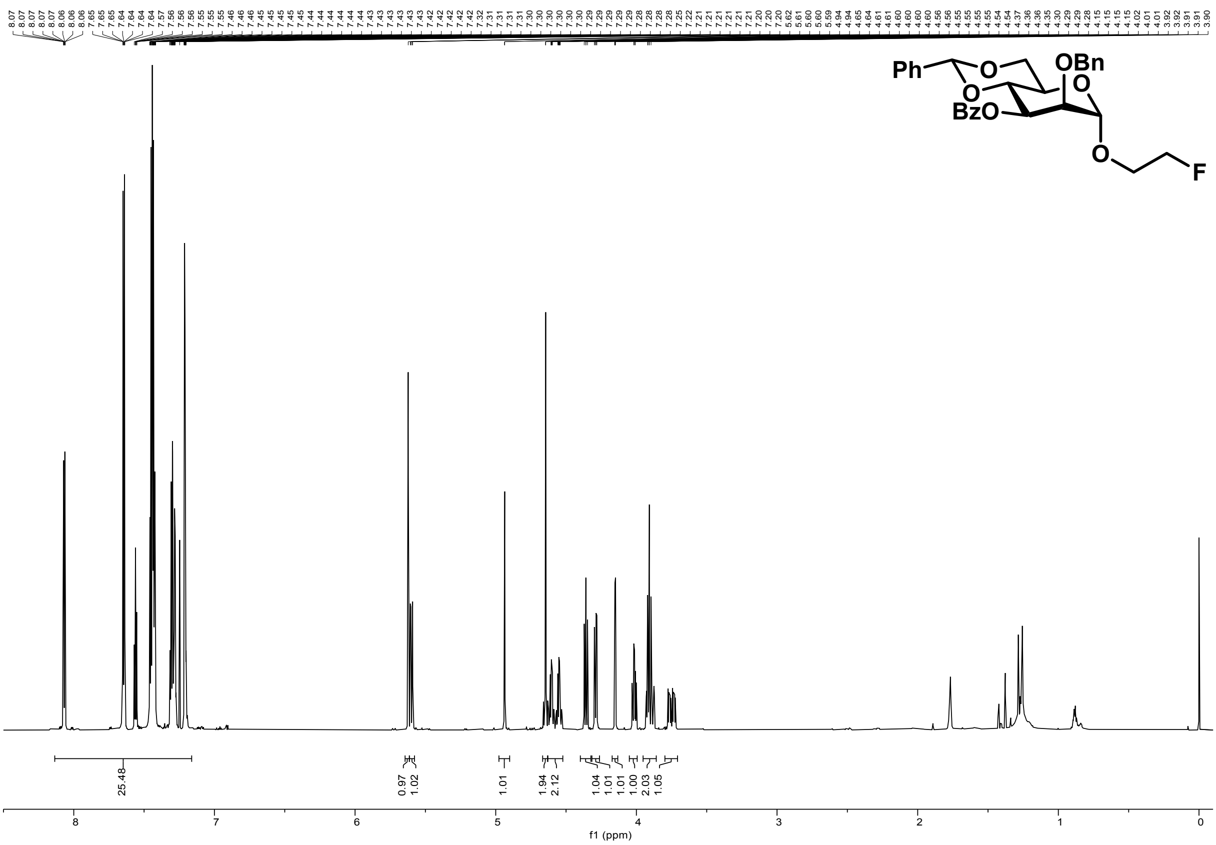

Supplementary Figure S116. ^{1}H NMR, 850 MHz, $CDCl_3$ of compound **S19**

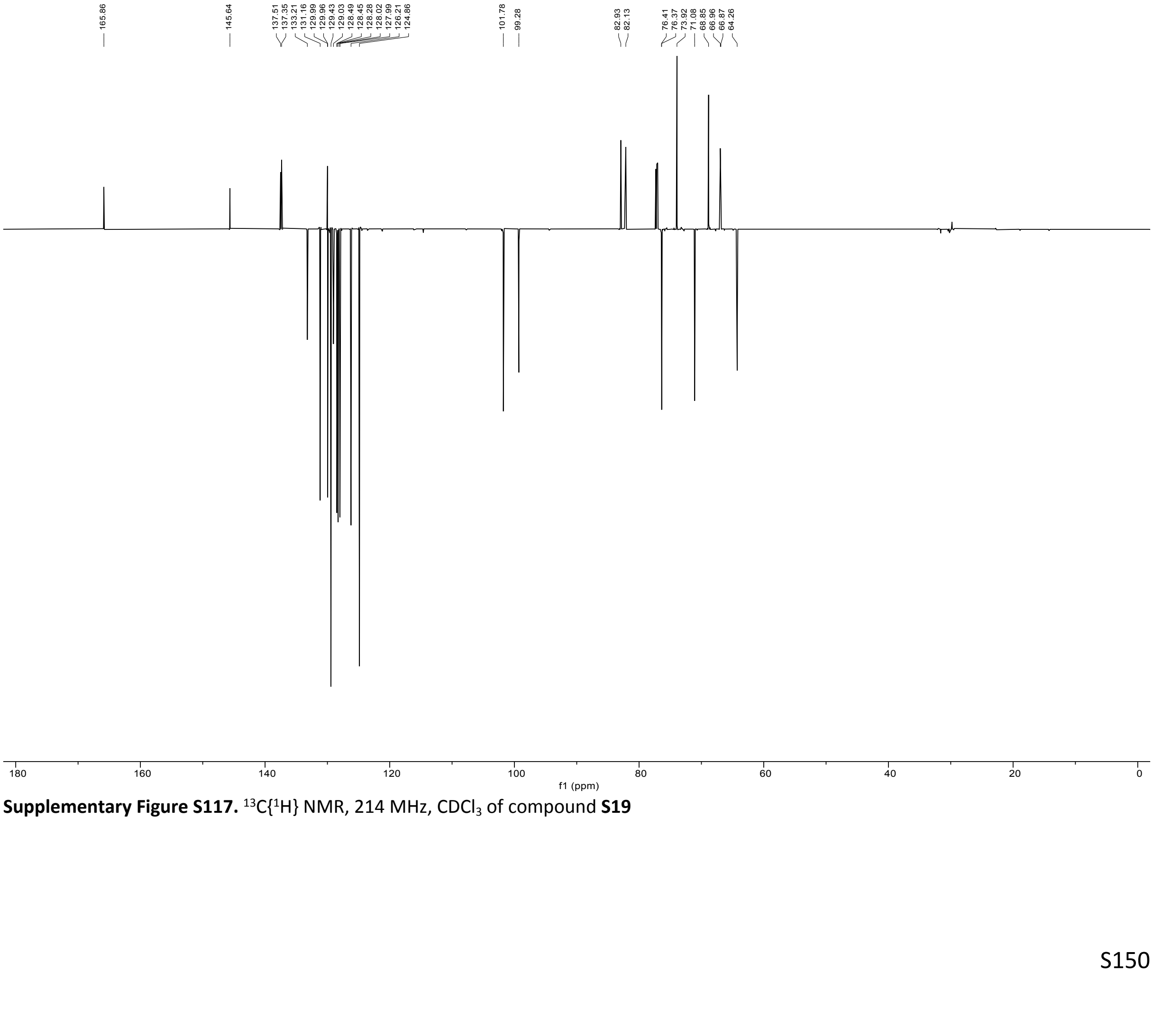

Supplementary Figure S117. $^{13}C\{^{1}H\}$ NMR, 214 MHz, $CDCl_3$ of compound **S19**

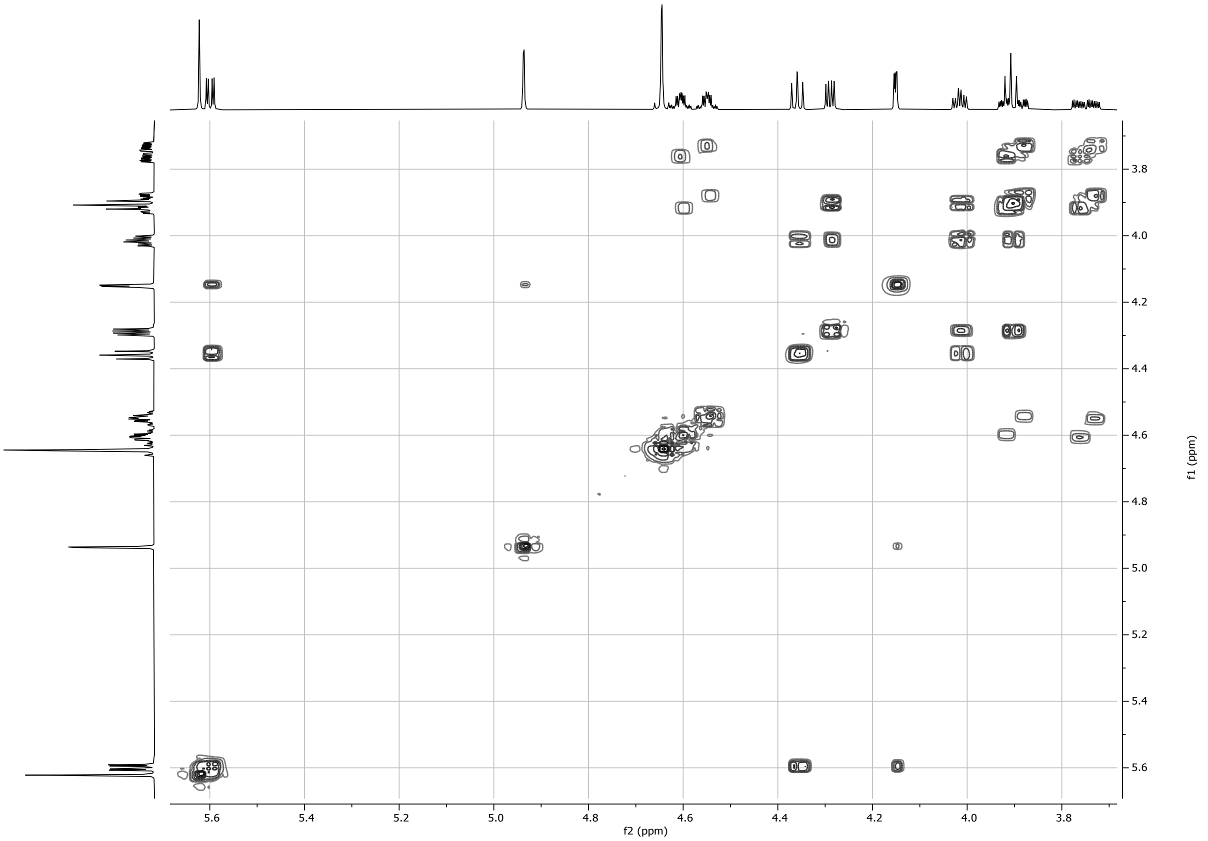

Supplementary Figure S118. HH-COSY NMR, $CDCl_3$ of compound **S19**

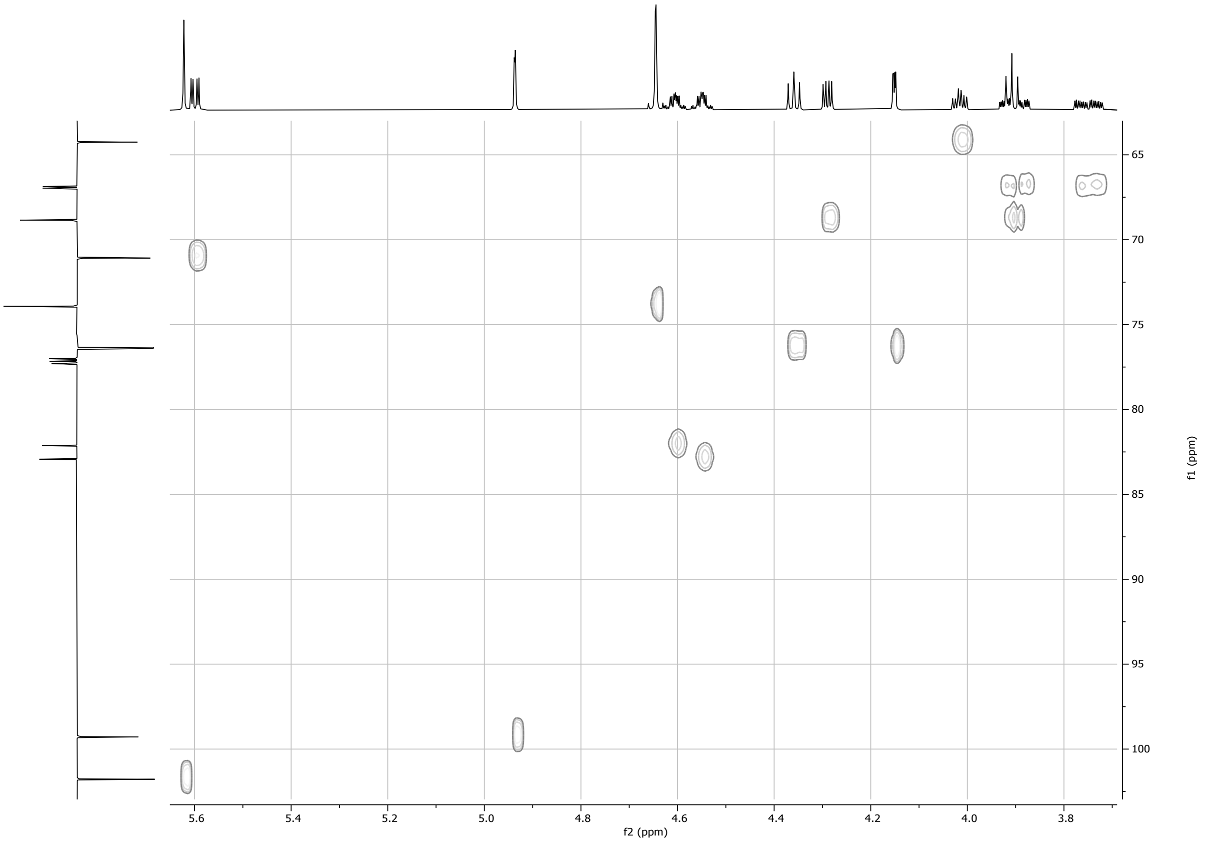

Supplementary Figure S119. HSQC{¹H} NMR, $CDCl_3$ of compound **S19**

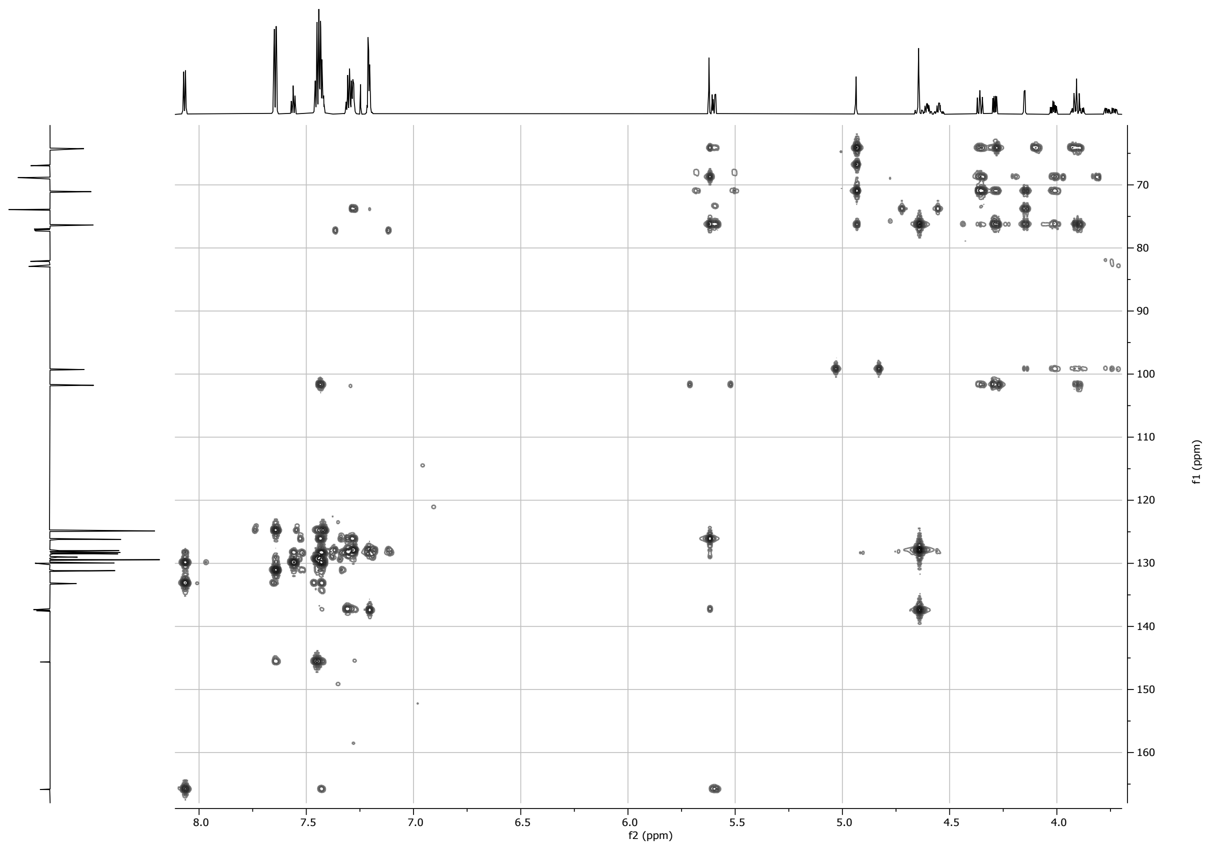

Supplementary Figure S120. HMBC NMR, $CDCl_3$ of compound **S19**

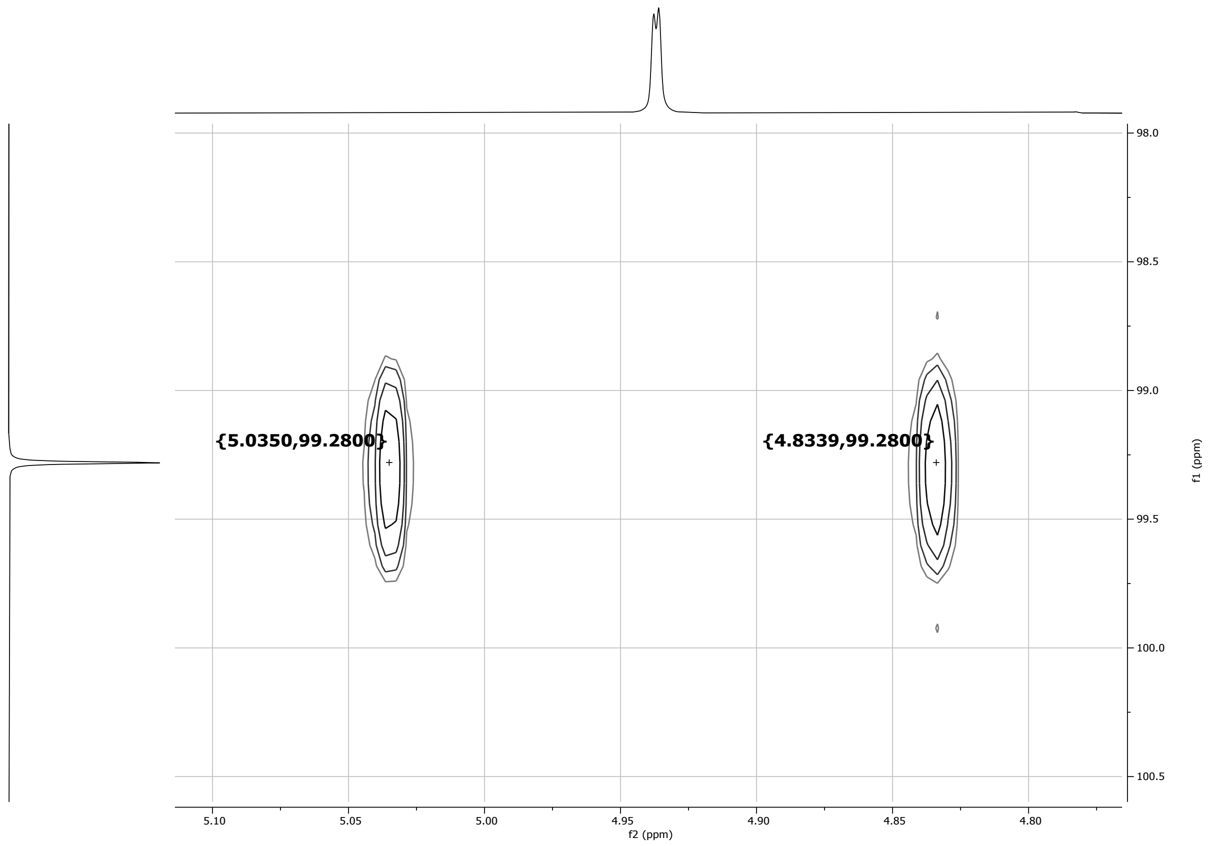

Supplementary Figure S121. HMBC-Gated NMR, $CDCl_3$ of compound **S19**

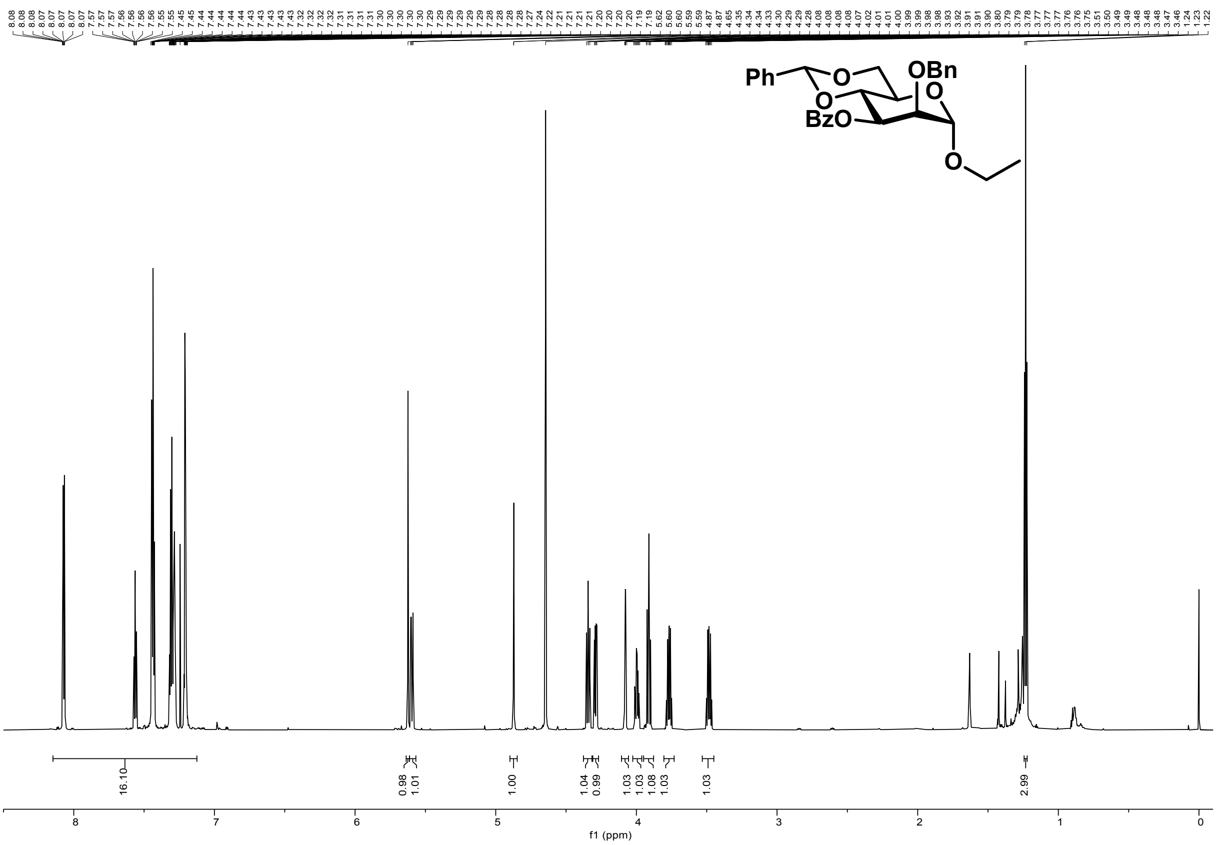

Supplementary Figure S122. ^{1}H NMR, 850 MHz, $CDCl_3$ of compound **S20**

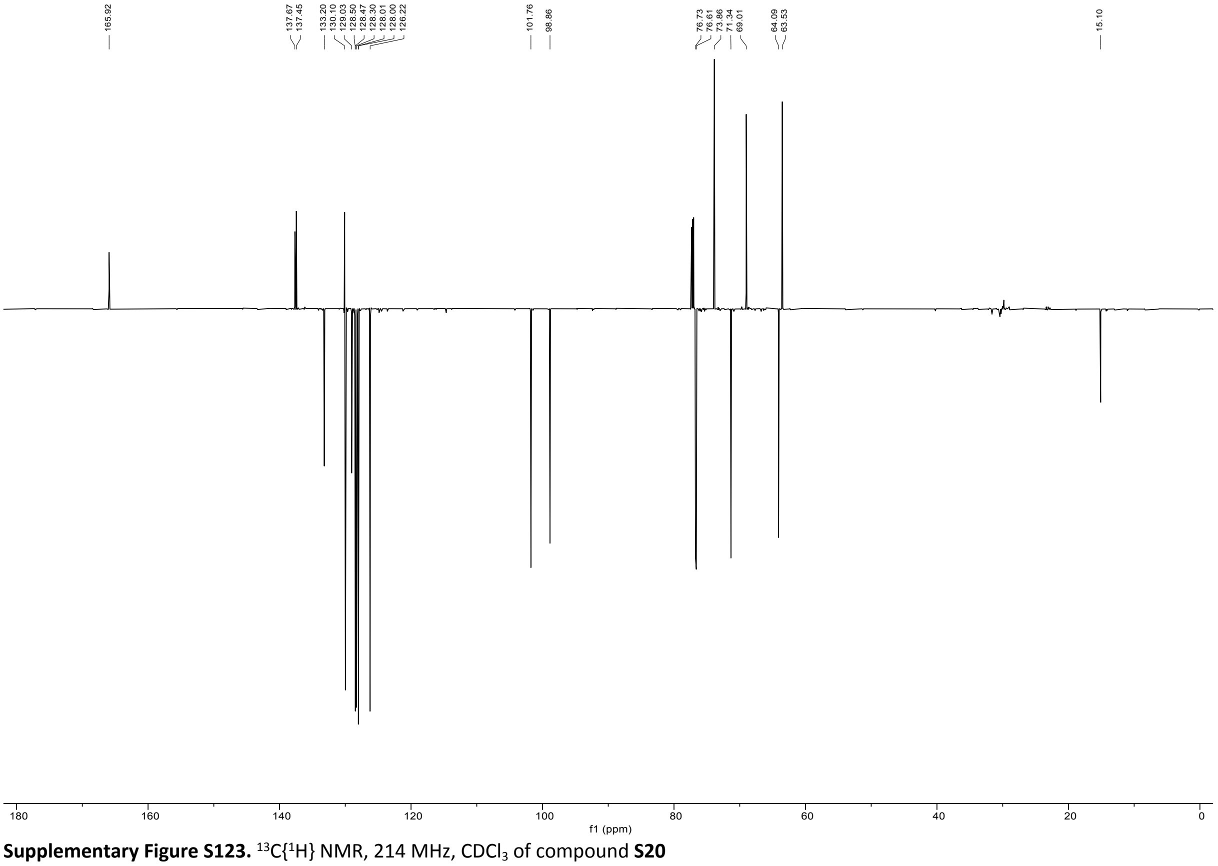

Supplementary Figure S123. $^{13}C\{^{1}H\}$ NMR, 214 MHz, $CDCl_3$ of compound **S20**

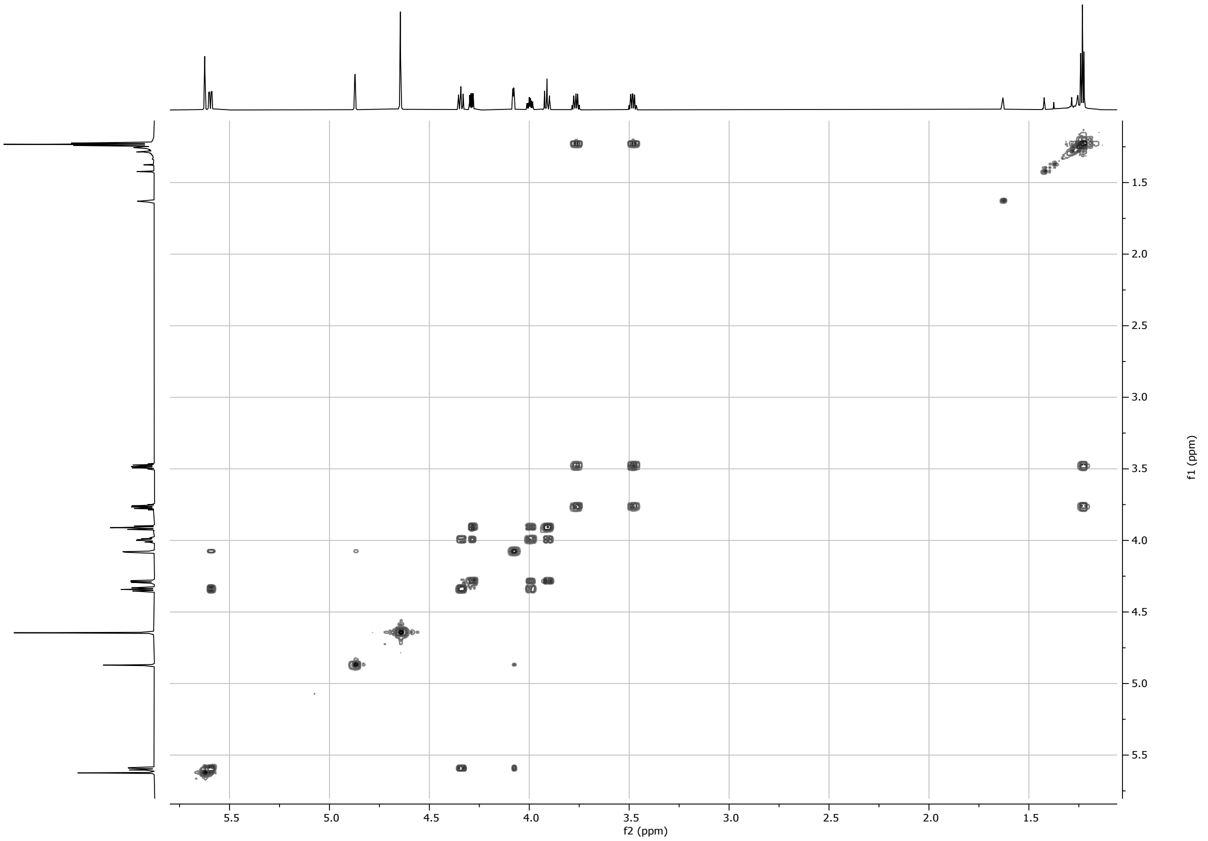

Supplementary Figure S124. HH-COSY NMR, $CDCl_3$ of compound **S20**

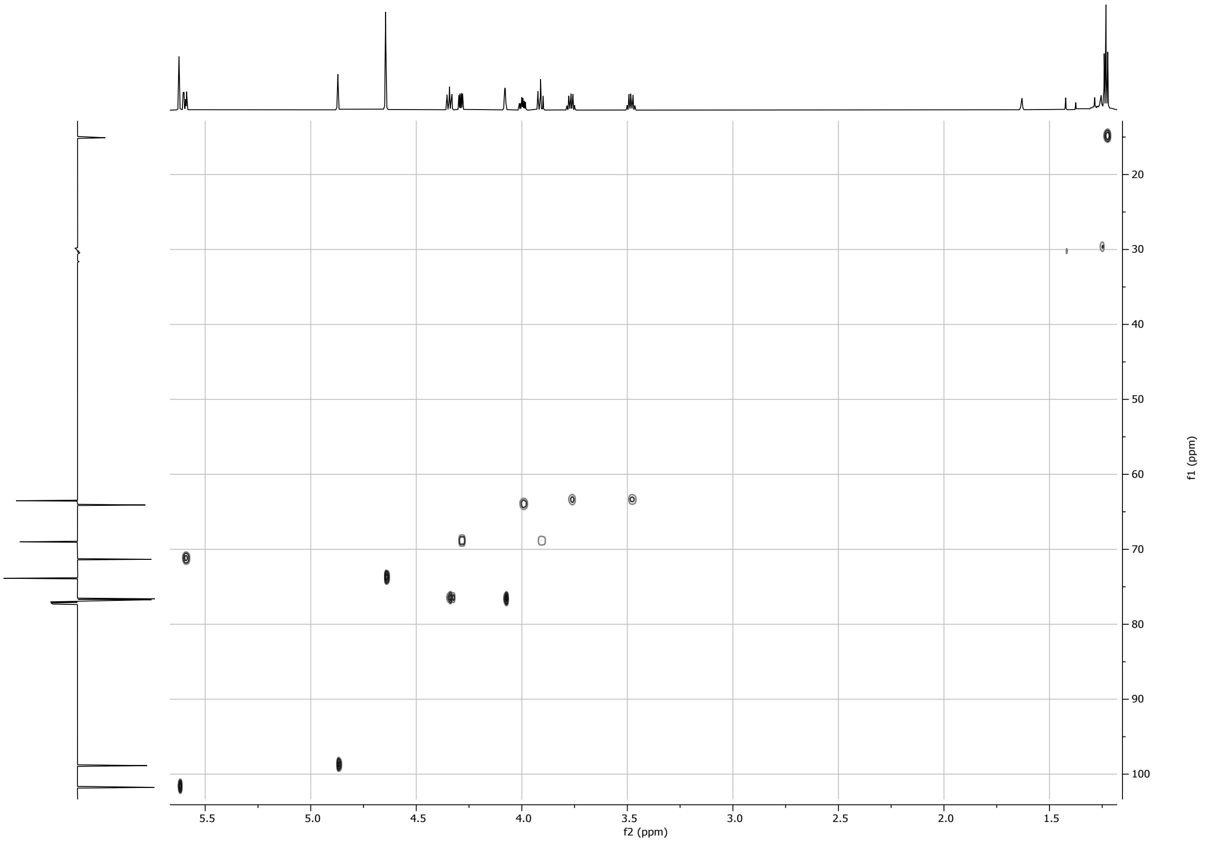

Supplementary Figure S125. HSQC{^{1}H} NMR, $CDCl_3$ of compound **S20**

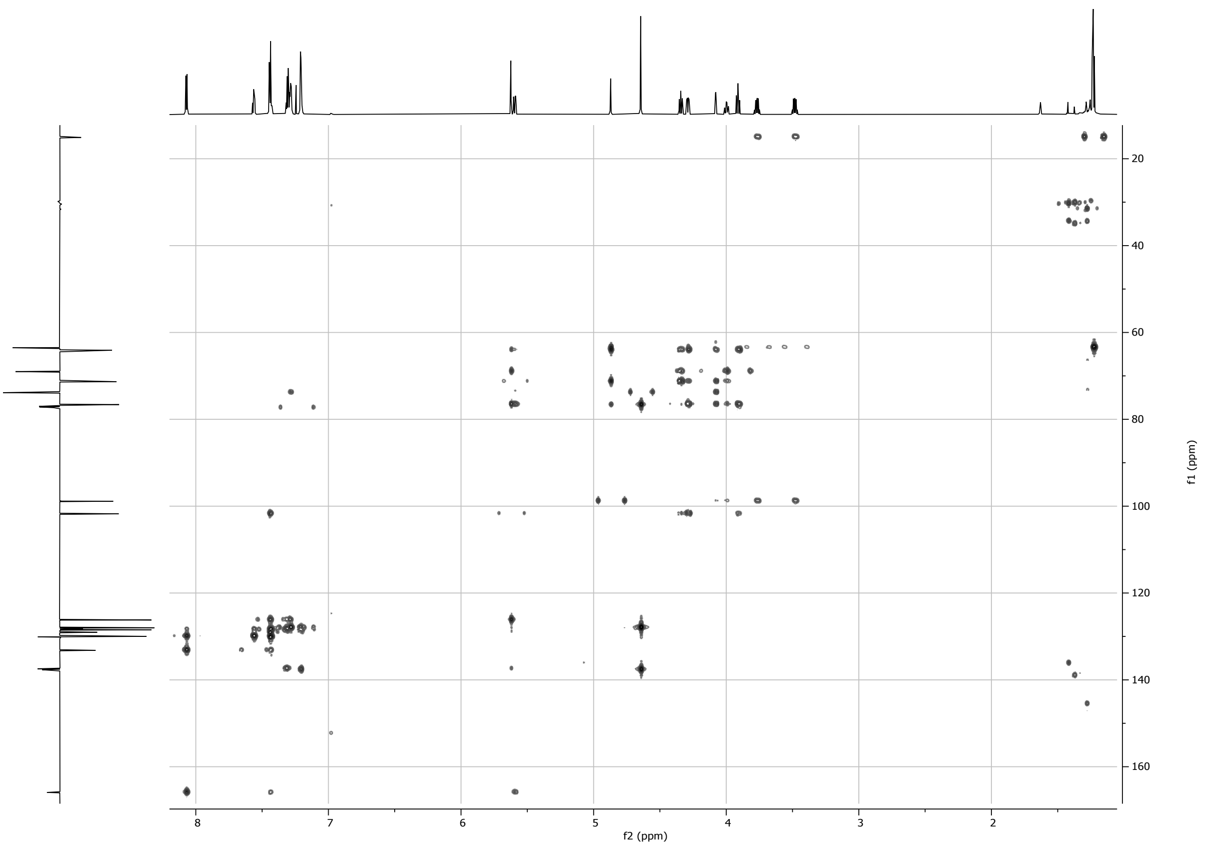

Supplementary Figure S126. HMBC NMR, $CDCl_3$ of compound **S20**

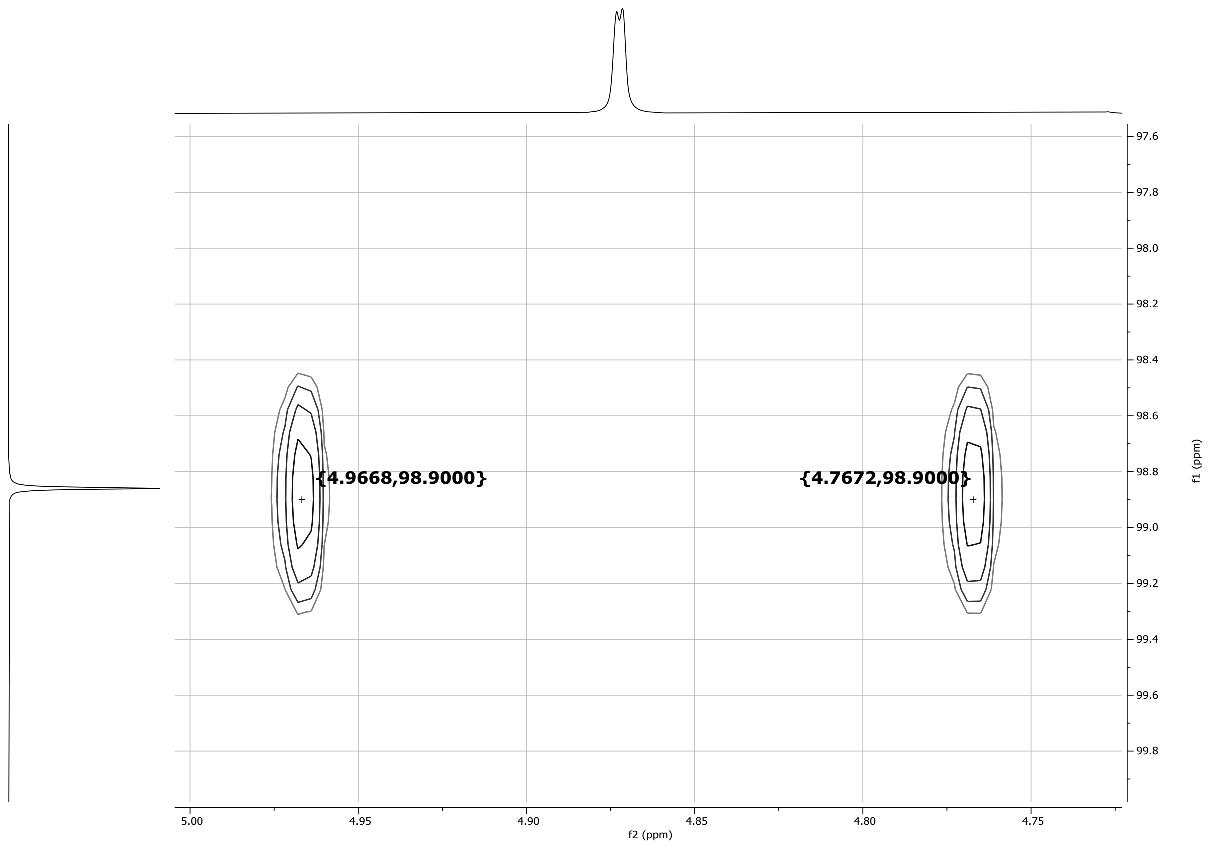

Supplementary Figure S127. HMBC-Gated NMR, $CDCl_3$ of compound **S20**

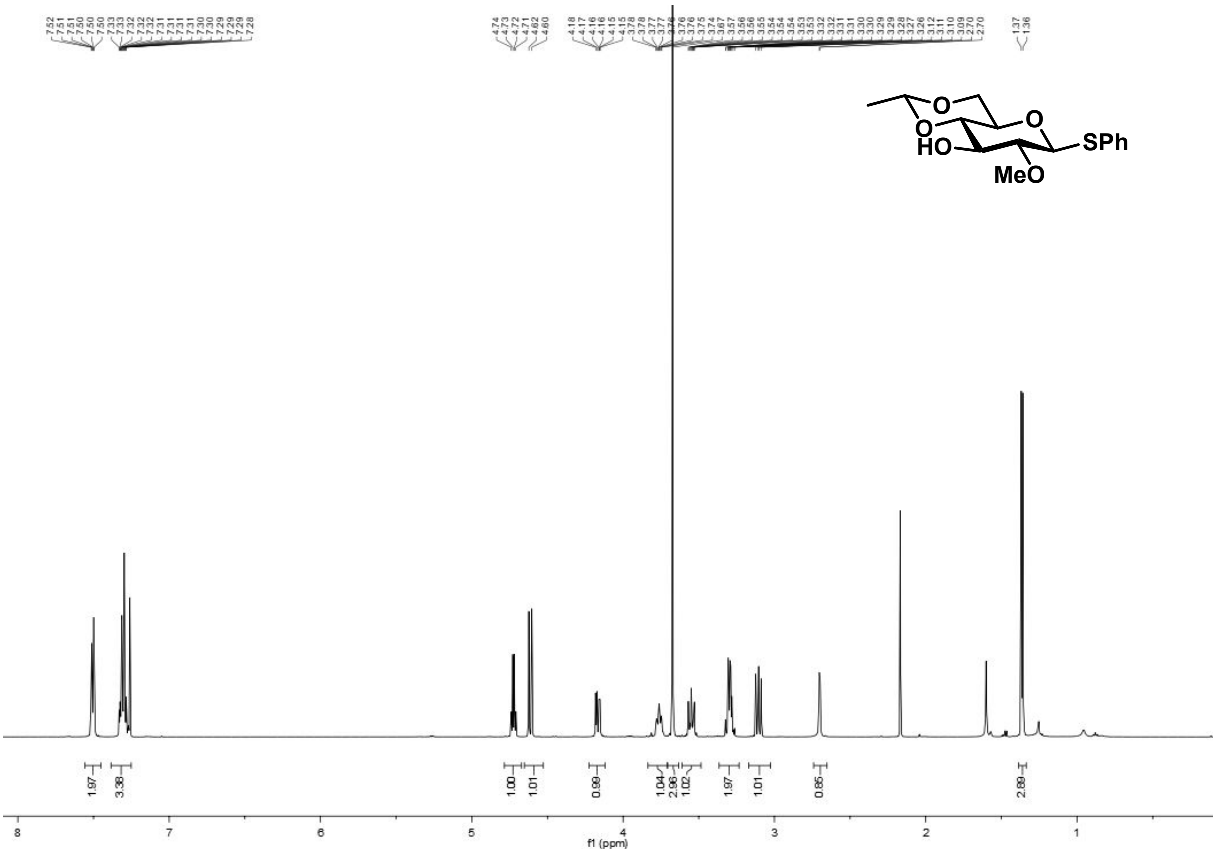

Supplementary Figure S128. ^{1}H NMR, 500 MHz, $CDCl_3$ of compound **S21**

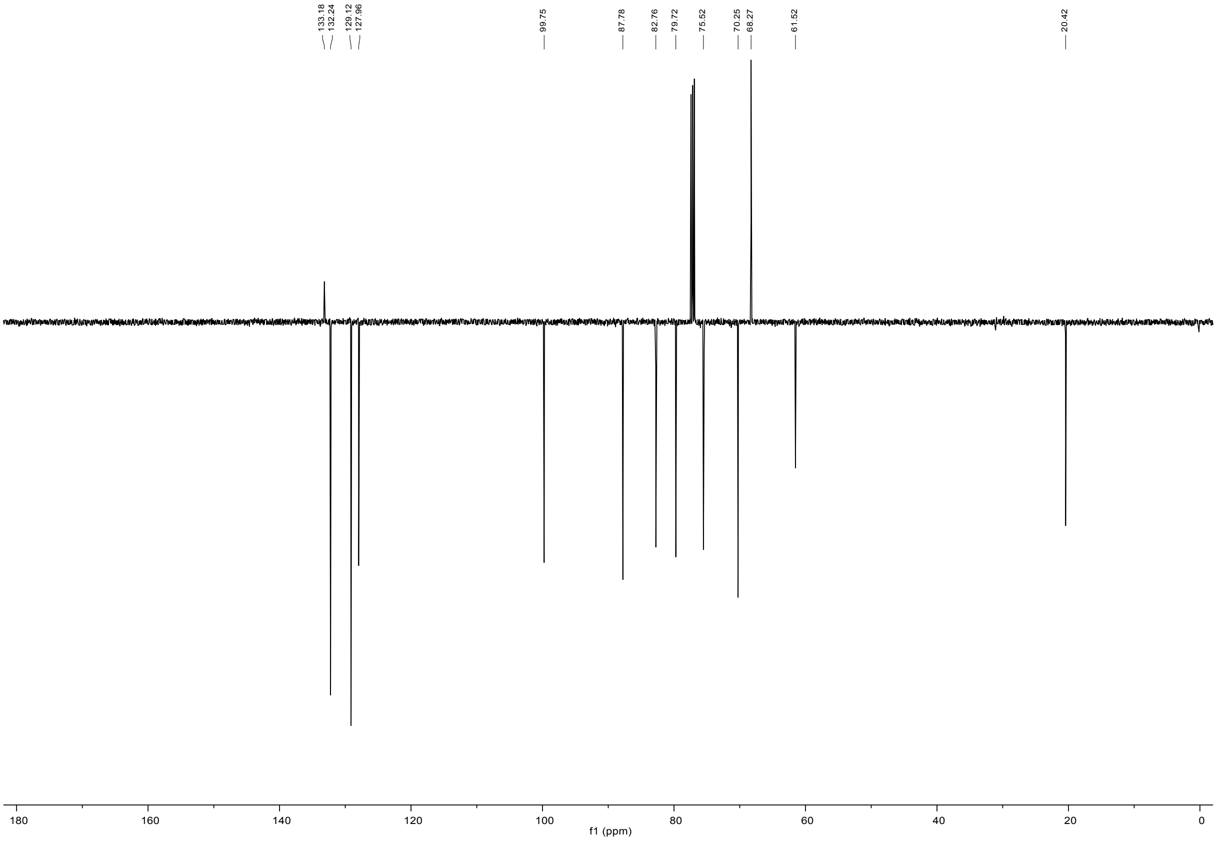

Supplementary Figure S129. $^{13}C\{^{1}H\}$ NMR, 126 MHz, $CDCl_3$ of compound **S21**

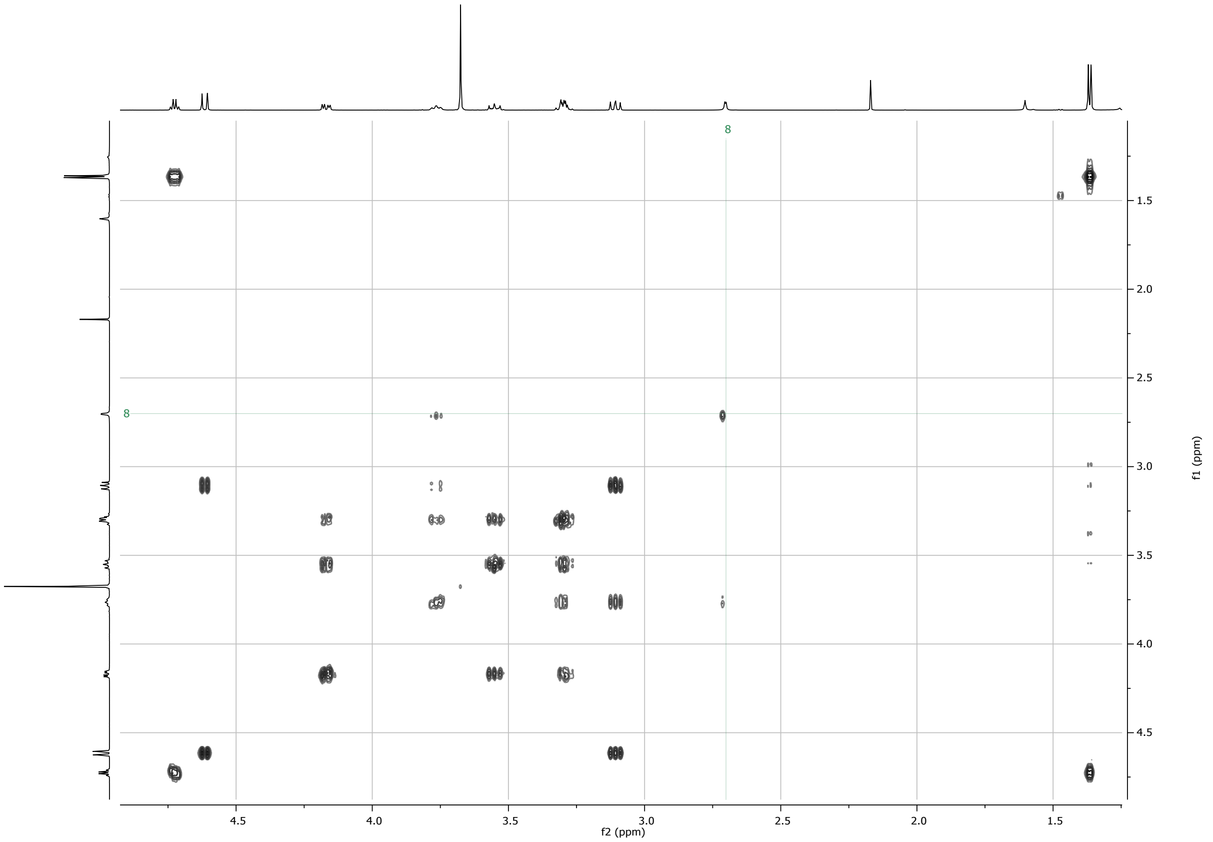

Supplementary Figure S130. HH-COSY NMR, $CDCl_3$ of compound **S21**

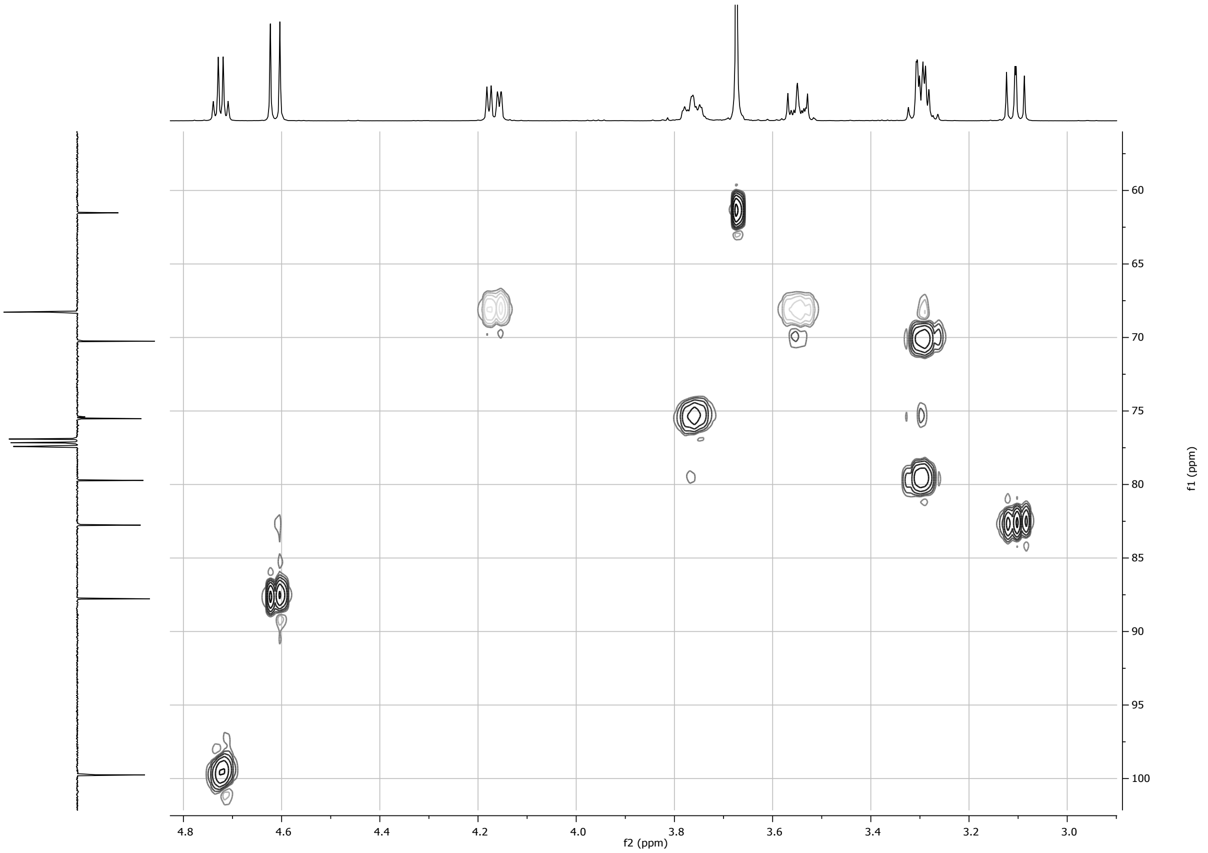

Supplementary Figure S131. HSQC{^{1}H} NMR, $CDCl_3$ of compound **S21**

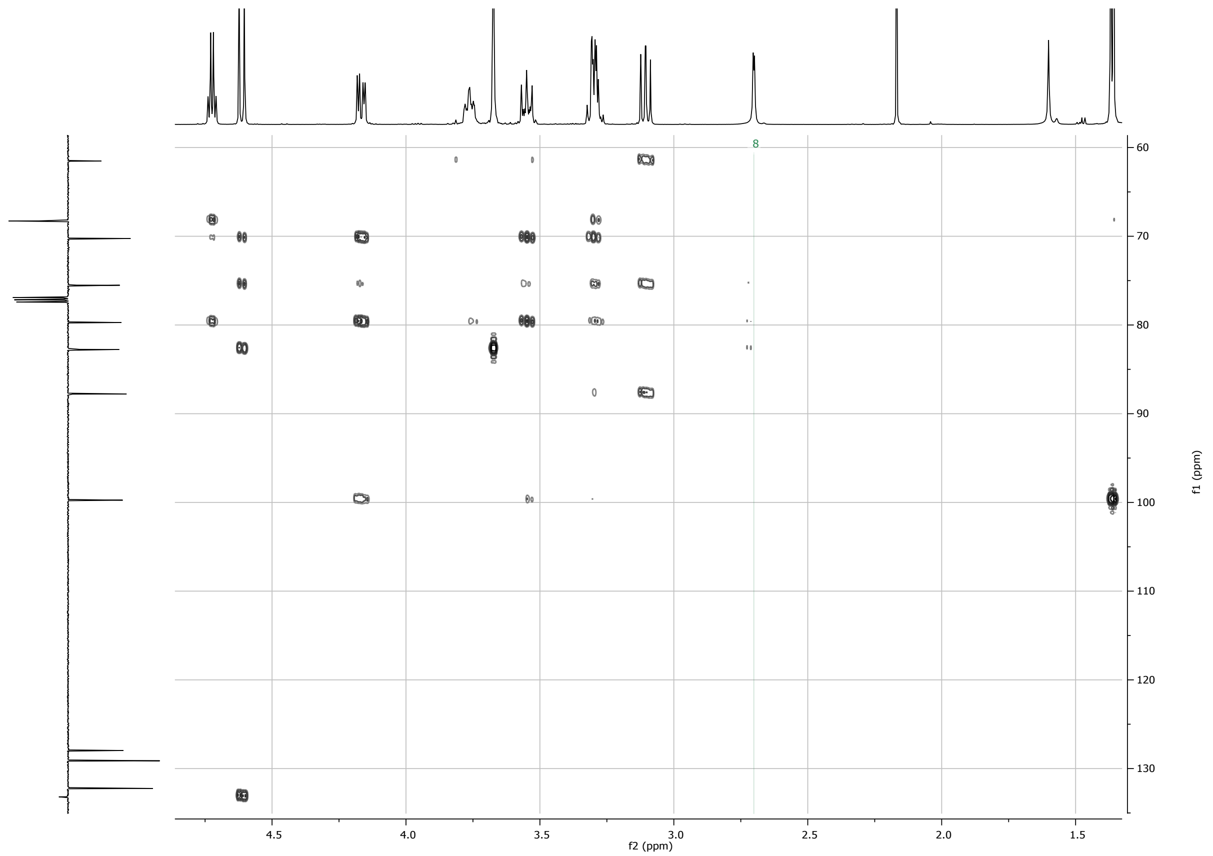

Supplementary Figure S132. HMBC NMR, $CDCl_3$ of compound **S21**

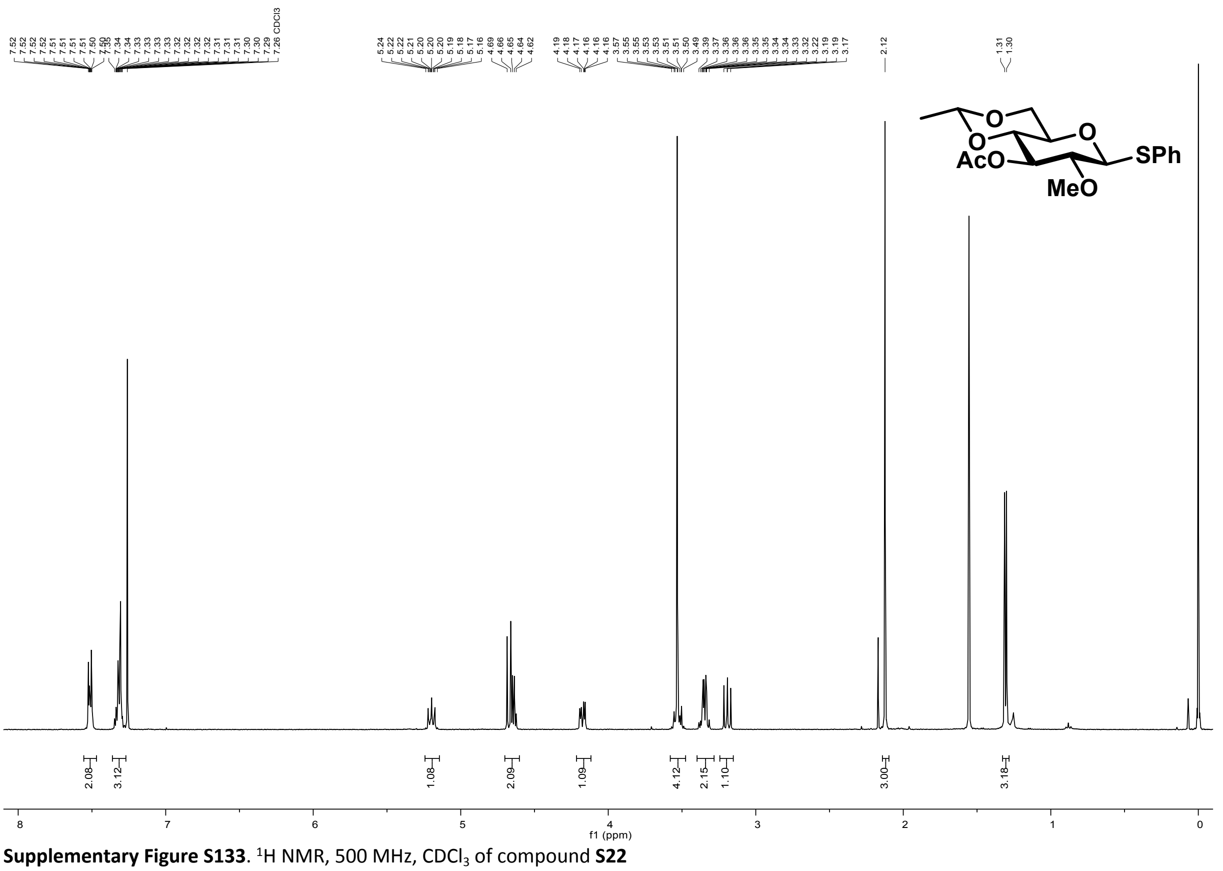

Supplementary Figure S133. ^1H NMR, 500 MHz, $CDCl_3$ of compound **S22**

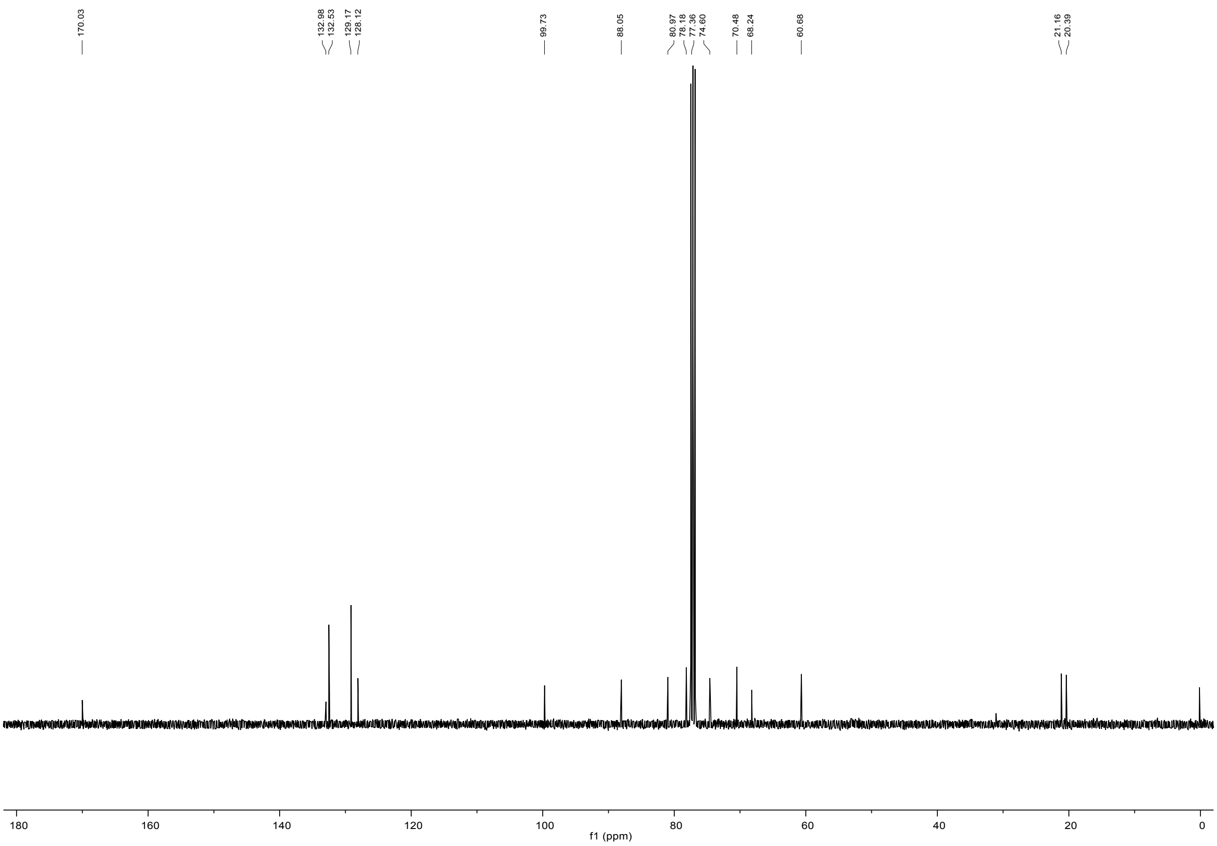

Supplementary Figure S134. $^{13}C\{^{1}H\}$ NMR, 126 MHz, $CDCl_3$ of compound **S22**

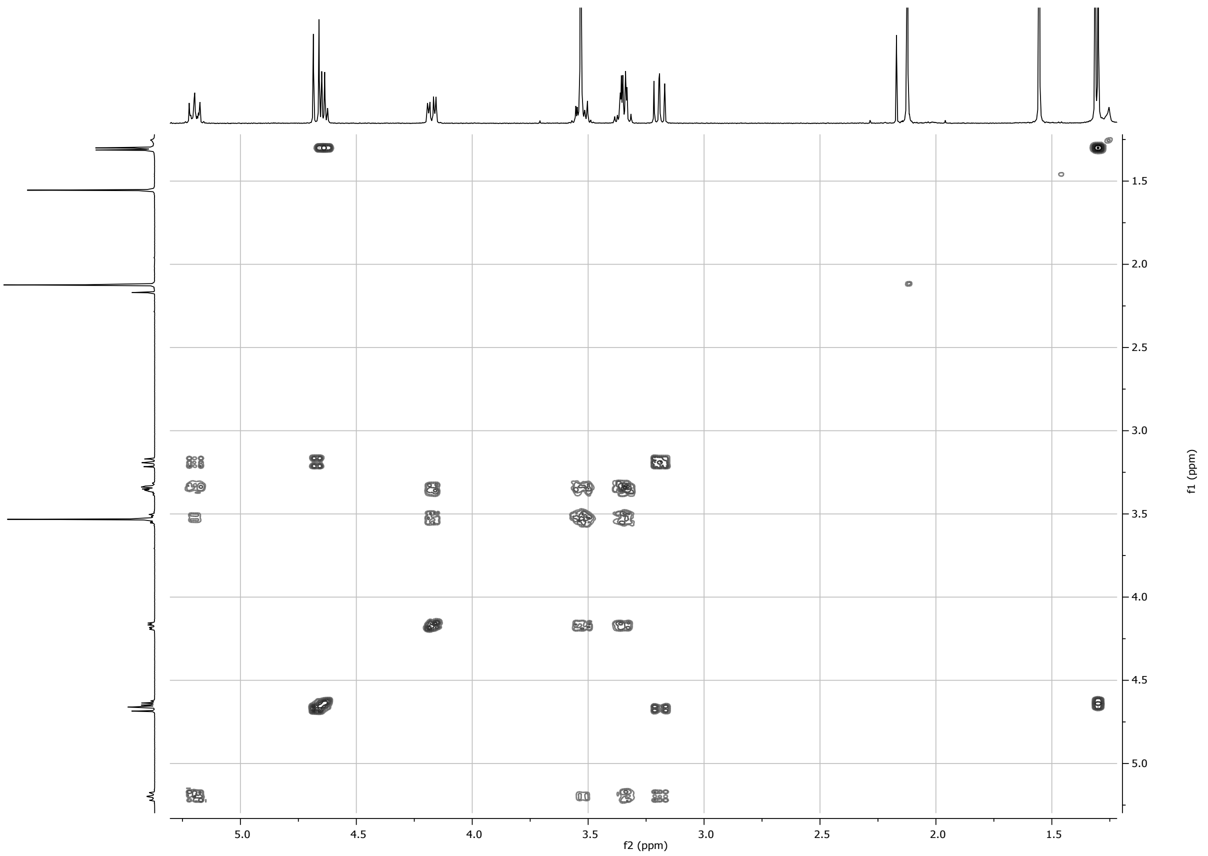

Supplementary Figure S135. HH-COSY NMR, $CDCl_3$ of compound **S22**

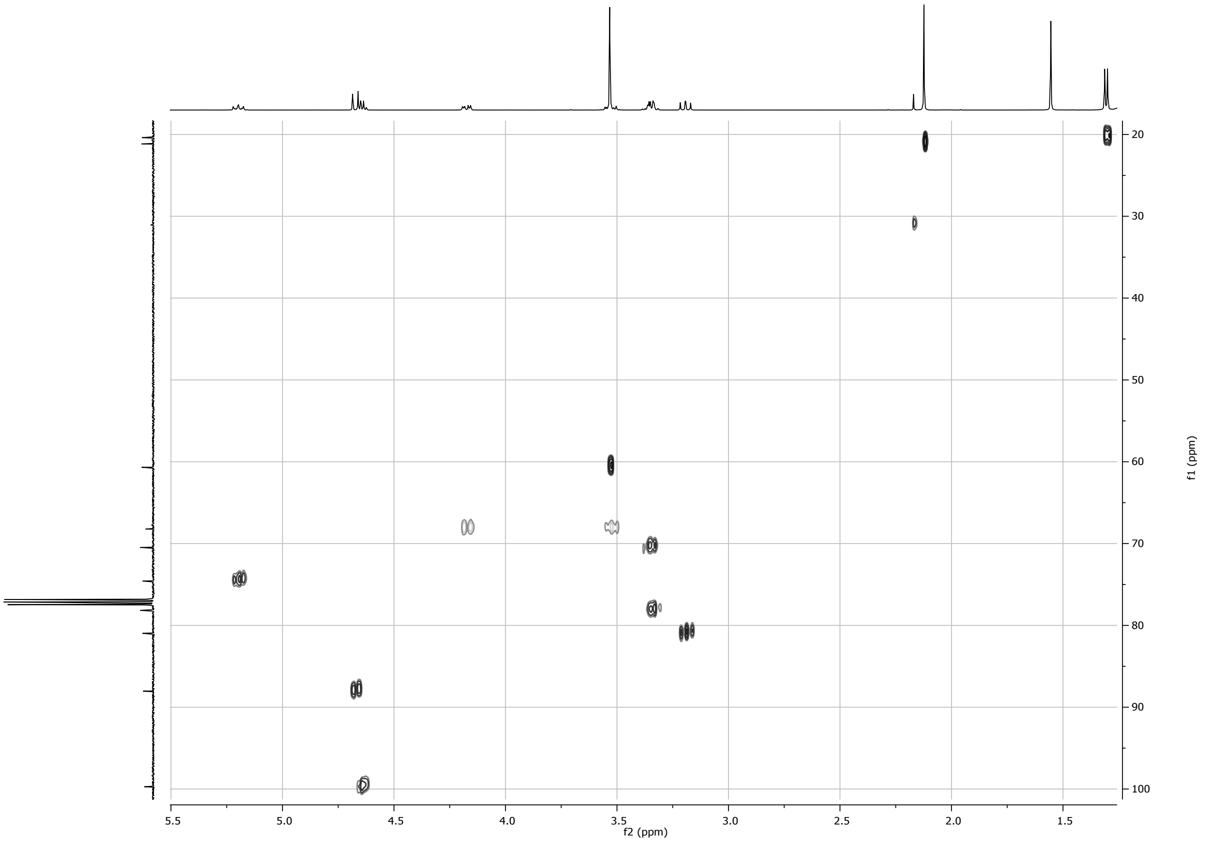

Supplementary Figure S136. HSQC{^{1}H} NMR, $CDCl_3$ of compound **S22**

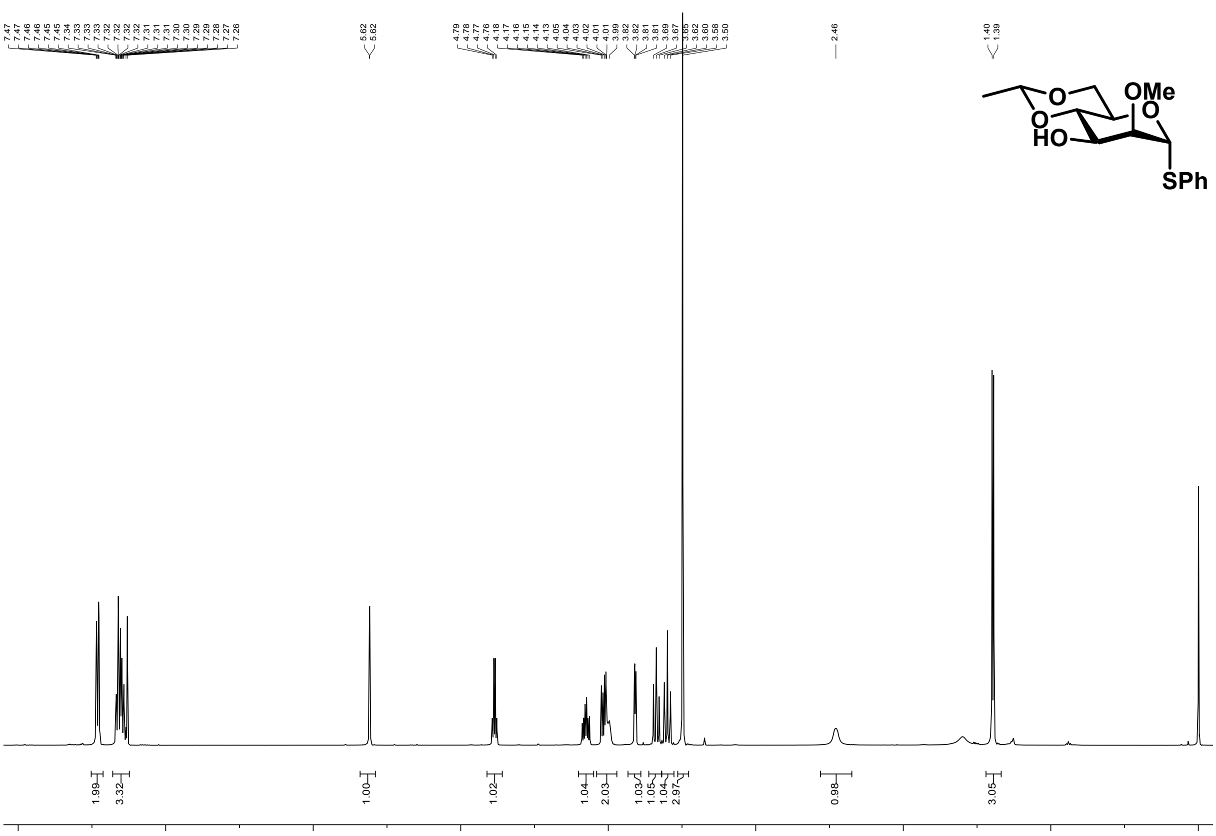

Supplementary Figure S137. ^{1}H NMR, 500 MHz, $CDCl_3$ of compound **S23**

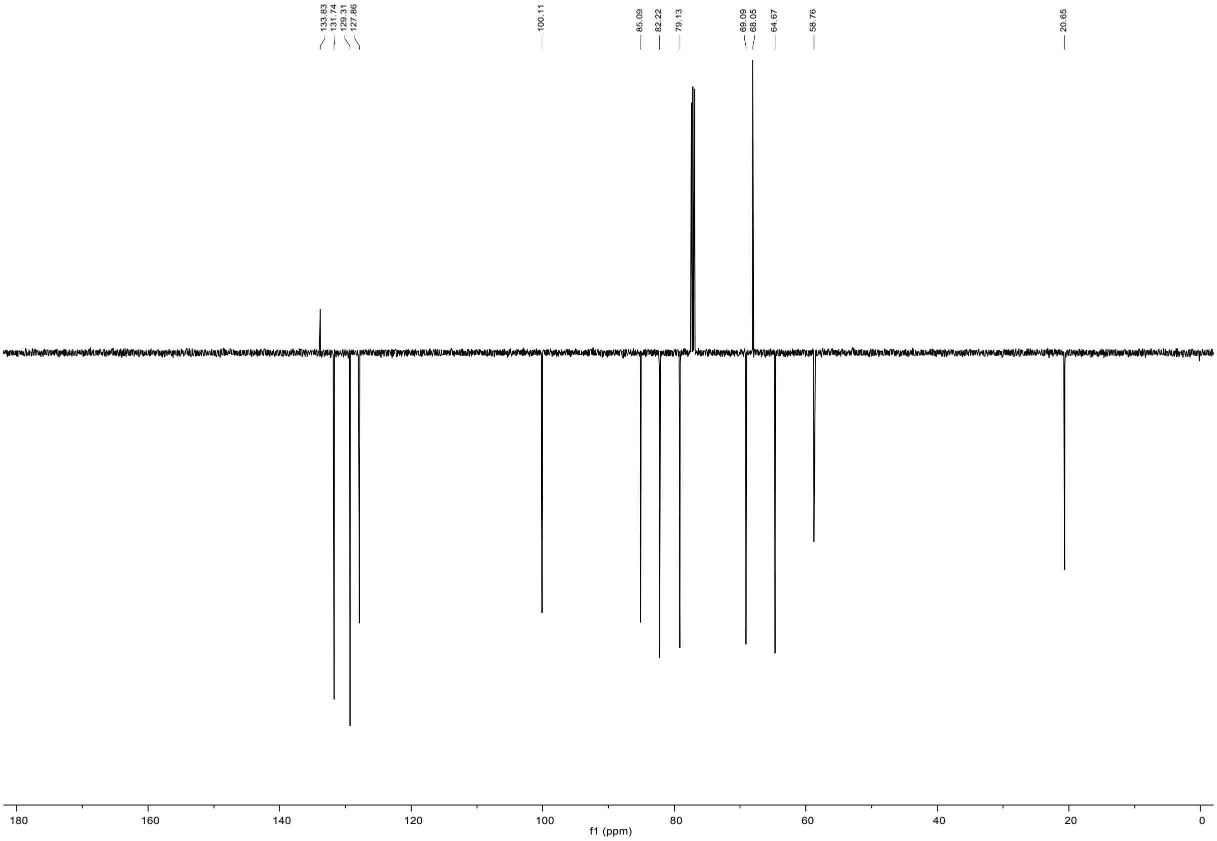

Supplementary Figure S138. $^{13}C\{^1H\}$ NMR, 126 MHz, $CDCl_3$ of compound **S23**

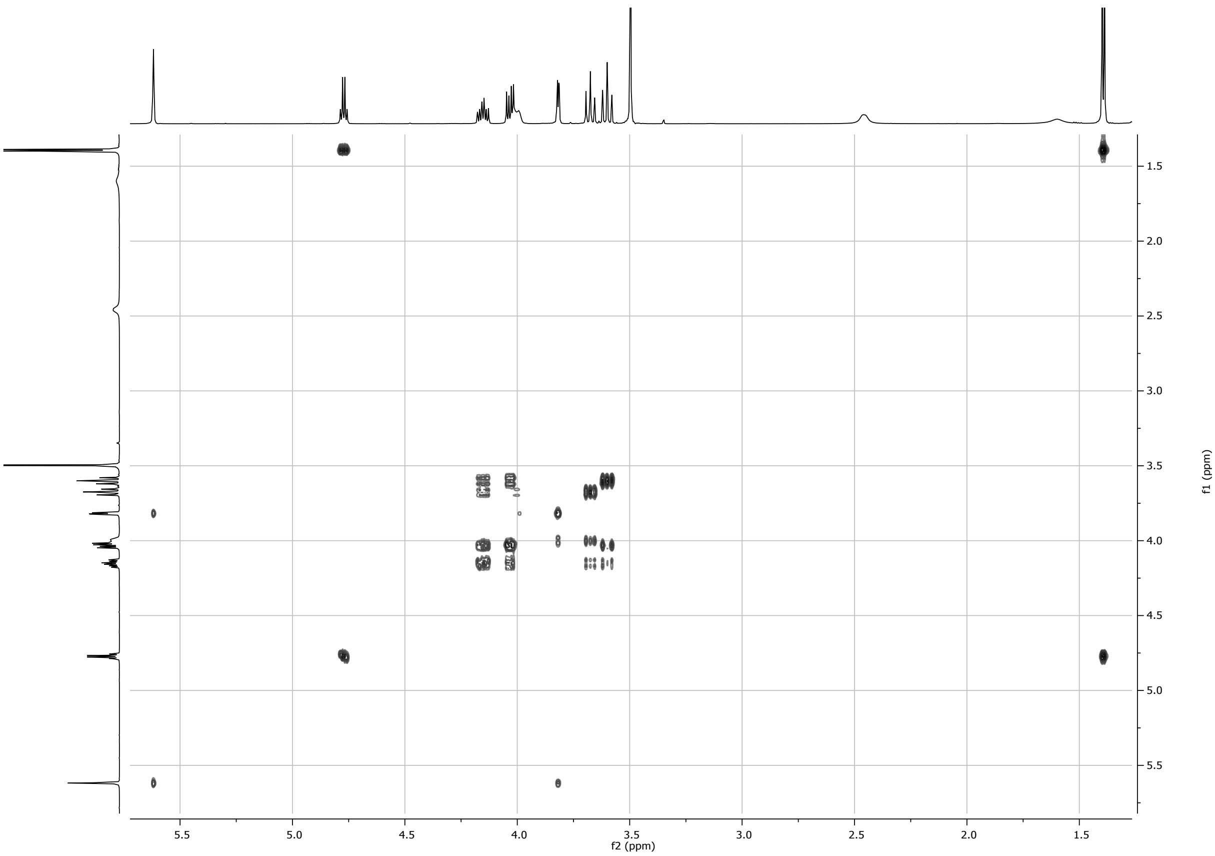

Supplementary Figure S139. HH-COSY NMR, $CDCl_3$ of compound **S23**

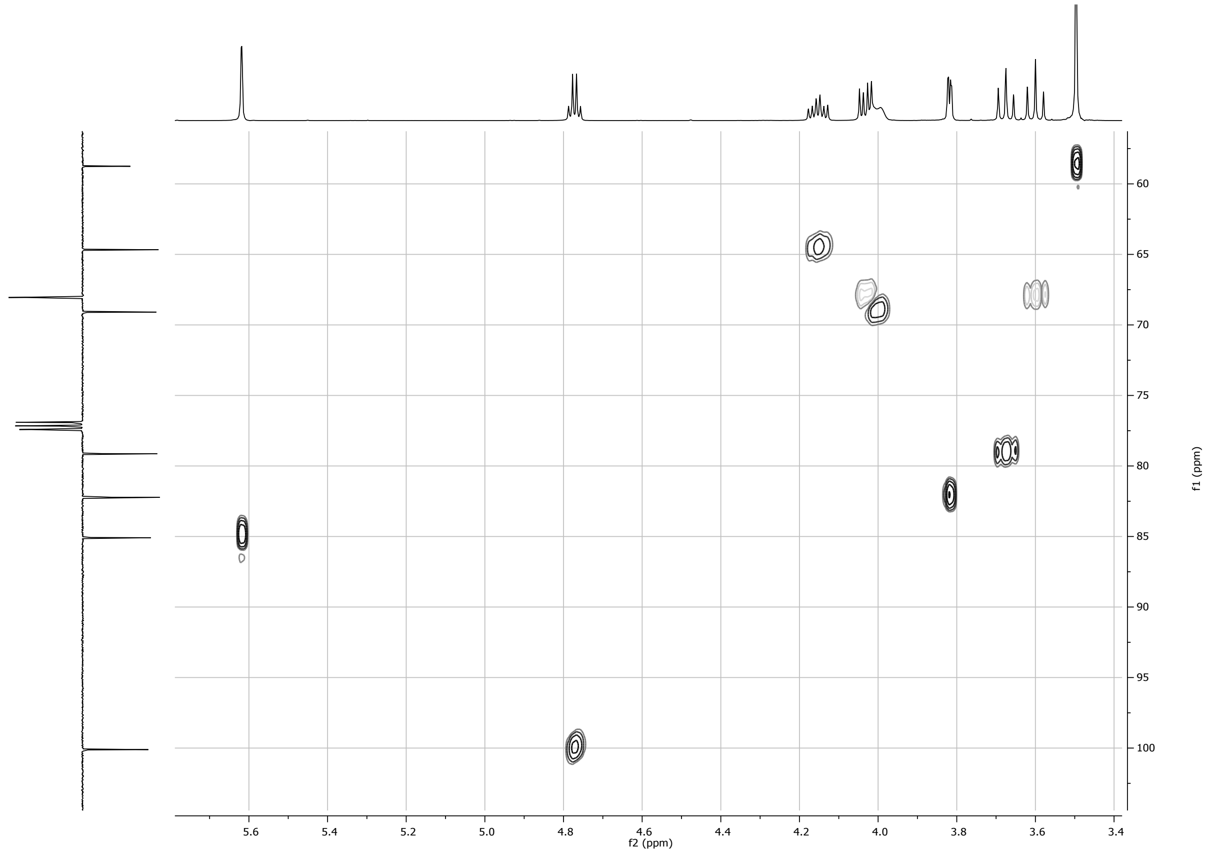

Supplementary Figure S140. HSQC{^{1}H} NMR, $CDCl_3$ of compound **S23**

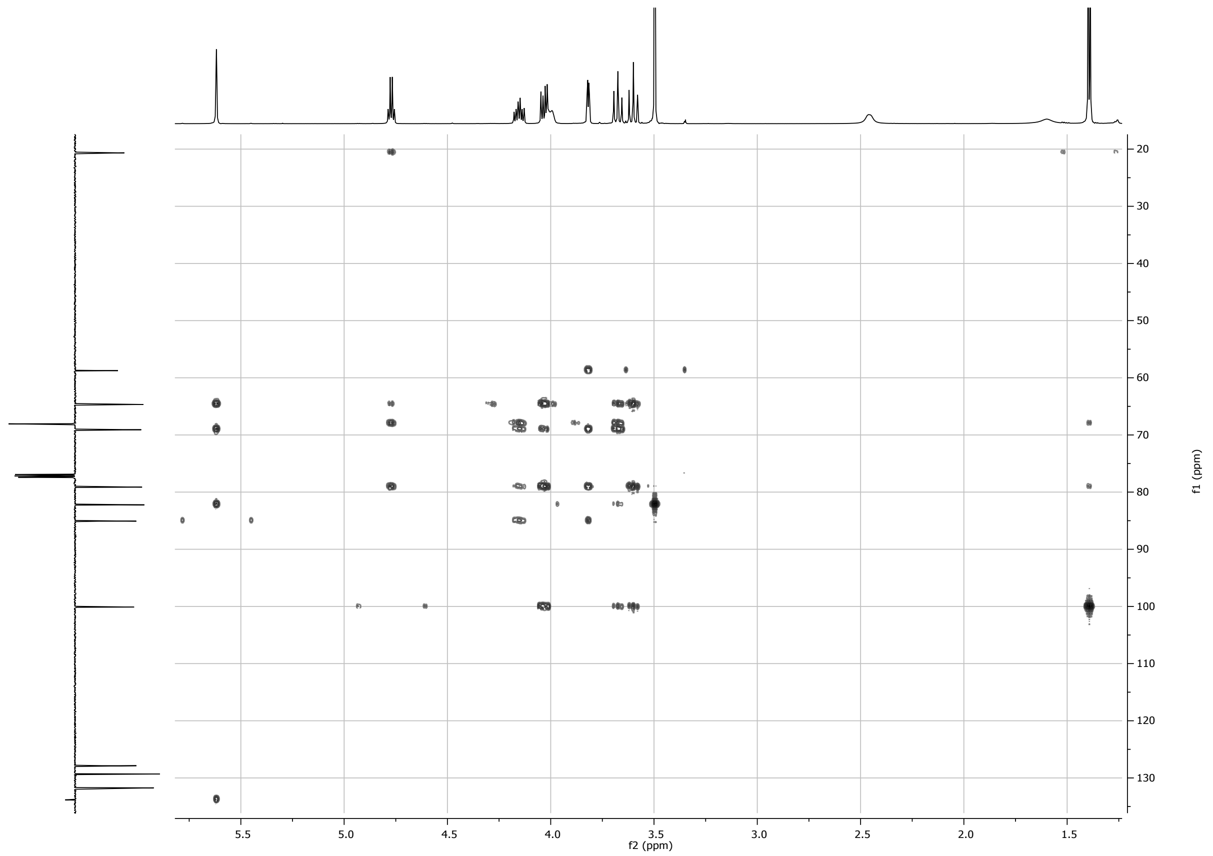

Supplementary Figure S141. HMBC NMR, $CDCl_3$ of compound **S23**

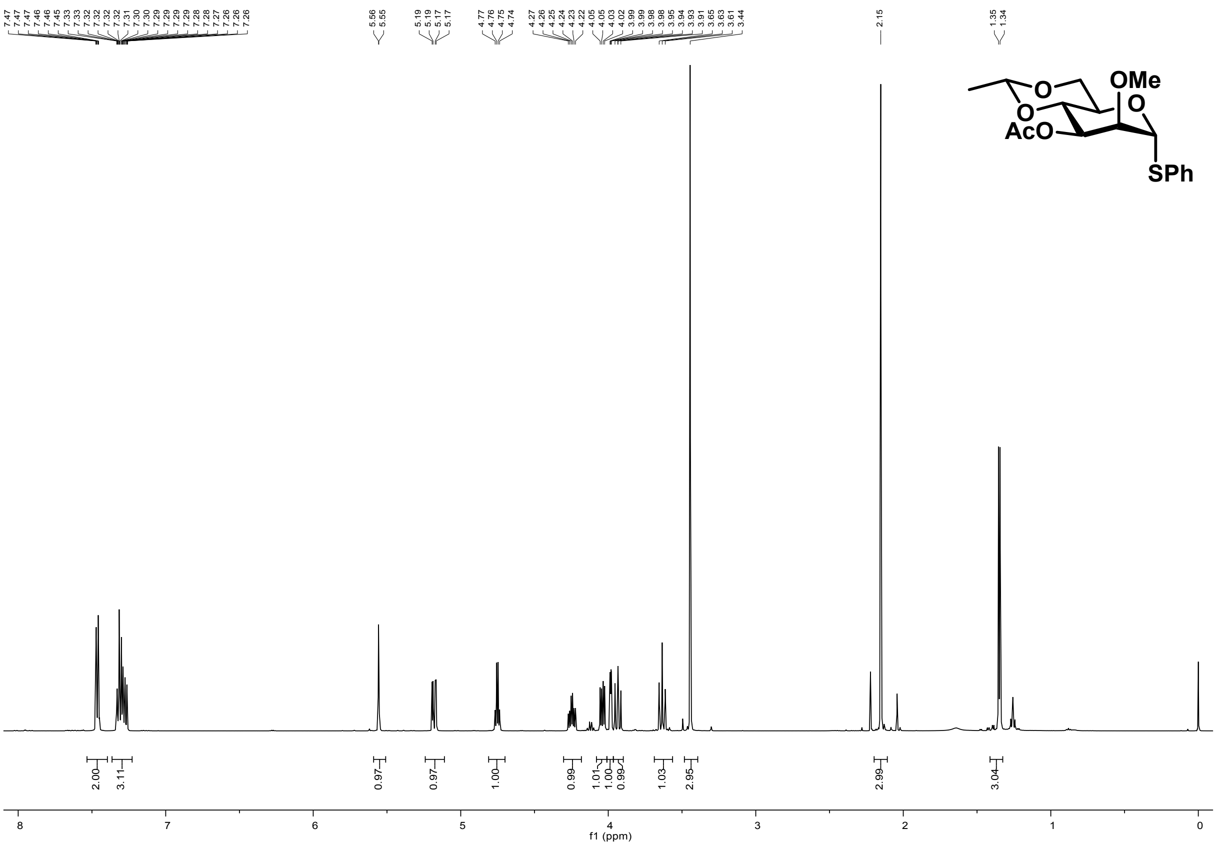

Supplementary Figure S142. ^{1}H NMR, 500 MHz, $CDCl_3$ of compound **S24**

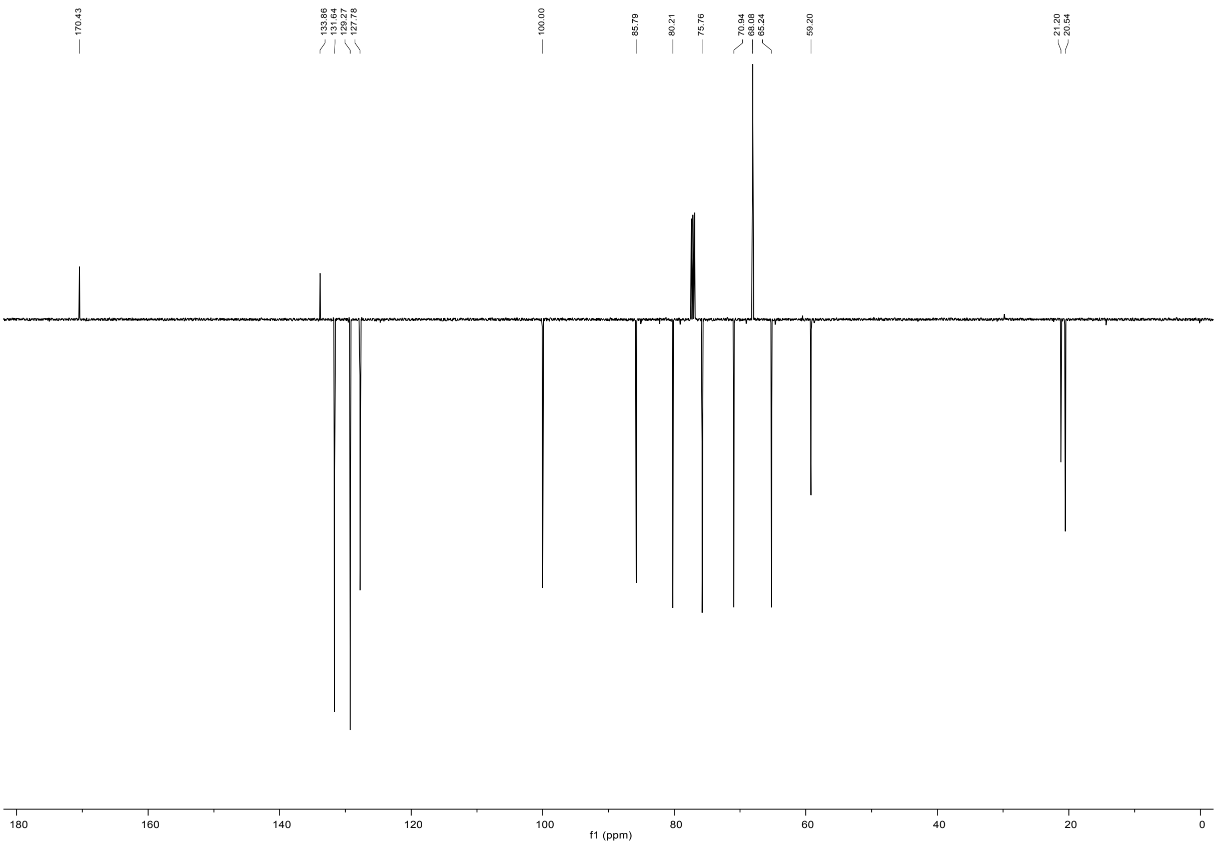

Supplementary Figure S143. $^{13}C\{^{1}H\}$ NMR, 126 MHz, $CDCl_3$ of compound **S24**

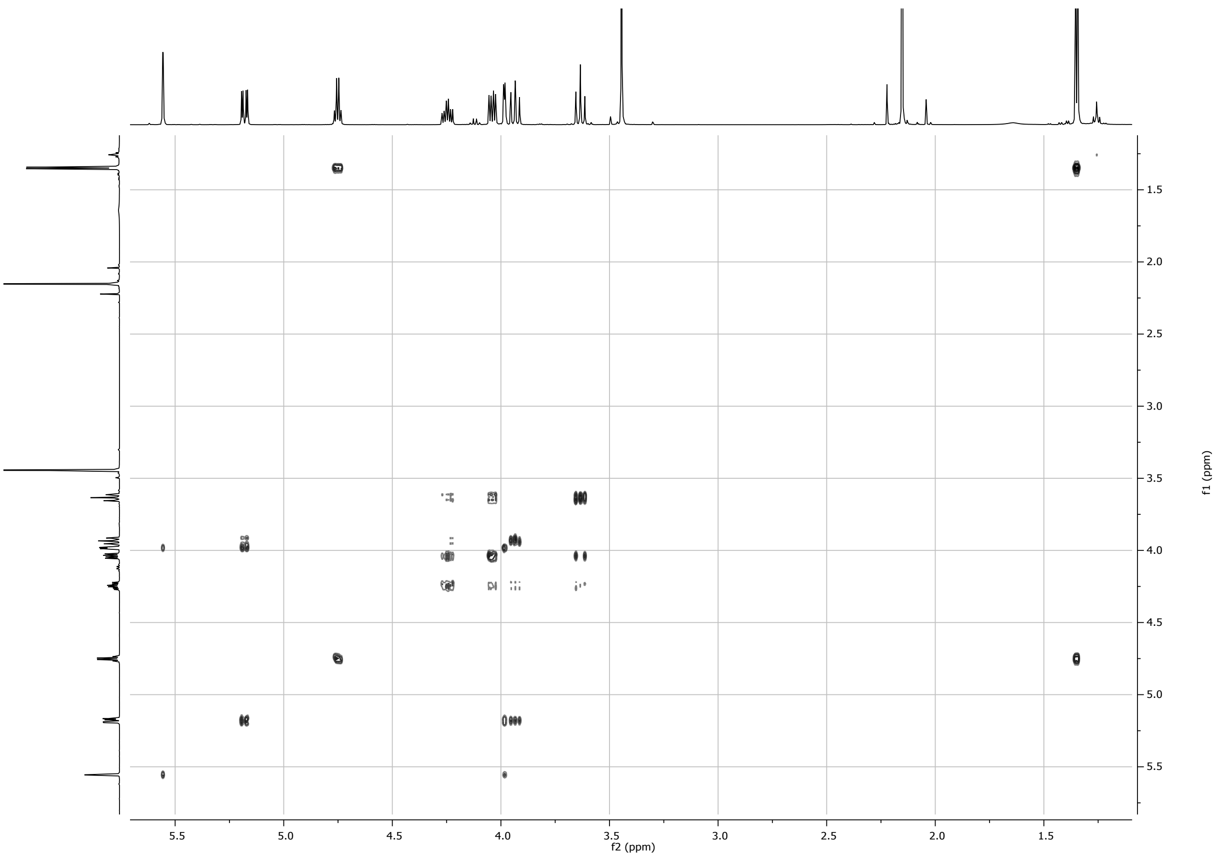

Supplementary Figure S144. HH-COSY NMR, $CDCl_3$ of compound **S24**

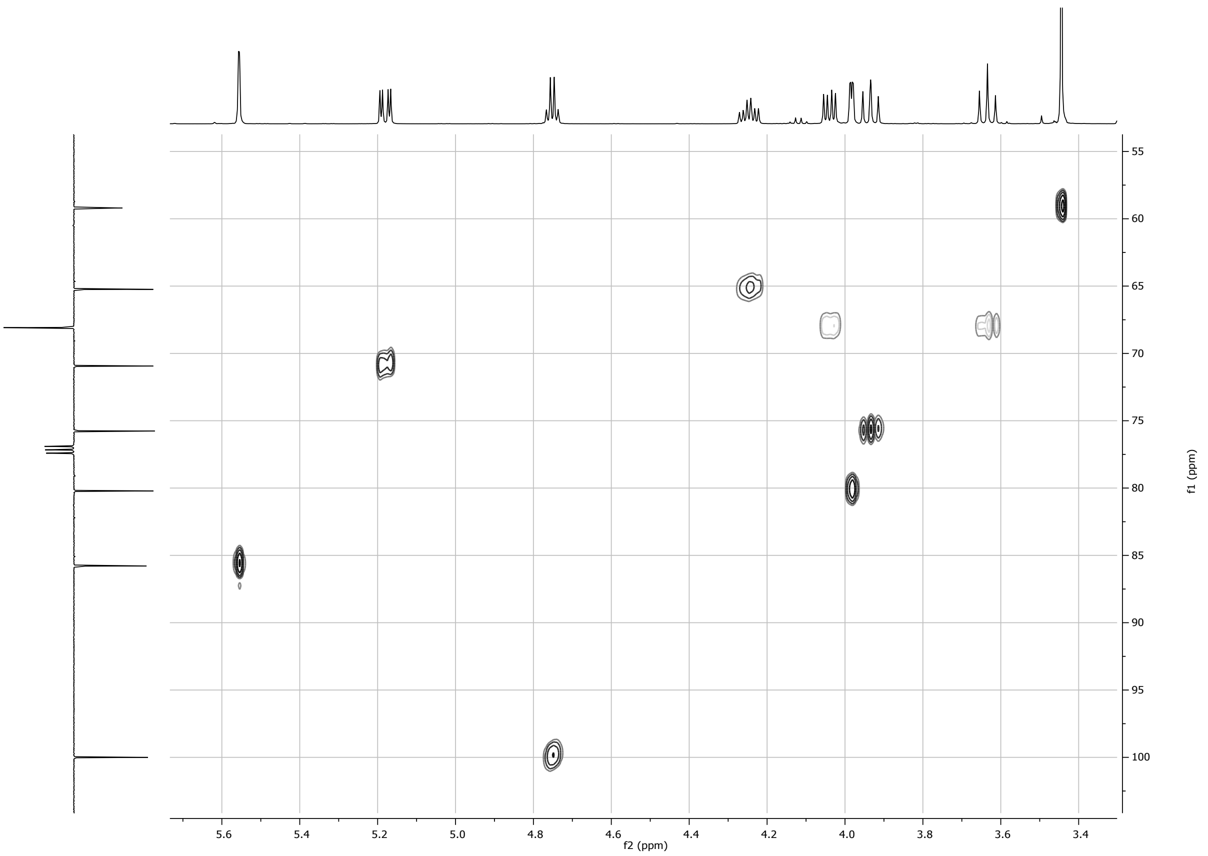

Supplementary Figure S145. HSQC{^{1}H} NMR, $CDCl_3$ of compound **S24**

Supplementary References

(1) Martens, J.; Berden, G.; Gebhardt, C. R.; Oomens, J. Infrared Ion Spectroscopy in a Modified Quadrupole Ion Trap Mass Spectrometer at the FELIX Free Electron Laser Laboratory. *Rev. Sci. Instrum.* **2016**, *87* (10), 103108. https://doi.org/10.1063/1.4964703.

(2) Oepts, D.; van der Meer, A. F. G.; van Amersfoort, P. W. The Free-Electron-Laser User Facility FELIX. *Infrared Phys. Technol.* **1995**, *36* (1), 297–308. https://doi.org/10.1016/1350-4495(94)00074-U.

(3) Berden, G.; Derksen, M.; Houthuijs, K. J.; Martens, J.; Oomens, J. An Automatic Variable Laser Attenuator for IRMPD Spectroscopy and Analysis of Power-Dependence in Fragmentation Spectra. *Int. J. Mass Spectrom.* **2019**, *443*, 1–8. https://doi.org/10.1016/j.ijms.2019.05.013.

(4) van Outersterp, R.E.; Houthuijs, K.J.; Berden, G.; Engelke, U.F.; Kluijtmans, L.A.J.; Wevers, R.A.; Coene, K.L.M.; Oomens, J.; Martens, J.; Molecular Spectroscopy (HIMS, FNWI). Reference-Standard Free Metabolite Identification Using Infrared Ion Spectroscopy. *Int. J. Mass Spectrom.* **2019**, *443*, 77–85. https://doi.org/10.1016/j.ijms.2019.05.015.

(5) Hansen, T.; Elferink, H.; van Hengst, J. M. A.; Houthuijs, K. J.; Remmerswaal, W. A.; Kromm, A.; Berden, G.; van der Vorm, S.; Rijs, A. M.; Overkleeft, H. S.; Filippov, D. V.; Rutjes, F. P. J. T.; van der Marel, G. A.; Martens, J.; Oomens, J.; Codée, J. D. C.; Boltje, T. J. Characterization of Glycosyl Dioxolenium Ions and Their Role in Glycosylation Reactions. *Nat. Commun.* **2020**, *11* (1), 2664. https://doi.org/10.1038/s41467-020-16362-x.

(6) Landrum, G.; Tosco, P.; Kelley, B.; Ric; sriniker; gedeck; Vianello, R.; NadineSchneider; Kawashima, E.; Dalke, A.; N, D.; Cole, B.; Cosgrove, D.; Swain, M.; Turk, S.; AlexanderSavelyev; Jones, G.; Vaucher, A.; Wójcikowski, M.; Probst, D.; Scalfani, V. F.; godin, guillaume; Pahl, A.; Berenger, F.; JLVarjo; Ujihara, K.; strets123; JP; DoliathGavid; Sforna, G. Rdkit/Rdkit: 2021_09_4 (Q3 2021) Release, 2022. https://doi.org/10.5281/zenodo.5835217.

(7) Frisch, M. J.; Trucks, G. W.; Schlegel, H. B.; Scuseria, G. E.; Robb, M. A.; Cheeseman, J. R.; Scalmani, G.; Barone, V.; Petersson, G. A.; Nakatsuji, H.; Li, X.; Caricato, M.; Marenich, A. V.; Bloino, J.; Janesko, B. G.; Gomperts, R.; Mennucci, B.; Hratchian, H. P.; Ortiz, J. V.; Izmaylov, A. F.; Sonnenberg, J. L.; Williams; Ding, F.; Lipparini, F.; Egidi, F.; Goings, J.; Peng, B.; Petrone, A.; Henderson, T.; Ranasinghe, D.; Zakrzewski, V. G.; Gao, J.; Rega, N.; Zheng, G.; Liang, W.; Hada, M.; Ehara, M.; Toyota, K.; Fukuda, R.; Hasegawa, J.; Ishida, M.; Nakajima, T.; Honda, Y.; Kitao, O.; Nakai, H.; Vreven, T.; Throssell, K.; Montgomery Jr., J. A.; Peralta, J. E.; Ogliaro, F.; Bearpark, M. J.; Heyd, J. J.; Brothers, E. N.; Kudin, K. N.; Staroverov, V. N.; Keith, T. A.; Kobayashi, R.; Normand, J.; Raghavachari, K.; Rendell, A. P.; Burant, J. C.; Iyengar, S. S.; Tomasi, J.; Cossi, M.; Millam, J. M.; Klene, M.; Adamo, C.; Cammi, R.; Ochterski, J. W.; Martin, R. L.; Morokuma, K.; Farkas, O.; Foresman, J. B.; Fox, D. J. Gaussian 16 Rev. C.01, 2016.

(8) Frisch, M. J.; Trucks, G. W.; Cheeseman, J. R.; Scalmani, G.; Caricato, M.; Hratchian, H. P.; Li, X.; Barone, V.; Bloino, J.; Zheng, G.; Vreven, T.; Montgomery, J. A.; Petersson, G. A.; Scuseria, G. E.; Schlegel, H. B.; Nakatsuji, H.; Izmaylov, A. F.; Martin, R. L.; Sonnenberg, J. L.; Peralta, J. E.; Heyd, J. J.; Brothers, E.; Ogliaro, F.; Bearpark, M.; Robb, M. A.; Mennucci, B.; Kudin, K. N.; Staroverov, V. N.; Kobayashi, R.; Normand, J.; Rendell, A.; Gomperts, R.; Zakrzewski, V. G.; Hada, M.; Ehara, M.; Toyota, K.; Fukuda, R.; Hasegawa, J.; Ishida, M.; Nakajima, T.; Honda, Y.; Kitao, O.; Nakai, H. Gaussian 09 Rev. D.01.

(9) Spartan'14.

(10) Halgren, T. A. Merck Molecular Force Field. I. Basis, Form, Scope, Parameterization, and Performance of MMFF94. *J. Comput. Chem.* **1996**, *17* (5–6), 490–519. https://doi.org/10.1002/(SICI)1096-987X(199604)17:5/6<490::AID-JCC1>3.0.CO;2-P.

(11) Hansen, T.; Lebedel, L.; Remmerswaal, W. A.; van der Vorm, S.; Wander, D. P. A.; Somers, M.; Overkleeft, H. S.; Filippov, D. V.; Désiré, J.; Mingot, A.; Bleriot, Y.; van der Marel, G. A.; Thibaudeau, S.; Codée, J. D. C. Defining the SN1 Side of Glycosylation Reactions: Stereoselectivity of Glycopyranosyl Cations. *ACS Cent. Sci.* **2019**, *5* (5), 781–788. https://doi.org/10.1021/acscentsci.9b00042.

(12) Ribeiro, R. F.; Marenich, A. V.; Cramer, C. J.; Truhlar, D. G. Use of Solution-Phase Vibrational Frequencies in Continuum Models for the Free Energy of Solvation. *J. Phys. Chem. B* **2011**, *115* (49), 14556–14562. https://doi.org/10.1021/jp205508z.

(13) Luchini, G.; Alegre-Requena, J. V.; Funes-Ardoiz, I.; Paton, R. S. GoodVibes: Automated Thermochemistry for Heterogeneous Computational Chemistry Data. *F1000Research* **2020**, *9*, 291. https://doi.org/10.12688/f1000research.22758.1.

(14) OriginPro, 9.0.0.

(15) de Kleijne, F. F. J.; ter Braak, F.; Piperoudis, D.; Moons, P. H.; Moons, S. J.; Elferink, H.; White, P. B.; Boltje, T. J. Detection and Characterization of Rapidly Equilibrating Glycosylation Reaction Intermediates Using Exchange NMR. *J. Am. Chem. Soc.* **2023**. https://doi.org/10.1021/jacs.3c08709.

(16) Vorm, S. van der; Hansen, T.; S. Overkleeft, H.; Marel, G. A. van der; C. Codée, J. D. The Influence of Acceptor Nucleophilicity on the Glycosylation Reaction Mechanism. *Chem. Sci.* **2017**, *8* (3), 1867–1875. https://doi.org/10.1039/C6SC04638J.

(17) Remmerswaal, W. A.; Houthuijs, K. J.; van de Ven, R.; Elferink, H.; Hansen, T.; Berden, G.; Overkleeft, H. S.; van der Marel, G. A.; Rutjes, F. P. J. T.; Filippov, D. V.; Boltje, T. J.; Martens, J.; Oomens, J.; Codée, J. D. C. Stabilization of Glucosyl Dioxolenium Ions by "Dual Participation" of the 2,2-Dimethyl-2-(*Ortho*-Nitrophenyl)Acetyl (DMNPA) Protection Group for 1,2-*Cis*-Glucosylation. *J. Org. Chem.* **2022**, *87* (14), 9139–9147. https://doi.org/10.1021/acs.joc.2c00808.
